# Exploiting rhodium-catalysed ynamide hydroacylation as a platform for divergent heterocycle synthesis†
†Electronic supplementary information (ESI) available: Full experimental procedures and characterisation for all compounds. See DOI: 10.1039/c7sc03795c


**DOI:** 10.1039/c7sc03795c

**Published:** 2017-10-05

**Authors:** Robert N. Straker, Manjeet K. Majhail, Michael C. Willis

**Affiliations:** a Department of Chemistry , University of Oxford , Chemistry Research Laboratory , Mansfield Road , Oxford , OX1 3TA , UK . Email: michael.willis@chem.ox.ac.uk

## Abstract

The first examples of ynamide hydroacylation are described. Using rhodium catalysis, linear β-enaminone products are generated in high yield and excellent regioselectivity from the combination of aldehydes and ynamides. The enaminone products are subsequently used as a platform to construct a diverse array of substituted pyrazoles, pyrimidines, and isoxazoles in a two-step, one-pot sequence. It was found that with judicious choice of catalyst system it was possible to overturn the regioselectivity of the hydroacylation reaction to generate α-enaminone products.

## Abstract 2

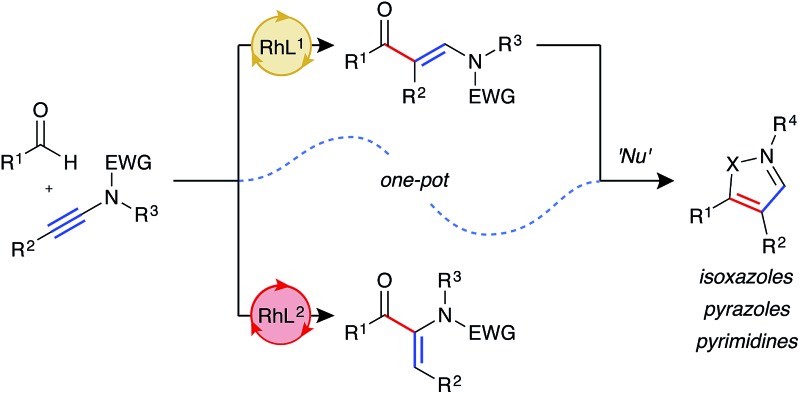
The first examples of ynamide hydroacylation are described. The choice of ligand system determines reaction regioselectivity, resulting in α- and β-enaminones. The latter are transformed into a variety of N-heterocycles.

## Introduction

**Scheme 1:**
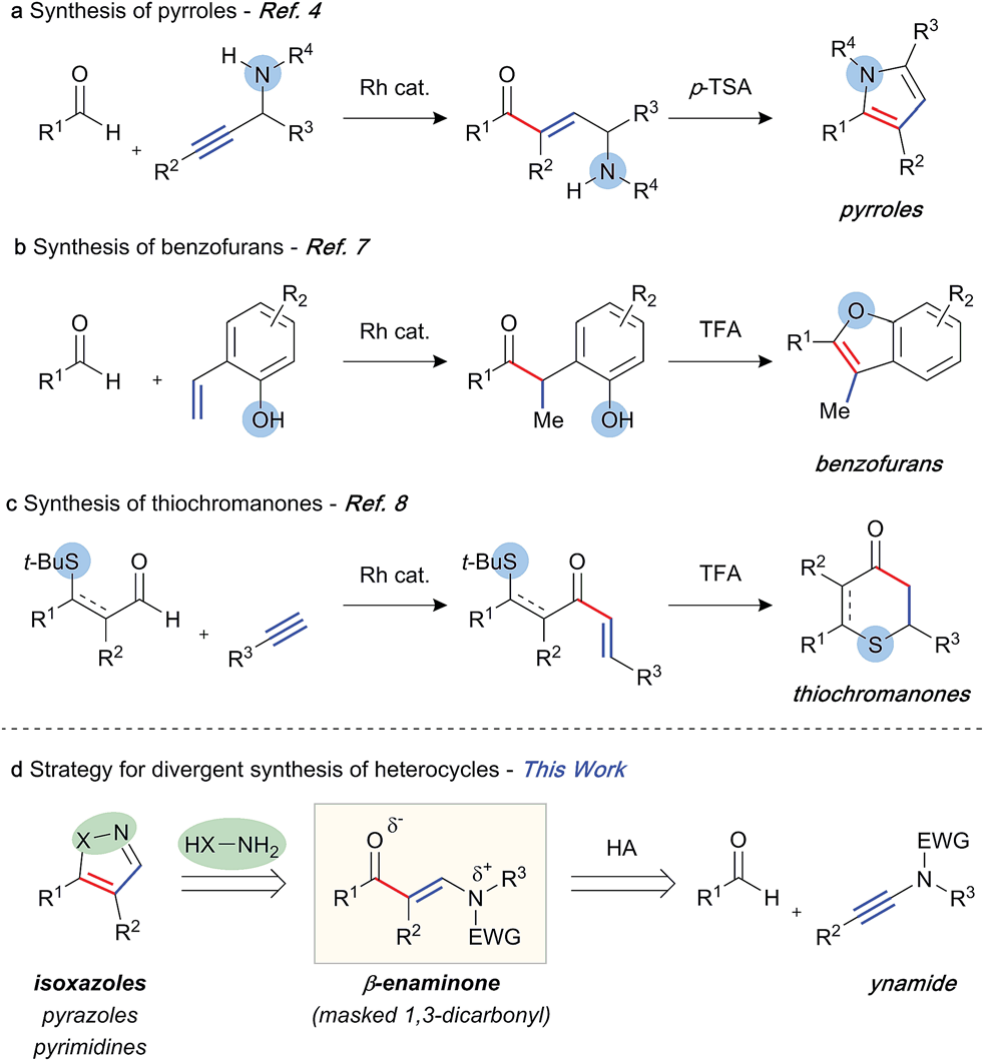
Hydroacylation strategies towards heterocycle synthesis.

The abundance of heterocycles in natural products and biologically active compounds has made them prime targets for the synthetic community.^1^ Despite many classical syntheses, the ability to construct these motifs in an efficient and atom-economical manner is of the utmost importance.^2^ Hydroacylation reactions enable the rapid assembly of diversely substituted carbonyl compounds, which can be further transformed into heterocycles.^3^ In this context, a number of strategies have been explored. Our laboratory has previously reported the synthesis of pyrroles,^4^ furans,^5^ and quinolines^6^ through intermolecular hydroacylation of alkynes, and subsequent intramolecular cyclisation of the enone product with a pendant nucleophile (Scheme 1a). The Dong group employed a similar tactic, in their report of vinylphenol-directed hydroacylation, to construct benzofurans *via* a cyclocondensation reaction (Scheme 1b).^7^ An alternative approach has been to incorporate the directing group, used to control the hydroacylation reaction and present in the aldehyde component, in the heterocyclic products (Scheme 1c). This method has been used to great effect to generate thiochroman-4-ones,^8^ 4-quinolones, and chroman-4-ones.^9^ Although elegant, each of these previous syntheses required a specific substrate class in order to construct the desired heterocycle, as the “heteroatom” of each specific heterocycle is pre-installed in the hydroacylation product. We envisaged a conceptually new strategy, in which diverse heterocycles could be prepared from a single hydroacylation-derived scaffold; crucially, the heteroatom(s) of the heterocycle would be introduced using an initial intermolecular step (Scheme 1d). β-Enaminones serve as dipolar 1,3-dicarbonyl surrogates with defined reactivity,^10^ and as such they have been used in the synthesis of various valuable heterocycles, including uniquely substituted isoxazoles,^11^ pyrazoles,^12^ and pyrimidines.^13^ Typically, the synthesis of enaminones is achieved by the condensation of ketones with amides, which requires forcing conditions, and thus is limited to a small range of substituents and functional groups. This route also presents regioselectivity issues with ketones containing more than one enolisable position.

We proposed an unprecedented disconnection of the enaminone acyl-enamine bond, which could be achieved synthetically *via* the hydroacylation of an ynamide. The field of ynamide chemistry has burgeoned over the past decade owing to the unique properties and reactivity of this motif.^14^ Although ynamides have been employed in a number of transition metal-catalysed transformations, and the related enamides have previously been demonstrated as efficient hydroacylation substrates,^15^ ynamides remain novel substrates for hydroacylation reactions. In this capacity, ynamides have the potential to provide modular access to highly substituted enaminone products, and thus provide a platform for heterocycle synthesis. Herein, we report rhodium-catalysed intermolecular ynamide hydroacylation, and the synthesis of 4,5-disubstituted isoxazoles *via* a one-pot hydroacylation/cyclisation sequence. We also show the potential of this method in the formation of pyrazoles and pyrimidines.

## Results and discussion

We began our investigation with *S*-substituted aldehyde **1a** (Table 1), which we have previously shown to be an excellent substrate in a range of rhodium-catalysed alkene and alkyne hydroacylation reactions.^16^ Conscious of the requirements for subsequent heterocycle formation reactions, we chose to examine *N*-tosyl-aniline ynamides, as the resultant amine functionality would readily act as a leaving group. Ynamide **2a** was submitted to rhodium-catalysed hydroacylation conditions with diphosphine ligands with varying bite-angle. Narrow bite-angle ligands dcpm and dppm, known to efficiently promote intermolecular alkene and alkyne hydroacylation,^17^ exhibited modest reactivity after 16 hours at 55 °C (entries 1 and 2). The electron-rich alkyl phosphine dcpm displayed a small preference for the linear β-enaminone product **3a** over the branched α-enaminone **4a** (2 : 1 rr). However, aryl phosphine dppm generated product **3a** as a single regioisomer (>20 : 1). Maintaining a narrow bite angle but varying the nature of the tether, PNP(Cy) led to greatly increased reactivity and enhanced regioselectivity for α-enaminone **4a** (1 : 2.5 rr) which was isolated in 60% yield (entry 3). Unfortunately, the aryl phosphine variant PNP(Ph) was not effective in promoting the reaction (entry 4). Increasing bite-angle with dcpe and dppe ligands, employed by Bosnich in intramolecular hydroacylation of cyclopentanones,^18^ resulted in lower levels of catalyst activity, but continued the trend of regioselectivity observed with alkyl and aryl phosphines (1 : 1 and >20 : 1 rr respectively, entries 5 and 6). Increasing bite-angle further with dppp and dppb resulted in loss of catalyst activity (entries 10 and 11). We next turned to ligands possessing hemi-labile *O*-tethers, which are known to minimise unwanted reductive decarbonylation in reactions of alkynes.^19^ DCEphos was found to be inactive (entry 13), however, DPEphos returned the catalyst activity, with the starting material entirely consumed after 16 hours, and the linear product **3a** isolated in 90% yield (>20 : 1 rr, entry 14). Xantphos was not effective in promoting the reaction, which could perhaps be attributed to the reduced conformational freedom of the ligand backbone (entry 15).

**Table 1:** Optimisation of ynamide hydroacylation^*a*^

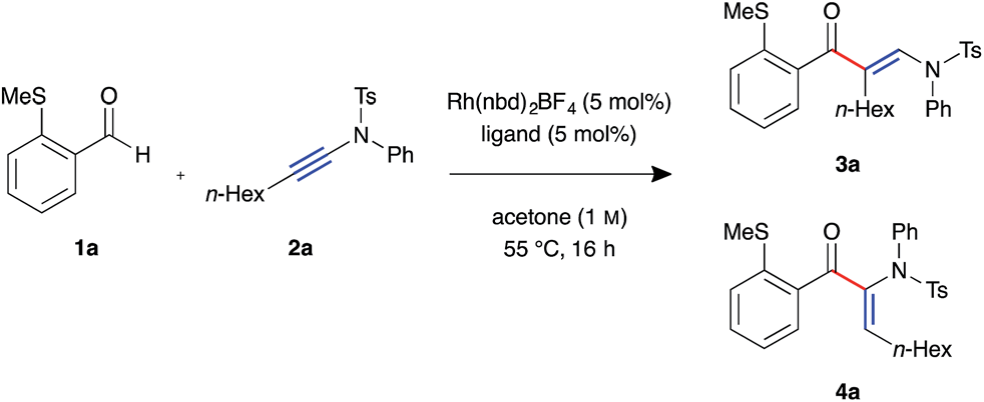			
Entry	Ligand	Yield^*b*^/%	**3a** : **4a**^*b*^
1	dcpm	28	2 : 1
2	dppm	36	>20 : 1
3	PNP(Cy)	92 (60)^*c*^	1 : 2.5
4	PNP(Ph)	0	—
5	dcpe	30	1 : 1
6	dppe	46	>20 : 1
7	dape	36	17 : 1
8	dtfpe	23	17 : 1
9	dppe(*o*-^*i*^Pr)	0	—
10	dppp	0	—
11	dppb	0	—
12	dppf	26	3 : 1
13	DCEphos	0	—
14	DPEphos	93 (90)^*d*^	>20 : 1
15	Xantphos	0	—
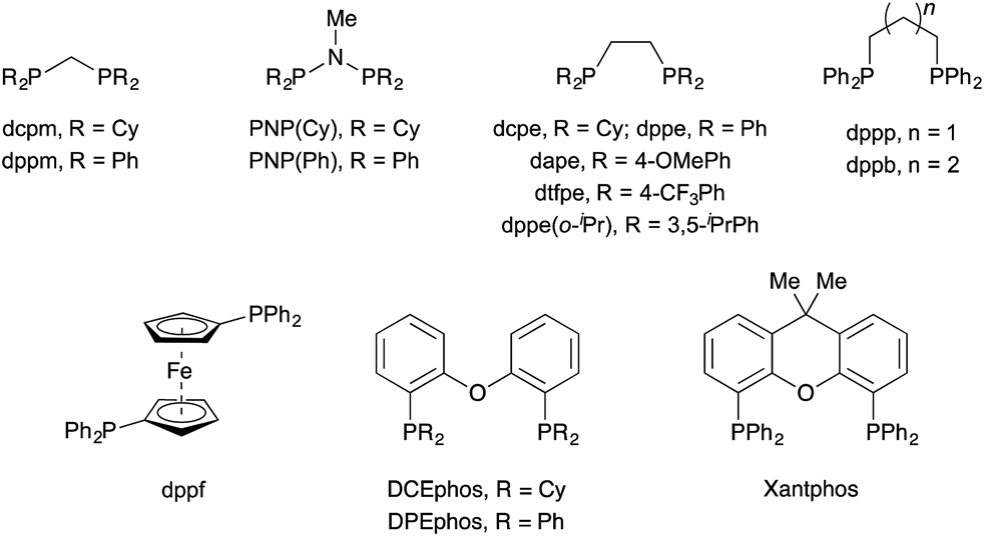			

^*a*^Reaction conditions: Rh(nbd)_2_BF_4_ (5 mol%), ligand (5 mol%), aldehyde (0.3 mmol, 1.0 equiv.), ynamide (1.1 equiv.), acetone (1.0 M), 55 °C for 16 h.

^*b*^Determined by ^1^H NMR spectroscopic analysis of the crude reaction mixture, using 1,3,5-trimethoxybenzene as the internal standard.

^*c*^Isolated yield of **4a**.

^*d*^Isolated yield of **3a**.

In order to elucidate the origin of the observed change in regioselectivity between alkyl and aryl phosphines, electron-rich and electron-poor aryl phosphine ligands dape and dtfpe were tested (entries 7 and 8). However, both ligands led to the generation of the β-enaminone product **3a** with identical selectivity (17 : 1 rr). The more sterically encumbered analogue dppe(*o*-^*i*^Pr) was ineffectual in the reaction (entry 9), with no product formation observed.

**Scheme 2:**
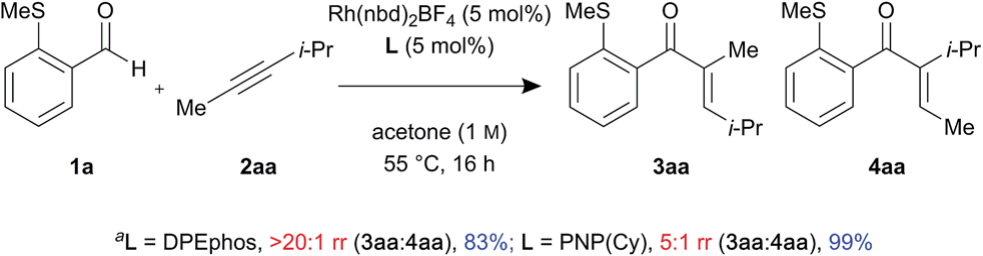
Control experiments with sterically biased internal alkyne (^*a*^determined by ^1^H NMR spectroscopic analysis of the crude reaction mixture, using 1,3,5-trimethoxybenzene as the internal standard).

In addition, control experiments were performed with an electronically neutral but sterically biased internal alkyne **2aa**, using DPEphos and PNP(Cy) ligands (Scheme 2). Interestingly, the linear enone product **3aa** was formed as a single regioisomer in the presence of DPEphos (>20 : 1 rr). However, in contrast to the ynamide substrate which gave branched selectivity with PNP(Cy) (1 : 2.5 rr), the alkyne substrate led to the linear product being formed but with lower regioselectivity (5 : 1 rr).

**Scheme 3:**
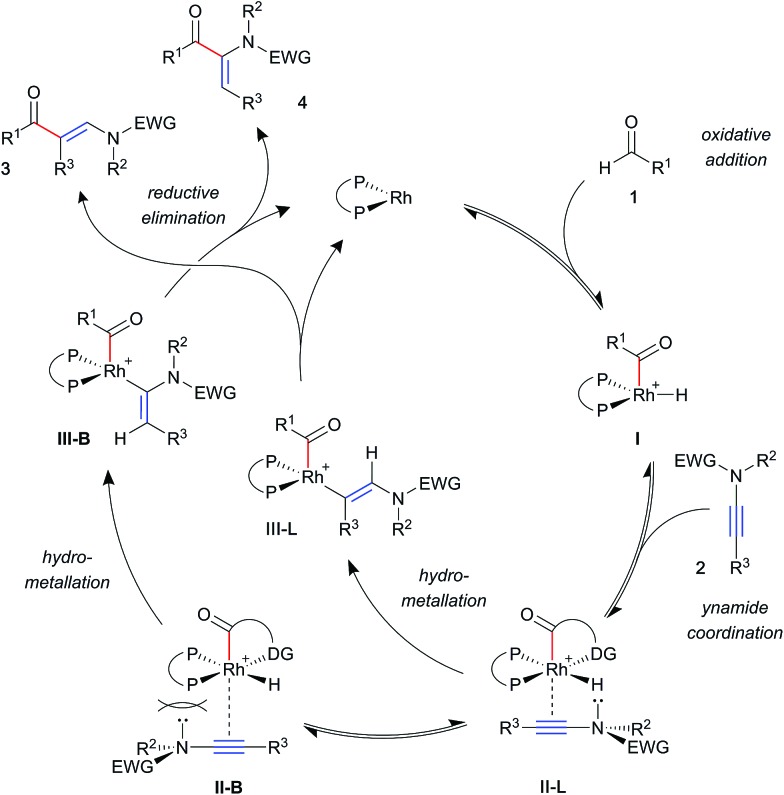
Proposed mechanism for regioselectivity in ynamide hydroacylation.

A general mechanism for ynamide hydroacylation, based on these results and our previous studies of alkene and alkyne systems,17*b* is illustrated in Scheme 3. Upon ynamide coordination, hydrometallation may proceed *via* one of two regioisomeric intermediate complexes; **II-L** leading to the linear product **3**, and **II-B** to the branched product **4**. Owing to their π-acidity, aryl phosphines result in an electron-poor rhodium metal centre, which is compensated for by stronger coordination of the ynamide. This effect is expected to exacerbate steric interactions between the substrate and ligand substituents, favouring intermediate **II-L**, and leading to the linear product **3**. In contrast, strongly σ-donating alkyl phosphines increase electron density on the metal, resulting in a more weakly bound substrate. This, paired with an electronically biased ynamide would allow for the formation of increasing amounts of the branched isomer **4**.

**Fig. 1:**
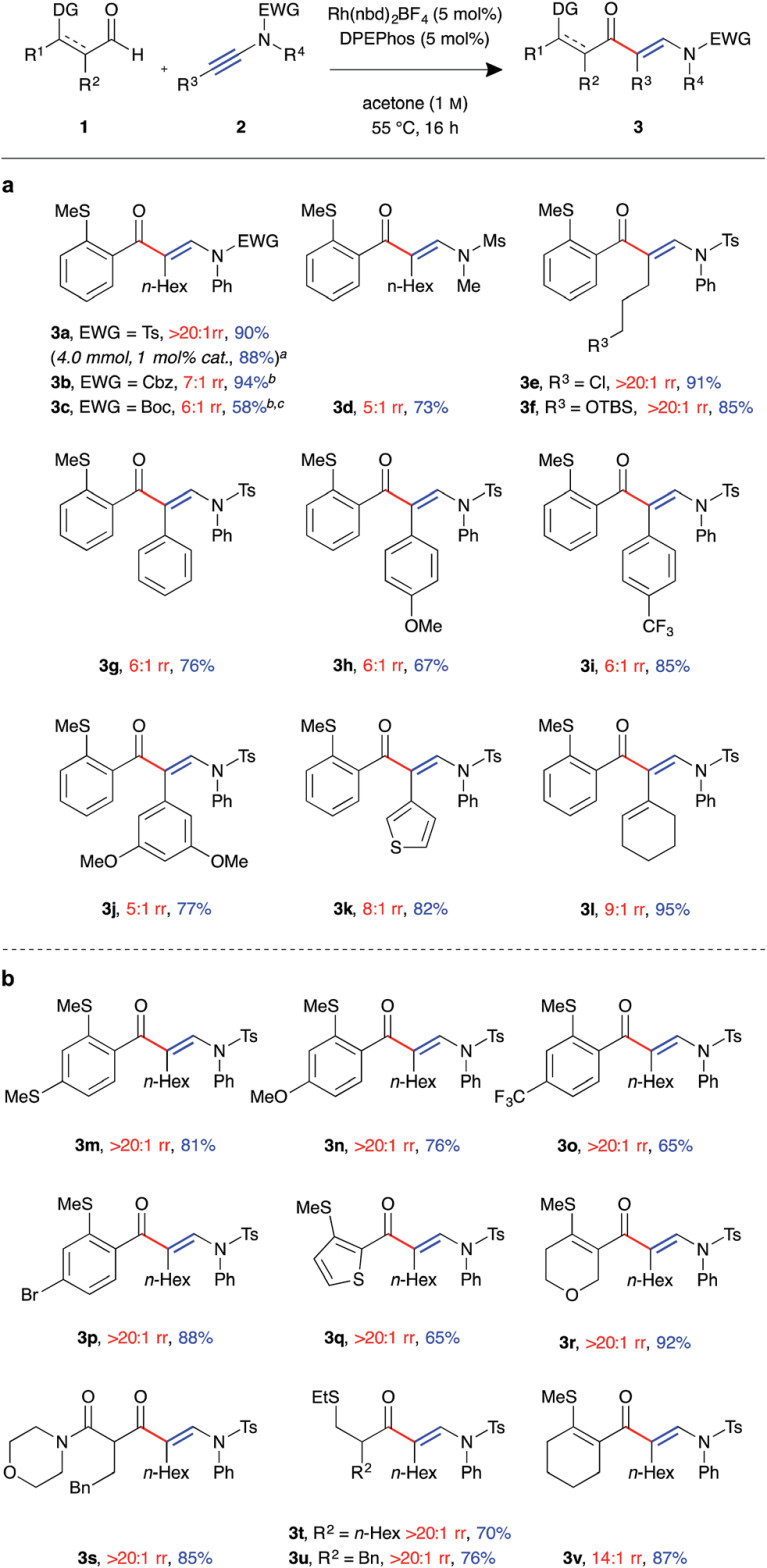
Linear-selective ynamide hydroacylation (a) scope of ynamide component. (b) Scope of aldehyde component. (Rh(nbd)_2_BF_4_ (5 mol%), DPEphos (5 mol%), aldehyde (0.30 mmol, 1.0 equiv.), ynamide (1.1 equiv.), acetone (1.0 M), 55 °C for 16 h. Regioisomeric ratio determined by ^1^H NMR spectroscopic analysis of the crude reaction mixture. ^*a*^Performed with 4 mmol of aldehyde, using 1 mol% catalyst, with the reaction mixture stirred for 40 h. ^*b*^Isolated as an inseparable mixture of isomers. ^*c*^Reaction performed at 2 M concentration).

With a suitable catalyst system in hand, we examined the tolerance of the linear-selective ynamide hydroacylation reaction towards various ynamide substituents (Fig. 1a). Ynamides **2a–l** were synthesised *via* copper-catalysed oxidative coupling of N-protected amines with the corresponding alkynes,^20^ and submitted to the optimised reaction conditions with aldehyde **1a**. Pleasingly, in addition to sulfonamides, the reaction also tolerated carbamate substrates, with carboxybenzyl-protected β-enaminone product **3b** generated in excellent yield, albeit with slightly reduced regioselectivity (7 : 1 rr). Boc-protected ynamide **1c** was less reactive, requiring increased concentration (2 M) to give **3c** in moderate yield. Mesyl-protected methylamine ynamide **1d** performed well, generating the linear product **3d** in high yield. However, here, again, lower levels of regioselectivity were observed (5 : 1 rr), perhaps due to reduced steric bias of the ynamide. Both sp^3^ and sp^2^ hybridised ynamide substituents were well tolerated, with the former providing higher linear:branched selectivities. It was found that under the mild reaction conditions, primary alkyl halides **3e** and silyl ethers **3f** were tolerated, both exhibiting perfect regioselectivity and isolated in >85% yield. Ynamides **2i** and **2j**, bearing electron-poor aromatic groups, gave higher yields compared to that of the neutral and electron-rich aryl substituted ynamides **2g** and **2h**. However, there was little observed change in regioselectivity between the para-substituted aryl ynamides (6 : 1 rr). Thiophenyl and cyclohexenyl substituted ynamides **2k** and **2l** also gave the corresponding β-enaminone products **3k** and **3l** in high yields. In order to assess the practicability of the methodology, reaction of ynamide **2b** was performed on a 4 mmol scale, using only 1 mol% catalyst, which successfully generated enaminone **3b** as a single regioisomer (>20 : 1 rr) in 88% yield (1.79 g) after 40 hours at 55 °C.

We next examined the scope of the reaction with respect to the aldehyde component using various substituted aldehydes **1** (Fig. 1b). Electron-rich aryl aldehydes performed well, with products **3m** and **3n** both obtained in high yields. Electron-poor aryl aldehydes exhibited the desired reactivity, however, β-enaminone **3o** was only isolated in moderate yield. In contrast, bromo-substituted product **3p** was obtained in excellent yield. Thiophenyl aldehyde **1q** was found to be less reactive, with the reaction not reaching completion after 16 h at 55 °C. As a result the product **3q** was isolated in 65% yield. Dihydropyran **3r** was formed in excellent yield. Our laboratory recently reported the use of β-carbonyl-substituted aldehydes in alkene and alkyne hydroacylation reactions,^21^ which here too demonstrated as efficient substrates; β-enaminone **3s** was obtained in an 85% yield as a single regioisomer (>20 : 1 rr). Pleasingly, α-substituted alkyl aldehydes also underwent the desired C–H oxidative addition, to yield hydroacylation products **3t** and **3u** in good yield. β-Substituted alkyl aldehydes were found to be unreactive using the current methodology. Cyclohexenyl aldehyde **1v** was the only example to exhibit lower than perfect levels of regioselectivity when combined with an alkyl substituted ynamide (14 : 1 rr); despite this, the product **3v** was isolated in excellent yield.

**Fig. 2:**
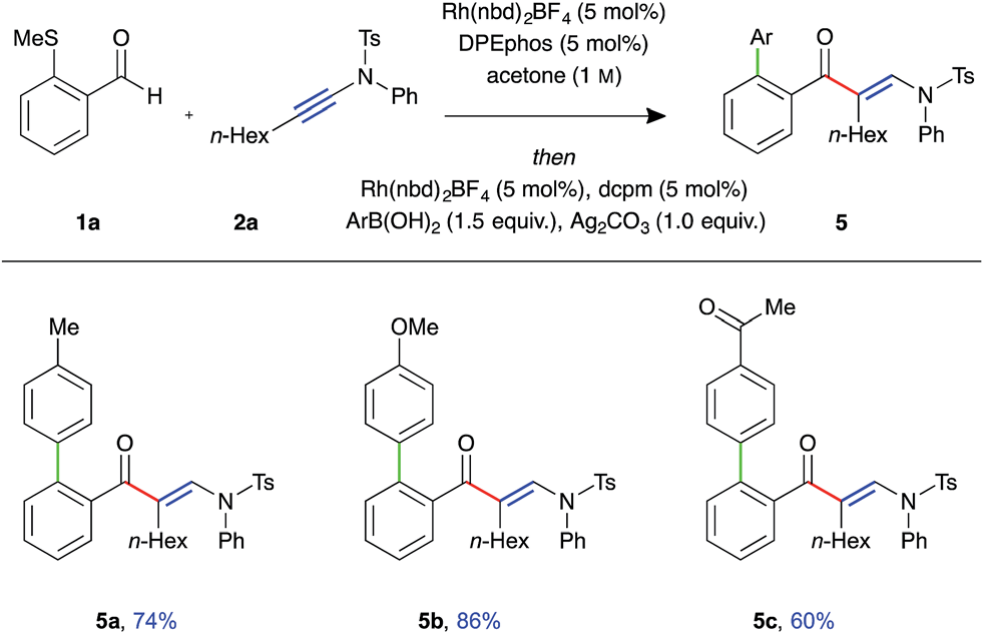
Three-component ynamide hydroacylation/Suzuki-type coupling (Rh(nbd)_2_BF_4_ (5 mol%), DPEphos (5 mol%), aldehyde (0.3 mmol, 1 equiv.), ynamide (1.1 equiv.), acetone (1.0 M), 55 °C for 16 h; then Rh(nbd)_2_BF_4_ (5 mol%), dcpm (5 mol%), silver carbonate (1.0 equiv.), boronic acid (1.5 equiv.), acetone (0.3 M), 55 °C for 16 h).

To demonstrate the utility of the requisite sulfide directing group present in the β-enaminone products, three-component ynamide hydroacylation/Suzuki-type coupling reactions were performed (Fig. 2).^22^ Upon consumption of the aldehyde starting material, the reaction mixture was transferred to a second reaction vessel containing a solution of Rh-dcpm catalyst, boronic acid, and silver carbonate in acetone, and the reaction mixture stirred for a further 16 hours at 55 °C. The coupled β-enaminone products **5a–c** were formed in high yield over two steps.

**Fig. 3:**
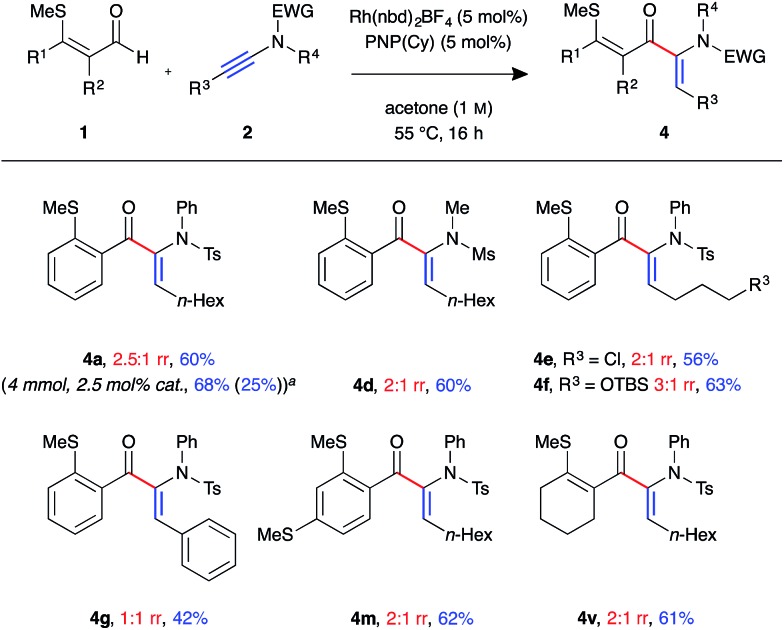
Scope of branched-selective ynamide hydroacylation (Rh(nbd)_2_BF_4_ (5 mol%), PNP(Cy) (5 mol%), aldehyde (0.3 mmol, 1 equiv.), ynamide (1.1 equiv.), acetone (1.0 M), 55 °C for 16 h; regioisomeric ratio determined by ^1^H NMR spectroscopic analysis of the crude reaction mixture; yields of isolated single regioisomers. ^*a*^Performed with 4 mmol of aldehyde, using 2.5 mol% catalyst, with the reaction mixture stirred for 16 h, value in parentheses is the isolated yield of linear isomer **3a**).

In the process of optimising the linear-selective hydroacylation reaction we observed a reversal in regioselectivity with the use of the PNP(Cy) ligand, which led to the formation of the α-enaminone product **4a**. These have been shown as valuable precursors for the synthesis of chiral α-amino acid derivatives *via* asymmetric reduction.^23^ As such we decided to examine the scope of the branched-selective reaction with a range of aldehydes and ynamides (Fig. 3). Overall, a lower level of regioselectivity was observed than in the linear selective reaction. Nevertheless, it was possible to separate, using simple silica column chromatography, and isolate the branched products **4** in moderate to good yields. For example, formation of branched product **4a** was achieved on a 4 mmol scale, with 2.5 mol% catalyst loading, and was isolated as a single regoisomer in 68% yield (1.38 g) after 16 h at 55 °C. The linear isomer **3b** was also isolated from this reaction in 25% yield (0.51 g). As in the linear-selective reaction, aryl substituted ynamide **2e** exhibited the lowest level of regioselectivity (1 : 1 rr), with the branched isomer isolated in 42%.

**Fig. 4:**
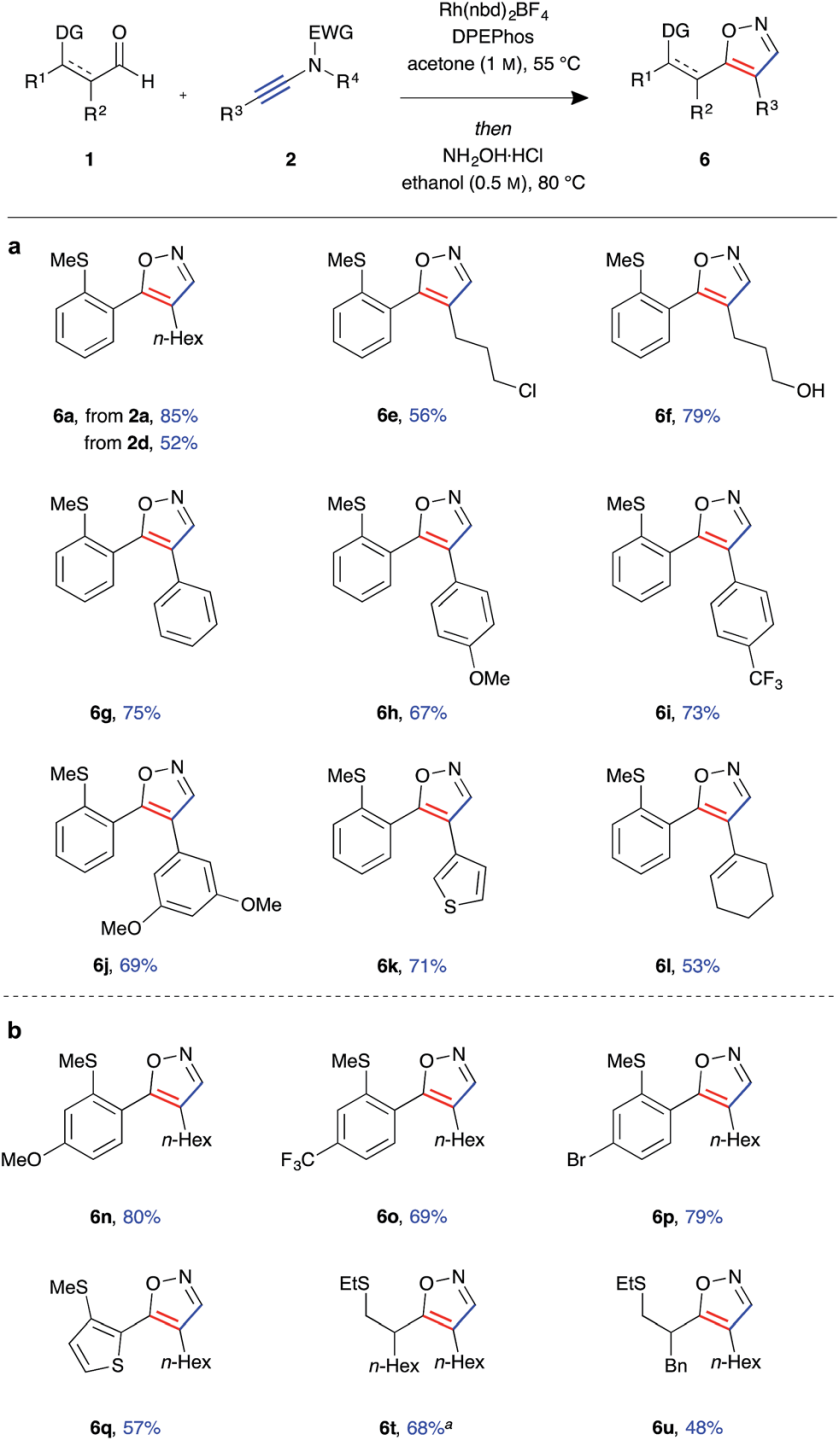
Tandem ynamide hydroacylation/isoxazole formation (a) scope of ynamide component. (b) Scope of aldehyde component (Rh(nbd)_2_BF_4_ (5 mol%), DPEphos (5 mol%), aldehyde (0.3 mmol, 1 equiv.), ynamide (1.1 equiv.), acetone (1.0 M), 55 °C for 16 h, then hydroxylamine hydrochloride (5 equiv.), ethanol (0.5 M), 80 °C for 16 h). ^*a*^Isolated along with the regioisomeric isoxazole (<10%).

Having established a robust protocol for ynamide hydroacylation, we turned our attention to utilising the β-enaminone products in the generation of heterocyclic compounds (Fig. 4). It was found that under acidic conditions, in the presence of an external nucleophile, these species indeed behave as 1,3-dicarbonyl surrogates. Furthermore, isoxazole products **6** could be obtained directly in a one-pot hydroacylation/nucleophilic addition/cyclisation process. Upon consumption of the aldehyde starting material, hydroxylamine hydrochloride and ethanol were added, and the reaction mixture stirred for a further 16 hours at 80 °C. The protic solvent was crucial for reactivity as the reaction was found to proceed through an enol ether adduct of the β-enaminone and alcohol, which could be isolated from the reaction mixture when the reaction was conducted at room temperature. In general, the isoxazole products **6a–u** were isolated in high yields and near quantitative conversion from β-enaminone intermediates **3** in a one-pot procedure. The reactions were highly selective, with a single regioisomer observed in almost all cases. The products were determined to be 4,5-disubstituted isoxazoles by observation of *n*Oe interactions of the *N*-methylated isoxazole derivative of **6g** (see ESI†). Formation of heterocycle **6a** could be achieved using either *N*-tosyl-aniline ynamide **2a** or *N*-mesyl-methylamine ynamide **2d**, the former providing the product in higher yield (85%) due to both higher selectivity in the hydroacylation reaction and better reactivity of the β-enaminone intermediate **3a** compared to **3d**. Primary alkyl halide **6e** was isolated in relatively low yield, likely due to unwanted side reaction arising from nucleophilic substitution by hydroxylamine. Similarly, under the acidic reaction conditions, the silyl-protected primary alcohol was deprotected, and the free alcohol product **6f** isolated in 79% yield.

**Fig. 5:**
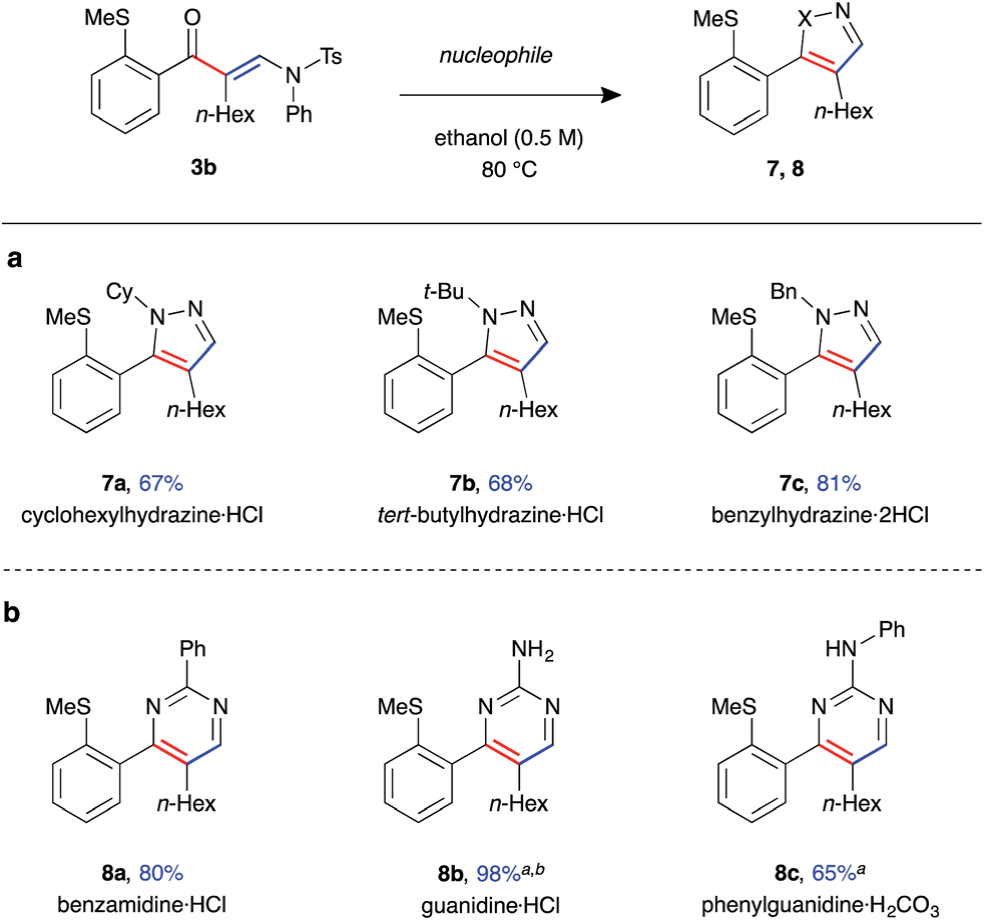
Scope of heterocycle formation from β-enaminone **3b** (a) pyrazoles. (b) Pyrimidines (enaminone (0.2 mmol, 1.0 equiv.), nucleophile (5 equiv.), ethanol (0.5 M), 80 °C for 16 h). ^*a*^Additional K_2_CO_3_ (6 equiv.), reaction performed in DMF (0.5 M) at 100 °C for 16 h. ^*b*^Isolated after work-up without purification.

Finally, enaminone **3b** could be further derivatised with a variety nucleophiles to generate an array of heterocyclic products (Fig. 5). Use of *N*-substituted hydrazines gave 4,5-disubstituted pyrazoles **7a–c** in high yields under the above cyclisation reaction conditions (Fig. 5a). A single regioisomer was observed in all cases, with the product regiochemistry of **7b** determined by *n*Oe experiment (see ESI†). Under the same reaction conditions, the synthesis of 3,4-disubstituted pyrimidine **8a** was achieved with benzamidine, with the product isolated in excellent yield (Fig. 5b). Reaction with guanidines was only possible under basic conditions, requiring addition of K_2_CO_3_ as base and DMF as solvent, yielding amino-pyrimidines **8b** and **8c** in good yield.

## Conclusions

In summary, we have developed the first examples of ynamide hydroacylation, which yield substituted α- and β-enaminones with tunable regioselectivity determined by the choice of catalyst system. Using this methodology, it was possible to synthesise a number of heterocyclic products, from a single set of hydroacylation starting materials and a selected nucleophile, in tandem hydroacylation/nuclophilic addition/cyclisation reactions.

## Conflicts of interest

There are no conflicts to declare.

## Supplementary Material

Supplementary information
