# Supplementary material for: Exploiting rhodium-catalysed ynamide hydroacylation as a platform for divergent heterocycle synthesis
Source: Chem Sci. 2017 Oct 5;8(12):7963–8. doi: 10.1039/c7sc03795c (PMC5853270; doi:10.1039/c7sc03795c)

Electronic Supplementary Material (ESI) for Chemical Science.
This journal is © The Royal Society of Chemistry 2017

Exploiting Rhodium-Catalysed Ynamide Hydroacylation as a Platform for Divergent Heterocycle Synthesis

Robert N. Straker, Manjeet K. Majhail and Michael C. Willis*

*Department of Chemistry, University of Oxford, Chemistry Research Laboratory, Mansfield Road, Oxford, OX1 3TA, United Kingdom.

Supplementary Information

Table of contents

1	**General experimental considerations**		**S2**
2	**General procedures**		**S3**
3	**Characterisation of compounds**		**S5**
	3.1	*Ynamides*	*S5*
	3.2	*Linear hydroacylation products*	*S11*
	3.3	*Branched hydroacylation products*	*S26*
	3.4	*Suzuki-type coupling products*	*S31*
	3.5	*Isoxazole products*	*S34*
	3.6	*Pyrazole products*	*S43*
	3.7	*Pyrimidine products*	*S46*
4	**Assignment of regiochemistry**		**S49**
5	**References**		**S50**
6	**NMR Spectra**		**S51**

1 General Experimental Considerations

Reactions were performed under inert atmosphere of argon with anhydrous solvent unless otherwise stated. All glassware was oven dried at >80 °C, and allowed to cool to room temperature under a positive argon pressure. Reactions were monitored by TLC until deemed complete using aluminum backed silica plates. Plates were visualized under ultraviolet light and/or by staining with vanillin or $KMnO_4$ stains.[1]

Reagents were purchased from Acros Organics Ltd., Alfa Aesar, Fluorochem, Sigma-Aldrich Chemical Co. Ltd. or Strem Chemicals Inc. and were used as supplied. Acetone was distilled from Drierite®. Dichloromethane was distilled from calcium hydride. Petrol refers to the petroleum fraction of aliphatic hydrocarbons with a boiling range between 40 and 60 °C. Ether refers to diethyl ether. Flash chromatography was carried out using matrix 60 silica. Alkynes were distilled prior to use. Aldehydes were prepared according to literature procedures.[2,3]

^{1}H NMR spectra were obtained on Bruker AVIII400 (400 MHz) or Bruker AVII500 (500 MHz) spectrometers using the residual solvent as an internal standard. ^{13}C NMR spectra were obtained on Bruker AVIII400 (100 MHz) or Bruker AVII500 (125 MHz) spectrometer using the residual solvent as an internal standard. Chemical shifts are reported in parts per million (ppm) with the multiplicities of the spectra reported as following: s, singlet; d, doublet; t, triplet; q, quartet; quin., quintet; m, multiplet. Low-resolution ESI mass spectra were recorded on a Waters LCT Premier spectrometer. High-resolution ESI mass spectrometry measurements were recorded on a Bruker Daltronics microTOF (ESI) spectrometer by the internal service at the Department of Organic Chemistry, University of Oxford. Infrared spectra were recorded as thin films on a Bruker Tensor 27 FT-IR spectrometer. Melting points were determined using a Stuart Scientific Melting Point Apparatus SMP1.

2 General Procedures

General Procedure A: *Copper-catalysed ynamide formation*

According to the procedure of Stahl et al.,[4] a flask containing sulfonamide (5.0 equiv.), $CuCl_2$ (0.2 equiv.) and Na_2CO_3 (2.0 equiv.) was purged with oxygen for 15 min. A solution of pyridine (2.0 equiv.) in toluene (5 mL/mmol alkyne) was added to the reaction flask, and the mixture was heated to 70 °C under an atmosphere of oxygen (1 atm). A solution of alkyne (1.0 equiv.) in toluene (5 mL/mmol alkyne) was added to the reaction flask over 4 h using a syringe pump. After addition of the alkyne, the reaction mixture was allowed to stir for 16 h at 70 °C. The crude reaction mixture was cooled to room temperature, filtered through a pad of celite, and concentrated *in vacuo*.

General Procedure B: *Linear-selective ynamide hydroacylation*

A 10 mL microwave vial containing $[Rh(nbd)_2]BF_4$ (5 mol%) and DPEphos (5 mol%) was placed under vacuum (0.05 mbar) and back-filled with argon. This vacuum cycle was repeated three times. The mixture was dissolved in acetone (1 mL/mmol aldehyde), and hydrogen bubbled through the solution until a colour change was observed (orange to dark red, approx. 2 min). The solvent was then removed by purging the vial with argon. The air and moisture sensitive catalyst residue was dissolved in acetone (1 mL/mmol aldehyde), and the solution added to an argon-filled 10 mL microwave vial containing aldehyde (1.0 equiv.) and ynamide (1.1 equiv.). The solution was stirred at 55 °C for 16 h, after which time the crude reaction mixture was concentrated *in vacuo*.

General Procedure C: *Sequential ynamide hydroacylation/Suzuki-type coupling*

Following execution of General Procedure B, upon consumption of aldehyde starting material, the reaction mixture was transferred *via* syringe to a 10 mL microwave vial containing a solution of Rh-dcpm catalyst (generated using the protocol in General Procedure B, 5 mol%), boronic acid (1.5 equiv.), and silver carbonate (1.0 equiv.) in acetone (3 mL/mmol aldehyde). The mixture was stirred at 55 °C for 16 h, after which time the crude reaction mixture was filtered through a pad of silica and concentrated *in vacuo*.

General Procedure D: *One-pot hydroacylation/isoxazole formation*

Following execution of General Procedure B, upon consumption of aldehyde starting material, the reaction mixture was cooled to room temperature. To the solution was added hydroxylamine hydrochloride (5.0 equiv.) and ethanol (2 mL/mmol aldehyde), and the reaction mixture stirred for 16 h at 80 °C. The resulting solution was partitioned between CH_2Cl_2 (10 mL/mmol aldehyde) and water (10 mL/mmol) and the aqueous layer extracted with CH_2Cl_2 (2 x 10 mL/mmol aldehyde). The combined organic extracts were dried over magnesium sulfate, filtered, and concentrated *in vacuo*.

3 Characterisation of compounds

3.1 Ynamides

4-Methyl-*N*-(oct-1-yn-1-yl)-*N*-phenylbenzenesulfonamide, 2a

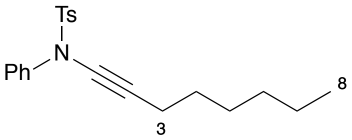

Prepared according to General Procedure A, using 1-octyne (0.48 mL, 3.23 mmol, 1.0 equiv.). The crude material was purified by column chromatography (petrol/EtOAc (98:2)) to give **2a** as a yellow oil (0.96 g, 2.69 mmol, 83%); **^{1}H NMR** (400 MHz, $CDCl_3$) δ_H 7.55 (2H, d, *J* = 8.5 Hz, Ts*H*), 7.34-7.24 (7H, m, Ph*H* and Ts*H*), 2.44 (3H, s, TsC*H*$_3$), 2.29 (2H, t, *J* = 7.0 Hz, H3), 1.50 (2H, quin., *J* = 7.0 Hz, H4), 1.40-1.23 (6H, m, H5-7), 0.89 (3H, t, *J* = 7.0 Hz, H8); **^{13}C NMR** (101 MHz, $CDCl_3$) δ_C 144.7, 139.5, 133.1, 129.4, 129.0, 128.4, 128.0, 126.2, 74.0, 70.5, 31.5, 28.9, 28.6, 22.7, 21.8, 18.6, 14.2; **HRMS** (ESI^+) calc. for $C_{21}H_{25}O_2NNaS$ $[M+Na]^+$ 378.1498, found 378.1505.
The spectroscopic data was found to be in agreement with that reported by Ye *et al.*[6]

Benzyl oct-1-yn-1-yl(phenyl)carbamate, 2b

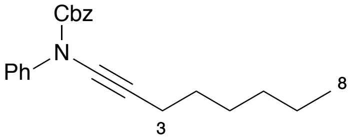

Prepared according to the procedure of Hsung *et al.*,[9] to a vial containing benzyl phenylcarbamate (1.35 g, 5.96 mmol, 1.0 equiv.), 1-bromooct-1-yne (2.25 g, 11.92 mmol, 2.0 equiv.), K_3PO_4 (2.53 g, 11.92 mmol, 2.0 equiv.), $CuSO_4{\cdot}5H_2O$ (595 mg, 2.38 mmol, 0.4 equiv.) and 1,10-phenanthroline (860 mg, 4.77 mmol, 0.8 equiv.) was added toluene (17.9 mL) and the reaction mixture heated to 85 °C and stirred for 16 h. The reaction mixture was filtered through a plug of Celite® and the crude material purified by column chromatography (petrol/EtOAc (99:1)) to give **2b** as an orange oil (1.16 g, 3.47 mmol, 58%); **R_f** 0.28 (petrol/EtOAc (98:2)); **IR** (thin film, ν_{max}/cm^{-1}) 3035, 2929, 2857, 2267, 1732, 1596, 1493, 1381, 1267, 1198; **^{1}H NMR** (400 MHz, $CDCl_3$) δ_H 7.41 (2H, d, *J* = 8.5 Hz, Ph*H*), 7.33-7.22 (7H, m, Ph*H*), 7.16 (1H, t, *J* = 7.5 Hz, Ph*H*), 5.20 (2H, s, PhC*H*$_2$O), 2.24 (2H, t, *J* =

7.0 Hz, H3), 1.48-1.41 (2H, m, H4), 1.34-1.27 (2H, m, H5), 1.23-1.14 (4H, m, H6 and 7), 0.80 (3H, t, J = 7.0 Hz, H8); **^{13}C NMR** (101 MHz, $CDCl_3$) δ_C 154.9, 140.1, 135.7, 128.9, 128.6, 128.4, 128.0, 126.7, 124.5, 73.8, 70.1, 68.7, 31.5, 29.0, 28.7, 22.7, 18.6, 14.2; **HRMS** (ESI^+) calc. for $C_{22}H_{25}O_2NNa$ $[M+Na]^+$ 358.1778, found 358.1778.

***tert*-Butyl oct-1-yn-1-yl(phenyl)carbamate, 2c**

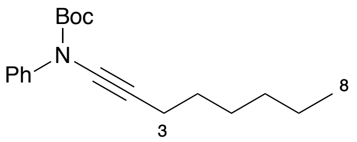

Prepared according to the procedure of Tam et al.,[10] to a stirred solution of *tert*-butyl phenylcarbamate (1.00 g, 5.17 mmol, 1.0 equiv.), 1-bromooct-1-yne (1.96 g, 10.3 mmol, 2.0 equiv.), CuI (197 mg, 1.03 mmol, 0.2 equiv.) and 1,10-phenanthroline (233 mg, 1.29 mmol, 0.25 equiv.) in toluene (8.62 mL) at 90 °C was added a solution of KHMDS (0.5 M in toluene, 20.7 mL, 10.3 mmol, 2.0 equiv.) dropwise over 4 h. The reaction mixture was stirred at 90 °C for a further 16 h under argon. The reaction mixture was partitioned between diethyl ether (50 mL) and water (50 mL), and the aqueous layer extracted with diethyl ether (2 x 50 mL). The combined organic extracts were dried over magnesium sulfate, filtered, and concentrated *in vacuo*. The crude material was purified by column chromatography (petrol/EtOAc (95:5)) to give **2c** as an orange oil (1.28 g, 4.07 mmol, 79%); **R_f** 0.30 (petrol/EtOAc (98:2)); **^{1}H NMR** (400 MHz, $CDCl_3$) δ_H 7.46 (2H, d, J = 7.5 Hz, ArH), 7.35 (2H, t, J = 7.5 Hz, ArH), 7.20 (1H, t, J = 7.5 Hz, ArH), 2.32 (2H, t, J = 7.0 Hz, H3), 1.57-1.50 (11H, m, H4 and $C(CH_3)_3$), 1.45-1.36 (2H, m, H5), 1.34-1.24 (4H, m, H6 and 7), 0.89 (3H, t, J = 7.0 Hz, H8); **^{13}C NMR** (101 MHz, $CDCl_3$) δ_C 153.7, 140.4, 128.7, 126.3, 124.7, 83.0, 74.5, 69.5, 31.5, 29.1, 28.7, 28.2, 22.7, 18.7, 14.2.

The spectroscopic data was found to be in agreement with that reported by Oshima *et al.*[11]

***N*-Methyl-*N*-(oct-1-yn-1-yl)methanesulfonamide, 2d**

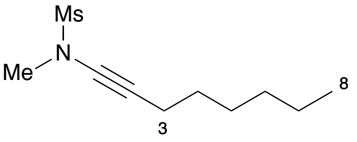

Prepared according to General Procedure A, using *N*-methylmethanesulfonamide (2.51 g, 23.0 mmol, 5.0 equiv.) and 1-octyne (0.68 mL, 4.60 mmol, 1.0 equiv.). The crude material was purified

by column chromatography (petrol/EtOAc (95:5)) to give **2d** as a colourless oil (766 mg, 3.52 mmol, 77%); **R*f*** 0.27 (petrol/EtOAc (90:10)); **^{1}H NMR** (400 MHz, $CDCl_3$) δ_H 3.14 (3H, s, NC*H*$_3$), 3.02 (3H, s, MsC*H*$_3$), 2.26 (2H, t, *J* = 7.5 Hz, H3), 1.53-1.46 (2H, m, H4), 1.40-1.25 (6H, m, H5-7), 0.88 (3H, t, *J* = 7.0 Hz, H8); **^{13}C NMR** (101 MHz, $CDCl_3$) δ_C 74.3, 69.4, 39.3, 36.0, 31.4, 29.0, 28.6, 22.7, 18.5, 14.2.

The spectroscopic data was found to be in agreement with that reported by Mezzetti *et al.*[8]

N-(5-Chloropent-1-yn-1-yl)-4-methyl-*N*-phenylbenzenesulfonamide, 2e

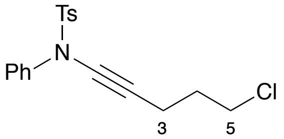

Prepared according to General Procedure A, using 5-chloro-1-pentyne (0.34 mL, 3.23 mmol, 1.0 equiv.). The crude material was purified by column chromatography (petrol/EtOAc (98:2)) to give **2e** as a colourless oil (0.97 g, 2.78 mmol, 86%); **R*f*** 0.29 (petrol/EtOAc (90:10)); **IR** (thin film, ν_{max}/cm^{-1}) 2952, 2254, 1595, 1488, 1369, 1269, 1174, 1090; **^{1}H NMR** (400 MHz, $CDCl_3$) δ_H 7.55 (2H, d, *J* = 8.5 Hz, Ts*H*), 7.35-7.23 (7H, m, Ph*H* and Ts*H*), 3.63 (2H, t, *J* = 6.5 Hz, H5), 2.51 (2H, d, *J* = 6.5 Hz, H3), 2.44 (3H, s, TsC*H*$_3$), 1.96 (2H, quint., *J* = 6.5 Hz, H4); **^{13}C NMR** (101 MHz, $CDCl_3$) δ_C 145.0, 139.2, 132.9, 129.6, 129.1, 128.3, 128.2, 126.2, 75.0, 68.5, 43.8, 31.5, 21.8, 16.0; **HRMS** (ESI$^+$) calc. for $C_{18}H_{18}O_2NClNaS$ $[M+Na]^+$ 370.0639, found 370.0644.

N-(5-((*tert*-Butyldimethylsilyl)oxy)pent-1-yn-1-yl)-4-methyl-*N*-phenylbenzenesulfonamide, 2f

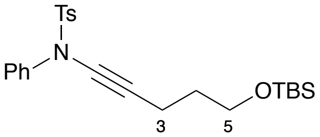

Prepared according to General Procedure A, using *tert*-butyldimethyl(pent-4-yn-1-yloxy)silane (0.641 g, 3.23 mmol, 1.0 equiv.). The crude material was purified by column chromatography (petrol/EtOAc (98:2)) to give **2f** as an amorphous solid (1.052 g, 2.37 mmol, 73%); **R*f*** 0.40 (petrol/EtOAc (90:10)); **IR** (thin film, ν_{max}/cm^{-1}) 2953, 2929, 2856, 2255, 1596, 1490, 1374, 1256, 1176, 1102; **^{1}H NMR** (400 MHz, $CDCl_3$) δ_H 7.55 (2H, d, *J* = 8.5 Hz, Ts*H*), 7.34-7.24 (7H, m, Ts*H* and Ph*H*), 3.66 (2H, t, *J* = 6.0 Hz, H5), 2.44 (3H, s, TsC*H*$_3$), 2.39 (2H, t, *J* = 7.0 Hz, H3), 1.72 (2H, tt, *J* = 7.0 and 6.0 Hz, H4), 0.89 (9H, s, SiC(C*H*$_3$)$_3$), 0.03 (6H, s, Si(C*H*$_3$)$_2$); **^{13}C NMR** (101 MHz,

$CDCl_3$) δ_C 144.7, 139.4, 133.1, 129.5, 129.0, 128.3, 128.0, 126.2, 74.0, 70.0, 61.7, 32.1, 26.1, 21.8, 18.4, 15.1, –5.2; **HRMS** (ESI^+) calc. for $C_{24}H_{33}O_3NNaSSi$ $[M+Na]^+$ 466.1843, found 466.1841.

4-Methyl-*N*-phenyl-*N*-(phenylethynyl)benzenesulfonamide, 2g

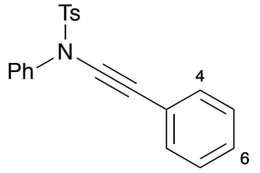

Prepared according to General Procedure A, using phenylacetylene (0.63 mL, 5.74 mmol, 1.0 equiv.). The crude material was purified by column chromatography (petrol/EtOAc (96:4)) to give **2g** as a colourless, amorphous solid (1.22 g, 3.51 mmol, 61%); **^{1}H NMR** (400 MHz, $CDCl_3$) δ_H 7.64 (2H, d, *J* = 8.5 Hz, Ts*H*), 7.41-7.29 (12H, m, H4-6, Ph*H* and Ts*H*), 2.45 (3H, s, TsCH_3); **^{13}C NMR** (101 MHz, $CDCl_3$) δ_C 145.1, 139.1, 133.0, 131.6, 129.6, 129.2, 128.4_1 (2C), 128.3_8, 128.1, 126.4, 122.7, 83.1, 70.6, 21.9; **HRMS** (ESI^+) calc. for $C_{21}H_{17}O_2NNaS$ $[M+Na]^+$ 370.0872, found 370.0875.
The spectroscopic data was found to be in agreement with that reported by Saá *et al.*[7]

***N*-((4-Methoxyphenyl)ethynyl)-4-methyl-*N*-phenylbenzenesulfonamide, 2h**

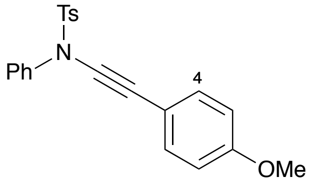

Prepared according to General Procedure A, using 4-ethynylanisole (0.42 mL, 3.23 mmol, 1.0 equiv.). The crude material was purified by column chromatography (petrol/EtOAc (90:10)) to give **2h** as a colourless amorphous solid (0.69 g, 1.83 mmol, 57%); **^{1}H NMR** (400 MHz, $CDCl_3$) δ_H 7.62 (2H, d, *J* = 8.5 Hz, Ts*H*), 7.36-7.28 (9H, m, H4, Ph*H*, and Ts*H*), 6.84 (2H, d, *J* = 9.0 Hz, H5), 3.80 (3H, s, OCH_3), 2.45 (3H, s, TsCH_3); **^{13}C NMR** (101 MHz, $CDCl_3$) δ_C 159.7, 145.0, 139.3, 133.5, 133.1, 129.6, 129.2, 128.4, 128.2, 126.3, 114.6, 114.1, 81.7, 70.4, 55.4, 21.8; **HRMS** (ESI^+) calc. for $C_{22}H_{19}O_3NNaS$ $[M+Na]^+$ 400.0978, found 400.0975.
The spectroscopic data was found to be in agreement with that reported by Saá *et al.*[7]

4-Methyl-*N*-phenyl-*N*-((4-(trifluoromethyl)phenyl)ethynyl)benzenesulfonamide, 2i

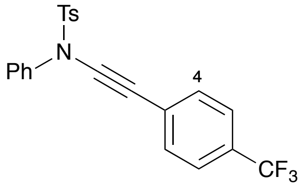

Prepared according to General Procedure A, using 4-ethynyl-α,α,α-trifluorotoluene (0.53 mL, 3.23 mmol, 1.0 equiv.). The crude material was purified by column chromatography (petrol/EtOAc (96:4)) to give **2i** as a colourless amorphous solid (0.77 g, 1.85 mmol, 57%); **R*f*** 0.33 (petrol/EtOAc (90:10)); **IR** (thin film, ν_{max}/cm^{-1}) 2968, 2918, 2251, 1683, 1602, 1511, 1353, 1122, 1065; **^{1}H NMR** (500 MHz, $CDCl_3$) δ_H 7.62 (2H, d, *J* = 8.5 Hz, Ts*H*), 7.55 (2H, d, *J* = 8.5 Hz, H5), 7.47 (2H, d, *J* = 8.5 Hz, H4), 7.39-7.35 (3H, m, Ph*H*), 7.32-7.29 (4H, m, Ph*H* and Ts*H*), 2.45 (3H, s, TsC*H*$_3$); **^{13}C NMR** (126 MHz, $CDCl_3$) δ_C 145.4, 138.7, 133.1, 131.3, 129.8, 129.5 (q, *J* = 32.0 Hz), 129.4, 128.7, 128.4, 126.8, 126.5, 125.3 (q, *J* = 4.0 Hz), 124.1 (q, *J* = 272.0 Hz), 85.7, 69.8, 21.6; **^{19}F NMR** (470 MHz, $CDCl_3$) δ_F –62.7; **HRMS** (ESI^+) calc. for $C_{22}H_{16}O_2NF_3NaS$ $[M+Na]^+$ 438.0746, found 438.0752.

***N*-((3,5-Dimethoxyphenyl)ethynyl)-4-methyl-*N*-phenylbenzenesulfonamide, 2j**

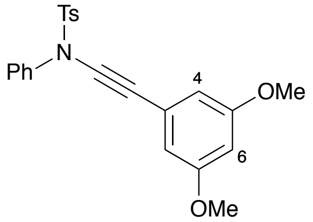

Prepared according to General Procedure A, using 1-ethynyl-3,5-dimethoxybenzene (350 mg, 2.16 mmol, 1.0 equiv.). The crude material was purified by column chromatography (petrol/EtOAc (90:10)), and then dissolved in CH_2Cl_2 (20 mL) and washed with sodium hydroxide (*aq.* 1.0 M, 3 x 20 mL) to give **2j** as a pale pink amorphous solid (522 mg, 1.28 mmol, 59%); **R*f*** 0.13 (petrol/EtOAc (90:10)); **IR** (thin film, ν_{max}/cm^{-1}) 3003, 2938, 2842, 2238, 1587, 1490, 1454, 1420, 1370, 1172, 1154, 1044; **^{1}H NMR** (400 MHz, $CDCl_3$) δ_H 7.62 (2H, d, *J* = 8.5 Hz, Ts*H*) 7.38-7.26 (7H, m, Ts*H* and Ar*H*), 6.54 (2H, d, *J* = 2.5 Hz, H4), 6.42 (1H, t, *J* = 2.5 Hz, H6), 3.77 (3H, s, OC*H*$_3$), 2.44 (3H, s, TsC*H*$_3$); **^{13}C NMR** (101 MHz, $CDCl_3$) δ_C 160.6, 145.2, 138.9, 133.0, 129.7, 129.2, 128.4, 128.4, 126.4, 124.1, 109.3, 101.3, 82.7, 70.7, 55.5, 21.9; **HRMS** (ESI^+) calc. for $C_{23}H_{21}O_4NNaS$ $[M+Na]^+$ 430.1084, found 430.1082.

4-Methyl-*N*-phenyl-*N*-(thiophen-3-ylethynyl)benzenesulfonamide, 2k

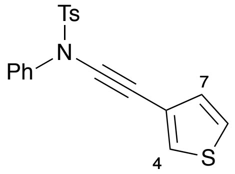

Prepared according to General Procedure A, using 3-ethynylthiophene (0.32 mL, 3.23 mmol, 1.0 equiv.). The crude material was purified by column chromatography (petrol/EtOAc (95:5)) to give **2k** as a yellow oil (0.632 g, 1.79 mmol, 55%); **R*f*** 0.16 (petrol/EtOAc (90:10)); **IR** (thin film, ν_{max}/cm^{-1}) 3109, 2241, 1594, 1490, 1371, 1212, 1089, 1027; **^{1}H NMR** (400 MHz, $CDCl_3$) δ_H 7.63 (2H, d, *J* = 8.5 Hz, Ts*H*), 7.41 (1H, dd, *J* = 3.0 and 1.0 Hz, H4), 7.37-7.29 (7H, m, Ph*H* and Ts*H*), 7.26 (1H, dd, *J* = 5.0 and 3.0 Hz, H6), 7.08 (1H, dd, *J* = 5.0 and 1.0 Hz, H7), 2.45 (3H, s, TsCH_3); **^{13}C NMR** (101 MHz, $CDCl_3$) δ_C 145.1, 139.0, 133.1, 130.3, 129.6, 129.2, 129.0, 128.4 (2C), 126.4, 125.4, 121.4, 82.4, 65.7, 21.8; **HRMS** (ESI$^+$) calc. for $C_{19}H_{15}NO_2S_2Na$ [M+Na]$^+$ 376.0436, found 376.0439.

***N*-(Cyclohex-1-en-1-ylethynyl)-4-methyl-*N*-phenylbenzenesulfonamide, 2l**

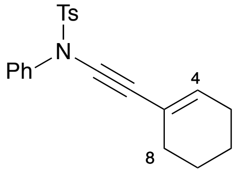

Prepared according to General Procedure A, using 1-ethynylcyclohexene (0.38 mL, 3.23 mmol, 1.0 equiv.). The crude material was purified by column chromatography (petrol/EtOAc (95:5)) to give **2l** as an orange amorphous solid (0.45 g, 1.28 mmol, 40%); **R*f*** 0.18 (petrol/EtOAc (90:10)); **IR** (thin film, ν_{max}/cm^{-1}) 2934, 1687, 1596, 1538, 1489, 1360, 1307, 1248, 1170, 1149; **^{1}H NMR** (400 MHz, $CDCl_3$) δ_H 7.66 (2H, d, *J* = 8.5 Hz, Ts*H*), 7.42-7.33 (7H, m, Ph*H* and Ts*H*), 6.15-6.12 (1H, m, H4), 2.52 (3H, s, TsCH_3), 2.22-2.15 (4H, m, H5 and 8), 1.75-1.64 (4H, m, H6 and 7); **^{13}C NMR** (101 MHz, $CDCl_3$) δ_C 144.9, 139.4, 134.7, 133.0, 129.5, 129.1, 128.4, 128.1, 126.3, 120.0, 80.6, 72.3, 29.5, 25.8, 22.5, 21.8, 21.6; **HRMS** (ESI$^+$) calc. for $C_{21}H_{21}O_2NNaS$ [M+Na]$^+$ 374.1185, found 374.1185.

3.2 Linear hydroacylation products

(*E*)-4-Methyl-*N*-(2-(2-(methylthio)benzoyl)oct-1-en-1-yl)-*N*-phenylbenzenesulfonamide, 3a

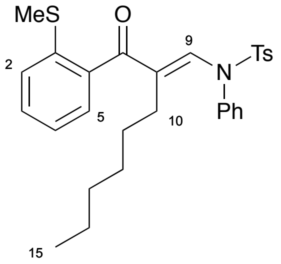

Method A: Prepared according to General Procedure B, using 2-(methylthio)benzaldehyde (40 μL, 0.300 mmol, 1.0 equiv.) and ynamide **2a** (117 mg, 0.330 mmol, 1.1 equiv.). The crude material was purified by column chromatography (petrol/EtOAc (98:2)) to give **3a** as a yellow oil (137 mg, 0.270 mmol, 90%).

Method B (4 mmol scale reaction): Prepared according to General Procedure B, using DPEPhos (22 mg, 0.040 mmol, 1 mol%), 2-(methylthio)benzaldehyde (609 mg, 4.00 mmol, 1.0 equiv.) and ynamide **2a** (1.56 g, 4.40 mmol, 1.1 equiv.). The crude material was purified by column chromatography (petrol/EtOAc (98:2)) to give **3a** as a yellow oil (1.79 g, 3.52 mmol, 88%).

$\mathbf{R}_f$ 0.33 (petrol/EtOAc (80:20)); **IR** (thin film, ν_{max}/cm^{-1}) 2926, 2856, 1694, 1647, 1493, 1433, 1290, 1171, 1128, 1087; ^{1}**H NMR** (400 MHz, $CDCl_3$) δ_H 7.48-7.44 (2H, m, Ar*H* and H9), 7.42-7.32 (4H, m, Ar*H*), 7.30-7.18 (6H, m, Ar*H*), 7.08 (2H, d, *J* = 8.0 Hz, Ar*H*), 2.43 (3H, s, TsCH_3), 2.41 (3H, s, SCH_3), 1.67-1.63 (2H, m, H10), 1.18-1.11 (2H, m, H14), 1.07-0.97 (4H, m, H11 and 13), 0.87-0.79 (5H, m, H12 and 15); ^{13}**C NMR** (101 MHz, $CDCl_3$) δ_C 197.8, 144.9, 144.0, 140.2, 137.7, 137.0, 134.0, 130.1, 129.8, 129.7, 129.3 (2C), 128.3, 128.0, 127.6, 126.5, 125.3, 31.6, 29.4, 27.9, 24.2, 22.6, 21.8, 17.2, 14.2; **HRMS** (ESI$^+$) calc. for $C_{29}H_{34}O_3NS_2$ $[M+H]^+$ 508.1975, found 508.1967.

(*E*)-2,4-Dimethyl-1-(2-(methylthio)phenyl)pent-2-en-1-one, 3aa

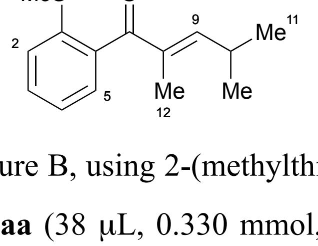

Prepared according to General Procedure B, using 2-(methylthio)benzaldehyde (40 μL, 0.300 mmol, 1.0 equiv.) and 4-methylpent-2-yne **2aa** (38 μL, 0.330 mmol, 1.1 equiv.). The crude material was purified by column chromatography (petrol/Et_2O (98:2)) to give **3aa** as a colourless oil (63 mg,

0.269 mmol, 90%); **R$_f$** 0.32 (petrol/Et_2O (90:10)); **IR** (thin film, ν_{max}/cm^{-1}) 2960, 2924, 2869, 1650, 1637, 1463, 1433, 1311, 1278, 1252, 1184, 1113; **^{1}H NMR** (400 MHz, $CDCl_3$) δ_H 7.38-7.32 (2H, m, Ar*H*), 7.21-7.15 (2H, m, Ar*H*), 5.99 (1H, d, *J* = 10.0 Hz, H9), 2.78-2.69 (1H, m, H10), 2.40 (3H, s, SC*H*$_3$), 1.95 (3H, s, H12), 0.97 (6H, d, *J* = 6.5 Hz, H11); **^{13}C NMR** (101 MHz, $CDCl_3$) δ_C 199.6, 155.2, 140.0, 137.2, 135.4, 130.0, 128.5, 127.7, 124.8, 28.6, 21.8, 17.3, 11.3; **HRMS** (ESI$^+$) calc. for $C_{14}H_{19}OS$ $[M+H]^+$ 235.1151, found 235.1151.

Benzyl (*E*)-(2-(2-(methylthio)benzoyl)oct-1-en-1-yl)(phenyl)carbamate, 3b

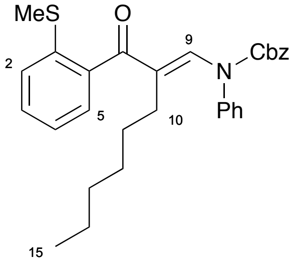

Prepared according to General Procedure B, using 2-(methylthio)benzaldehyde (40 µL, 0.300 mmol, 1.0 equiv.) and ynamide **2b** (110 mg, 0.330 mmol, 1.1 equiv.). The crude material was purified by column chromatography (petrol/EtOAc (95:5)) to give **3b** as an inseparable mixture of regioisomers (7:1 (L:B)) as a colourless oil (137 mg, 0.281 mmol, 94%); **R$_f$** 0.25 (petrol/EtOAc (90:10)); **IR** (thin film, ν_{max}/cm^{-1}) 3063, 2955, 2925, 2856, 1726, 1648, 1616, 1593, 1382, 1228, 1155; **^{1}H NMR** (400 MHz, $CDCl_3$) δ_H 7.79 (1H, s, H9), 7.63-7.54 (4H, m, Ar*H*), 7.51-7.42 (7H, m, Ar*H*), 7.36 (1H, t, *J* = 7.0 Hz, Ar*H*), 7.30-7.28 (2H, m, Ar*H*), 5.30 (2H, s, CbzC*H*$_2$), 2.61 (3H, s, SC*H*$_3$), 1.92-1.88 (2H, m, H10), 1.41-1.32 (2H, m, H14), 1.30-1.19 (4H, m, H11 and 13), 1.10-1.04 (2H, m, H12), 1.01 (3H, t, *J* = 7.5 Hz, H15); **^{13}C NMR** (101 MHz, $CDCl_3$) δ_C 198.4, 153.9, 143.4, 140.3, 139.4, 136.9, 135.3, 130.0, 129.3, 128.6, 128.3_4, 128.2_8, 128.1_5, 128.0, 127.9, 127.7, 127.5, 125.1, 68.7, 31.6, 29.5, 28.3, 24.3, 22.7, 17.1, 14.2; **HRMS** (ESI$^+$) calc. for $C_{30}H_{34}O_3NS$ $[M+H]^+$ 488.2254, found 488.2252.

***tert*-Butyl (*E*)-(2-(2-(methylthio)benzoyl)oct-1-en-1-yl)(phenyl)carbamate, 3c**

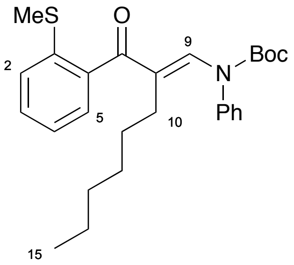

Prepared according to General Procedure B, using 2-(methylthio)benzaldehyde (80 μL, 0.600 mmol, 1.0 equiv.) and ynamide **2c** (208 mg, 0.660 mmol, 1.1 equiv.) in acetone (0.30 mL). The crude material was purified by column chromatography (petrol/EtOAc (96:4)) to give **3c** as an inseparable mixture of regoisomers (6:1 (L:B)) as an orange oil (159 mg, 0.350 mmol, 58%); $\mathbf{R}_f$ 0.33 (petrol/EtOAc (90:10)); **IR** (thin film, ν_{max}/cm^{-1}) 2926, 2856, 1723, 1649, 1615, 1593, 1493, 1334, 1269, 1237, 1143; ^{1}**H NMR** (400 MHz, $CDCl_3$) δ_H 7.59 (1H, s, H9), 7.44-7.33 (5H, m, Ar*H*), 7.30-7.21 (4H, m, Ar*H*), 2.47 (3H, s, SC*H*$_3$), 1.73-1.69 (2H, m, H10), 1.34 (9H, s, OC(C*H*$_3$)$_3$), 1.24-1.02 (6H, m, H11, 13 and 14), 0.92-0.81 (5H, m, H12 and 15); ^{13}**C NMR** (101 MHz, $CDCl_3$) δ_C 198.5, 152.6, 144.2, 140.7, 139.7, 136.8, 129.8, 129.1, 128.1$_4$, 128.0$_8$, 127.9, 127.7, 126.5, 125.1, 83.1, 31.7, 29.5, 28.3, 28.0, 24.2, 22.7, 17.2, 14.2; **HRMS** (ESI$^+$) calc. for $C_{27}H_{34}O_3NS$ [M–H]$^-$ 452.2265, found 452.2271.

(*E*)-*N*-Methyl-*N*-(2-(2-(methylthio)benzoyl)oct-1-en-1-yl)methanesulfonamide, 3d

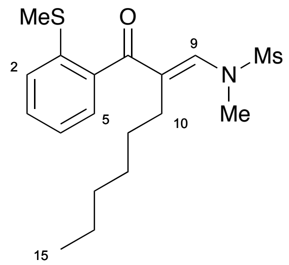

Prepared according to General Procedure B, using 2-(methylthio)benzaldehyde (40 μL, 0.300 mmol, 1.0 equiv.) and ynamide **2d** (72 mg, 0.330 mmol, 1.1 equiv.). The crude material was purified by column chromatography (petrol/EtOAc (9:1)) to give **3d** as a colourless oil (81 mg, 0.220 mmol, 73%); $\mathbf{R}_f$ 0.20 (petrol/EtOAc (80:20)); **IR** (thin film, ν_{max}/cm^{-1}) 2955, 2925, 2856, 1607, 1462, 1433, 1350, 1289, 1269, 1155; ^{1}**H NMR** (400 MHz, $CDCl_3$) δ_H 7.36 (1H, ddd, *J* = 8.0, 7.0 and 2.0 Hz, Ar*H*), 7.29 (1H, d, *J* = 8.0 Hz, Ar*H*), 7.21-7.15 (2H, m, Ar*H*), 7.12 (1H, s, H9), 3.37 (3H, s, MsC*H*$_3$), 2.87 (3H, s, NC*H*$_3$), 2.60-2.56 (2H, m, H10), 2.41 (3H, s, SC*H*$_3$), 1.53-1.46 (2H, m, H11), 1.41-1.24 (6H, m, H12-14), 0.87 (3H, t, *J* = 7.0 Hz, H15); ^{13}**C NMR** (101 MHz, $CDCl_3$) δ_C 197.7, 142.8,

139.5, 136.8, 130.3, 128.3, 127.2, 126.5, 125.1, 38.6, 35.2, 31.7, 30.1, 29.6, 24.9, 22.7, 16.8, 14.2; **HRMS** (ESI^+) calc. for $C_{18}H_{28}O_3NS_2$ $[M+H]^+$ 370.1505, found 370.1505.

(*E*)-*N*-(5-Chloro-2-(2-(methylthio)benzoyl)pent-1-en-1-yl)-4-methyl-*N*-phenylbenzenesulfonamide, 3e

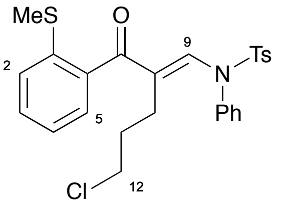

Prepared according to General Procedure B, using 2-(methylthio)benzaldehyde (40 μL, 0.300 mmol, 1.0 equiv.) and ynamide **2e** (115 mg, 0.330 mmol, 1.1 equiv.). The crude material was purified by column chromatography (petrol/EtOAc (90:10)) to give **3e** as a colourless oil (136 mg, 0.272 mmol, 91%); **R*f*** 0.18 (petrol/EtOAc (80:20)); **IR** (thin film, ν_{max}/cm^{-1}) 3062, 2955, 2923, 1647, 1610, 1590, 1492, 1433, 1365, 1306, 1169, 1131, 1087; **^{1}H NMR** (500 MHz, $CDCl_3$) δ_H 7.55 (1H, s, H9), 7.47 (1H, ddd, *J* = 8.0, 7.5 and 1.5 Hz, Ar*H*), 7.43-7.35 (4H, m, Ar*H*), 7.29 (1H, td, *J* = 7.5 and 1.0 Hz, Ar*H*), 7.23 (1H, dd, *J* = 7.5 and 1.0 Hz, Ar*H*), 7.20 (4H, app. s, Ar*H*), 7.09 (2H, d, *J* = 7.5 Hz, Ar*H*), 3.11 (2H, t, *J* = 7.0 Hz, H12), 2.43 (3H, s, TsC*H*3), 2.41 (3H, s, SC*H*3), 1.82-1.79 (2H, m, H10), 1.56-1.50 (2H, m, H11); **^{13}C NMR** (126 MHz, $CDCl_3$) δ_C 197.6, 145.1, 144.9, 140.0, 137.5, 136.8, 133.8, 130.3, 129.8, 129.7_3, 129.6_8, 129.5, 128.1, 128.0, 127.7, 125.5, 124.2, 44.6, 30.8, 21.9, 21.8, 17.2; **HRMS** (ESI^+) calc. for $C_{26}H_{27}O_3NClS_3$ $[M+H]^+$ 500.1115, found 500.1107.

(*E*)-*N*-(5-((*tert*-Butyldimethylsilyl)oxy)-2-(2-(methylthio)benzoyl)pent-1-en-1-yl)-4-methyl-*N*-phenylbenzenesulfonamide, 3f

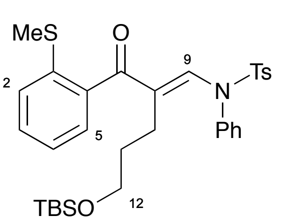

Prepared according to General Procedure B, using 2-(methylthio)benzaldehyde (40 μL, 0.300 mmol, 1.0 equiv.) and ynamide **2f** (146 mg, 0.330 mmol, 1.1 equiv.). The crude material was purified by column chromatography (petrol/EtOAc (95:5)) to give **3f** as a yellow oil (151 mg, 0.253 mmol, 85%); **R*f*** 0.31 (petrol/EtOAc (80:20)); **IR** (thin film, ν_{max}/cm^{-1}) 2953, 2927, 2856, 1650, 1612, 1493,

1434, 1367, 1171, 1125, 1088; **^{1}H NMR** (400 MHz, $CDCl_3$) δ_H 7.48-7.44 (2H, m, Ar*H* and H9), 7.41-7.32 (4H, m, Ar*H*), 7.30-7.17 (6H, m, Ar*H*), 7.09 (2H, d, *J* = 8.0 Hz, Ar*H*), 3.18 (2H, t, *J* = 7.0 Hz, H12), 2.43 (3H, s, TsC*H*$_3$), 2.41 (3H, s, SC*H*$_3$), 1.71-1.67 (2H, m, H10), 1.32-1.25 (2H, m, H11), 0.83 (9H, s, SiC(C*H*$_3$)$_3$), –0.04 (6H, m, Si(C*H*$_3$)$_2$); **^{13}C NMR** (101 MHz, $CDCl_3$) δ_C 197.6, 144.9, 144.2, 140.2, 137.7, 136.9, 133.9, 130.2, 129.7, 129.7, 129.4, 129.4, 128.3, 128.0, 127.7, 126.0, 125.4, 63.3, 30.9, 26.1, 21.8, 20.6, 18.5, 17.2, -5.1; **HRMS** (ESI$^+$) calc. for $C_{32}H_{42}O_4NS_2Si$ $[M+H]^+$ 596.2319, found 596.2318.

(*E*)-4-Methyl-*N*-(3-(2-(methylthio)phenyl)-3-oxo-2-phenylprop-1-en-1-yl)-*N*-phenylbenzenesulfonamide, 3g

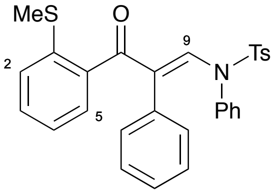

Prepared according to General Procedure B, using 2-(methylthio)benzaldehyde (40 μL, 0.300 mmol, 1.0 equiv.) and ynamide **2g** (115 mg, 0.330 mmol, 1.1 equiv.). The crude material was purified by column chromatography (petrol/EtOAc (90:10)) to give **3g** as colourless needles (114 mg, 0.228 mmol, 76%); **R*f*** 0.18 (petrol/EtOAc (80:20)); **IR** (thin film, ν_{max}/cm^{-1}) 2922, 1657, 1614, 1492, 1447, 1324, 1122, 1088; **^{1}H NMR** (400 MHz, $CDCl_3$) δ_H 7.89 (1H, s, H9), 7.47 (1H, ddd, *J* = 8.0, 7.0 and 1.5 Hz, Ar*H*), 7.41 (1H, dd, *J* = 8.0 and 1.0 Hz, Ar*H*), 7.36 (1H, dd, *J* = 7.5 and 1.5 Hz, Ar*H*), 7.29 (1H, td, *J* = 7.5 and 1.0 Hz, Ar*H*), 7.26 (2H, d, *J* = 8.5 Hz, Ts*H*), 7.21 (2H, d, *J* = 8.5 Hz, Ts*H*), 6.98 (1H, tt, *J* = 7.5 and 1.0 Hz, Ar*H*), 6.92-6.88 (5H, m, Ar*H*), 6.82-6.78 (2H, m, Ar*H*), 6.71 (2H, d, *J* = 8.5 Hz, Ar*H*), 2.45 (3H, s, SC*H*$_3$), 2.44 (3H, s, TsC*H*$_3$); **^{13}C NMR** (101 MHz, $CDCl_3$) δ_C 196.7, 145.0, 143.9, 140.4, 137.0, 135.9, 133.9, 132.7, 130.5, 130.3, 130.0, 129.7, 128.5, 128.4, 128.4, 128.0, 127.7, 127.4, 126.9, 125.5, 125.5, 21.8, 17.3; **HRMS** (ESI$^+$) calc. for $C_{29}H_{26}O_3NS_2$ $[M+H]^+$ 500.1349, found 500.1343.

(*E*)-*N*-(2-(4-Methoxyphenyl)-3-(2-(methylthio)phenyl)-3-oxoprop-1-en-1-yl)-4-methyl-*N*-phenylbenzenesulfonamide, 3h

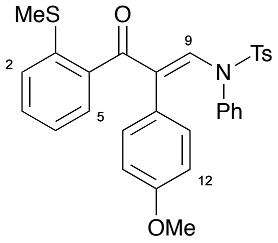

Prepared according to General Procedure B, using 2-(methylthio)benzaldehyde (40 μL, 0.300 mmol, 1.0 equiv.) and ynamide **2h** (125 mg, 0.330 mmol, 1.1 equiv.). The crude material was purified by column chromatography (petrol/EtOAc (80:20)) to give **3h** as a yellow oil (106 mg, 0.200 mmol, 67%); **R*f*** 0.42 (petrol/EtOAc (60:40)); **IR** (thin film, ν_{max}/cm^{-1}) 2930, 1600, 1511, 1492, 1365, 1289, 1170, 1084; **^{1}H NMR** (400 MHz, $CDCl_3$) δ_H 7.83 (1H, s, H9), 7.46 (1H, ddd, *J* = 8.0, 7.0 and 1.5 Hz, Ar*H*), 7.40 (1H, d, *J* = 8.0 Hz, Ar*H*), 7.35 (1H, dd, *J* = 7.5 and 1.5 Hz, Ar*H*), 7.28 (1H, ddd, *J* = 7.5 and 1.0 Hz, Ar*H*), 7.24 (2H, d, *J* = 8.5 Hz, Ar*H*), 7.19 (2H, d, *J* = 8.5 Hz, Ar*H*), 7.01 (1H, tt, *J* = 7.5 and 1.0 Hz, Ar*H*), 6.92 (2H, app. t, *J* = 7.5 Hz, Ar*H*), 6.73-6.70 (4H, m, Ar*H*), 6.44 (2H, d, *J* = 8.5 Hz, Ar*H*), 3.65 (3H, s, OCH_3), 2.43 (6H, m, $TsCH_3$ and SCH_3); **^{13}C NMR** (101 MHz, $CDCl_3$) δ_C 197.0, 158.4, 144.9, 143.7, 140.5, 137.0, 136.0, 133.9, 131.6, 130.3, 129.9, 129.7, 128.4 (2C), 128.3, 128.0, 127.6, 125.5, 125.3, 125.0, 113.0, 55.3, 21.8, 17.3; **HRMS** (ESI^+) calc. for $C_{30}H_{28}O_4NS_2$ $[M+H]^+$ 530.1454, found 530.1446.

(*E*)-4-Methyl-*N*-(3-(2-(methylthio)phenyl)-3-oxo-2-(4-(trifluoromethyl)phenyl)prop-1-en-1-yl)-*N*-phenylbenzenesulfonamide, 3i

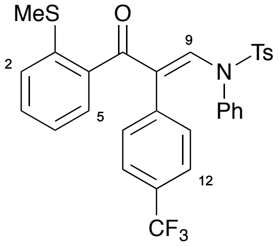

Prepared according to General Procedure B, using 2-(methylthio)benzaldehyde (40 μL, 0.300 mmol, 1.0 equiv.) and ynamide **2i** (137 mg, 0.330 mmol, 1.1 equiv.). The crude material was purified by column chromatography (petrol/EtOAc (90:10)) to give **3i** as a yellow oil (145 mg, 0.255 mmol, 85%); **R*f*** 0.22 (petrol/EtOAc (80:20)); **IR** (thin film, ν_{max}/cm^{-1}) 2951, 1605, 1516, 1434, 1406, 1323, 1169, 1122; **^{1}H NMR** (500 MHz, $CDCl_3$) δ_H 7.96 (1H, s, H9), 7.50 (1H, ddd, *J* = 8.0, 7.0 and 1.5 Hz,

Ar*H*), 7.43 (1H, d, *J* = 8.0 Hz, Ar*H*), 7.37 (1H, dd, *J* = 7.5 and 1.5 Hz, Ar*H*), 7.32 (1H, td, *J* = 7.0 and 1.0 Hz, Ar*H*), 7.26 (2H, d, *J* = 8.0 Hz, Ar*H*), 7.21 (2H, d, *J* = 8.0 Hz, Ar*H*), 7.13 (2H, d, *J* = 8.0 Hz, Ar*H*), 7.00 (1H, t, *J* = 7.5 Hz, Ar*H*), 6.90-6.87 (4H, m, Ar*H*), 6.65 (2H, d, *J* = 8.0 Hz, Ar*H*), 2.46 (3H, s, TsCH_3), 2.43 (3H, s, SCH_3); **^{13}C NMR** (126 MHz, $CDCl_3$) δ_C 196.3, 145.3, 144.9, 140.0, 136.9, 136.8, 135.5, 133.7, 131.0, 130.6, 130.1, 129.8, 128.9, 128.8 (q, *J* = 32.0 Hz), 128.6, 128.3, 128.1, 127.8, 125.8, 124.1 (q, *J* = 4.0 Hz), 124.1 (q, *J* = 272.0 Hz), 123.3, 21.8, 17.3; **^{19}F NMR** (470 MHz, $CDCl_3$) δ_F –63.0; **HRMS** (ESI$^+$) calc. for $C_{30}H_{25}O_3F_3S_2$ $[M+H]^+$ 568.1222, found 568.1215.

(*E*)-*N*-(2-(3,5-Dimethoxyphenyl)-3-(2-(methylthio)phenyl)-3-oxoprop-1-en-1-yl)-4-methyl-*N*-phenylbenzenesulfonamide, 3j

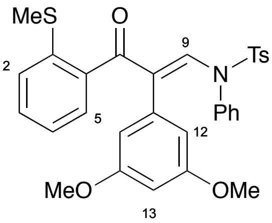

Prepared according to General Procedure B, using 2-(methylthio)benzaldehyde (40 μL, 0.300 mmol, 1.0 equiv.) and ynamide **2j** (135 mg, 0.330 mmol, 1.1 equiv.). The crude material was purified by column chromatography (petrol/EtOAc (60:40)) to give **3j** as a colourless oil (130 mg, 0.232 mmol, 77%); **R*f*** 0.37 (petrol/EtOAc (60:40)); **IR** (thin film, ν_{max}/cm^{-1}) 2925, 1650, 1590, 1492, 1455, 1424, 1365, 1306, 1171, 1152; **^{1}H NMR** (400 MHz, $CDCl_3$) δ_H 7.87 (1H, s, H9), 7.48 (1H, ddd, *J* = 8.5, 7.5 and 1.5 Hz, Ar*H*), 7.41 (1H, d, *J* = 8.0 Hz, Ar*H*), 7.37 (1H, dd, *J* = 7.5 and 1.5 Hz, Ar*H*), 7.31-7.27 (3H, m, Ar*H*), 7.23 (2H, d, *J* = 8.0 Hz, Ar*H*), 7.05 (1H, tt, *J* = 7.5 and 2.0 Hz, Ar*H*), 6.96 (2H, t, *J* = 7.5 Hz, Ar*H*), 6.75 (2H, d, *J* = 7.5 Hz, Ar*H*), 6.01 (1H, t, *J* = 2.0 Hz, H13), 5.95 (2H, d, *J* = 2.0 Hz, H12), 3.61 (6H, s, OCH_3), 2.46 (3H, s, SCH_3), 2.45 (3H, s, TsCH_3); **^{13}C NMR** (101 MHz, $CDCl_3$) δ_C 196.5, 159.6, 145.0, 143.7, 140.2, 137.0, 135.8, 134.6, 133.9, 130.4, 129.9, 129.7, 128.5, 128.4, 128.2, 128.0, 127.6, 125.5, 125.1, 108.8, 99.7, 55.2, 21.8, 17.3; **HRMS** (ESI$^+$) calc. for $C_{31}H_{30}O_5NS_2$ $[M+H]^+$ 560.1560, found 560.1560.

(*E*)-4-Methyl-*N*-(3-(2-(methylthio)phenyl)-3-oxo-2-(thiophen-3-yl)prop-1-en-1-yl)-*N*-phenylbenzenesulfonamide, 3k

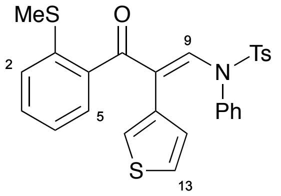

Prepared according to General Procedure B, using 2-(methylthio)benzaldehyde (40 μL, 0.300 mmol, 1.0 equiv.) and ynamide **2k** (117 mg, 0.330 mmol, 1.1 equiv.). The crude material was purified by column chromatography (petrol/EtOAc (90:10)) to give **3k** as a yellow oil (125 mg, 0.247 mmol, 82%); $\mathbf{R}_f$ 0.16 (petrol/EtOAc (80:20)); **IR** (thin film, ν_{max}/cm^{-1}) 3063, 2923, 1650, 1610, 1592, 1363, 1291, 1170, 1059; **^{1}H NMR** (400 MHz, $CDCl_3$) δ_H 7.87 (1H, s, H9), 7.46 (1H, td, *J* = 7.5 and 1.5 Hz, Ar*H*), 7.39 (1H, d, *J* = 7.5 Hz, Ar*H*), 7.34 (1H, dd, *J* = 7.5 and 1.5 Hz, Ar*H*), 7.29-7.23 (3H, m, Ar*H*), 7.19 (2H, d, *J* = 8.5 Hz, Ar*H*), 7.08 (1H, tt, *J* = 7.5 and 2.0 Hz, Ar*H*), 6.98 (2H, dd, *J* = 8.0 and 7.5 Hz, Ar*H*), 6.80 (1H, dd, *J* = 5.0 and 3.0 Hz, H14), 6.77 (2H, d, *J* = 8.0 Hz, Ar*H*), 6.70 (1H, dd, *J* = 3.0 and 1.0 Hz, H11), 6.52 (1H, dd, *J* = 5.0 and 1.0 Hz, H13), 2.43 (6H, s, TsC*H*$_3$ and SC*H*$_3$); **^{13}C NMR** (101 MHz, $CDCl_3$) δ_C 196.5, 145.0, 144.0, 140.2, 137.1, 135.8, 133.8, 132.2, 130.4, 129.7, 129.4, 129.1, 128.6, 128.5, 128.4, 128.0, 127.6, 125.6, 125.5, 123.8, 120.5, 21.8, 17.2; **HRMS** (ESI$^+$) calc. for $C_{27}H_{24}O_3NS_3$ $[M+H]^+$ 506.0913, found 506.0905.

(*E*)-*N*-(2-(Cyclohex-1-en-1-yl)-3-(2-(methylthio)phenyl)-3-oxoprop-1-en-1-yl)-4-methyl-*N*-phenylbenzenesulfonamide, 3l

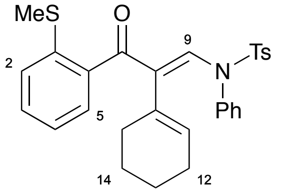

Prepared according to General Procedure B, using 2-(methylthio)benzaldehyde (40 μL, 0.300 mmol, 1.0 equiv.) and ynamide **2l** (116 mg, 0.330 mmol, 1.1 equiv.). The crude material was purified by column chromatography (petrol/EtOAc (90:10)) to give **3l** as a yellow oil (144 mg, 0.286 mmol, 95%); $\mathbf{R}_f$ 0.22 (petrol/EtOAc (80:20)); **IR** (thin film, ν_{max}/cm^{-1}) 3063, 2927, 2858, 1646, 1602, 1493, 1434, 1365, 1290, 1170, 1079; **^{1}H NMR** (400 MHz, $CDCl_3$) δ_H 7.55 (1H, s, H9), 7.46 (1H, ddd, *J* = 8.0, 7.0 and 1.5 Hz, Ar*H*), 7.36 (1H, d, *J* = 8.0 Hz, Ar*H*), 7.33-7.19 (9H, m, Ar*H*), 6.98 (2H, d, *J* = 8.5 Hz, Ar*H*), 5.33-5.31 (1H, m, H11), 2.44 (3H, TsC*H*$_3$), 2.39 (3H, s, SC*H*$_3$), 1.68-1.63 (2H, m,

H12), 1.61-1.56 (2H, m, H15), 1.13-1.02 (4H, m, H13 and 14); **^{13}C NMR** (101 MHz, $CDCl_3$) δ_C 197.1, 144.9, 143.4, 140.3, 137.6, 137.2, 134.0, 131.5, 130.6, 130.3, 123.0, 129.7, 128.8, 128.8, 128.6, 128.0, 127.8, 127.2, 125.2, 26.7, 24.9, 21.9, 21.8, 21.3, 17.1; **HRMS** (ESI^+) calc. for $C_{29}H_{30}O_3NS_2$ $[M+H]^+$ 504.1662, found 504.1658.

(*E*)-*N*-(2-(2,4-*bis*(Methylthio)benzoyl)oct-1-en-1-yl)-4-methyl-*N*-phenylbenzenesulfonamide, 3m

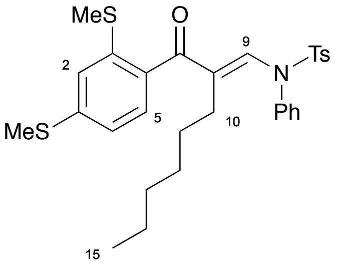

Prepared according to General Procedure B, using 2,4-*bis*(methythio)benzaldehyde (60 mg, 0.300 mmol, 1.0 equiv.) and ynamide **2a** (117 mg, 0.330 mmol, 1.1 equiv.). The crude material was purified by column chromatography (petrol/EtOAc (90:10)) to give **3m** as a yellow oil (135 mg, 0.244 mmol, 81%); **R_f** 0.24 (petrol/EtOAc (80:20): **IR** (thin film, ν_{max}/cm^{-1}) 2924, 2856, 1609, 1575, 1491, 1436, 1364, 1288, 1187, 1169, 1126, 1089; **^{1}H NMR** (500 MHz, $CDCl_3$) δ_H 7.43 (1H, s, H9), 7.39 (1H, tt, J = 7.5 and 1.0 Hz, Ar*H*), 7.34 (2H, tt, J = 8.0 and 1.5 Hz, Ar*H*), 7.27 (2H, d, J = 8.5 Hz, Ar*H*), 7.22-7.19 (4H, m, Ar*H*), 7.09 (3H, m, Ar*H*), 2.58 (3H, s, SC*H*$_3$), 2.44 (3H, s, TsC*H*$_3$), 2.41 (3H, s, SC*H*$_3$), 1.68-1.65 (2H, m, H10), 1.18-1.11 (2H, m, H14), 1.04-0.97 (4H, m, H11 and 13), 0.89-0.79 (5H, m, H12 and H15); **^{13}C NMR** (126 MHz, $CDCl_3$) δ_C 197.2, 144.9, 143.0, 142.0, 138.7, 137.9, 136.1, 134.1, 129.8, 129.7, 129.4, 129.3, 129.3, 128.1, 127.1, 124.3, 122.1, 31.6, 29.4, 27.8, 24.6, 22.6, 21.8, 17.0, 15.6, 14.2; **HRMS** (ESI^+) calc. for $C_{30}H_{36}O_3NS_3$ $[M+H]^+$ 554.1852, found 554.1855.

(*E*)-*N*-(2-(4-Methoxy-2-(methylthio)benzoyl)oct-1-en-1-yl)-4-methyl-*N*-phenylbenzenesulfonamide, 3n

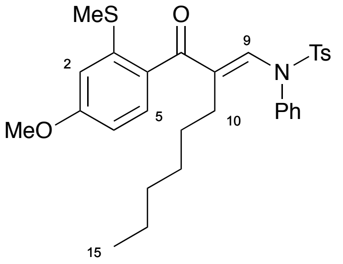

Prepared according to General Procedure B, using 4-methoxy-2-(methylthio)benzaldehyde (27 mg, 0.150 mmol, 1.0 equiv.) and ynamide **2a** (59 mg, 0.165 mmol, 1.1 equiv.). The crude material was purified by column chromatography (petrol/EtOAc (90:10)) to give **3n** as a colourless oil (61 mg, 0.113 mmol, 76%); **R*f*** 0.22 (petrol/EtOAc (80:20)); **IR** (thin film, ν_{max}/cm^{-1}) 2925, 2855, 1610, 1590, 1490, 1364, 1288, 1229, 1169, 1124, 1087; **^{1}H NMR** (400 MHz, $CDCl_3$) δ_H 7.43 (1H, s, H9), 7.42-7.33 (4H, m, Ar*H*), 7.30 (2H, d, *J* = 8.5 Hz, Ar*H*), 7.22 (2H, d, *J* = 8.5 Hz, Ar*H*), 7.11 (2H, d, *J* = 8.0 Hz, Ar*H*), 6.88 (1H, d, *J* = 2.5 Hz, Ar*H*), 6.77 (1H, dd, *J* = 8.5 and 2.5 Hz, Ar*H*), 3.93 (3H, s, OCH_3), 2.44 (3H, s, $TsCH_3$), 2.41 (3H, s, SCH_3), 1.73-1.69 (2H, m, H10), 1.22-1.12 (2H, m, H14), 1.08-0.99 (4H, m, H11 and 13), 0.90-0.79 (5H, m, H12 and 15); **^{13}C NMR** (101 MHz, $CDCl_3$) δ_C 197.0, 161.3, 144.8, 141.9, 141.0, 138.0, 134.0, 131.3, 129.7, 129.6, 129.2, 129.2, 129.1, 128.0, 127.7, 112.7, 109.1, 55.6, 31.5, 29.3, 27.7, 24.9, 22.6, 21.8, 16.6, 14.1; **HRMS** (ESI^+) calc. for $C_{30}H_{36}O_4NS_2$ $[M+H]^+$ 538.2080, found 538.2084.

(*E*)-4-Methyl-*N*-(2-(2-(methylthio)-4-(trifluoromethyl)benzoyl)oct-1-en-1-yl)-*N*-phenylbenzenesulfonamide, 3o

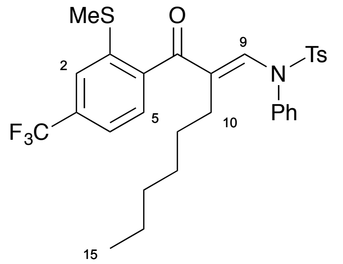

Prepared according to General Procedure B, using 2-(methylthio)-4-(trifluoromethyl)benzaldehyde (27 mg, 0.150 mmol, 1.0 equiv.) and ynamide **2a** (59 mg, 0.165 mmol, 1.1 equiv.). The crude material was purified by column chromatography (petrol/EtOAc (95:5)) to give **3o** as a colourless oil (56 mg, 0.097 mmol, 65%); **R*f*** 0.23 (petrol/EtOAc (90:10)); **IR** (thin film, ν_{max}/cm^{-1}) 2927, 2857, 1651, 1609, 1492, 1455, 1368, 1318, 1169, 1127, 1082; **^{1}H NMR** (500 MHz, $CDCl_3$) δ_H 7.57 (1H, s, H9), 7.53 (1H, d, *J* = 8.0 Hz, Ar*H*), 7.43 (1H, tt, *J* = 7.5 and 1.0 Hz, Ar*H*), 7.41 (1H, s, Ar*H*), 7.37

(2H, t, J = 8.0 Hz, Ar*H*), 7.33 (1H, d, J = 8.0 Hz, Ar*H*), 7.23-7.19 (4H, m, Ts*H*), 7.10 (2H, d, J = 8.5 Hz, Ar*H*), 2.46 (3H, s, SCH_3), 2.44 (3H, s, TsCH_3), 1.67-1.64 (2H, m, H10), 1.20-1.13 (2H, m, H14), 1.07-0.99 (4H, m, H11 and 13), 0.88-0.82 (2H, m, H12), 0.82 (3H, t, J = 7.5 Hz, H15); **^{13}C NMR** (126 MHz, $CDCl_3$) δ_C 196.4, 145.2, 144.6, 143.2, 138.6, 137.4, 133.8, 132.1 (q, J = 32.5 Hz), 129.8, 129.8, 129.6, 129.3, 128.4, 128.0, 125.7, 123.8 (q, J = 272.0 Hz), 123.5 (q, J = 4.0 Hz), 122.0 (q, J = 4.0 Hz), 31.6, 29.4, 27.9, 24.0, 22.6, 21.8, 16.7, 14.2; **^{19}F NMR** (470 MHz, $CDCl_3$) δ_F –62.8; **HRMS** (ESI$^+$) calc. for $C_{30}H_{22}O_3NF_3S_2$ [M+H]$^+$ 576.1849, found 576.1854.

(*E*)-*N*-(2-(4-Bromo-2-(methylthio)benzoyl)oct-1-en-1-yl)-4-methyl-*N*-phenylbenzenesulfonamide, 3p

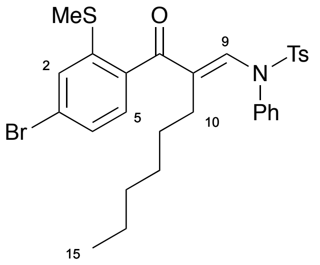

Prepared according to General Procedure B, using 4-bromo-2-(methylthio)benzaldehyde (69 mg, 0.300 mmol, 1.0 equiv.) and ynamide **2a** (117 mg, 0.330 mmol, 1.1 equiv.). The crude material was purified by column chromatography (petrol/EtOAc (95:5)) to give **3p** as a colourless oil (154 mg, 0.263 mmol, 88%); **R*f*** 0.25 (petrol/EtOAc (90:10)); **IR** (thin film, ν_{max}/cm^{-1}) 3065, 2954, 2925, 2856, 1648, 1609, 1591, 1571, 1366, 1290, 1171, 1128; **^{1}H NMR** (400 MHz, $CDCl_3$) δ_H 7.44-7.43 (2H, m, Ar*H* and H9), 7.41-7.33 (4H, m, Ar*H*), 7.26 (2H, d, J = 8.5 Hz, Ar*H*), 7.22 (2H, d, J = 8.5 Hz, Ar*H*), 7.11-7.07 (3H, m, Ar*H*), 2.44 (3H, s, TsCH_3), 2.42 (3H, s, SCH_3), 1.65-1.61 (2H, m, H10), 1.19-1.10 (2H, m, H14), 1.03-0.96 (4H, m, H11 and 13), 0.85-0.78 (5H, m, H13 and 15); **^{13}C NMR** (101 MHz, $CDCl_3$) δ_C 196.7, 145.1, 143.9, 139.9, 138.3, 137.5, 133.9, 129.8, 129.8, 129.7, 129.5, 129.3_2, 129.2_9, 128.0_4, 128.0_0, 126.1, 124.4, 31.6, 29.4, 27.9, 24.2, 22.6, 21.8, 16.8, 14.2; **HRMS** (ESI$^+$) calc. for $C_{29}H_{33}O_3NBrS_2$ [M+H]$^+$ 586.1080, found 586.1079.

(*E*)-4-Methyl-*N*-(2-(3-(methylthio)thiophene-2-carbonyl)oct-1-en-1-yl)-*N*-phenylbenzenesulfonamide, 3q

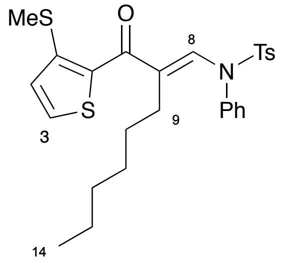

Prepared according to General Procedure B, using 3-(methylthio)thiophene-2-carbaldehyde (47 mg, 0.300 mmol, 1.0 equiv.) and ynamide **2a** (117 mg, 0.330 mmol, 1.1 equiv.). The crude material was purified by column chromatography (petrol/EtOAc (90:10)) to give **3q** as a yellow oil (100 mg, 0.195 mmol, 65%); **R*f*** 0.23 (petrol/EtOAc (80:20)); **IR** (thin film, ν_{max}/cm^{-1}) 2926, 1601, 1478, 1392, 1355, 1168, 1120, 1088; **^{1}H NMR** (400 MHz, $CDCl_3$) δ_H 7.62 (1H, s, H8), 7.57 (1H, d, *J* = 5.5 Hz, H3), 7.43 (2H, d, *J* = 8.5 Hz, Ts*H*), 7.35-7.29 (3H, m, Ar*H*), 7.21 (2H, d, *J* = 8.5 Hz, Ts*H*), 7.15-7.12 (2H, m, Ar*H*), 7.07 (1H, d, *J* = 5.5 Hz, H2), 2.50 (3H, s, SC*H*$_3$), 2.40 (3H, s, TsC*H*$_3$), 1.80-1.76 (2H, m, H9), 1.14-1.05 (2H, m, H13), 0.99-0.92 (4H, m, H10 and 12), 0.87-0.82 (2H, m, H11), 0.76 (3H, t, *J* = 7.5 Hz, H14); **^{13}C NMR** (101 MHz, $CDCl_3$) δ_C 188.2, 145.8, 144.7, 138.8, 136.7, 134.1, 131.6, 130.6, 130.2, 129.6, 129.2, 129.2, 128.7, 128.0, 126.7, 31.4, 29.2, 27.4, 26.6, 22.5, 21.7, 16.9, 14.1; **HRMS** (ESI$^+$) calc. for $C_{27}H_{32}NO_3S_3$ $[M+H]^+$ 514.1539, 514.1539.

(*E*)-4-Methyl-*N*-(2-(4-(methylthio)-5,6-dihydro-2*H*-pyran-3-carbonyl)oct-1-en-1-yl)-*N*-phenylbenzenesulfonamide, 3r

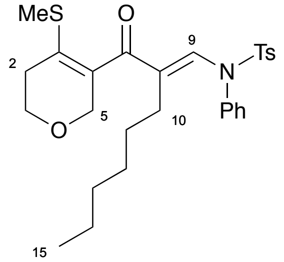

Prepared according to General Procedure B, using 4-(methylthio)-5,6-dihydro-2*H*-pyran-3-carbaldehyde (69 mg, 0.300 mmol, 1.0 equiv.) and ynamide **2a** (117 mg, 0.330 mmol, 1.1 equiv.). The crude material was purified by column chromatography (petrol/EtOAc (80:20)) to give **3r** as a colourless oil (141 mg, 0.274 mmol, 92%); **R*f*** 0.18 (petrol/EtOAc (80:20)); **IR** (thin film, ν_{max}/cm^{-1}) 2956, 2927, 2855, 1610, 1492, 1455, 1365, 1288, 1170, 1130; **^{1}H NMR** (400 MHz, $CDCl_3$) δ_H 8.04 (1H, s, H9), 7.50 (2H, d, *J* = 8.5 Hz, Ar*H*), 7.39 (1H, tt, *J* = 7.5 and 1.0 Hz, Ar*H*), 7.33 (2H, t, *J* = 7.5

Hz, Ar*H*), 7.28 (2H, d, *J* = 8.5 Hz, Ar*H*), 7.06 (2H, d, *J* = 7.5 Hz, Ar*H*), 4.27 (2H, t, *J* = 2.5 Hz, H5), 3.99 (2H, t, *J* = 5.5 Hz, H2), 2.51-2.47 (2H, m, H3), 2.44 (3H, s, TsCH_3), 2.21 (3H, s, SCH_3), 1.52-1.48 (2H, m, H10), 1.17-1.08 (2H, m, H14), 1.01-0.89 (4H, m, H11 and 13), 0.81-0.76 (5H, m, H12 and 15); **^{13}C NMR** (101 MHz, $CDCl_3$) δ_C 196.9, 145.1, 142.5, 137.4, 136.0, 134.2, 130.4, 129.9, 129.8, 129.4, 129.3, 128.1, 124.9, 67.9, 65.0, 31.6, 29.4, 28.6, 28.0, 23.6, 22.6, 21.8, 14.3, 14.2; **HRMS** (ESI$^+$) calc. for $C_{28}H_{36}O_4NS_2$ $[M+H]^+$ 514.2080, found 514.2081.

(*E*)-*N*-(2-(2-Benzyl-3-morpholino-3-oxopropanoyl)oct-1-en-1-yl)-4-methyl-*N*-phenylbenzenesulfonamide, 3s

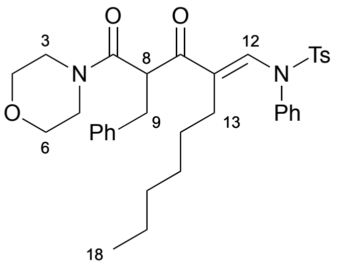

Prepared according to General Procedure B, using 2-benzyl-3-morpholino-3-oxopropanal (74 mg, 0.300 mmol, 1.0 equiv.) and ynamide **2a** (117 mg, 0.330 mmol, 1.1 equiv.). The crude material was purified by column chromatography (petrol/EtOAc (20:80)) to give **3s** as a colourless oil (154 mg, 0.255 mmol, 85%); **R*f*** 0.38 (petrol/EtOAc (40:60)); **IR** (thin film, ν_{max}/cm^{-1}) 2957, 2926, 2856, 1666, 1634, 1593, 1492, 1454, 1432, 1361, 1273, 1167; **^{1}H NMR** (400 MHz, $CDCl_3$) δ_H 7.91 (1H, s, H12), 7.48 (2H, d, 8.5 Hz, Ar*H*), 7.37-7.22 (10H, m, Ar*H*), 6.92 (2H, d, *J* = 7.5 Hz, Ar*H*), 4.35 (1H, t, *J* = 7.5 Hz, H8), 3.80-3.74 (1H, m, H3), 3.68-3.63 (1H, m, H6), 3.55-3.45 (4H, m, H2, 3, 5 and 6), 3.31 (1H, dd, *J* = 14.0 and 8.5 Hz, H9), 3.25-3.20 (2H, m, H5), 3.05-3.00 (1H, m, H2), 2.44 (3H, s, TsCH_3), 1.62-1.55 (1H, m, H13), 1.52-1.45 (1H, m, H13), 1.16-1.07 (2H, m, H17), 0.98-0.91 (2H, m, H16), 0.83-0.71 (7H, m, H14, 15 and 18); **^{13}C NMR** (101 MHz, $CDCl_3$) δ_C 195.3, 168.3, 145.1, 139.0, 138.5, 137.8, 134.2, 129.8, 129.5, 129.4, 129.3, 129.2, 128.7, 128.0, 126.8, 126.6, 66.7, 66.2, 53.6, 46.3, 42.6, 36.4, 31.5, 29.3, 27.8, 24.8, 22.6, 21.8, 14.2; **HRMS** (ESI$^+$) calc. for $C_{35}H_{41}O_5N_2S$ $[M+H]^+$ 601.2742, found 601.2742.

(*E*)-*N*-(4-((Ethylthio)methyl)-2-hexyl-3-oxodec-1-en-1-yl)-4-methyl-*N*-phenylbenzenesulfonamide, 3t

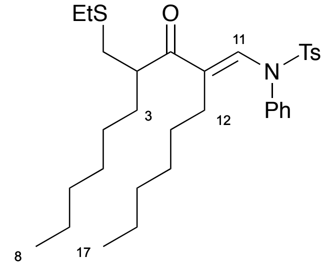

Prepared according to General Procedure B, using 2-((ethylthio)methyl)octanal (28 mg, 0.139 mmol, 1.0 equiv.) and ynamide **2a** (55 mg, 0.153 mmol, 1.1 equiv.). The crude material was purified by column chromatography (petrol/EtOAc (98:2)) to give **3t** as a yellow oil (54 mg, 0.097 mmol, 70%); **R*f*** 0.35 (petrol/EtOAc (90:10)); **IR** (thin film, ν_{max}/cm^{-1}) 2955, 2925, 2855, 1656, 1617, 1593, 1492, 1455, 1366, 1267, 1168, 1127, 1088; **^{1}H NMR** (400 MHz, $CDCl_3$) δ_H 7.97 (1H, s, H11), 7.52 (2H, d, *J* = 8.5 Hz, Ts*H*), 7.36 (1H, tt, *J* = 7.5 and 1.5 Hz, Ph*H*), 7.31 (2H, tt, *J* = 7.5 and 1.5 Hz, Ph*H*), 7.26 (2H, d, *J* = 8.5 Hz, Ts*H*), 7.04 (2H, dt, *J* = 7.5 and 1.5 Hz, Ph*H*), 3.35-3.29 (1H, m, H2), 2.81 (1H, dd, *J* = 13.0 and 9.0 Hz, H1), 2.61 (1H, dd, *J* = 13.0 and 5.5 Hz, H1), 2.53 (2H, qd, *J* = 7.5 and 1.5 Hz, SCH_2CH_3), 2.43 (3H, s, $TsCH_3$), 1.71-1.62 (2H, m, H3 and H12), 1.57-1.49 (2H, m, H3 and H12), 1.31-1.20 (11H, m, H5, 6, 7, 14 and SCH_2CH_3), 1.17-1.08 (2H, m, H16), 1.01-0.94 (2H, m, H15), 0.90-0.77 (10H, m, H4, 8, 13 and 17); **^{13}C NMR** (101 MHz, $CDCl_3$) δ_C 203.4, 144.9, 138.5, 138.1, 134.2, 129.8, 129.5, 129.2, 129.0, 128.7, 128.0, 45.9, 34.7, 33.8, 31.8, 31.6, 29.5, 29.3, 28.0, 27.6, 27.0, 24.7, 22.7, 22.6, 21.8, 14.9, 14.2_1, 14.1_5; **HRMS** (ESI^+) calc. for $C_{32}H_{48}O_3NS_2$ $[M+H]^+$ 558.3070, found 558.3071.

(*E*)-*N*-(2-(2-Benzyl-3-(ethylthio)propanoyl)oct-1-en-1-yl)-4-methyl-*N*-phenylbenzenesulfonamide, 3u

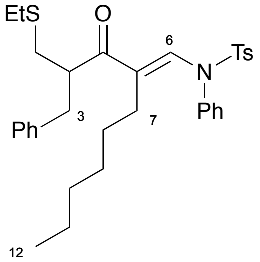

Prepared according to General Procedure B, using 2-benzyl-3-(ethylthio)propanal (62 mg, 0.300 mmol, 1.0 equiv.) and ynamide **2a** (117 mg, 0.330 mmol, 1.1 equiv.). The crude material was purified by column chromatography (petrol/Et_2O (90:10)) to give **3u** as a colourless oil (129 mg,

0.229 mmol, 76%); **R*f*** 0.27 (petrol/EtOAc (90:10)); **IR** (thin film, ν_{max}/cm^{-1}) 2956, 2926, 2856, 1655, 1616, 1593, 1492, 1454, 1366, 1267, 1168, 1126, 1089; **^{1}H NMR** (400 MHz, $CDCl_3$) δ_H 7.79 (1H, s, H6), 7.43 (2H, d, *J* = 8.0 Hz, Ar*H*), 7.33-7.18 (8H, m, Ar*H*), 7.12 (2H, d, *J* = 7.0 Hz, Ar*H*), 6.81 (2H, d, *J* = 8.0 Hz, Ar*H*), 3.66-3.59 (1H, m, H2), 2.93-2.83 (3H, m, H1 and 3), 2.66 (1H, dd, *J* = 13.0 and 5.0 Hz, H1), 2.58-2.49 (2H, m, SCH_2CH_3), 2.46 (3H, s, $TsCH_3$), 1.50-1.46 (2H, m, H7), 1.24 (3H, t, *J* = 7.5 Hz, SCH_2CH_3), 1.15-1.06 (2H, m, H11), 0.97-0.90 (2H, m, H10), 0.80-0.58 (7H, m, H8, 9 and 12); **^{13}C NMR** (126 MHz, $CDCl_3$) δ_C 202.7, 144.9, 139.3, 139.0, 138.1, 134.3, 129.8, 129.4, 129.2, 129.1, 128.9 (2C), 128.6, 128.0, 126.3, 48.0, 39.9, 34.4, 31.6, 29.3, 27.8, 27.0, 24.6, 22.6, 21.8, 14.9, 14.2; **HRMS** (ESI^+) calc. for $C_{33}H_{42}O_3NS_2$ $[M+H]^+$ 564.2601, found 564.2599.

(*E*)-4-Methyl-*N*-(2-(2-(methylthio)cyclohex-1-ene-1-carbonyl)oct-1-en-1-yl)-*N*-phenylbenzenesulfonamide, 3v

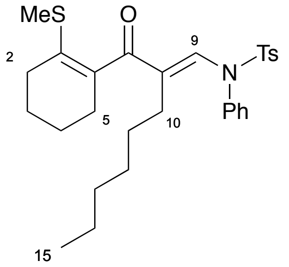

Prepared according to General Procedure B, using 2-(methylthio)cyclohex-1-ene-1-carbaldehyde (23 mg, 0.150 mmol, 1.0 equiv.) and ynamide **2a** (59 mg, 0.165 mmol, 1.1 equiv.). The crude material was purified by column chromatography (petrol/EtOAc (90:10)) to give **3v** as a yellow oil (67 mg, 0.131 mmol, 87%); **R*f*** 0.38 (petrol/EtOAc (80:20)); **IR** (thin film, ν_{max}/cm^{-1}) 2926, 2857, 1646, 1614, 1492, 1454, 1365, 1285, 1169, 1126, 1088; **^{1}H NMR** (400 MHz, $CDCl_3$) δ_H 7.86 (1H, s, H9), 7.40 (2H, d, *J* = 8.0 Hz, Ar*H*), 7.32-7.23 (3H, m, Ar*H*), 7.20 (2H, d, *J* = 8.0 Hz, Ar*H*), 6.99 (2H, d, *J* = 8.0 Hz, Ar*H*), 2.36 (3H, s, $TsCH_3$), 2.33-2.30 (2H, m, H2), 2.21-2.18 (2H, m, H5), 2.06 (3H, s, SCH_3), 1.82-1.76 (2H, m, H3), 1.73-1.67 (2H, m, H4), 1.48-1.44 (2H, m, H10), 1.11-1.02 (2H, m, H14), 0.95-0.84 (4H, m, H11 and 13), 0.76-0.69 (5H, m, H12 and 15); **^{13}C NMR** (101 MHz, $CDCl_3$) δ_C 200.0, 144.9, 141.8, 138.6, 137.7, 134.2, 130.9, 129.8, 129.7, 129.2, 129.2, 128.0, 124.9, 31.6, 29.4, 29.3_2, 29.2_6, 27.8, 23.5 (2C), 22.6, 22.1, 21.8, 14.8, 14.2; **HRMS** (ESI^+) calc. for $C_{29}H_{38}O_3NS_2$ $[M+H]^+$ 512.2288, found 512.2286.

3.3 Branched hydroacylation products

(*Z*)-4-Methyl-*N*-(1-(2-(methylthio)phenyl)-1-oxonon-2-en-2-yl)-*N*-phenylbenzenesulfonamide, 4a

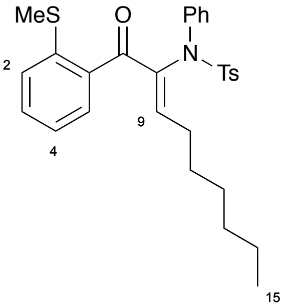

Method A: Prepared according to General Procedure B, using PNP(Cy) (13 mg, 0.030 mmol, 5 mol%), 2-(methylthio)benzaldehyde (91 mg, 0.600 mmol, 1.0 equiv.) and ynamide **2a** (235 mg, 0.660 mmol, 1.1 equiv.). The crude material was purified by column chromatography (petrol/EtOAc (90:10)) to give **4a** as a colourless oil (183 mg, 0.360 mmol, 60%).

Method B (4 mmol scale reaction): Prepared according to General Procedure B, using PNP(Cy) (42 mg, 0.10 mmol, 2.5 mol%), 2-(methylthio)benzaldehyde (609 mg, 4.00 mmol, 1.0 equiv.) and ynamide **2a** (1.56 g, 4.40 mmol, 1.1 equiv.). The crude material was purified by column chromatography (petrol/EtOAc (90:10)) to give **4a** as a colourless amorphous solid (1.38 g, 2.72 mmol, 68%) and 3a as a colourless oil (0.51 g, 1.00 mmol, 25%).

R*f* 0.37 (petrol/EtOAc (80:20)); **IR** (thin film, ν_{max}/cm^{-1}) 2970, 1632, 1555, 1466, 1378, 1160, 1128; **^{1}H NMR** (400 MHz, $CDCl_3$) δ_H 7.61-7.57 (3H, m, Ar*H*), 7.45 (2H, d, *J* = 8.0 Hz, Ar*H*), 7.35 (1H, ddd, *J* = 8.0, 7.5 and 1.5 Hz, Ar*H*), 7.25 (1H, d, *J* = 8.0 Hz, Ar*H*), 7.17-7.11 (3H, m, Ar*H*), 7.06-7.02 (3H, m, Ar*H*), 6.27 (1H, t, *J* = 7.5 Hz, H9), 2.35 (3H, s, SCH_3), 2.26-2.23 (2H, dt, *J* = 7.5 and 7.0 Hz, H10), 2.23 (3H, s, $TsCH_3$), 1.17 (8H, m, H11-14), 0.74 (3H, t, *J* = 7.5 Hz, H15); **^{13}C NMR** (101 MHz, $CDCl_3$) δ_C 194.6, 151.4, 143.6, 140.8, 140.5, 139.0, 137.4, 135.7, 131.2, 130.5, 129.0, 129.0, 128.7, 126.4, 126.3, 125.7, 124.3, 31.4, 29.1, 28.8, 27.6, 22.5, 21.6, 16.3, 14.0; **HRMS** (ESI^+) calc. for $C_{29}H_{33}NO_3S_2Na$ $[M+Na]^+$ 530.1794, found 530.1793.

(*Z*)-*N*-Methyl-*N*-(1-(2-(methylthio)phenyl)-1-oxonon-2-en-2-yl)methanesulfonamide, 4d

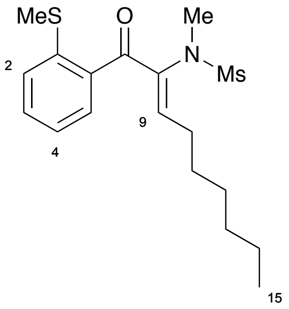

Prepared according to General Procedure B, using PNP(Cy) (6 mg, 0.015 mmol, 5 mol%), 2-(methylthio)benzaldehyde (40 μL, 0.300 mmol, 1.0 equiv.) and ynamide **2d** (72 mg, 0.330 mmol, 1.1 equiv.). The crude material was purified by column chromatography (petrol/EtOAc (90:10)) to give **4d** as a colourless oil (67 mg, 0.181 mmol, 60%); $\mathbf{R}_f$ 0.35 (petrol/EtOAc (80:20)); **IR** (thin film, ν_{max}/cm^{-1}) 2955, 2926, 2856, 1662, 1632, 1463, 1433, 1337, 1228, 1146; ^{1}**H NMR** (400 MHz, $CDCl_3$) δ_H 7.42 (1H, t, *J* = 7.5 Hz, Ar*H*), 7.34 (1H, d, *J* = 7.5 Hz, Ar*H*), 7.30 (1H, d, *J* = 7.5 Hz, Ar*H*), 7.20 (1H, t, *J* = 7.5 Hz, Ar*H*), 6.49 (1H, t, *J* = 7.5 Hz, H9), 3.19 (3H, s, MsC*H*$_3$), 3.09 (3H, s, NC*H*$_3$), 2.46 (2H, quart., *J* = 7.5 Hz, H10), 2.43 (3H, s, SC*H*$_3$), 1.44-1.37 (2H, m, H11), 1.32-1.18 (6H, m, H12-14), 0.84 (3H, t, *J* = 7.0 Hz, H15); ^{13}**C NMR** (101 MHz, $CDCl_3$) δ_C 194.7, 155.2, 139.6, 138.1, 137.8, 131.0, 128.8, 127.5, 124.9, 38.6, 36.9, 31.5, 29.5, 29.2, 28.0, 22.6, 16.9, 14.1; **HRMS** (ESI$^+$) calc. for $C_{18}H_{27}O_3NNaS_2$ $[M+Na]^+$ 392.1325, found 392.1324.

(*Z*)-*N*-(6-Chloro-1-(2-(methylthio)phenyl)-1-oxohex-2-en-2-yl)-4-methyl-*N*-phenylbenzenesulfonamide, 4e

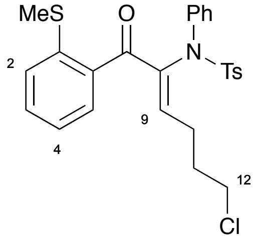

Prepared according to General Procedure B, using PNP(Cy) (4 mg, 0.010 mmol, 5 mol%), 2-(methylthio)benzaldehyde (32 mg, 0.209 mmol, 1.0 equiv.) and ynamide **2e** (80 mg, 0.230 mmol, 1.1 equiv.). The crude material was purified by column chromatography (petrol/EtOAc (90:10)) to give **4e** as a colourless oil (58 mg, 0.116 mmol, 56%); $\mathbf{R}_f$ 0.18 (petrol/EtOAc (80:20)); **IR** (thin film, ν_{max}/cm^{-1}) 3063, 2957, 2923, 1663, 1596, 1490, 1433, 1347, 1236, 1158, 1089; ^{1}**H NMR** (400 MHz, $CDCl_3$) δ_H 7.59-7.57 (3H, m, Ar*H*), 7.46 (2H, d, *J* = 8.5 Hz, Ar*H*), 7.38 (1H, ddd, *J* = 8.0, 7.5 and

1.5 Hz, Ar*H*), 7.27 (1H, d, *J* = 8.0 Hz, Ar*H*), 7.20-7.13 (3H, m, Ar*H*), 7.11-7.07 (3H, m, Ar*H*), 7.60 (1H, t, *J* = 7.5 Hz, H9), 3.30 (2H, t, *J* = 6.5 Hz, H12), 2.43 (2H, dt, *J* = 7.5 and 7.5 Hz, H10), 2.37 (3H, s, SC*H*$_3$), 2.26 (3H, s, TsC*H*$_3$), 1.64 (2H, tt, *J* = 7.5 and 6.5 Hz, H11); **^{13}C NMR** (101 MHz, $CDCl_3$) δ_C 194.4, 148.5, 143.8, 141.5, 140.6, 139.2, 137.1, 135.6, 131.4, 130.5, 129.3, 129.1, 128.8, 126.8, 126.3, 126.0, 124.4, 44.3, 30.6, 26.4, 21.7, 16.4; **HRMS** (ESI$^+$) calc. for $C_{26}H_{27}O_3NClS_2$ $[M+H]^+$ 500.1115, found 500.1112.

(*Z*)-*N*-(6-((*tert*-Butyldimethylsilyl)oxy)-1-(2-(methylthio)phenyl)-1-oxohex-2-en-2-yl)-4-methyl-*N*-phenylbenzenesulfonamide, 4f

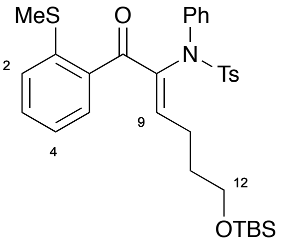

Prepared according to General Procedure B, using PNP(Cy) (6 mg, 0.015 mmol, 5 mol%), 2-(methylthio)benzaldehyde (40 µL, 0.300 mmol, 1.0 equiv.) and ynamide **2f** (146 mg, 0.330 mmol, 1.1 equiv.). The crude material was purified by column chromatography (petrol/EtOAc (95:5)) to give **4f** as a colourless oil (110 mg, 0.185 mmol, 63%); **R*f*** 0.41 (petrol/EtOAc (80:20)); **IR** (thin film, ν_{max}/cm^{-1}) 2953, 2927, 2856, 1665, 1597, 1491, 1434, 1353, 1250, 1160, 1090, 1004; **^{1}H NMR** (400 MHz, $CDCl_3$) δ_H 7.69-7.65 (3H, m, Ar*H*), 7.54 (2H, d, *J* = 8.5 Hz, Ar*H*), 7.44 (1H, td, *J* = 8.0 and 1.5 Hz, Ar*H*), 7.34 (1H, d, *J* = 8.0 Hz, Ar*H*), 7.27-7.20 (3H, m, Ar*H*), 7.16-7.12 (3H, m, Ar*H*), 6.40 (1H, t, *J* = 7.5 Hz, H9), 3.46 (2H, t, *J* = 6.5 Hz, H12), 2.44 (3H, s, SC*H*$_3$), 2.44-2.39 (2H, m, H10), 1.49-1.42 (2H, m, H11), 0.83 (9H, s, SiC(C*H*$_3$)$_3$), –0.03 (6H, s, Si(C*H*$_3$)$_2$); **^{13}C NMR** (101 MHz, $CDCl_3$) δ_C 194.5, 150.7, 143.6, 140.8, 140.7, 139.0, 137.4, 135.8, 131.2, 130.5, 129.1, 129.0, 128.8, 126.5, 126.3, 125.9, 124.4, 62.5, 30.7, 26.0, 25.5, 21.7, 18.3, 16.3, -5.3; **HRMS** (ESI$^+$) calc. for $C_{32}H_{42}O_4NS_2Si$ $[M+H]^+$ 596.2319, found 596.2320.

(*Z*)-4-Methyl-*N*-(3-(2-(methylthio)phenyl)-3-oxo-1-phenylprop-1-en-2-yl)-*N*-phenylbenzenesulfonamide, 4g

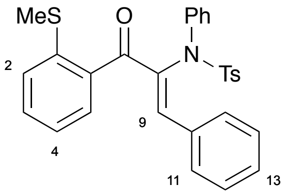

Prepared according to General Procedure B, using PNP(Cy) (6 mg, 0.015 mmol, 5 mol%), 2-(methylthio)benzaldehyde (40 μL, 0.300 mmol, 1.0 equiv.) and ynamide **2g** (115 mg, 0.330 mmol, 1.1 equiv.). The crude material was purified by column chromatography (petrol/EtOAc (90:10)) to give **4g** as a colourless oil (63 mg, 0.13 mmol, 42%); **R*f*** 0.18 (petrol/EtOAc (80:20)); **IR** (thin film, ν_{max}/cm^{-1}) 2922, 1657, 1597, 1447, 1324, 1268, 1122, 1088, 1007; **^{1}H NMR** (400 MHz, $CDCl_3$) δ_H 7.92 (1H, d, *J* = 7.5 Hz, Ar*H*), 7.76-7.73 (2H, m, Ar*H*), 7.70 (2H, d, *J* = 8.5 Hz, Ar*H*), 7.54-7.47 (3H, m, Ar*H*), 7.43 (1H, d, *J* = 8.0 Hz, Ar*H*), 7.37-7.32 (3H, m, Ar*H*), 7.28 (1H, t, *J* = 7.5 Hz, Ar*H*), 7.17 (2H, d, *J* = 8.5 Hz, Ar*H*), 7.13-7.10 (2H, m, Ar*H*), 7.06 (1H, s, H9), 7.04-7.00 (1H, m, Ar*H*), 2.54 (3H, s, SC*H*$_3$), 2.35 (3H, s, TsC*H*$_3$); **^{13}C NMR** (101 MHz, $CDCl_3$) δ_C 195.7, 143.9, 143.7, 139.8, 139.7, 138.4, 136.9, 135.3, 131.9, 131.6, 131.4, 130.5, 130.5, 129.0, 129.0, 128.7, 128.5, 126.2, 126.0, 125.1, 124.4, 21.7, 16.2; **HRMS** (ESI^+) calc. for $C_{29}H_{26}NO_3S_2$ [M+H]+ 500.1349, found 500.1346.

(*Z*)-*N*-(1-(2,4-*bis*(Methylthio)phenyl)-1-oxonon-2-en-2-yl)-4-methyl-*N*-phenylbenzenesulfonamide, 4m

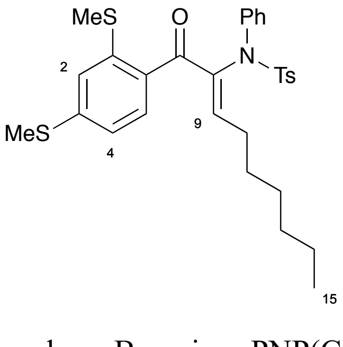

Prepared according to General Procedure B, using PNP(Cy) (6 mg, 0.015 mmol, 5 mol%), 2,4-*bis*(methythio)benzaldehyde (60 mg, 0.300 mmol, 1.0 equiv.) and ynamide **2a** (117 mg, 0.330 mmol, 1.1 equiv.). The crude material was purified by column chromatography (petrol/EtOAc (90:10)) to give **4m** as a yellow oil (103 mg, 0.186 mmol, 62%); **R*f*** 0.35 (petrol/EtOAc (80:20)); **IR** (thin film, ν_{max}/cm^{-1}) 2924, 2856, 1653, 1573, 1488, 1352, 1239, 1158, 1110; **^{1}H NMR** (400 MHz,

$CDCl_3$) δ_H 7.63 (1H, d, *J* = 8.0 Hz, Ar*H*), 7.56 (2H, d, *J* = 8.5 Hz, Ts*H*), 7.47 (2H, d, *J* = 8.5 Hz, Ar*H*), 7.15 (2H, t, *J* = 8.0 Hz, Ar*H*), 7.07-7.03 (4H, m, Ts*H* and Ar*H*), 6.95 (1H, dd, *J* = 8.0 and 2.0 Hz, Ar*H*), 6.20 (1H, t, *J* = 7.5 Hz, H9), 2.45 (3H, s, SC*H*₃), 2.37 (3H, s, SC*H*₃), 2.26-2.19 (5H, m, TsC*H*₃ and H10), 1.18-1.06 (8H, m, H11-14), 0.76 (3H, t, *J* = 7.0 Hz, H15); **^{13}C NMR** (101 MHz, $CDCl_3$) δ_C 193.8, 149.4, 143.9, 143.7, 141.0, 140.9, 140.2, 135.5, 132.8, 132.0, 129.0, 129.0, 128.7, 126.4, 125.8, 122.4, 120.5, 31.5, 29.1, 28.6, 27.7, 22.5, 21.6, 16.1, 15.1, 14.1; **HRMS** (ESI$^+$) calc. for $C_{30}H_{36}O_3NS_3$ [M+H]$^+$ 554.1854, found 554.1855.

(*Z*)-4-Methyl-*N*-(1-(2-(methylthio)cyclohex-1-en-1-yl)-1-oxonon-2-en-2-yl)-*N*-phenylbenzenesulfonamide, 4v

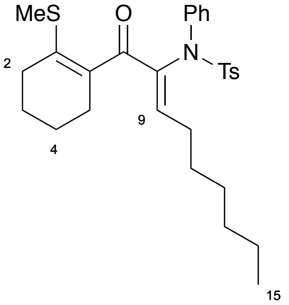

Prepared according to General Procedure B, using PNP(Cy) (6 mg, 0.015 mmol, 5 mol%), 2-(methylthio)cyclohex-1-ene-1-carbaldehyde (47 mg, 0.300 mmol, 1.0 equiv.) and ynamide **2a** (117 mg, 0.330 mmol, 1.1 equiv.). The crude material was purified by column chromatography (petrol/EtOAc (90:10)) to give **4v** as a yellow oil (94 mg, 0.184 mmol, 61%); **R*f*** 0.34 (petrol/EtOAc (80:20)); **IR** (thin film, ν_{max}/cm^{-1}) 2927, 2857, 1670, 1624, 1597, 1490, 1348, 1232, 1159, 1091; **^{1}H NMR** (400 MHz, $CDCl_3$) δ_H 7.62 (2H, d, *J* = 8.5 Hz, Ts*H*), 7.35 (2H, d, *J* = 8.5 Hz, Ar*H*), 7.14 (2H, tt, *J* = 8.5 and 1.5 Hz, Ar*H*), 7.08 (2H, d, *J* = 8.5 Hz, Ar*H*), 7.04 (1H, tt, *J* = 7.5 and 1.0 Hz, Ar*H*), 6.72 (1H, t, *J* = 7.5 Hz, H9), 2.37 (2H, q, *J* = 7.5 Hz, H10), 2.30-2.27 (7H, m, H2, 5 and TsC*H*₃), 2.06 (3H, s, SC*H*₃), 1.73-1.60 (4H, m, H3 and 4), 1.30-1.23 (2H, m, H11), 1.18-1.10 (6H, m, H12-14), 0.77 (3H, t, *J* = 7.0 Hz, H15); **^{13}C NMR** (101 MHz, $CDCl_3$) δ_C 196.8, 151.4, 143.5, 140.8, 139.1, 138.1, 136.4, 132.8, 129.0, 129.0, 128.8, 126.3, 125.8, 31.6, 29.2, 29.1, 29.0, 28.8, 27.9, 23.2, 22.6, 21.9, 21.7, 14.9, 14.1; **HRMS** (ESI$^+$) calc. for $C_{29}H_{38}O_3NS_2$ [M+H]$^+$ 512.2288, found 512.2289.

3.4 Suzuki-type coupling products

(*E*)-4-Methyl-*N*-(2-(4'-methyl-[1,1'-biphenyl]-2-carbonyl)oct-1-en-1-yl)-*N*-phenylbenzenesulfonamide, 5a

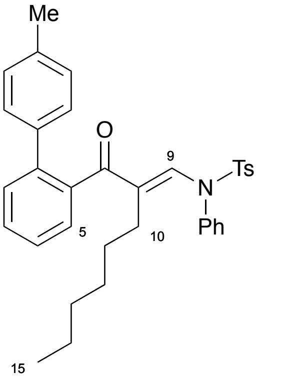

Prepared according to General Procedure C, using 2-(methylthio)benzaldehyde (40 μL, 0.300 mmol, 1.0 equiv.), ynamide **2a** (117 mg, 0.330 mmol, 1.1 equiv.), and *p*-tolylboronic acid (61 mg, 0.450 mmol, 1.5 equiv.). The crude material was purified by column chromatography (petrol/EtOAc (95:5)) to give **5a** as a colourless oil (123 mg, 0.223 mmol, 74%); $\mathbf{R}_f$ 0.25 (petrol/EtOAc (90:10)); **IR** (thin film, ν_{max}/cm^{-1}) 3060, 2926, 2857, 1646, 1613, 1518, 1366, 1287, 1172, 1125, 1088; **^{1}H NMR** (400 MHz, $CDCl_3$) δ_H 7.57 (1H, t, *J* = 7.5 Hz, Ar*H*), 7.48 (1H, t, *J* = 7.5 Hz, Ar*H*), 7.42 (1H, d, *J* = 7.5 Hz, Ar*H*), 7.37 (1H, d, *J* = 7.5 Hz, Ar*H*), 7.29-7.25 (3H, m, Ar*H*), 7.15-7.09 (4H, m, Ar*H*), 7.03 (1H, s, H9), 7.00 (2H, d, *J* = 8.0 Hz, Ar*H*), 6.87 (2H, d, *J* = 8.0 Hz, Ar*H*), 6.45 (2H, d, *J* = 8.0 Hz, Ar*H*), 2.52 (3H, s, TsCH_3), 2.38 (3H, s, PhCH_3), 1.48-1.45 (2H, m, H10), 1.16-1.07 (2H, m, H14), 0.98-0.91 (2H, m, H13), 0.81 (3H, t, *J* = 7.5 Hz, H15), 0.75-0.66 (4H, m, H11 and 12); **^{13}C NMR** (101 MHz, $CDCl_3$) δ_C 199.8, 144.8, 143.2, 140.0, 139.8, 138.0, 137.8, 137.0, 134.1, 129.8, 129.7, 129.5, 129.4, 129.2, 128.9$_4$, 128.8$_9$, 128.8, 128.6, 128.4, 128.1, 127.5, 31.6, 29.3, 27.1, 24.5, 22.6, 21.8, 21.3, 14.2; **HRMS** (ESI$^+$) calc. for $C_{35}H_{38}O_3NS$ [M+H]$^+$ 552.2567, found 552.2564.

(*E*)-*N*-(2-(4'-Methoxy-[1,1'-biphenyl]-2-carbonyl)oct-1-en-1-yl)-4-methyl-*N*-phenylbenzenesulfonamide, 5b

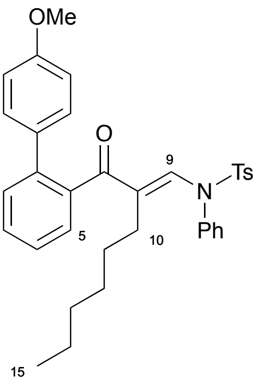

Prepared according to General Procedure C, using 2-(methylthio)benzaldehyde (40 μL, 0.300 mmol, 1.0 equiv.), ynamide **2a** (117 mg, 0.330 mmol, 1.1 equiv.), and 4-methoxyphenylboronic acid (68 mg, 0.450 mmol, 1.5 equiv.). The crude material was purified by column chromatography (petrol/EtOAc (90:10)) to give **5b** as a colourless oil (147 mg, 0.259 mmol, 86%); **R*f*** 0.40 (petrol/EtOAc (80:20)); **IR** (thin film, ν_{max}/cm^{-1}) 2955, 2929, 2856, 1645, 1611, 1517, 1366, 1287, 1248, 1172, 1125, 1088; **^{1}H NMR** (400 MHz, $CDCl_3$) δ_H 7.56 (1H, t, *J* = 7.5 Hz, Ar*H*), 7.47 (1H, t, *J* = 7.5 Hz, Ar*H*), 7.41 (1H, d, *J* = 7.5 Hz, Ar*H*), 7.37 (1H, d, *J* = 7.5 Hz, Ar*H*), 7.30-7.24 (3H, m, Ar*H*), 7.15 (2H, t, *J* = 7.5 Hz, Ar*H*), 7.10 (2H, d, *J* = 8.0 Hz, Ar*H*), 7.04 (1H, s, H9), 6.92 (2H, d, *J* = 8.0 Hz, Ar*H*), 6.71 (2H, d, *J* = 8.0 Hz, Ar*H*), 6.46 (2H, d, *J* = 8.0 Hz, Ar*H*), 3.81 (3H, s, OCH_3), 2.52 (3H, s, $TsCH_3$), 1.50-1.47 (2H, m, H10), 1.16-1.07 (2H, m, H14), 0.98-0.91 (2H, m, H13), 0.80 (3H, t, *J* = 7.5 Hz, H15), 0.74-0.68 (4H, m, H11 and 12); **^{13}C NMR** (101 MHz, $CDCl_3$) δ_C 199.9, 159.2, 144.8, 143.3, 139.7, 139.6, 138.0, 134.0, 133.1, 130.2, 129.8, 129.7, 129.5, 129.3, 128.9_3, 128.8_6, 128.6, 128.4, 128.1, 127.3, 113.9, 55.4, 31.7, 29.4, 27.2, 24.6, 22.6, 21.8, 14.2; **HRMS** (ESI^+) calc. for $C_{35}H_{38}O_4NS$ $[M+H]^+$ 568.2516, found 568.2512.

(*E*)-*N*-(2-(4'-Acetyl-[1,1'-biphenyl]-2-carbonyl)oct-1-en-1-yl)-4-methyl-*N*-phenylbenzenesulfonamide, 3c

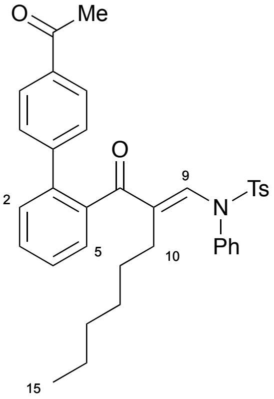

Prepared according to General Procedure C, using 2-(methylthio)benzaldehyde (40 μL, 0.300 mmol, 1.0 equiv.), ynamide **2a** (117 mg, 0.330 mmol, 1.1 equiv.), and 4-acetylphenylboronic acid (74 mg, 0.450 mmol, 1.5 equiv.). The crude material was purified by column chromatography (petrol/EtOAc (90:10)) to give **5c** as a colourless oil (105 mg, 0.181 mmol, 60%); **R*f*** 0.29 (petrol/EtOAc (80:20)); **IR** (thin film, ν_{max}/cm^{-1}) 3063, 2927, 2856, 1684, 1646, 1607, 1493, 1366, 1267, 1172, 1126, 1088; **^{1}H NMR** (500 MHz, $CDCl_3$) δ_H 7.81 (2H, d, *J* = 8.0 Hz, Ar*H*), 7.62 (1H, t, *J* = 7.5 Hz, Ar*H*), 7.55 (1H, t, *J* = 7.5 Hz, Ar*H*), 7.45-7.41 (2H, m, Ar*H*), 7.29-7.25 (3H, m, Ar*H*), 7.17 (2H, d, *J* = 8.0 Hz, Ar*H*), 7.13-7.09 (5H, m, Ar*H* and H9), 6.49 (2H, d, *J* = 8.0 Hz, Ar*H*), 2.60 (3H, s, COCH_3), 2.51 (3H, s, TsCH_3), 1.45-1.42 (2H, m, H10), 1.12-1.05 (2H, m, H14), 0.94-0.88 (2H, m, H13), 0.79 (3H, t, *J* = 7.5 Hz, H15), 0.70-0.61 (4H, m, H11 and 12); **^{13}C NMR** (126 MHz, $CDCl_3$) δ_C 199.2, 197.7, 145.5, 145.1, 143.8, 140.0, 139.0, 137.6, 135.9, 134.0, 130.0, 129.8, 129.5, 129.4$_2$, 129.3$_6$, 129.2, 129.1, 128.7, 128.6, 128.4, 128.1, 127.9, 31.7, 29.3, 27.3, 26.8, 24.3, 22.6, 21.9, 14.2; **HRMS** (ESI$^+$) calc. for $C_{36}H_{38}O_4NS$ [M+H]$^+$ 580.2516, found 580.2511.

3.5 Isoxazole products

4-Hexyl-5-(2-(methylthio)phenyl)isoxazole, 6a

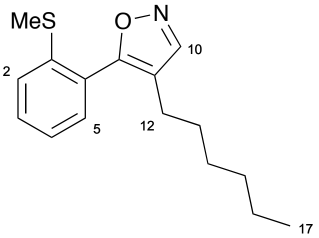

Prepared according to General Procedure D, using 2-(methylthio)benzaldehyde (40 μL, 0.300 mmol, 1.0 equiv.) and ynamide **2a** (117 mg, 0.330 mmol, 1.1 equiv.). The crude material was purified by column chromatography (petrol/EtOAc (98:2)) to give **6a** as a pale green oil (70 mg, 0.254 mmol, 85%); **R*f*** 0.38 (petrol/EtOAc (90:10)); **IR** (thin film, ν_{max}/cm^{-1}) 2925, 2855, 1738, 1452, 1377, 1260, 1143, 1041; **^{1}H NMR** (400 MHz, $CDCl_3$) δ_H 8.23 (1H, s, H10), 7.44 (1H, ddd, *J* = 8.0, 7.5 and 2.0 Hz, H4), 7.33 (1H, d, *J* = 8.0 Hz, H5), 7.28 (1H, dd, *J* = 7.5 and 2.0 Hz, H2), 7.23 (1H, ddd, *J* = 7.5, 7.5 and 1.0 Hz, H3), 2.42 (3H, s, SCH_3), 2.39 (2H, t, *J* = 7.5 Hz, H12), 1.51 (2H, quin., *J* = 7.5 Hz, H13), 1.30-1.17 (6H, m, H14-16), 0.84 (3H, t, *J* = 7.0 Hz, H17); **^{13}C NMR** (101 MHz, $CDCl_3$) δ_C 164.4, 151.4, 139.9, 130.7, 130.6, 127.0, 126.1, 124.8, 117.4, 31.6, 29.8, 28.9, 22.9, 22.7, 16.1, 14.2; **HRMS** (ESI$^+$) calc. for $C_{16}H_{22}NOS$ [M+H]+ 276.1417, found 276.1417.

4-(3-Chloropropyl)-5-(2-(methylthio)phenyl)isoxazole, 6e

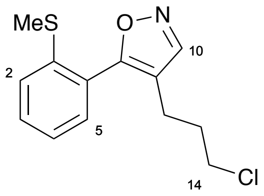

Prepared according to General Procedure D, using 2-(methylthio)benzaldehyde (40 μL, 0.300 mmol, 1.0 equiv.) and ynamide **2e** (115 mg, 0.330 mmol, 1.1 equiv.). The crude material was purified by column chromatography (petrol/EtOAc (95:5)) to give **6e** as a yellow oil (45 mg, 0.168 mmol, 56%); **R*f*** 0.17 (petrol/EtOAc (90:10)); **IR** (thin film, ν_{max}/cm^{-1}) 2957, 2922, 1630, 1584, 1486, 1451, 1434, 929; **^{1}H NMR** (400 MHz, $CDCl_3$) δ_H 8.26 (1H, s, H10), 7.46 (1H, ddd, *J* = 8.0, 7.5 and 1.5 Hz, Ar*H*), 7.34 (1H, d, *J* = 8.0 Hz, Ar*H*), 7.29 (1H, dd, *J* = 7.5 and 1.5 Hz, Ar*H*), 7.24 (1H, td, *J* = 7.5 and 1.0 Hz, Ar*H*), 3.49 (2H, t, *J* = 6.5 Hz, H14), 2.60 (2H, t, *J* = 7.5 Hz, H12), 2.43 (3H, s, SCH_3),

1.97 (2H, tt, J = 7.5 and 6.5 Hz, H13); ^{13}**C NMR** (101 MHz, $CDCl_3$) δ_C 165.1, 151.3, 139.8, 130.8, 130.6, 126.6, 126.1, 124.9, 115.5, 44.0, 32.3, 20.2, 16.0; **HRMS** (ESI^+) calc. for $C_{13}H_{15}ONClS$ $[M+H]^+$ 268.0557, found 268.0558.

3-(5-(2-(Methylthio)phenyl)isoxazol-4-yl)propan-1-ol, 6f

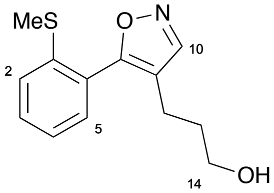

Prepared according to General Procedure D, using 2-(methylthio)benzaldehyde (40 μL, 0.300 mmol, 1.0 equiv.) and ynamide **2f** (146 mg, 0.330 mmol, 1.1 equiv.). The crude material was purified by column chromatography (toluene/EtOAc (90:10)) to give **6f** as a colourless oil (59 mg, 0.237 mmol, 79%); $\mathbf{R}_f$ 0.13 (toluene/EtOAc (90:10)); **IR** (thin film, ν_{max}/cm^{-1}) 3389 (br), 2922, 2867, 1584, 1487, 1451, 1434, 1043; ^{1}**H NMR** (500 MHz, $CDCl_3$) δ_H 8.26 (1H, s, H10), 7.45 (1H, td, J = 8.0 and 1.5 Hz, Ar*H*), 7.34 (1H, d, J = 8.0 Hz, Ar*H*), 7.29 (1H, dd, J = 7.5 and 1.5 Hz, Ar*H*), 7.23 (1H, td, J = 7.5 and 1.0 Hz, Ar*H*), 3.61 (2H, t, J = 6.5 Hz, H14), 2.52 (2H, t, J = 7.5 Hz, H12), 2.43 (3H, s, SCH_3), 1.82-1.76 (3H, m, H13 and O*H*); ^{13}**C NMR** (126 MHz, $CDCl_3$) δ_C 164.6, 151.3, 139.7, 130.7, 130.6, 126.7, 126.1, 124.9, 116.6, 61.7, 32.3, 19.2, 16.0; **HRMS** (ESI^+) calc. for $C_{13}H_{16}O_2NS$ $[M+H]^+$ 250.0896, found 250.0897.

5-(2-(Methylthio)phenyl)-4-phenylisoxazole, 6g

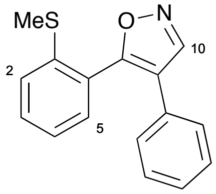

Method A: Prepared according to General Procedure D, using 2-(methylthio)benzaldehyde (40 μL, 0.300 mmol, 1.0 equiv.) and ynamide **2g** (115 mg, 0.330 mmol, 1.1 equiv.). The crude material was purified by column chromatography (petrol/EtOAc (98:2)) to give **6g** as a colourless oil (60 mg, 0.224 mmol, 75%).

Method B (4 mmol scale reaction): Prepared according to General Procedure D, using 2-(methylthio)benzaldehyde (609 mg, 4.00 mmol, 1.0 equiv.) and ynamide **2g** (1.53 g, 4.40 mmol,

1.1 equiv.). The crude material was purified by column chromatography (petrol/EtOAc (98:2)) to give **6g** as a colourless oil (652 mg, 2.44 mmol, 61%).

R_f 0.23 (petrol/EtOAc (90:10)); **IR** (thin film, ν_{max}/cm^{-1}) 3058, 2921, 1601, 1562, 1433, 1377, 1156; **^{1}H NMR** (400 MHz, $CDCl_3$) δ_H 8.58 (1H, s, H10), 7.48 (1H, ddd, *J* = 8.0, 7.5 and 1.5 Hz, H4), 7.37 (1H, dd, *J* = 8.0 and 1.0 Hz, H5), 7.34 (1H, dd, *J* = 7.5 and 1.5 Hz, H2), 7.32-7.21 (6H, m, H3 and Ph*H*), 2.37 (3H, s, SC*H*$_3$); **^{13}C NMR** (101 MHz, $CDCl_3$) δ_C 163.9, 150.1, 139.9, 130.9, 130.8, 129.5, 128.8, 127.7, 127.0 (2C), 126.7, 125.1, 118.3, 16.2; **HRMS** (ESI$^+$) calc. for $C_{16}H_{14}ONS$ [M+H]$^+$ 268.0791, found 268.0790.

5-(2-(Dimethylsulfonio)phenyl)-2-methyl-4-phenylisoxazol-2-ium *bis*-tetrafluoroborate, S1

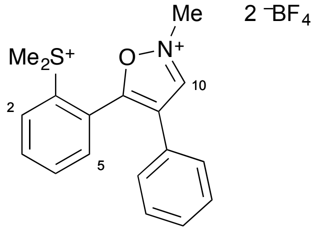

To a stirred suspension of trimethyloxonium tetrafluoroborate (415 mg, 2.81 mmol, 2.5 equiv.) in CH_2Cl_2 (60 mL) was added a solution of isoxazole **6g** (300 mg, 1.12 mmol, 1.0 equiv.) in CH_2Cl_2 (6.0 mL) dropwise and the suspension stirred for 16 h at room temperature. The solvent was removed *in vacuo* and the resulting residue was crystallised with Et_2O (10 mL) to give **S1** as a colourless crystalline solid (471 mg, 1.00 mmol, 89%); **m.p.** 179-183 °C (Et_2O); **IR** (thin film, ν_{max}/cm^{-1}) 3129, 3039, 2943, 1592, 1555, 1448, 1427, 1096, 1028, 1000; **^{1}H NMR** (400 MHz, DMSO-d$_6$) δ_H 10.31 (1H, s, H10), 8.59 (1H, dd, *J* = 8.0 and 1.0 Hz, H2), 8.18 (1H, td, *J* = 8.0 and 1.5 Hz, H3), 8.12 (1H, td, *J* = 7.5 and 1.0 Hz, H4) 8.01 (1H, dd, *J* = 7.5 and 1.5 Hz, H5), 7.59-7.57 (3H, m, Ph*H*), 7.42-7.39 (2H, m, Ph*H*), 4.58 (3H, s, NC*H*$_3$), 2.94 (6H, s, S(C*H*$_3$)$_2$); **^{13}C NMR** (101 MHz, DMSO-d$_6$) δ_C 162.6, 149.7, 135.7, 135.2, 132.8, 131.4, 130.8, 130.2, 128.7, 126.3, 125.7, 124.7, 122.9, 41.9, 29.0; **^{19}F NMR** (376 MHz, DMSO-d$_6$) δ_F –148.2; **HRMS** (ESI$^+$) calc. for $C_{18}H_{19}ONS$ [M–2BF$_4$]$^{2+}$ 148.5588, found 148.5589.

4-(4-Methoxyphenyl)-5-(2-(methylthio)phenyl)isoxazole, 6h

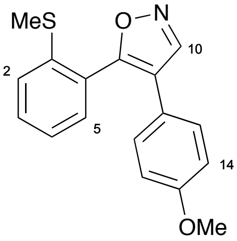

Prepared according to General Procedure D, using 2-(methylthio)benzaldehyde (40 μL, 0.300 mmol, 1.0 equiv.) and ynamide **2h** (125 mg, 0.330 mmol, 1.1 equiv.). The crude material was purified by column chromatography (petrol/EtOAc (90:10)) to give **6h** as a yellow oil (60 mg, 0.202 mmol, 67%); **R*f*** 0.30 (petrol/EtOAc (80:20)); **IR** (thin film, ν_{max}/cm^{-1}) 3062, 3006, 2959, 2921, 2836, 1608, 1516, 1452, 1433, 1249; **^{1}H NMR** (500 MHz, $CDCl_3$) δ_H 8.53 (1H, s, H10), 7.46 (1H, ddd, *J* = 8.0, 7.5 and 1.5 Hz, Ar*H*), 7.36 (1H, d, *J* = 8.0 Hz, Ar*H*), 7.33 (1H, dd, *J* = 7.5 and 1.5 Hz, Ar*H*), 7.22 (1H, td, *J* = 7.5 and 1.0 Hz, Ar*H*), 7.17 (2H, d, *J* = 9.0 Hz, H13), 6.82 (2H, d, *J* = 9.0 Hz, H14), 3.77 (3H, s, OC*H*$_3$), 2.36 (3H, s, SC*H*$_3$); **^{13}C NMR** (126 MHz, $CDCl_3$) δ_C 163.1, 159.2, 150.1, 140.0, 130.9, 130.8, 128.3, 127.3, 126.6, 125.1, 121.9, 118.0, 114.4, 55.3, 16.2; **HRMS** (ESI$^+$) calc. for $C_{17}H_{16}O_2NS$ $[M+H]^+$ 298.0896, found 298.0896.

5-(2-(Methylthio)phenyl)-4-(4-(trifluoromethyl)phenyl)isoxazole, 6i

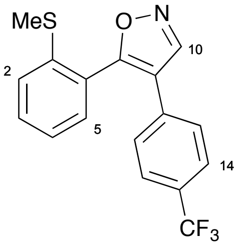

Prepared according to General Procedure D, using 2-(methylthio)benzaldehyde (40 μL, 0.300 mmol, 1.0 equiv.) and ynamide **2i** (137 mg, 0.330 mmol, 1.1 equiv.). The crude material was purified by column chromatography (petrol/EtOAc (98:2)) to give **6i** as a yellow oil (73 mg, 0.218 mmol, 73%); **R*f*** 0.27 (petrol/EtOAc (90:10)); **IR** (thin film, ν_{max}/cm^{-1}) 3064, 2924, 2849, 1625, 1453, 1434, 1417 1323, 1160; **^{1}H NMR** (500 MHz, $CDCl_3$) δ_H 8.62 (1H, s, H10), 7.56 (2H, d, *J* = 8.0 Hz, H14), 7.51 (1H, ddd, *J* = 8.0, 7.5 and 1.5 Hz, Ar*H*), 7.39-7.33 (4H, m, Ar*H* and H13), 7.26 (1H, td, *J* = 7.5 and 1.0 Hz, Ar*H*), 2.38 (3H, s, SC*H*$_3$); **^{13}C NMR** (126 MHz, $CDCl_3$) δ_C 164.9, 149.8, 139.9, 133.3, 131.4, 130.7, 130.1, 129.6 (q, *J* = 32.5 Hz), 127.3, 126.6, 125.9 (q, *J* = 4.0 Hz), 125.3, 124.1 (q, *J* =

272.0 Hz), 117.2, 16.1; **^{19}F NMR** (470 MHz, $CDCl_3$) δ_F –62.6; **HRMS** (ESI^+) calc. for $C_{17}H_{13}ONF_3S$ $[M+H]^+$ 336.0665, found 336.0663.

4-(3,5-Dimethoxyphenyl)-5-(2-(methylthio)phenyl)isoxazole, 6j

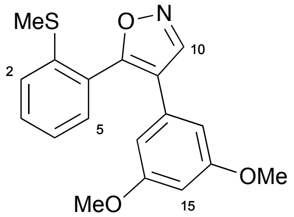

Prepared according to General Procedure D, using 2-(methylthio)benzaldehyde (40 μL, 0.300 mmol, 1.0 equiv.) and ynamide **2j** (135 mg, 0.330 mmol, 1.1 equiv.). The crude material was purified by column chromatography (petrol/Et_2O (80:20)) to give **6j** as an orange oil (68 mg, 0.208 mmol, 69%); **R*f*** 0.18 (petrol/EtOAc (80:20)); **IR** (thin film, ν_{max}/cm^{-1}) 2935, 2841, 1592, 1454, 1424, 1380, 1287, 1204, 1154; **^{1}H NMR** (400 MHz, $CDCl_3$) δ_H 8.58 (1H, s, H10), 7.48 (1H, t, *J* = 7.5 Hz, Ar*H*), 7.39-7.35 (2H, m, Ar*H*), 7.28-7.23 (1H, m, Ar*H*), 6.42 (2H, s, Ar*H*), 6.37 (1H, s, Ar*H*), 3.66 (6H, s, OC*H*3), 2.39 (3H, s, SC*H*3); **^{13}C NMR** (101 MHz, $CDCl_3$) δ_C 164.2, 161.0, 150.0, 140.1, 131.2, 131.0, 130.9, 127.2, 126.6, 125.2, 118.3, 105.2, 99.8, 55.3, 16.3; **HRMS** (ESI^+) calc. for $C_{18}H_{18}O_3NS$ $[M+H]^+$ 328.1002, found 328.1002.

5-(2-(Methylthio)phenyl)-4-(thiophen-3-yl)isoxazole, 6k

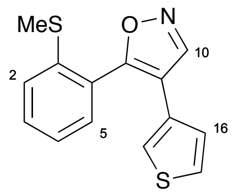

Prepared according to General Procedure D, using 2-(methylthio)benzaldehyde (40 μL, 0.300 mmol, 1.0 equiv.) and ynamide **2k** (117 mg, 0.330 mmol, 1.1 equiv.). The crude material was purified by column chromatography (petrol/EtOAc (98:2)) to give **6k** as a yellow oil (58 mg, 0.212 mmol, 71%); **R*f*** 0.19 (petrol/EtOAc (90:10)); **IR** (thin film, ν_{max}/cm^{-1}) 3101, 2920, 2849, 1663, 1633, 1586, 1452, 1432, 1150; **^{1}H NMR** (400 MHz, $CDCl_3$) δ_H 8.56 (1H, s, H10), 7.50 (1H, ddd, *J* = 8.0, 7.5 and 1.5 Hz, Ar*H*), 7.39 (1H, d, *J* = 8.0 Hz, Ar*H*), 7.36 (1H, dd, *J* = 7.5 and 1.5 Hz, Ar*H*), 7.28-7.24 (2H, m, Ar*H* and H16), 7.14 (1H, dd, *J* = 3.0 and 1.5 Hz, H13), 6.94 (1H, dd, *J* = 5.0 and 1.5 Hz, H15), 2.39 (3H, s, SC*H*3); **^{13}C NMR** (101 MHz, $CDCl_3$) δ_C 163.5, 150.0, 140.2, 131.1, 130.9, 129.7, 127.1,

126.7, 126.4, 126.3, 125.2, 121.6, 114.2, 16.2; **HRMS** (ESI^+) calc. for $C_{14}H_{12}ONS_2$ $[M+H]^+$ 274.0355, found 274.0355.

4-(Cyclohex-1-en-1-yl)-5-(2-(methylthio)phenyl)isoxazole, 6l

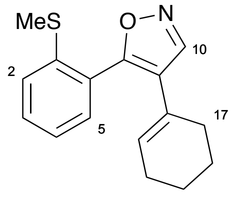

Prepared according to General Procedure D, using 2-(methylthio)benzaldehyde (40 μL, 0.300 mmol, 1.0 equiv.) and ynamide **2l** (116 mg, 0.330 mmol, 1.1 equiv.). The crude material was purified by column chromatography (petrol/EtOAc (98:2)) to give **6l** as a yellow oil (43 mg, 0.158 mmol, 53%); $\mathbf{R_f}$ 0.36 (petrol/EtOAc (90:10)); **IR** (thin film, ν_{max}/cm^{-1}) 2924, 2857, 2833, 1607, 1581, 1451, 1433, 1150; ^{1}**H NMR** (500 MHz, $CDCl_3$) δ_H 8.32 (1H, s, H10), 7.44 (1H, td, J = 7.5 and 1.5 Hz, Ar*H*), 7.34-7.31 (2H, m, Ar*H*), 7.22 (1H, td, J = 7.5 and 1.0 Hz, Ar*H*), 5.85-5.82 (1H, m, H13), 2.42 (3H, s, SC*H*$_3$), 2.09-2.05 (2H, m, H14), 2.03-1.99 (2H, m, H17), 1.63-1.54 (4H, m, H15 and 16); ^{13}**C NMR** (126 MHz, $CDCl_3$) δ_C 162.3, 149.5, 140.0, 130.9, 130.6, 128.2, 126.8, 126.6, 126.3, 124.9, 120.3, 27.4, 25.7, 22.7, 21.9, 16.3; **HRMS** (ESI^+) calc. for $C_{16}H_{18}ONS$ $[M+H]^+$ 272.1104, found 272.1103.

4-Hexyl-5-(4-methoxy-2-(methylthio)phenyl)isoxazole, 6n

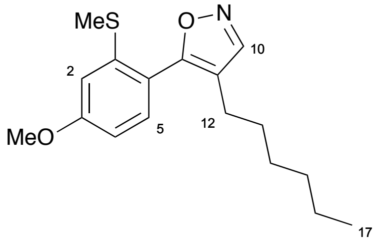

Prepared according to General Procedure D, using 4-methoxy-2-(methylthio)benzaldehyde (55 mg, 0.300 mmol, 1.0 equiv.) and ynamide **2a** (117 mg, 0.330 mmol, 1.1 equiv.). The crude material was purified by column chromatography (petrol/EtOAc (98:2)) to give **6n** as a yellow oil (73 mg, 0.240 mmol, 80%); $\mathbf{R_f}$ 0.27 (petrol/EtOAc (90:10)); **IR** (thin film, ν_{max}/cm^{-1}) 2954, 2925, 2856, 1592, 1562, 1456, 1433, 1292, 1225, ; ^{1}**H NMR** (400 MHz, $CDCl_3$) δ_H 8.22 (1H, s, H10), 7.21 (1H, d, J = 8.5 Hz, H5), 6.85 (1H, d, J = 2.5 Hz, H2), 6.75 (1H, dd, J = 8.5 and 2.5 Hz, H4), 3.87 (3H, s, OC*H*$_3$), 2.41 (3H, s, SC*H*$_3$), 2.38 (2H, t, J = 8.0 Hz, H12), 1.56-1.48 (2H, m, H13), 1.31-1.20 (6H, m,

H14-16), 0.85 (3H, t, *J* = 7.0 Hz, H17); **^{13}C NMR** (101 MHz, $CDCl_3$) δ_C 164.4, 161.2, 151.4, 141.4, 131.8, 119.1, 117.1, 112.2, 109.6, 55.5, 31.5, 29.7, 28.9, 22.9, 22.6, 15.9, 14.1; **HRMS** (ESI^+) calc. for $C_{17}H_{24}O_2NS$ $[M+H]^+$ 306.1522, found 306.1522.

4-Hexyl-5-(2-(methylthio)-4-(trifluoromethyl)phenyl)isoxazole, 6o

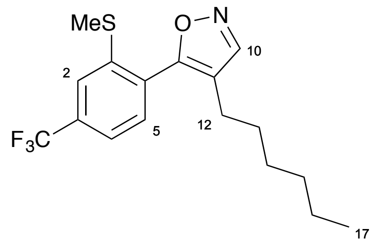

Prepared according to General Procedure D, using 2-(methylthio)-4-(trifluoromethyl)benzaldehyde (66 mg, 0.300 mmol, 1.0 equiv.) and ynamide **2a** (117 mg, 0.330 mmol, 1.1 equiv.). The crude material was purified by column chromatography (petrol/EtOAc (99:1)) to give **6o** as a yellow oil (71 mg, 0.207 mmol, 69%); **R_f** 0.46 (petrol/EtOAc (90:10)); **IR** (thin film, ν_{max}/cm^{-1}) 2928, 2858, 1457, 1397, 1170, 1127, 1082; **^{1}H NMR** (500 MHz, $CDCl_3$) δ_H 8.25 (1H, s, H10), 7.51 (1H, s, Ar*H*), 7.46 (1H, d, *J* = 8.0 Hz, Ar*H*), 7.39 (1H, d, *J* = 8.0 Hz, Ar*H*), 2.47 (3H, s, SCH_3), 2.39 (2H, t, *J* = 8.0 Hz, H12), 1.54-1.48 (2H, m, H13), 1.28-1.16 (6H, m, H14-16), 0.83 (3H, t, *J* = 7.5 Hz, H17); **^{13}C NMR** (126 MHz, $CDCl_3$) δ_C 162.8, 151.5, 141.8, 132.7 (q, *J* = 32.5 Hz), 131.0, 130.0, 123.8 (q, *J* = 272.0 Hz), 122.1 (q, *J* = 4.0 Hz), 121.3 (q, *J* = 4.0 Hz), 118.2, 31.5, 29.7, 28.9, 22.8, 22.6, 15.7, 14.1; **^{19}F NMR** (470 MHz, $CDCl_3$) δ_F –63.1; **HRMS** (ESI^+) calc. for $C_{17}H_{21}ONF_3S$ $[M+H]^+$ 344.1291, found 344.1291.

5-(4-Bromo-2-(methylthio)phenyl)-4-hexylisoxazole, 6p

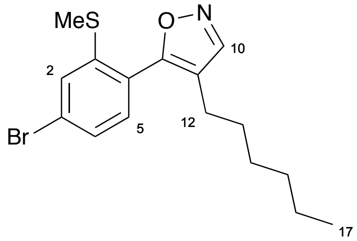

Prepared according to General Procedure D, using 4-bromo-2-(methylthio)benzaldehyde (69 mg, 0.300 mmol, 1.0 equiv.) and ynamide **2a** (117 mg, 0.330 mmol, 1.1 equiv.). The crude material was purified by column chromatography (petrol/Et_2O (98:2)) to give **6p** as a colourless oil (83 mg,

0.234 mmol, 79%); **R$_f$** 0.25 (petrol/Et$_2$O (95:5)); **IR** (thin film, ν_{max}/cm^{-1}) 2954, 2925, 2856, 1572, 1448, 1371, 1255, 1132, 1085; **^{1}H NMR** (400 MHz, CDCl$_3$) δ_H 8.22 (1H, s, H10), 7.40 (1H, s, H2), 7.34 (1H, d, J = 8.0 Hz, H4), 7.12 (1H, d, J = 8.0 Hz, H5), 2.42 (3H, s, SCH_3), 2.36 (2H, t, J = 7.5 Hz, H12), 1.51-1.46 (2H, m, H13), 1.28-1.17 (6H, m, H14-16), 0.83 (3H, t, J = 7.0 Hz, H17); **^{13}C NMR** (101 MHz, CDCl$_3$) δ_C 163.2, 151.5, 142.4, 131.8, 128.2, 127.7, 125.4, 125.1, 117.8, 31.5, 29.7, 28.9, 22.8, 22.6, 15.9, 14.1; **HRMS** (ESI$^+$) calc. for $C_{16}H_{21}ON^{79}BrS$ [M+H]$^+$ 354.0522, found 354.0523.

4-Hexyl-5-(3-(methylthio)thiophen-2-yl)isoxazole, 6q

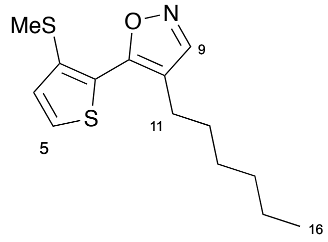

Prepared according to General Procedure D, using 3-(methylthio)thiophene-2-carbaldehyde (48 mg, 0.300 mmol, 1.0 equiv.) and ynamide **2a** (117 mg, 0.330 mmol, 1.1 equiv.). The crude material was purified by column chromatography (petrol/EtOAc (99:1)) to give **6q** as a yellow oil (48 mg, 0.171 mmol, 57%); **R$_f$** 0.38 (petrol/EtOAc (90:10)); **IR** (thin film, ν_{max}/cm^{-1}) 3105, 2954, 2924, 2856, 1609, 1495, 1467, 1347, 116, 1129; **^{1}H NMR** (500 MHz, CDCl$_3$) δ_H 8.19 (1H, s, H9), 7.50 (1H, d, J = 5.5 Hz, ArH), 7.09 (1H, d, J = 5.5 Hz, ArH), 2.55 (2H, t, J = 8.0 Hz, H11), 2.47 (3H, s, SCH_3), 1.61-1.55 (2H, m, H12), 1.36-1.24 (6H, m, H13-15), 0.87 (3H, t, J = 7.0 Hz, H16); **^{13}C NMR** (126 MHz, CDCl$_3$) δ_C 159.0, 151.8, 136.3, 128.6, 127.9, 122.4, 117.4, 31.6, 29.8, 29.1, 23.3, 22.7, 18.1, 14.2; **HRMS** (ESI$^+$) calc. for $C_{14}H_{20}ONS_2$ [M+H]$^+$ 282.0981, found 282.0981.

5-(1-(Ethylthio)octan-2-yl)-4-hexylisoxazole, 6t

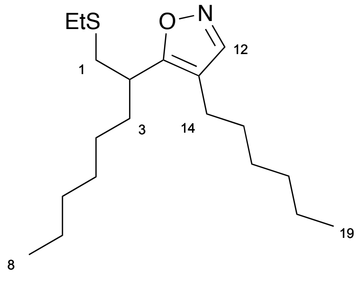

Prepared according to General Procedure D, using 2-((ethylthio)methyl)octanal (61 mg, 0.300 mmol, 1.0 equiv.) and ynamide **2a** (117 mg, 0.330 mmol, 1.1 equiv.). The crude material was purified by column chromatography (petrol/CH_2Cl_2/EtOAc (50:48:2)) to give **6t** as a colourless oil (67 mg, 0.204 mmol, 68%); **R*f*** 0.28 (petrol/CH_2Cl_2/EtOAc (50:48:2)); **IR** (thin film, ν_{max}/cm^{-1}) 2956, 2926, 2857, 1468, 1458, 1377, 1264; **^{1}H NMR** (400 MHz, $CDCl_3$) δ_H 8.05 (1H, s, H12), 3.04-2.96 (1H, m, H2), 2.82 (2H, d, *J* = 8.0 Hz, H1), 2.43-2.34 (4H, m, H14 and SC*H*$_2$CH$_3$), 1.80-1.71 (2H, m, H3), 1.59-1.48 (2H, m, H15), 1.38-1.08 (17H, m, H4-7, H16-18, and SCH$_2$C*H*$_3$), 0.90-0.83 (6H, m, H8 and H19); **^{13}C NMR** (101 MHz, $CDCl_3$) δ_C 168.4, 151.2, 115.9, 38.1, 36.0, 33.6, 31.8, 31.7, 30.4, 29.2, 29.1, 27.5, 26.7, 22.7 (2C), 22.5, 14.8, 14.2 (2C); **HRMS** (ESI$^+$) calc. for $C_{19}H_{36}ONS$ [M+H]$^+$ 326.2512, found 326.2512.

5-(1-(Ethylthio)-3-phenylpropan-2-yl)-4-hexylisoxazole, 6u

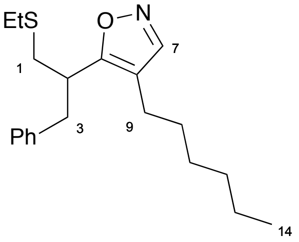

Prepared according to General Procedure D, using 2-benzyl-3-(ethylthio)propanal (62 mg, 0.300 mmol, 1.0 equiv.) and ynamide **2a** (117 mg, 0.330 mmol, 1.1 equiv.). The crude material was purified by column chromatography (petrol/Et_2O (98:2)) to give **6u** as a colourless oil (48 mg, 0.145 mmol, 48%); **R*f*** 0.27 (petrol/Et_2O (95:5)); **IR** (thin film, ν_{max}/cm^{-1}) 2956, 2927, 2857, 1495, 1472, 1454, 1265, 1073; **^{1}H NMR** (400 MHz, $CDCl_3$) δ_H 7.95 (1H, s, H7), 7.20-7.12 (3H, m, Ar*H*), 6.97 (2H, d, *J* = 7.5 Hz, Ar*H*), 3.25-3.11 (2H, m, H2 and 3), 2.99-2.88 (3H, m, H1 and 3), 2.46-2.37 (2H, m, H9), 2.08-1.92 (2H, m, SC*H*$_2$CH$_3$), 1.27-1.04 (11H, m, H10-13 and SCH$_2$C*H*$_3$), 0.86 (3H, t, *J* = 7.0 Hz, H14); **^{13}C NMR** (101 MHz, $CDCl_3$) δ_C 167.1, 151.1, 139.0, 128.9, 128.4, 126.5, 116.5, 40.7, 40.0, 35.4, 31.6, 29.9, 28.9, 26.7, 22.6, 22.0, 14.8, 14.2; **HRMS** (ESI$^+$) calc. for $C_{20}H_{30}ONS$ [M+H]$^+$ 332.2043, found 332.2042.

3.6 Pyrazole products

1-Cyclohexyl-4-hexyl-5-(2-(methylthio)phenyl)-1*H*-pyrazole, 7a

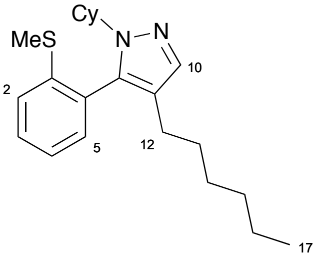

To a vial containing β-enaminone **3a** (100 mg, 0.197 mmol, 1.0 equiv.) and cyclohexylhydrazine hydrochloride (148 mg, 0.985 mmol, 5.0 equiv.) was added ethanol (0.39 mL), and the reaction mixture stirred for 16 h at 80 °C. After reaction completion, water (20 mL) and CH_2Cl_2 (20 mL) were added, and the aqueous phase extracted with CH_2Cl_2 (2 x 20 mL). The combined organic extracts were dried over magnesium sulfate, filtered, and concentrated *in* vacuo. The crude material was purified by column chromatography (petrol/EtOAc (98:2)) to give **7a** as a colourless oil (47 mg, 0.132 mmol, 67%); $\mathbf{R}_f$ 0.40 (petrol/EtOAc (90:10)); **IR** (thin film, ν_{max}/cm^{-1}) 2925, 2854, 1488, 1450, 1404, 1359, 1325, 988; **^{1}H NMR** (400 MHz, $CDCl_3$) δ_H 7.45 (1H, s, H10), 7.42 (1H, ddd, *J* = 8.0, 7.5 and 1.5 Hz, Ar*H*), 7.26 (1H, d, *J* = 8.0 Hz, Ar*H*), 7.19 (1H, td, *J* = 7.5 and 1.0 Hz, Ar*H*), 7.09 (1H, dd, *J* = 7.5 and 6.0 Hz, Ar*H*), 3.60-3.53 (1H, m, Cy*H*), 2.38 (3H, s, SCH_3), 2.25 (1H, ddd, *J* = 15.0, 8.5 and 6.5 Hz, H12), 2.17 (1H, ddd, *J* = 15.0, 8.5 and 7.0 Hz, H12), 2.06-1.95 (2H, m, Cy*H*), 1.89-1.72 (4H, m, Cy*H*), 1.62-1.56 (1H, m, Cy*H*), 1.47-1.38 (2H, m, H13), 1.25-1.10 (9H, m, Cy*H* and H14-16), 0.82 (3H, t, *J* = 7.0 Hz, H17); **^{13}C NMR** (101 MHz, $CDCl_3$) δ_C 140.8, 137.9, 137.4, 131.2, 129.6, 129.1, 124.4, 124.3, 120.2, 58.1, 33.9, 32.6, 31.7, 30.5, 29.1, 25.8, 25.7, 25.4, 23.9, 22.7, 15.1, 14.2; **HRMS** (ESI^+) calc. for $C_{22}H_{33}N_2S$ $[M+H]^+$ 357.2359, found 357.2357.

1-(*tert*-Butyl)-4-hexyl-5-(2-(methylthio)phenyl)-1*H*-pyrazole, 7b

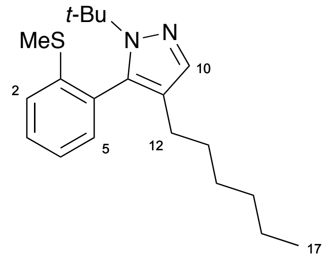

To a vial containing β-enaminone **3a** (100 mg, 0.197 mmol, 1.0 equiv.) and *tert*-butylhydrazine hydrochloride (123 mg, 0.985 mmol, 5.0 equiv.) was added ethanol (0.39 mL), and the reaction mixture stirred for 16 h at 80 °C. After reaction completion, water (20 mL) and CH_2Cl_2 (20 mL) were added, and the aqueous phase extracted with CH_2Cl_2 (2 x 20 mL). The combined organic extracts were dried over magnesium sulfate, filtered, and concentrated *in* vacuo. The crude material was purified by column chromatography (petrol/EtOAc (98:2)) to give **7b** as a colourless oil (44 mg, 0.133 mmol, 68%); **R*f*** 0.45 (petrol/EtOAc (90:10)); **IR** (thin film, ν_{max}/cm^{-1}) 2957, 2925, 2854, 1433, 1365, 1262, 1080; **^{1}H NMR** (400 MHz, $CDCl_3$) δ_H 7.43 (1H, s, H10), 7.41-7.37 (1H, m, Ar*H*), 7.21 (1H, d, *J* = 8.0 Hz, Ar*H*), 7.18-7.15 (2H, m, Ar*H*), 2.40 (3H, s, SCH_3), 2.15 (1H, ddd, *J* = 14.5, 9.5 and 6.5 Hz, H12), 1.99 (1H, ddd, *J* = 14.5, 9.0 and 6.5 Hz, H12), 1.49-1.37 (11H, m, H13 and $C(CH_3)_3$), 1.29-1.15 (6H, m, H14-16), 0.83 (3H, t, *J* = 7.0 Hz, H17); **^{13}C NMR** (101 MHz, $CDCl_3$) δ_C 141.0, 137.2, 136.5, 131.8, 131.8, 129.3, 123.8, 123.8, 122.3, 61.1, 31.7, 30.6, 30.2, 29.2, 23.7, 22.7, 14.9, 14.2; **HRMS** (ESI^+) calc. for $C_{20}H_{31}N_2S$ $[M+H]^+$ 331.2203, found 331.22014.

1-Benzyl-4-hexyl-5-(2-(methylthio)phenyl)-1*H*-pyrazole, 7c

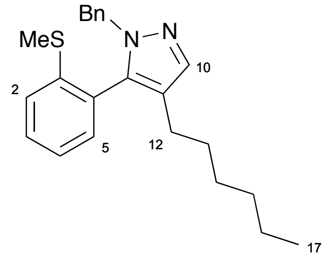

To a vial containing β-enaminone **3a** (100 mg, 0.197 mmol, 1.0 equiv.) and benzylhydrazine hydrochloride (192 mg, 0.985 mmol, 5.0 equiv.) was added ethanol (0.39 mL), and the reaction mixture stirred for 16 h at 80 °C. After reaction completion, water (20 mL) and CH_2Cl_2 (20 mL) were added, and the aqueous phase extracted with CH_2Cl_2 (2 x 20 mL). The crude material was purified by column chromatography (petrol/EtOAc (96:4)) to give **7c** as a colourless oil (58 mg, 0.159 mmol, 81%); **R*f*** 0.28 (petrol/EtOAc (90:10)); **IR** (thin film, ν_{max}/cm^{-1}) 3031, 2924, 2854,

1495, 1453, 1395, 1319, ; **^{1}H NMR** (400 MHz, $CDCl_3$) δ_H 7.51 (1H, s, H10), 7.42 (1H, dt, J = 7.5 and 1.5 Hz, Ar*H*), 7.26 (1H, d, J = 8.0 Hz, Ar*H*), 7.24-7.18 (3H, m, Ar*H*), 7.14 (1H, dt, J = 7.5 and 1.0 Hz, Ar*H*), 7.02-6.98 (3H, m, Ar*H*), 5.19 (1H, d, J = 15.5 Hz, BnCH_2), 4.99 (1H, d, J = 15.5 Hz, BnCH_2), 2.36 (3H, s, SCH_3), 2.34 (1H, ddd, J = 14.5, 8.5 and 7.0 Hz, H12), 2.24 (1H, ddd, J = 14.5, 8.0 and 7.0 Hz, H12), 1.54-1.41 (2H, m, H13), 1.32-1.17 (6H, m, H14-16), 0.86 (3H, t, J = 7.5 Hz, H17); **^{13}C NMR** (101 MHz, $CDCl_3$) δ_C 140.6, 138.6, 138.5, 137.4, 131.5, 129.7, 128.6, 128.4, 127.6, 127.4, 124.4, 124.3, 121.5, 53.6, 31.7, 30.5, 29.0, 24.0, 22.7, 15.1, 14.2; **HRMS** (ESI$^+$) calc. for $C_{22}H_{29}N_2S$ [M+H]$^+$ 365.2046, found 365.2044.

3.7 Pyrimidine products

5-Hexyl-4-(2-(methylthio)phenyl)-2-phenylpyrimidine, 8a

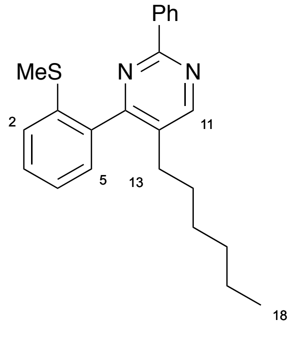

To a vial containing β-enaminone **3a** (100 mg, 0.197 mmol, 1.0 equiv.) and benzamidine hydrochloride (154 mg, 0.985 mmol, 5.0 equiv.) was added ethanol (0.39 mL) and the reaction mixture stirred for 16 h at 80 °C. After reaction completion, water (20 mL) and CH_2Cl_2 (20 mL) were added, and the aqueous phase extracted with CH_2Cl_2 (2 x 20 mL). The crude material was purified by column chromatography (petrol/EtOAc (96:4)) to give **8a** as a colourless oil (57 mg, 0.157 mmol, 80%); **R_f** 0.43 (petrol/EtOAc (90:10)); **IR** (thin film, ν_{max}/cm^{-1}) 2960, 2920, 2850, 2359, 1677, 1648, 1588, 1423, 1292, 1159; **^{1}H NMR** (500 MHz, $CDCl_3$) δ_H 8.75 (1H, s, H11), 8.50-8.47 (2H, m, Ar*H*), 7.50-7.40 (5H, m, Ar*H*), 7.31-7.26 (2H, m, Ar*H*), 2.54 (2H, t, *J* = 8.0 Hz, H13), 2.41 (3H, s, SC*H*3), 1.52-1.46 (2H, m, H14), 1.26-1.15 (6H, m, H15-17), 0.85 (3H, t, *J* = 7.5 Hz, H18); **^{13}C NMR** (125 MHz, $CDCl_3$) δ_C 165.4, 162.2, 158.2, 137.9, 137.9, 137.2, 131.6, 130.3, 129.3, 129.0, 128.5, 128.2, 126.8, 125.1, 31.5, 30.0, 29.6, 28.9, 22.5, 16.5, 14.1; **HRMS** (ESI^+) calc. for $C_{23}H_{27}N_2S$ $[M+H]^+$ 363.1890, found 363.1890.

5-Hexyl-4-(2-(methylthio)phenyl)pyrimidin-2-amine, 8b

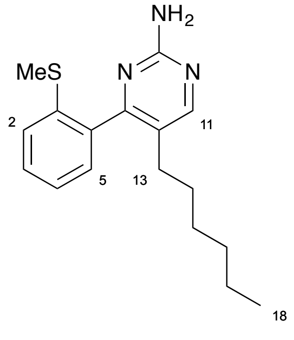

To a vial containing β-enaminone **3a** (100 mg, 0.197 mmol, 1.0 equiv.), guanidine hydrochloride (94 mg, 0.985 mmol, 5.0 equiv.) and K_2CO_3 (163 mg, 1.182 mmol, 6.0 equiv.) was added ethanol

(0.39 mL) and the reaction mixture stirred for 16 h at 80 °C. After reaction completion, 1 M *aq.* HCl (20 mL) and CH_2Cl_2 (20 mL) were added, and the organic phase extracted with 1 M *aq.* HCl (2 x 20 mL), and 1 M *aq.* NaOH (3 x 20 mL). The organic phase was dried over magnesium sulfate, filtered, and concentrated *in vacuo* to give **8b** as a colourless oil (58 mg, 0.192 mmol, 98%); **R*f*** 0.23 (petrol/EtOAc (50:50)); **IR** (thin film, ν_{max}/cm^{-1}) 2923, 1624, 1542, 1469, 1277, 1260; **^{1}H NMR** (400 MHz, $CDCl_3$) δ_H 8.22 (1H, s, H11), 7.38 (1H, ddd, *J* = 8.0, 7.5 and 1.5 Hz, Ar*H*), 7.30 (1H, d, *J* = 8.0 Hz, Ar*H*), 7.22 (1H, td, *J* = 7.5 and 1.0 Hz, Ar*H*), 7.14 (1H, dd, *J* = 7.5 and 1.5 Hz, Ar*H*), 5.11 (2H, s, N*H*$_2$), 2.40 (3H, s, SC*H*$_3$), 2.30 (2H, t, *J* = 7.5 Hz, H13), 1.39-1.31 (2H, m, H14), 1.20-1.10 (6H, m, H15-17), 0.80 (3H, t, *J* = 7.0 Hz, H18); **^{13}C NMR** (101 MHz, $CDCl_3$) δ_C 166.3, 161.3, 159.3, 137.5, 136.7, 129.2, 128.5, 126.0, 125.0, 123.8, 31.5, 30.4, 28.9, 28.9, 22.5, 16.1, 14.2; **HRMS** (ESI$^+$) calc. for $C_{17}H_{24}N_3S^+$ [M+H]$^+$ 302.1686, found 302.1687.

5-Hexyl-4-(2-(methylthio)phenyl)-*N*-phenylpyrimidin-2-amine, 8c

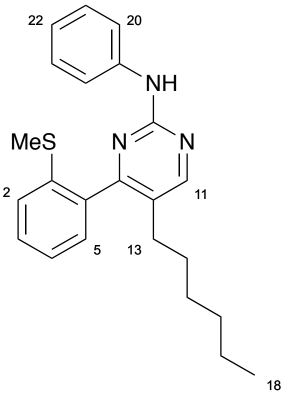

To a vial containing β-enaminone **3a** (100 mg, 0.197 mmol, 1.0 equiv.), phenylguanidine acetate salt (194 mg, 0.985 mmol, 5.0 equiv.) and K_2CO_3 (27 mg, 0.197 mmol, 1.0 equiv.) was added DMF (0.39 mL) and the reaction mixture stirred for 16 h at 80 °C. After reaction completion, water (20 mL) and CH_2Cl_2 (20 mL) were added, and the aqueous phase extracted with CH_2Cl_2 (2 x 20 mL). The crude material was purified by column chromatography (petrol/EtOAc (95:5)) to give **8c** as a colourless oil (48 mg, 0.127 mmol, 65%); **R*f*** 0.23 (petrol/EtOAc (90:10)); **IR** (thin film, ν_{max}/cm^{-1}) 3772, 2959, 2919, 2852, 1581, 1553, 1516, 1497, 1444, 1265; **^{1}H NMR** (400 MHz, $CDCl_3$) δ_H 8.39 (1H, br. s, H11), 7.65 (2H, d, *J* = 8.5 Hz, Ar*H*), 7.41 (1H, ddd, *J* = 8.0, 7.0 and 1.5 Hz, Ar*H*), 7.35 (1H, d, *J* = 8.0 Hz, Ar*H*), 7.31 (2H, t, *J* = 8.0 Hz, Ar*H*), 7.27-7.23 (3H, m, N*H* and Ar*H*), 7.20 (1H, dd, *J* = 7.5 and 1.5 Hz, Ar*H*), 7.00 (1H, tt, *J* = 7.5 and 1.0 H, Ar*H*), 2.42 (3H, s, SC*H*$_3$), 2.37 (2H, t, *J* = 8.0 Hz, H13), 1.43-1.36 (2H, m, H14), 1.24-1.11 (6H, m, H15-17), 0.82 (3H, t, *J* = 7.0 Hz, H18); **^{13}C NMR** (101 MHz, $CDCl_3$) δ_C 166.2, 158.8, 158.3, 140.0, 137.5, 136.9, 129.2, 129.0, 128.6,

126.1, 125.0, 124.8, 122.1, 118.8, 31.5, 30.3, 29.0, 28.8, 22.5, 16.2, 14.2; **HRMS** (ESI^+) calc. for $C_{23}H_{28}N_3S$ $[M+H]^+$ 378.1999, found 378.2014.

4 Assignment of regiochemistry

4.1: The regiochemical assignment of isoxazoles **5** are based on nOe ehancements measured using ^{1}H-^{1}H nOe experiments for the *N*-methylated compound **S1**. A strong enhancement was observed between the protons of the *N*-methyl group and H_a. An equally strong enhancement was observed between H_a and the *ortho*-protons, H_c, of the phenyl ring. Crucially, no enhancement was observed between the SMe_2 protons and the *N*-methyl protons, suggesting a 4,5-substitution pattern, as depicted.

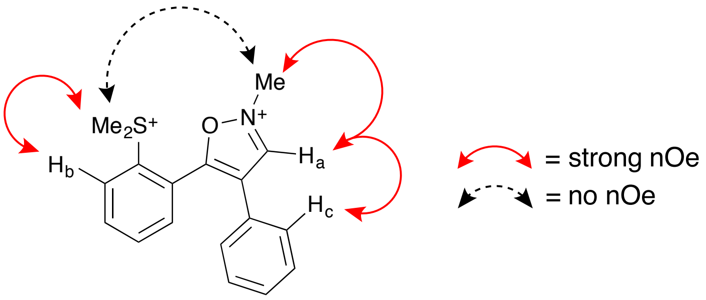

4.2: The regiochemical assignment of pyrazoles **6** are based on nOe enhancements measured using ^{1}H-^{1}H nOe experiements for compound **6b** (see Section 6). A strong enhancement was observed between the protons of the *tert*-butyl imidazole substituent and the *ortho*-proton of the phenyl ring. A similarly strong enhancement was observed between the imidazole proton H_a and the H_b and H_c protons of the hexyl chain. Only a weak enhancement was observed between the *tert*-butyl protons and the imidazole proton H_a. These observed through-space interactions suggest a 4,5-substitution pattern, as depicted.

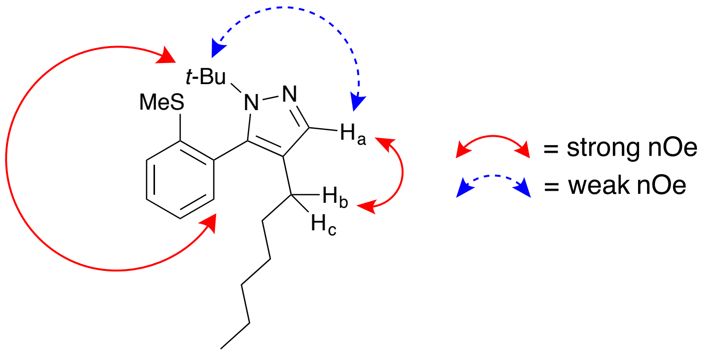

5 References

(1) Stahl, E. *Thin Layer Chromatography*; Springer-Verlag, Berlin, 1969.

(2) Majhail, M. K.; Ylioja, P. M.; Willis, M. C. *Chem. Eur. J.* **2016**, *22*, 7879.

(3) Neuhaus, J. D.; Morrow, S. M.; Brunavs, M.; Willis, M. C. *Org. Lett.* **2016**, *18*, 1562.

(4) Hamada, T.; Ye, X.; Stahl, S. S. *J. Am. Chem. Soc.* **2008**, *130*, 833.

(5) Mansfield, S. J.; Campbell, C. D.; Jones, M. W.; Anderson, E. A. *Chem. Commun.* **2015**, *51*, 3316.

(6) Pan, F.; Shu, C.; Ping, Y.-F.; Pan, Y.-F.; Ruan, P.-P.; Fei, Q.-R.; Ye, L.-W. *J. Org. Chem.* **2015**, *80*, 10009.

(7) Martínez-Esperón, M. F.; Rodríguez, D.; Castedo, L.; Saá, C. *Tetrahedron* **2006**, *62*, 3843.

(8) Schotes, C.; Mezzetti, A. *Angew. Chem. Int. Ed.* **2011**, *50*, 3072.

(9) Zhang, Y.; Hsung, R. P.; Tracey, M. R.; Kurtz, K. C. M.; Vera, E. L. *Org. Lett.* **2004**, *6*, 1151.

(10) Riddell, N.; Villeneuve, K.; Tam, W. *Org. Lett.* **2005**, *7*, 3681.

(11) Sato, A.; Yorimitsu, H.; Oshima, K. *Bull. Korean Chem. Soc.* **2010**, *31*, 570.

6 NMR Spectra

Benzyl oct-1-yn-1-yl(phenyl)carbamate, 2b

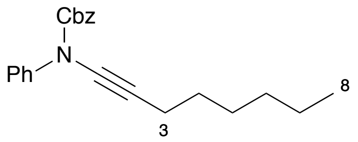

^{1}H NMR (400 MHz, $CDCl_3$)

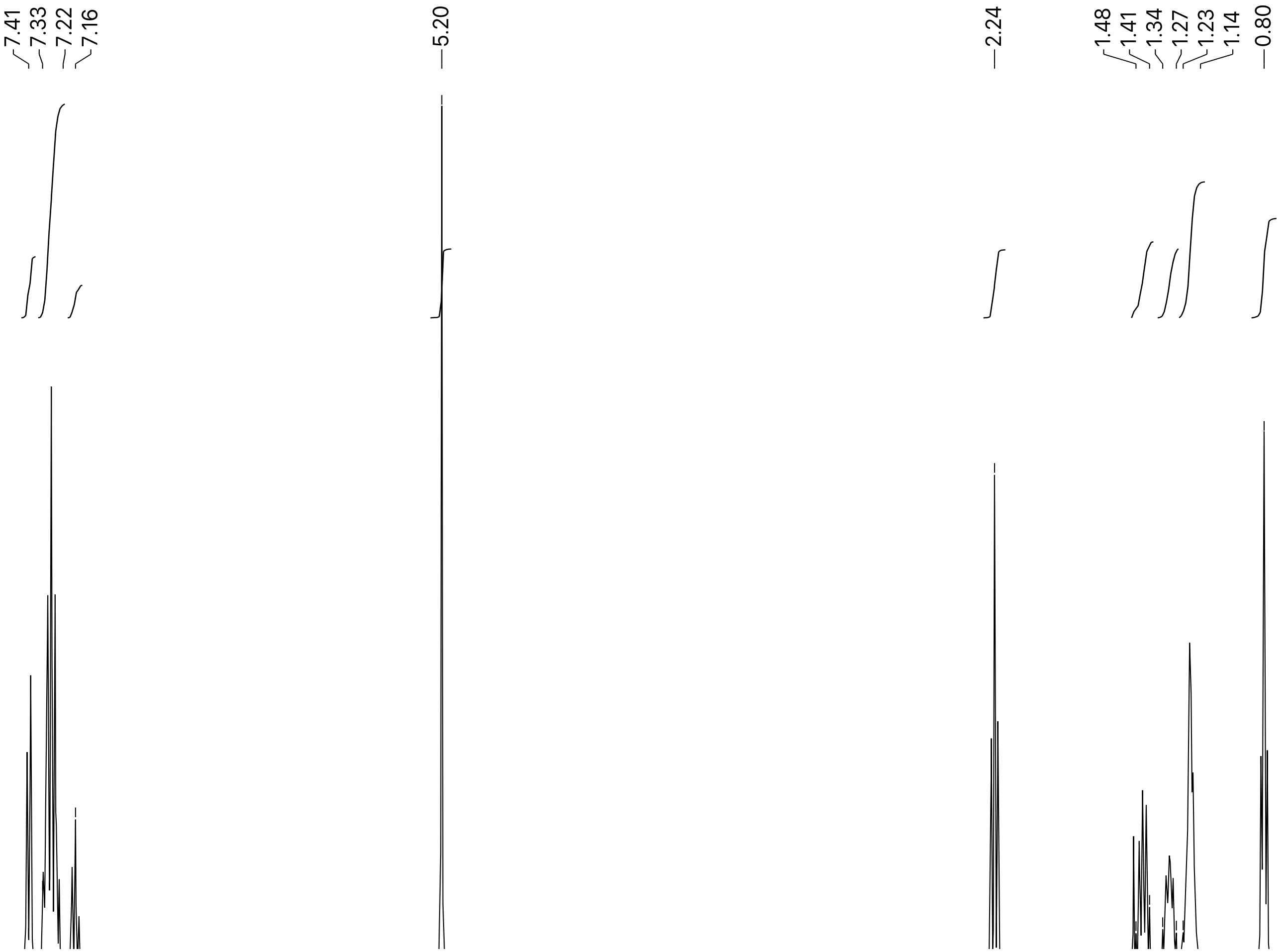

Benzyl oct-1-yn-1-yl(phenyl)carbamate, 2b

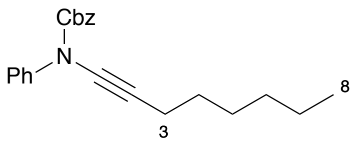

^{13}C NMR (101 MHz, $CDCl_3$)

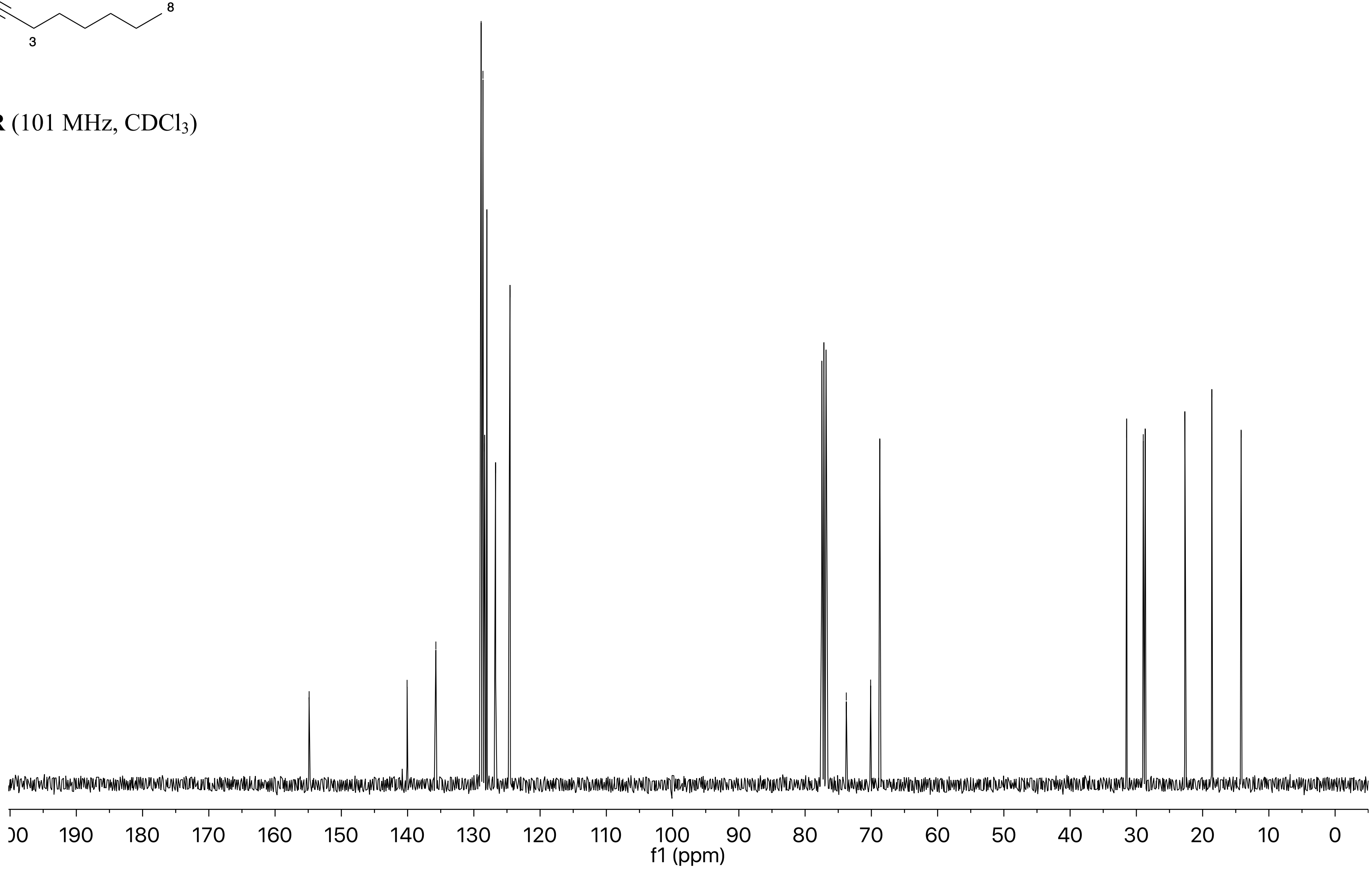

N-(5-Chloropent-1-yn-1-yl)-4-methyl-*N*-phenylbenzenesulfonamide, 2e

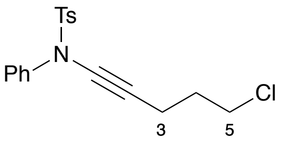

^{1}H NMR (400 MHz, $CDCl_3$)

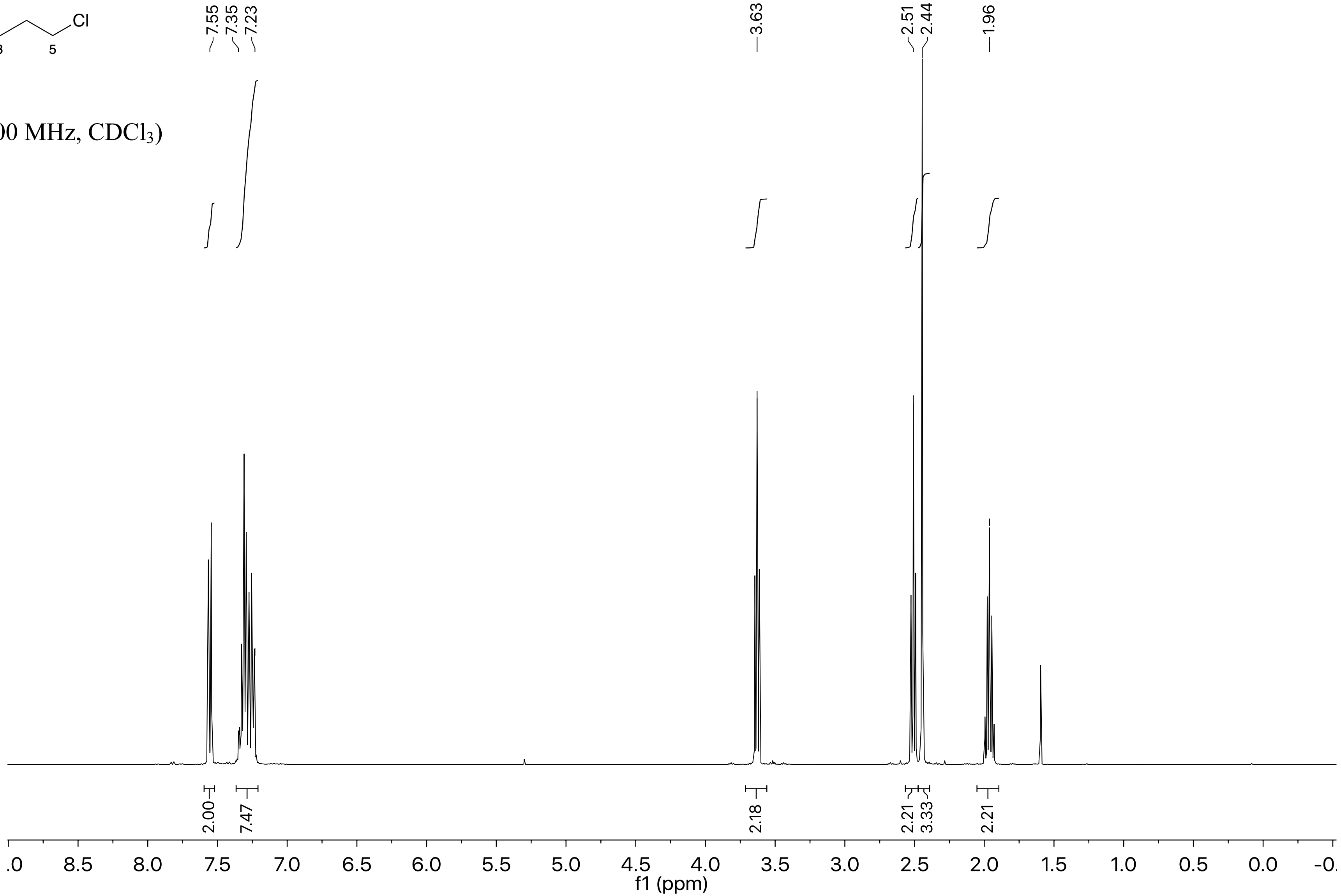

***N*-(5-Chloropent-1-yn-1-yl)-4-methyl-*N*-phenylbenzenesulfonamide, 2e**

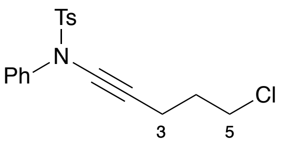

^{13}C NMR (101 MHz, CDCl$_3$)

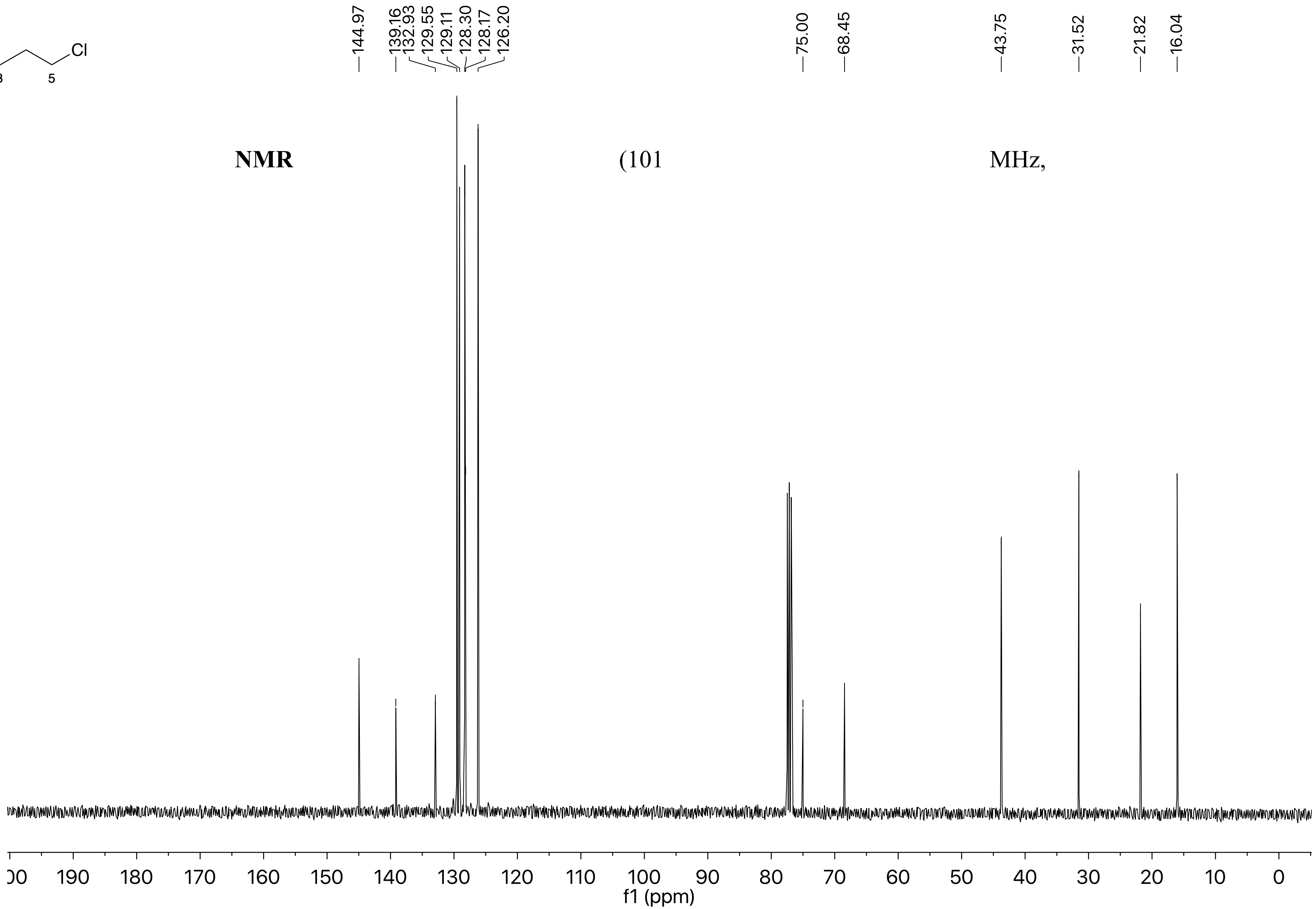

N-(5-((tert-Butyldimethylsilyl)oxy)pent-1-yn-1-yl)-4-methyl-N-phenylbenzenesulfonamide, 2f

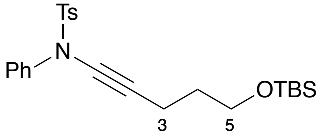

^{1}H NMR (400 MHz, CDCl$_3$)

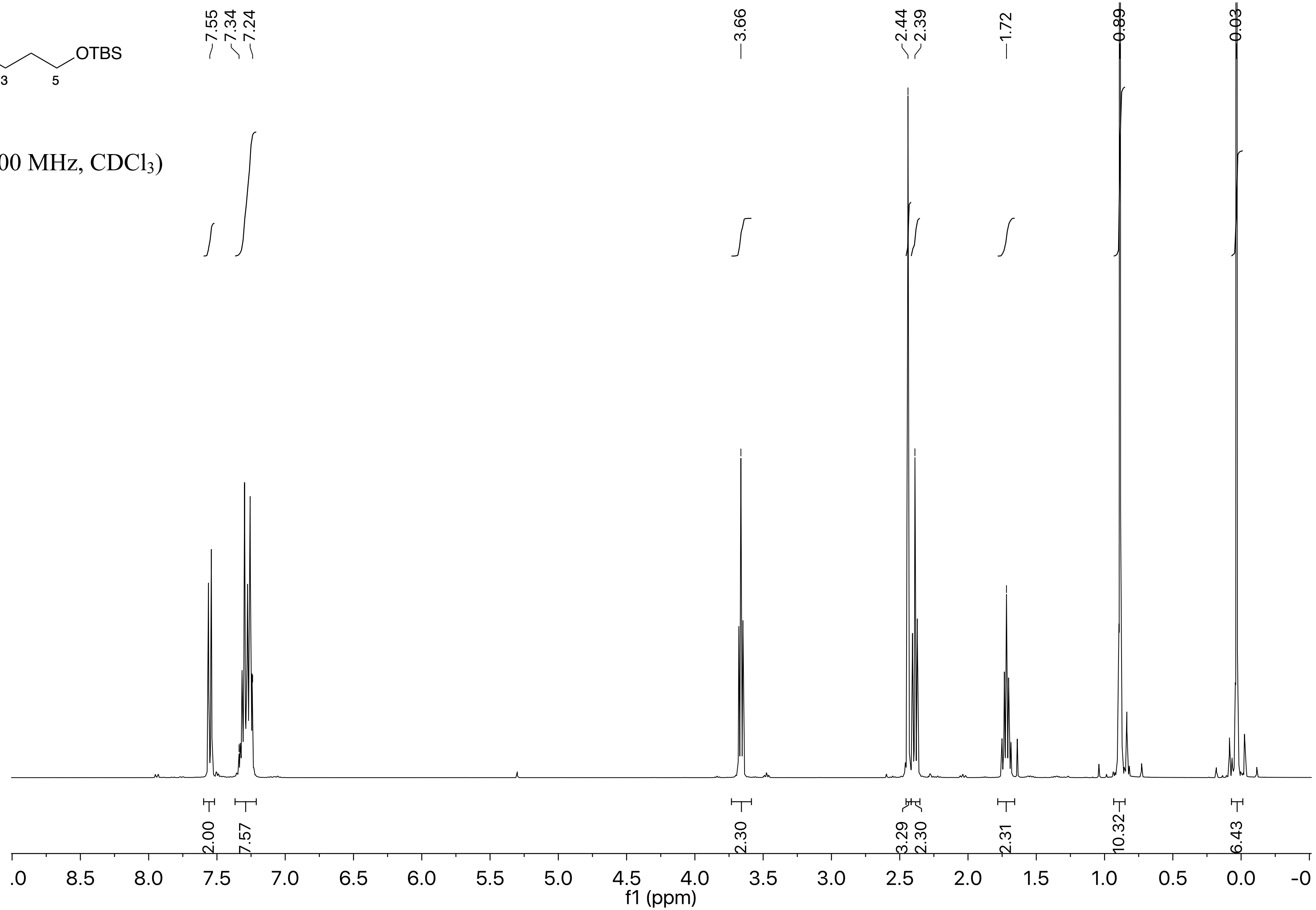

N-(5-((*tert*-Butyldimethylsilyl)oxy)pent-1-yn-1-yl)-4-methyl-*N*-phenylbenzenesulfonamide, 2f

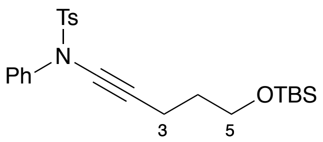

^{13}C NMR (101 MHz, $CDCl_3$)

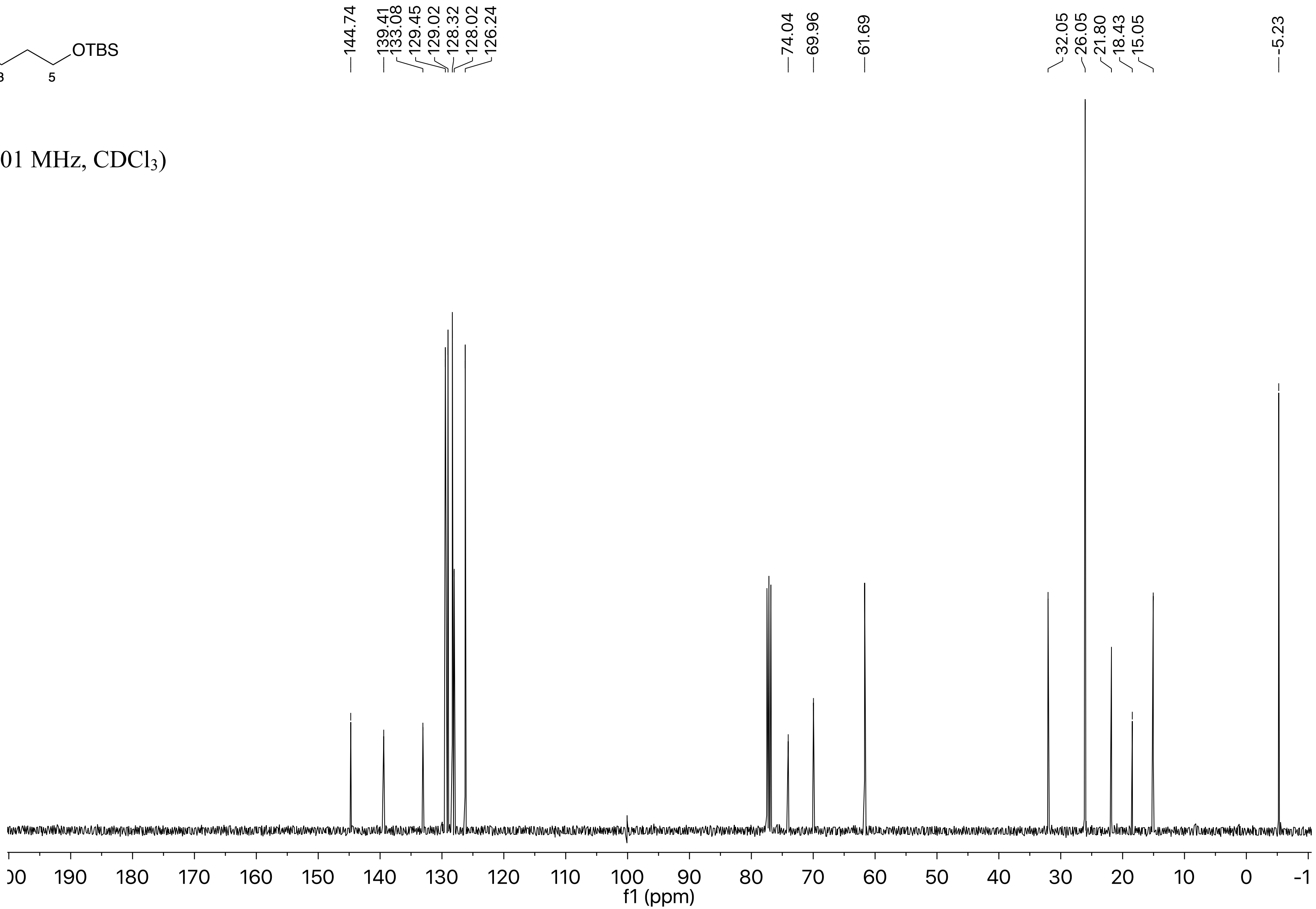

N-((3,5-Dimethoxyphenyl)ethynyl)-4-methyl-N-phenylbenzenesulfonamide, 2j

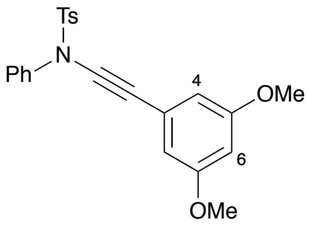

^{1}H NMR (400 MHz, $CDCl_3$)

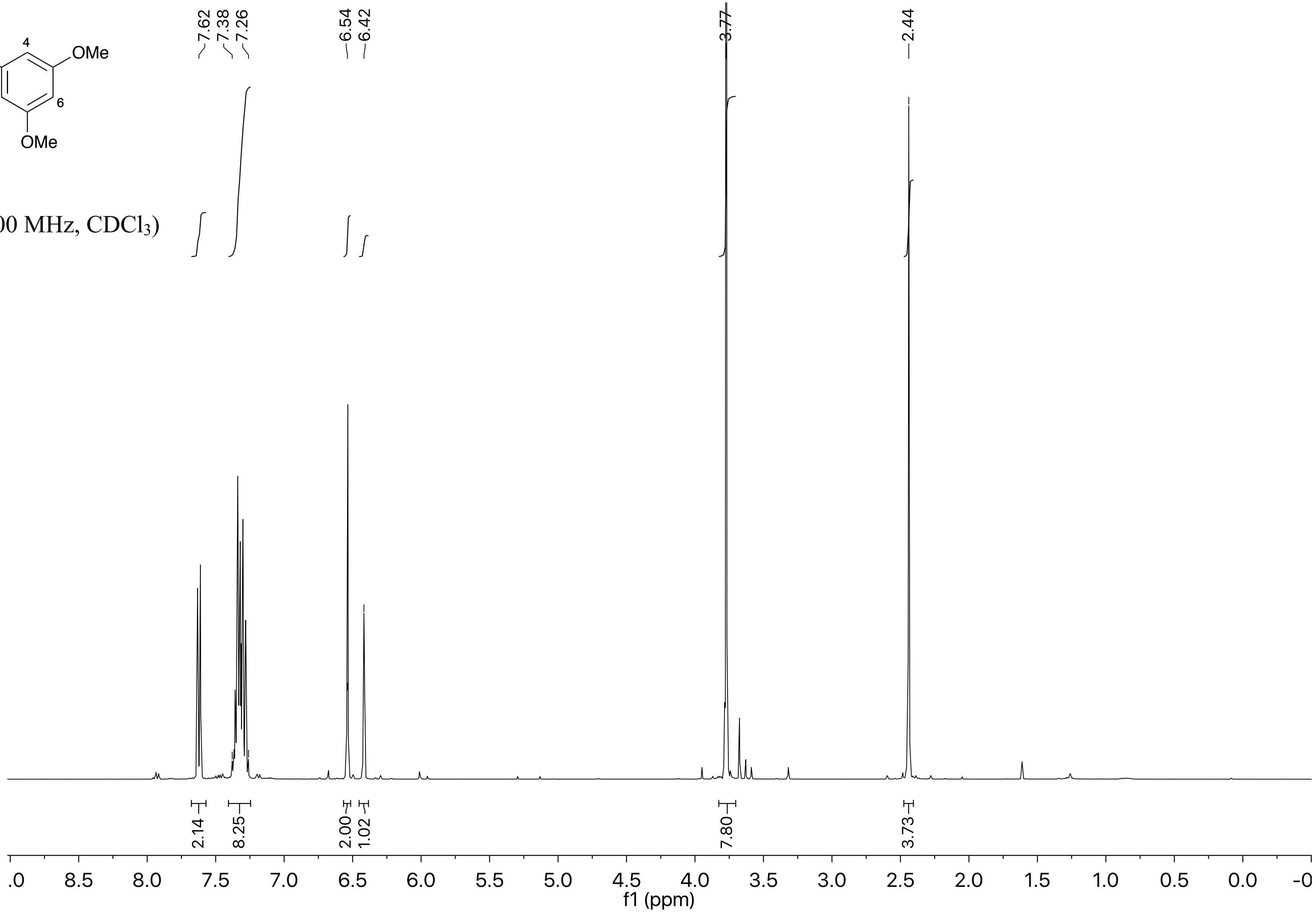

N-((3,5-Dimethoxyphenyl)ethynyl)-4-methyl-N-phenylbenzenesulfonamide, 2j

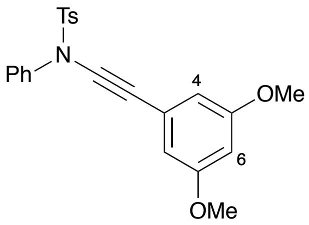

^{13}C NMR (101 MHz, $CDCl_3$)

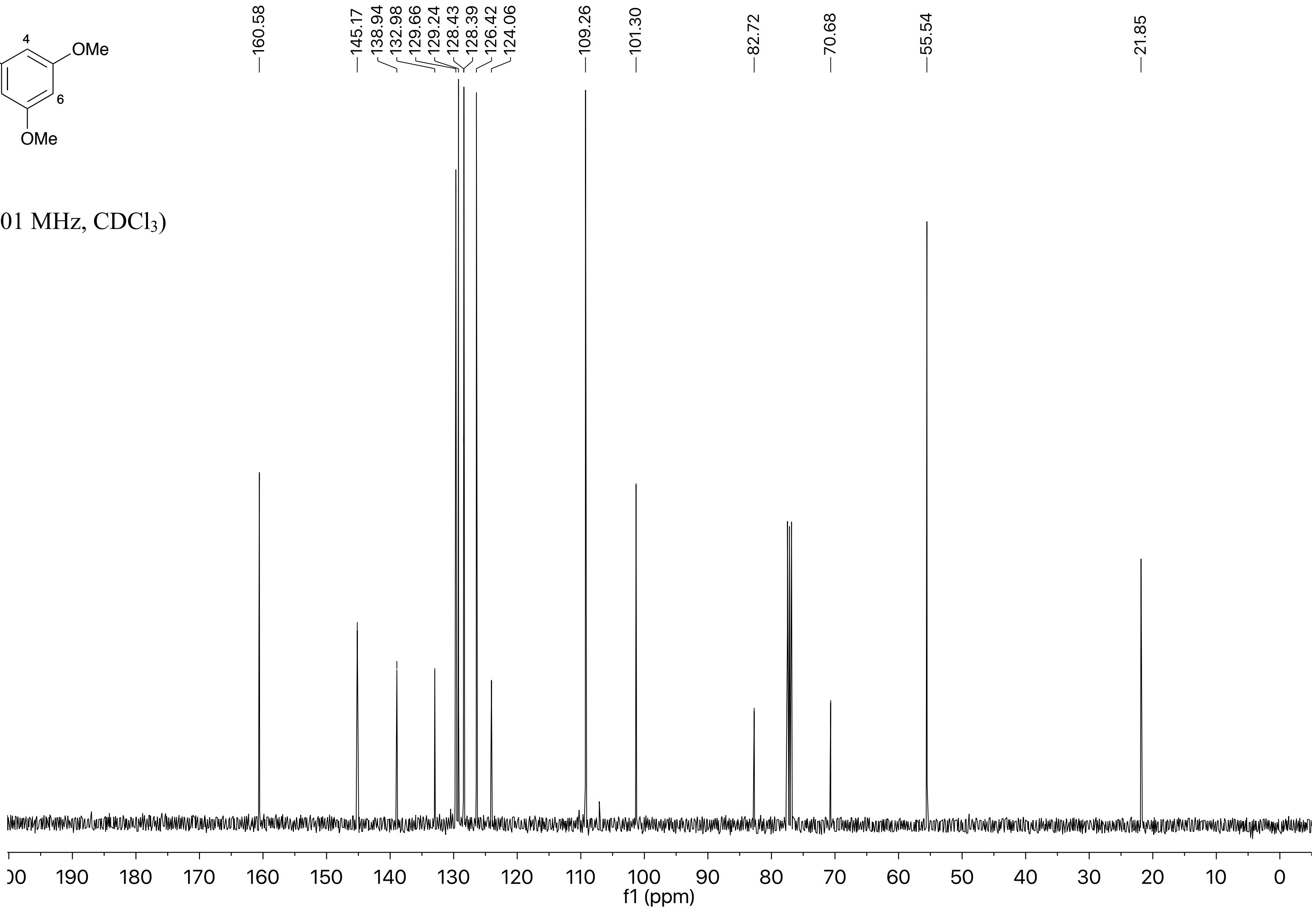

4-Methyl-*N*-phenyl-*N*-(thiophen-3-ylethynyl)benzenesulfonamide, 2k

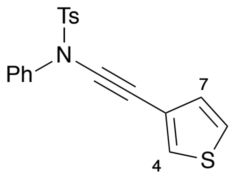

^{1}H NMR (400 MHz, $CDCl_3$)

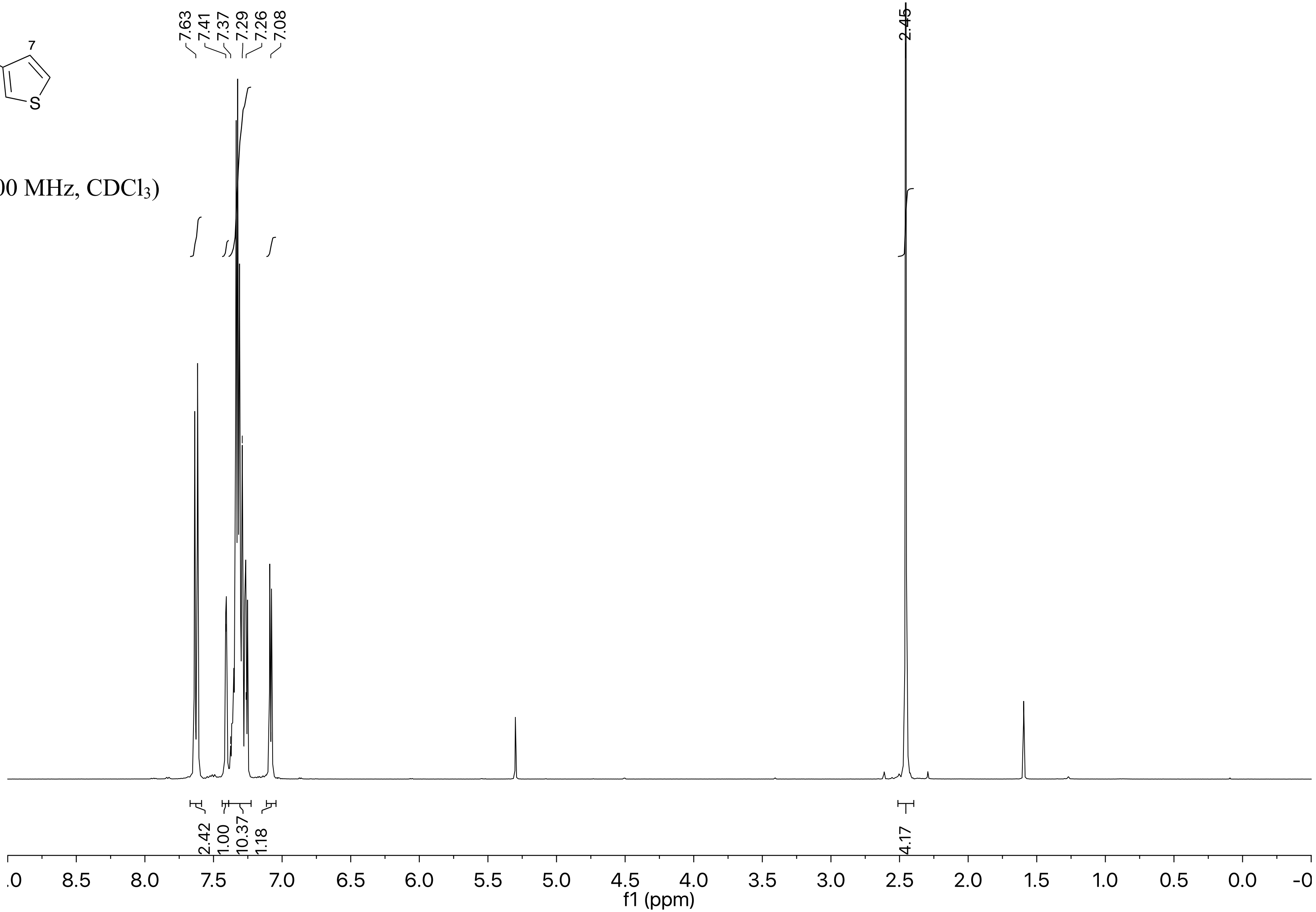

4-Methyl-*N*-phenyl-*N*-(thiophen-3-ylethynyl)benzenesulfonamide, 2k

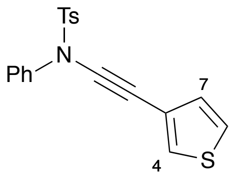

^{13}C NMR (101 MHz, $CDCl_3$)

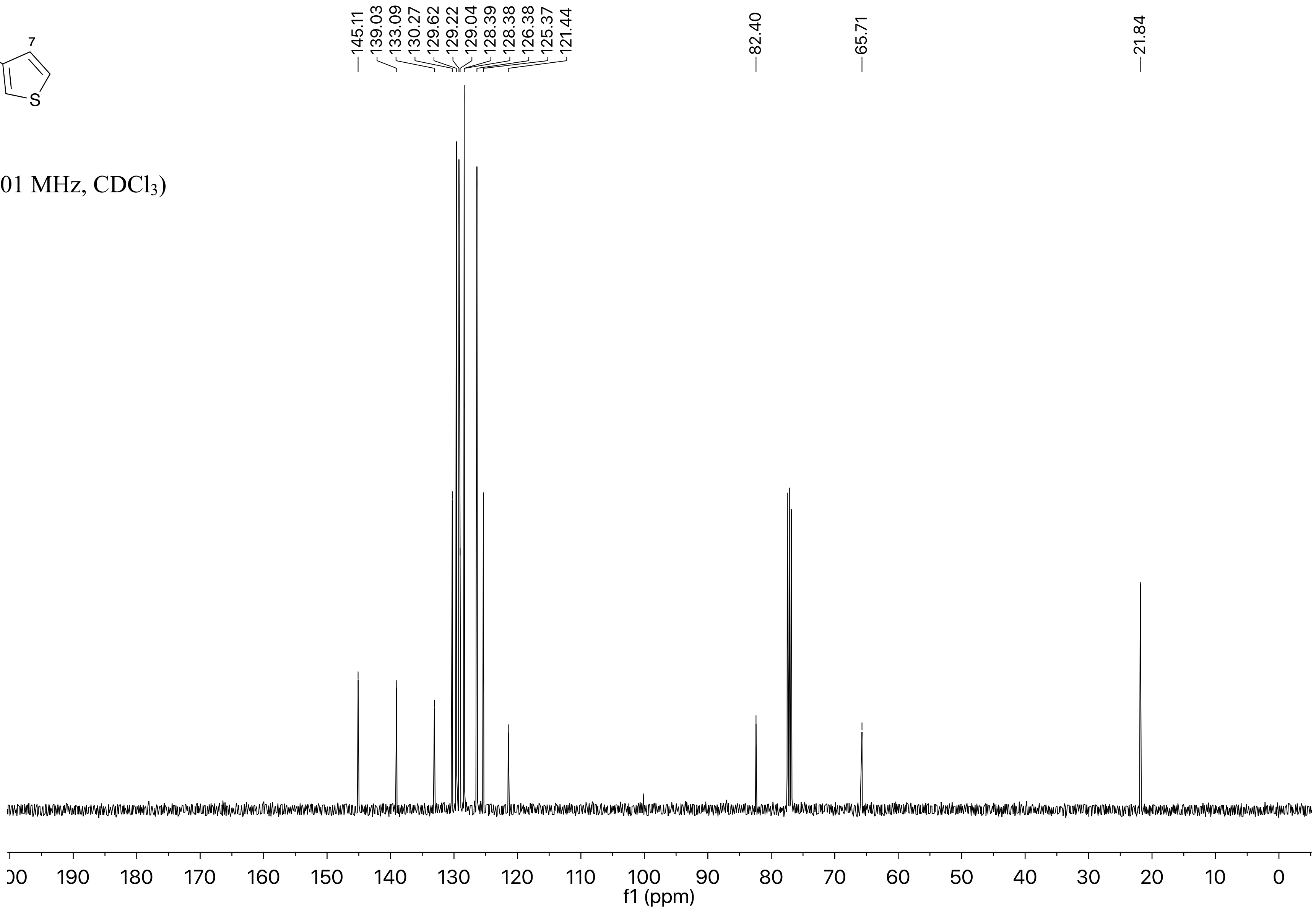

***N*-(Cyclohex-1-en-1-ylethynyl)-4-methyl-*N*-phenylbenzenesulfonamide, 2l**

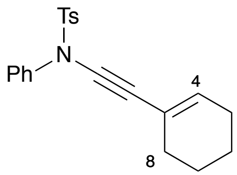

^{1}H NMR (400 MHz, $CDCl_3$)

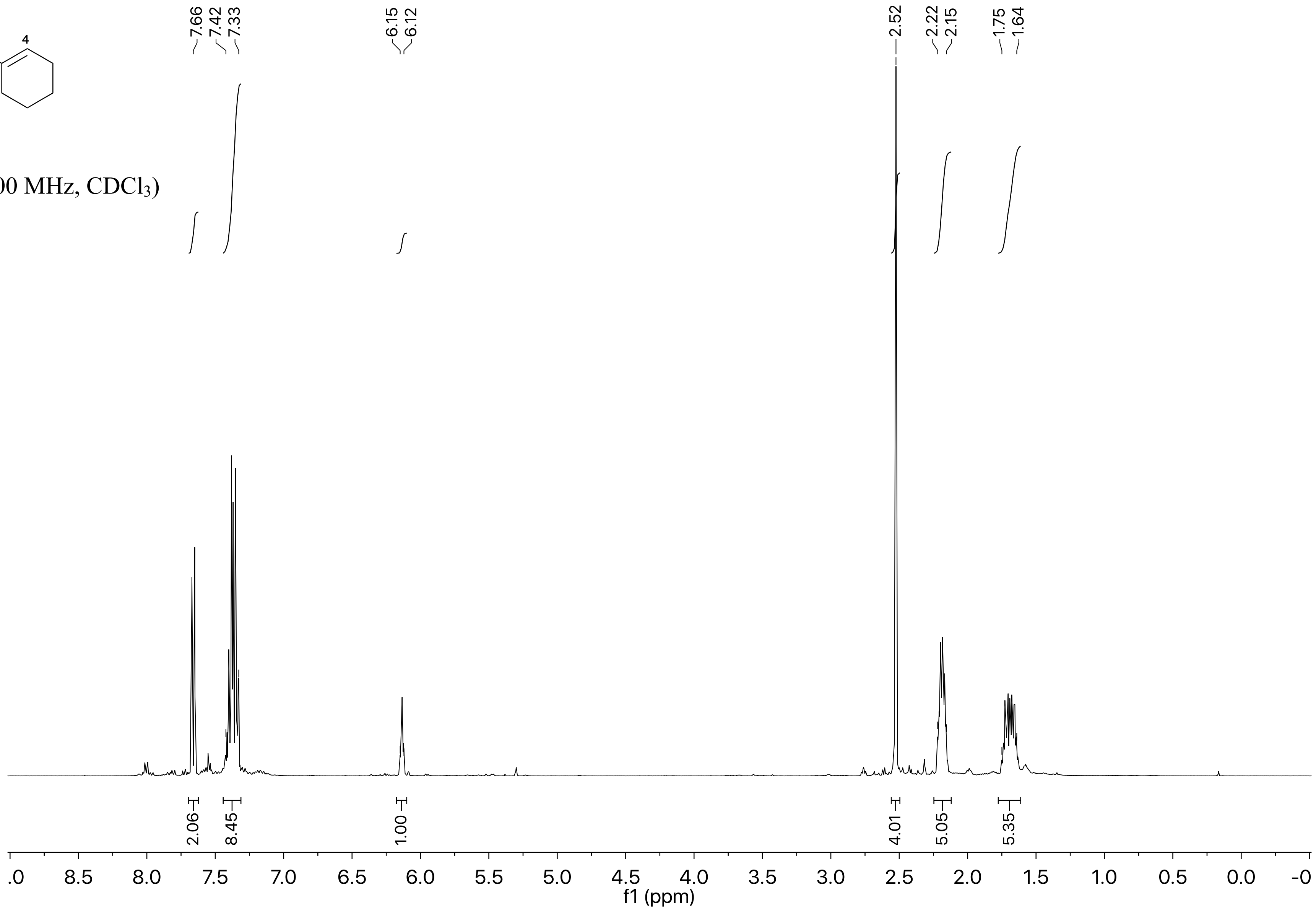

N-(Cyclohex-1-en-1-ylethynyl)-4-methyl-*N*-phenylbenzenesulfonamide, 2l

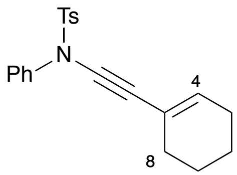

^{13}C NMR (101 MHz, $CDCl_3$)

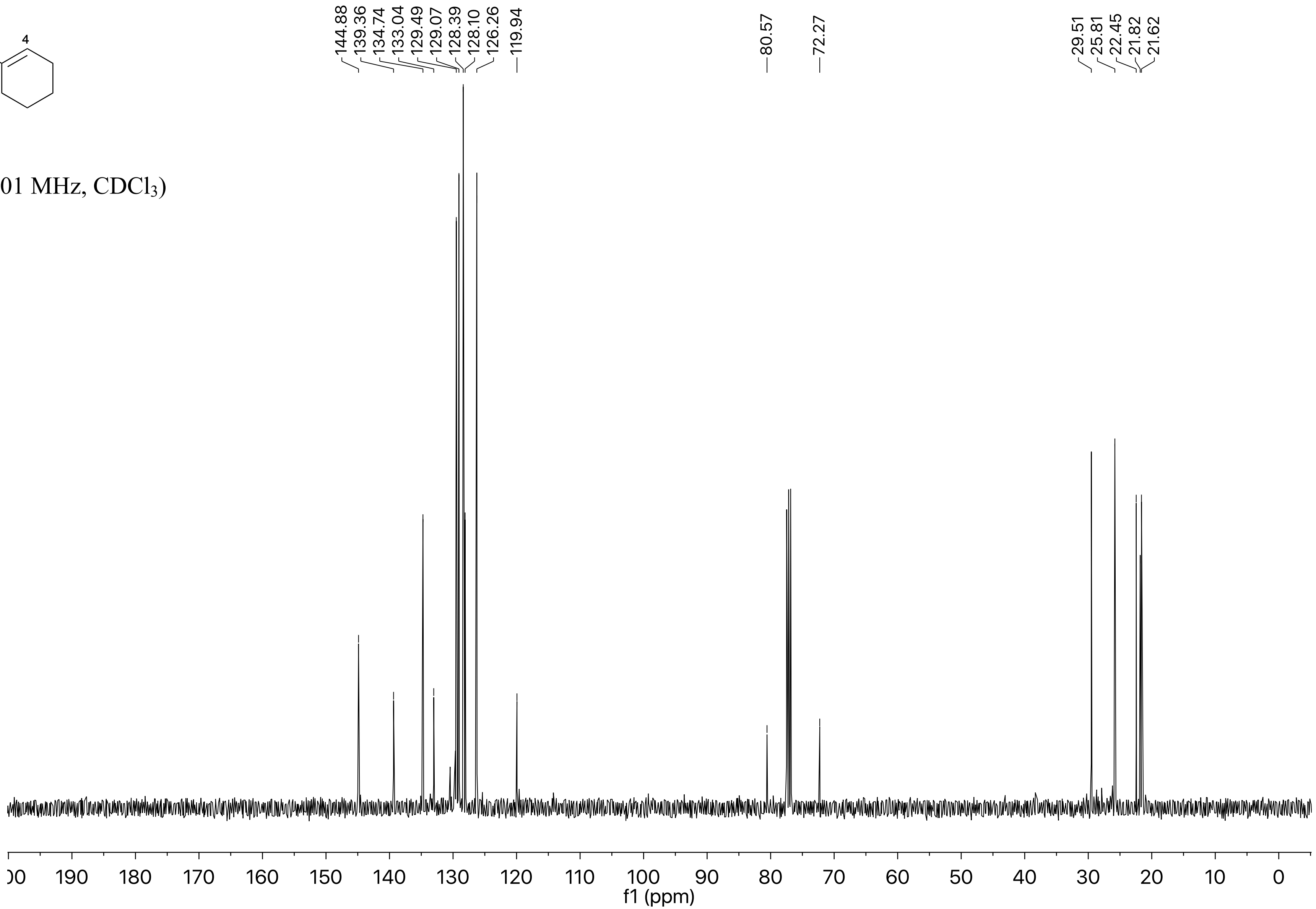

(*E*)-4-Methyl-*N*-(2-(2-(methylthio)benzoyl)oct-1-en-1-yl)-*N*-phenylbenzenesulfonamide, 3a

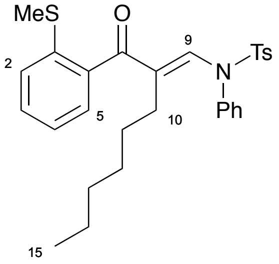

^{1}H NMR (400 MHz, $CDCl_3$)

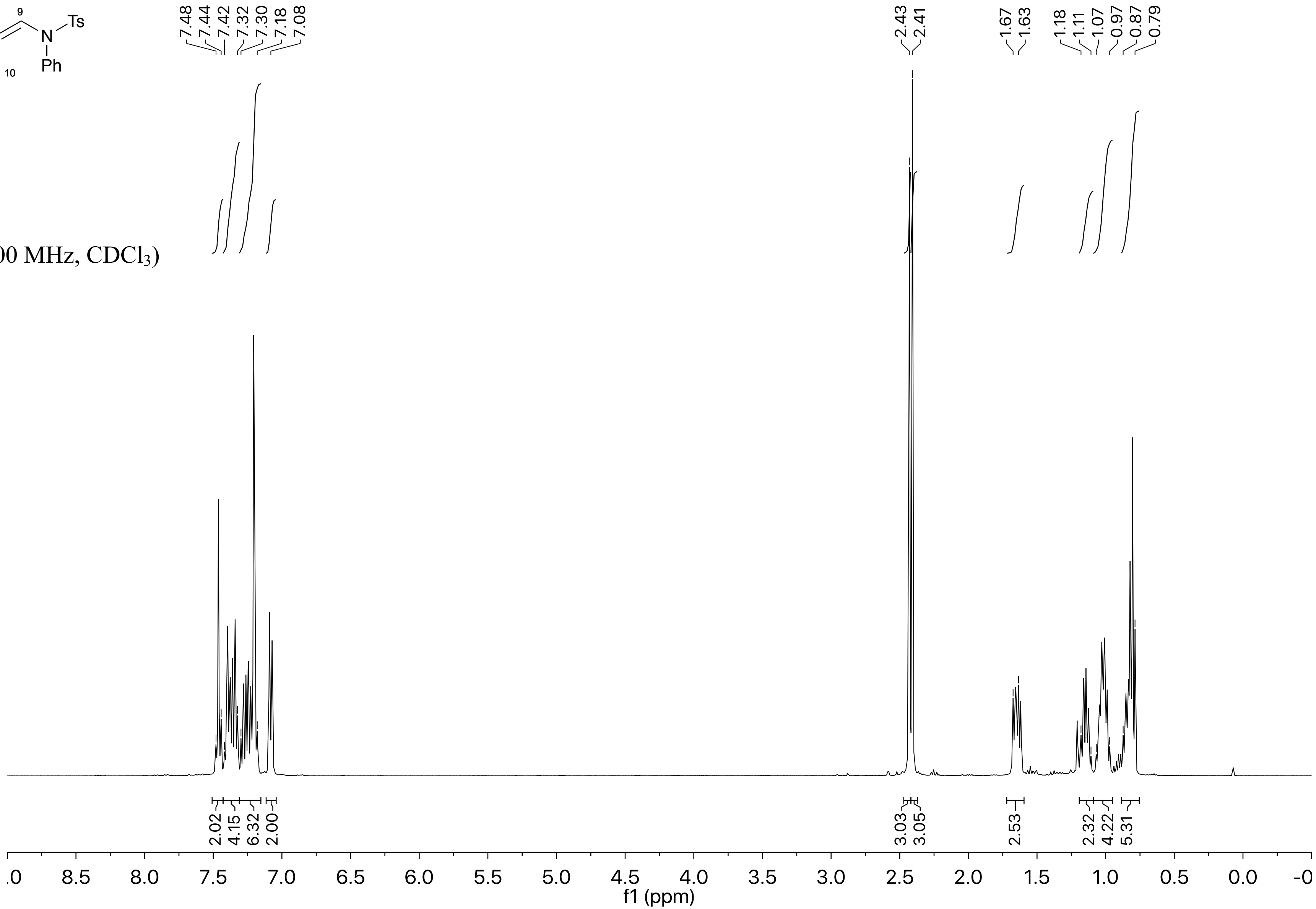

(*E*)-4-Methyl-*N*-(2-(2-(methylthio)benzoyl)oct-1-en-1-yl)-*N*-phenylbenzenesulfonamide, 3a

^{13}C NMR (101 MHz, $CDCl_3$)

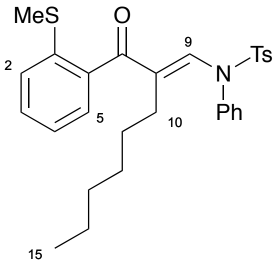

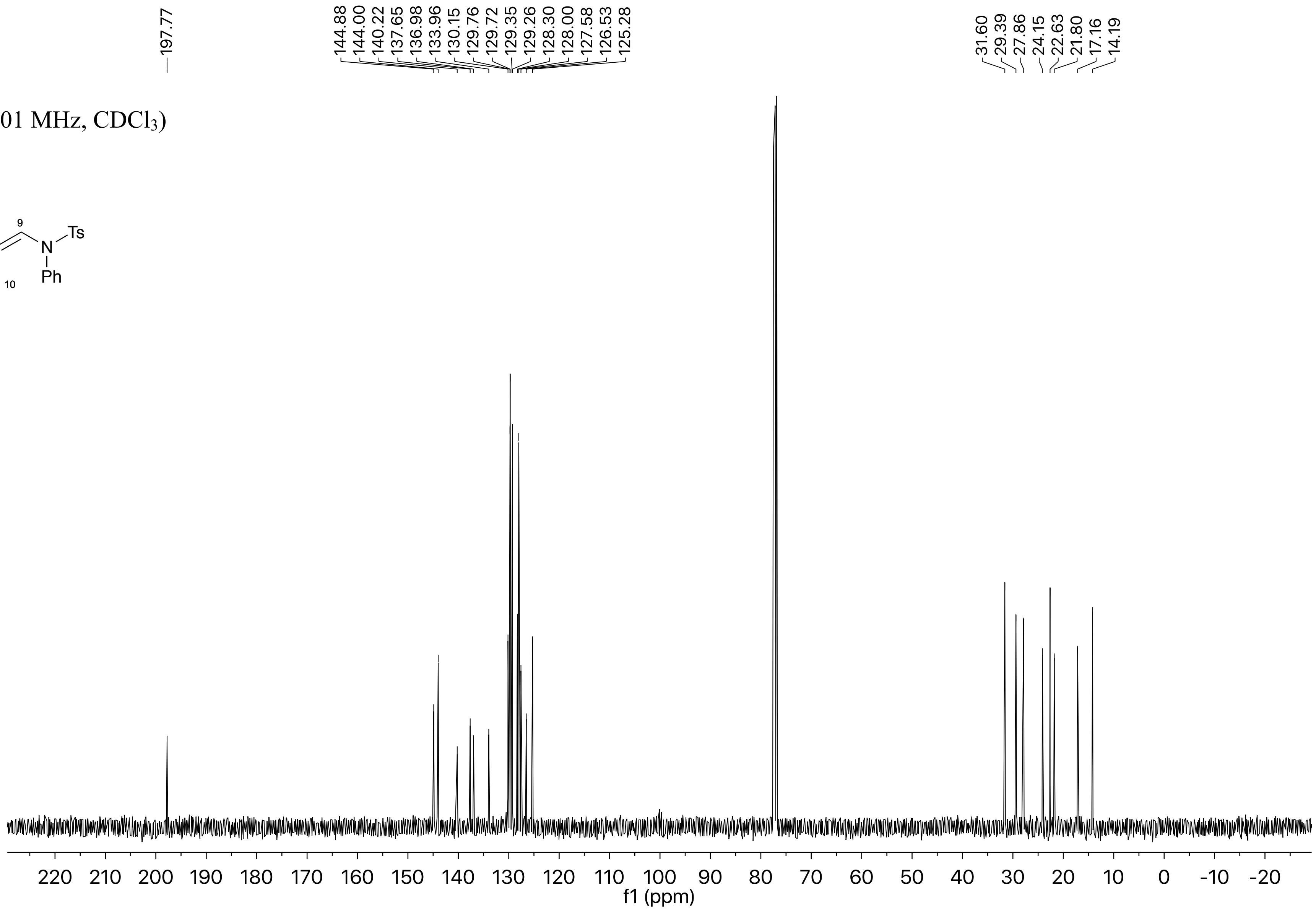

(*Z*)-4-Methyl-*N*-(1-(2-(methylthio)phenyl)-1-oxonon-2-en-2-yl)-*N*-phenylbenzenesulfonamide, 4a

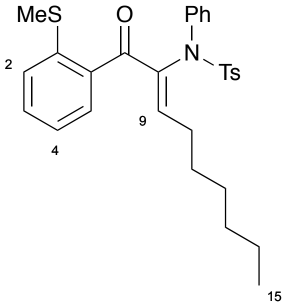

^{1}H NMR (400 MHz, $CDCl_3$)

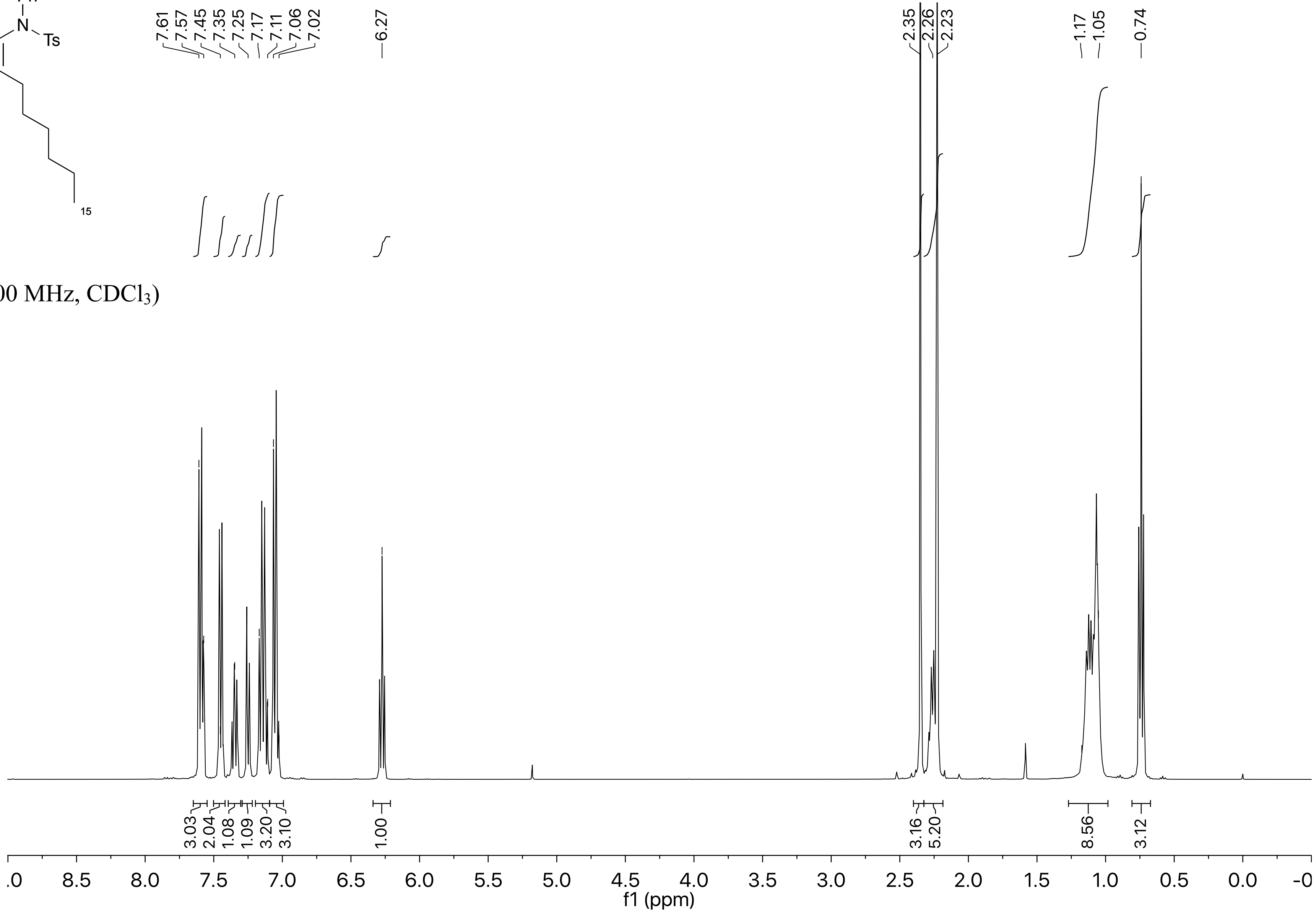

(*Z*)-4-Methyl-*N*-(1-(2-(methylthio)phenyl)-1-oxonon-2-en-2-yl)-*N*-phenylbenzenesulfonamide, 4a

^{13}C NMR (101 MHz, $CDCl_3$)

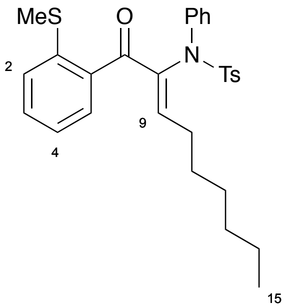

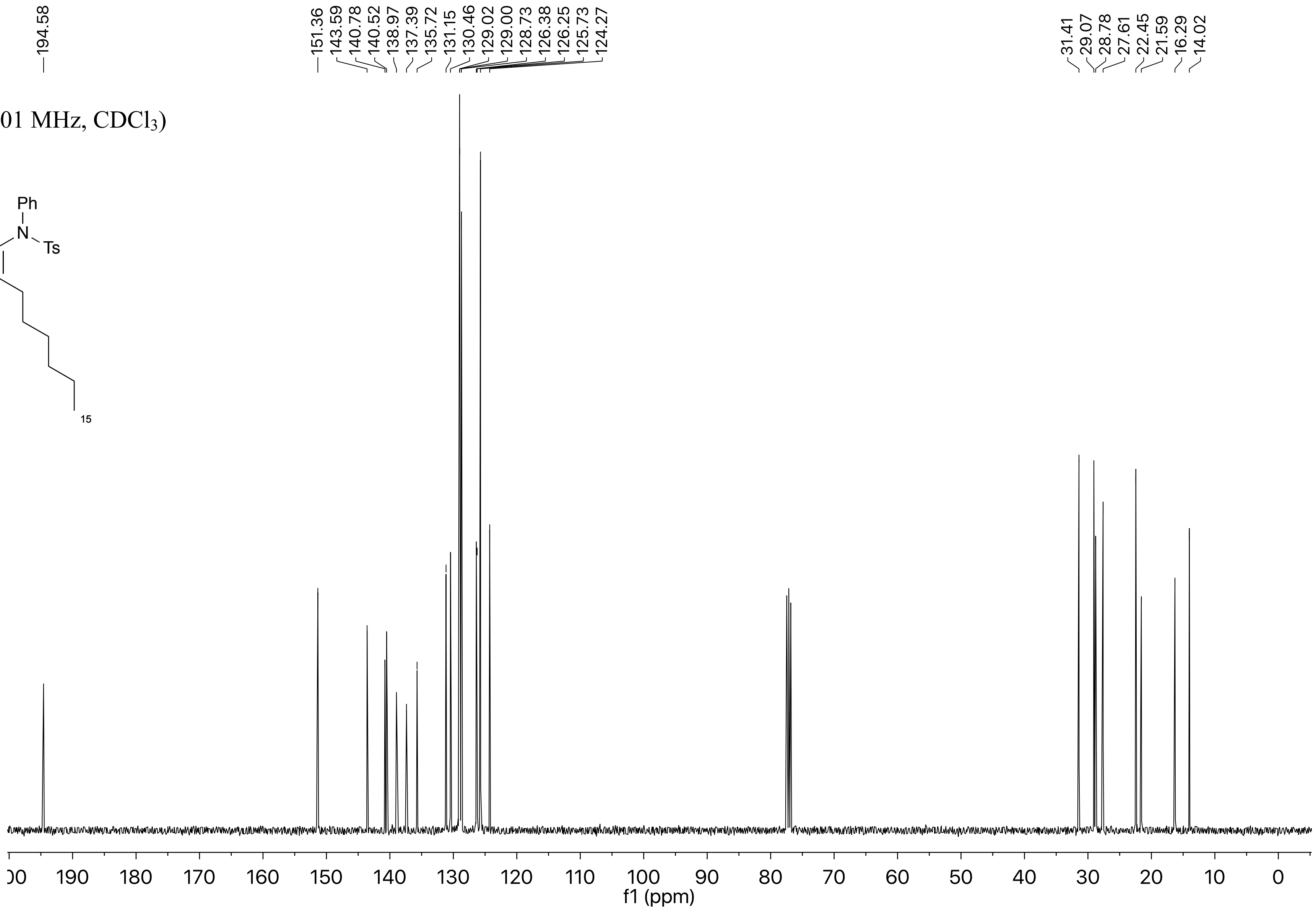

(E)-2,4-Dimethyl-1-(2-(methylthio)phenyl)pent-2-en-1-one, 3aa

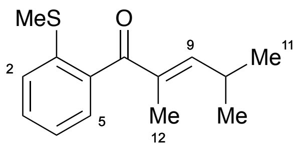

^{1}H NMR (400 MHz, $CDCl_3$)

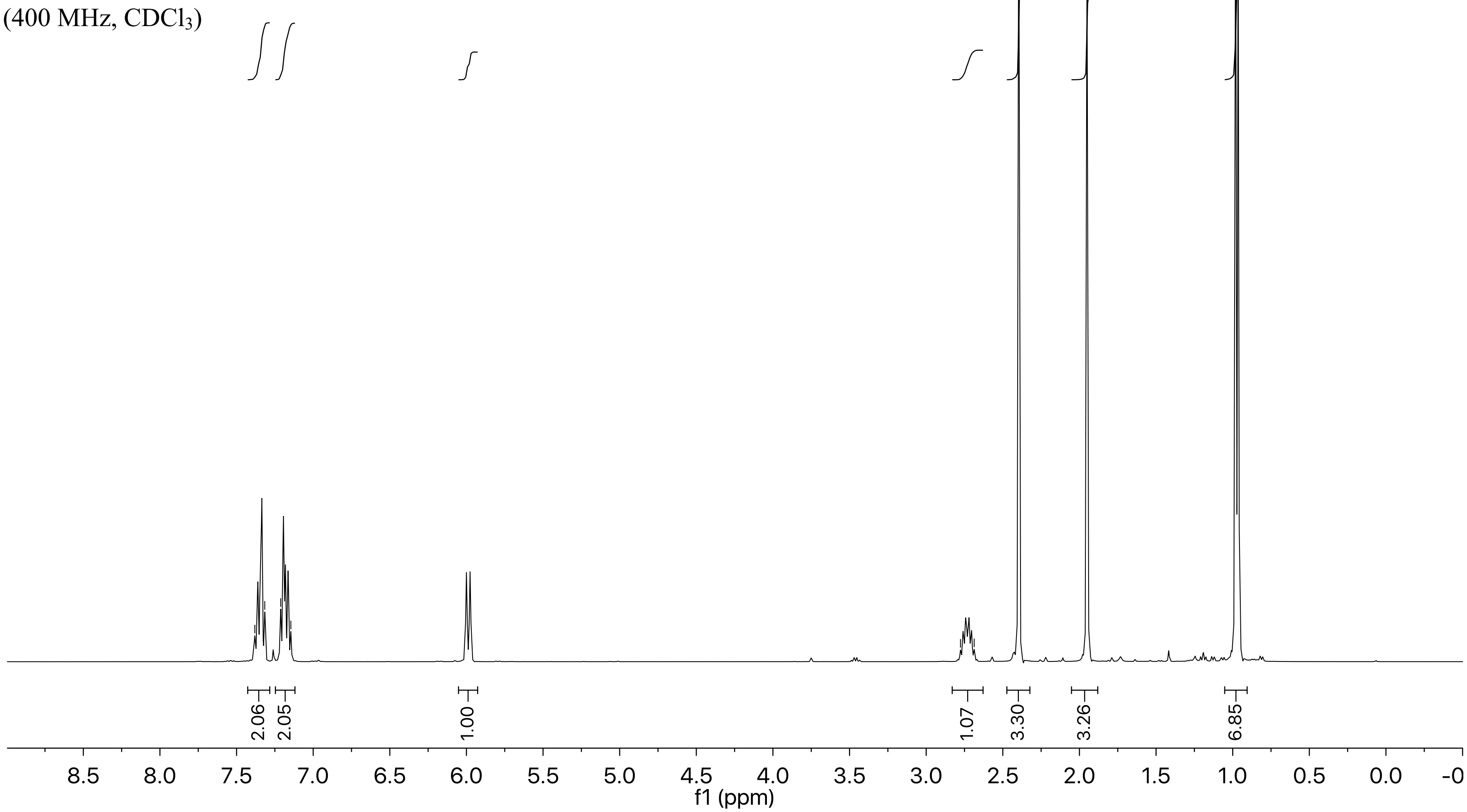

(*E*)-2,4-Dimethyl-1-(2-(methylthio)phenyl)pent-2-en-1-one, 3aa

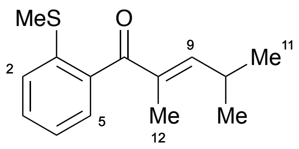

^{13}C NMR (101 MHz, $CDCl_3$)

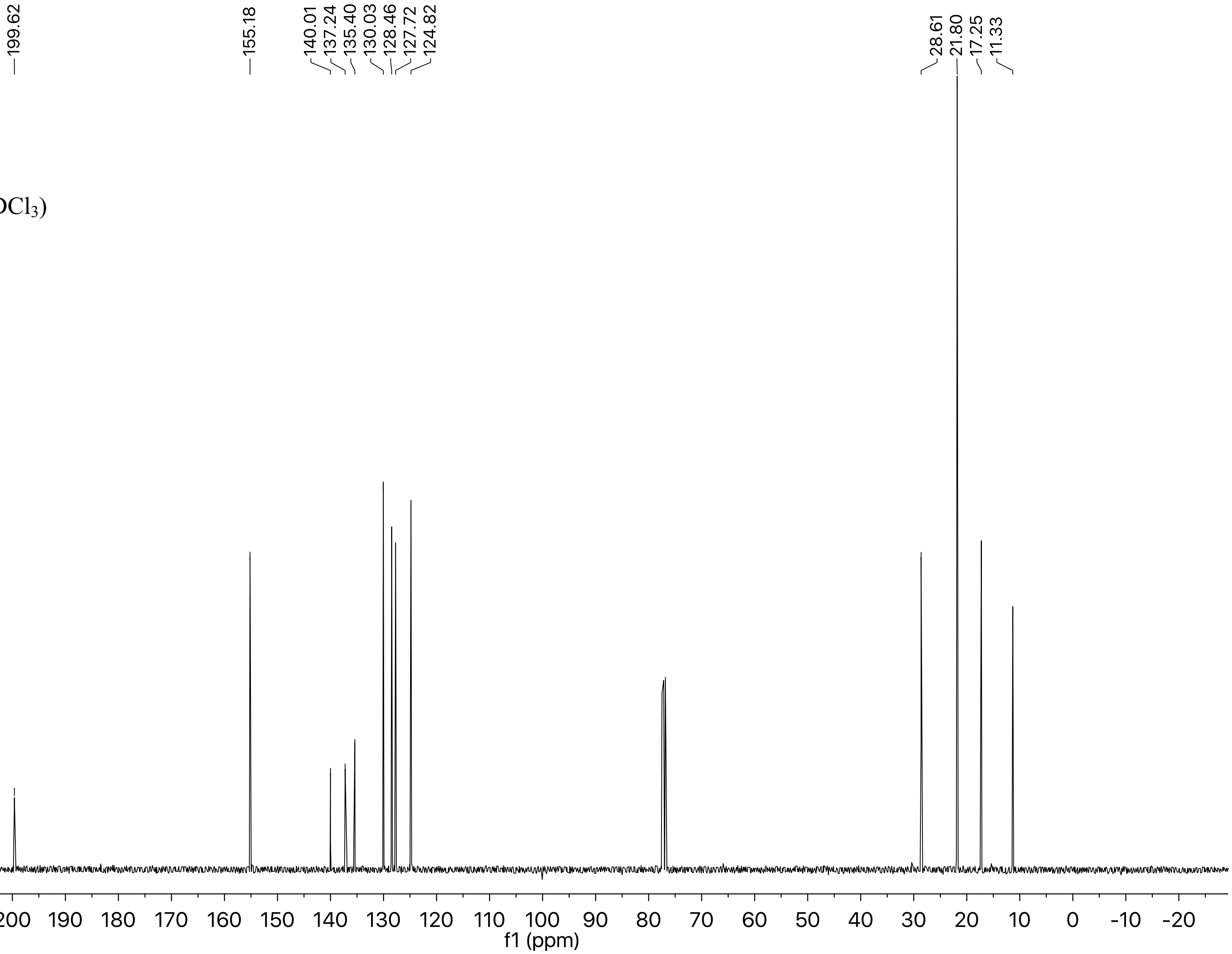

Benzyl (*E*)-(2-(2-(methylthio)benzoyl)oct-1-en-1-yl)(phenyl)carbamate, 3b

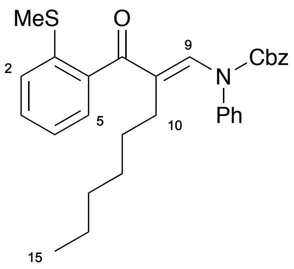

^{1}H NMR (400 MHz, $CDCl_3$)

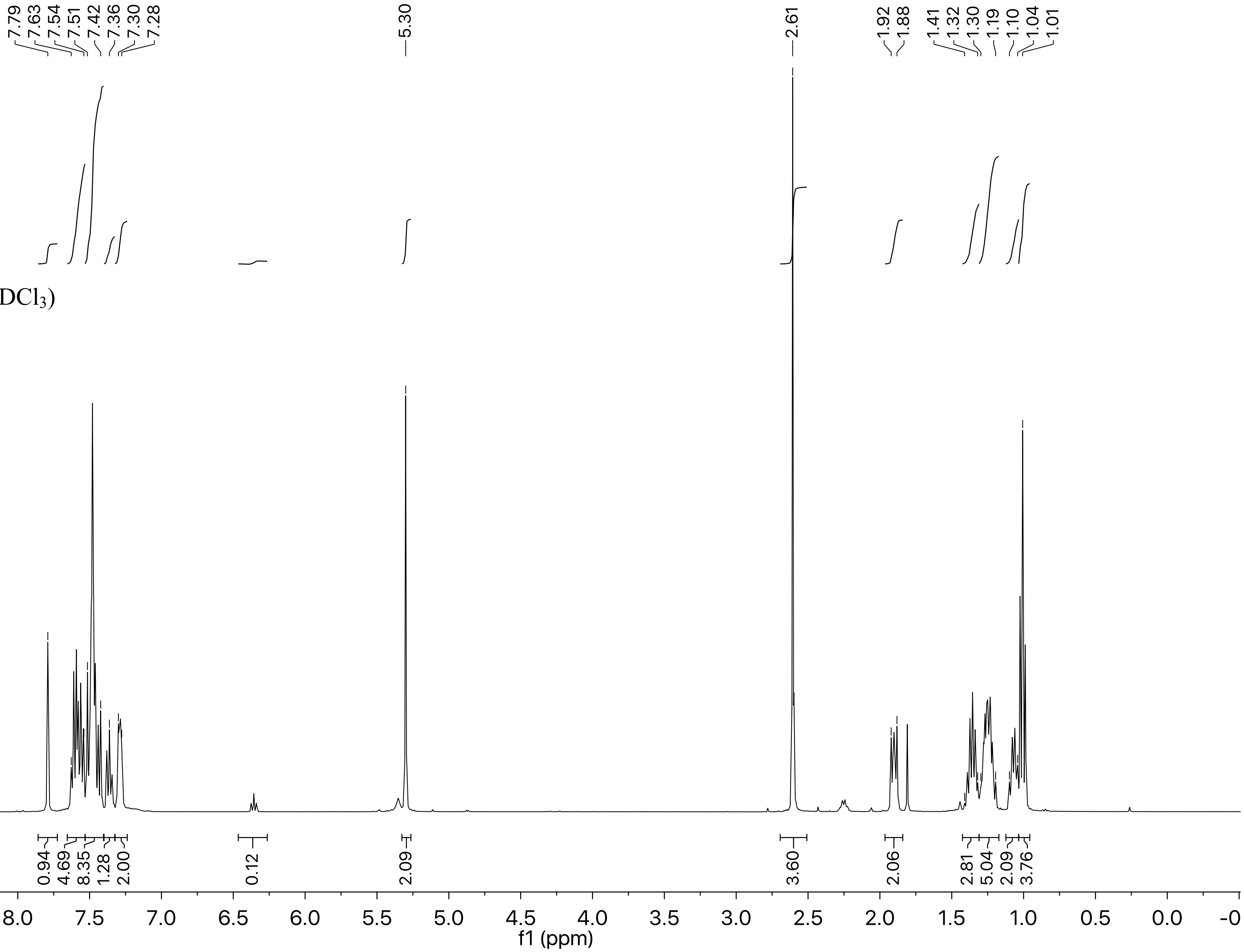

Benzyl (*E*)-(2-(2-(methylthio)benzoyl)oct-1-en-1-yl)(phenyl)carbamate, 3b

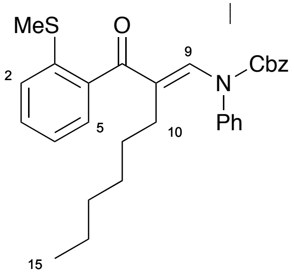

^{13}C NMR (101 MHz, $CDCl_3$)

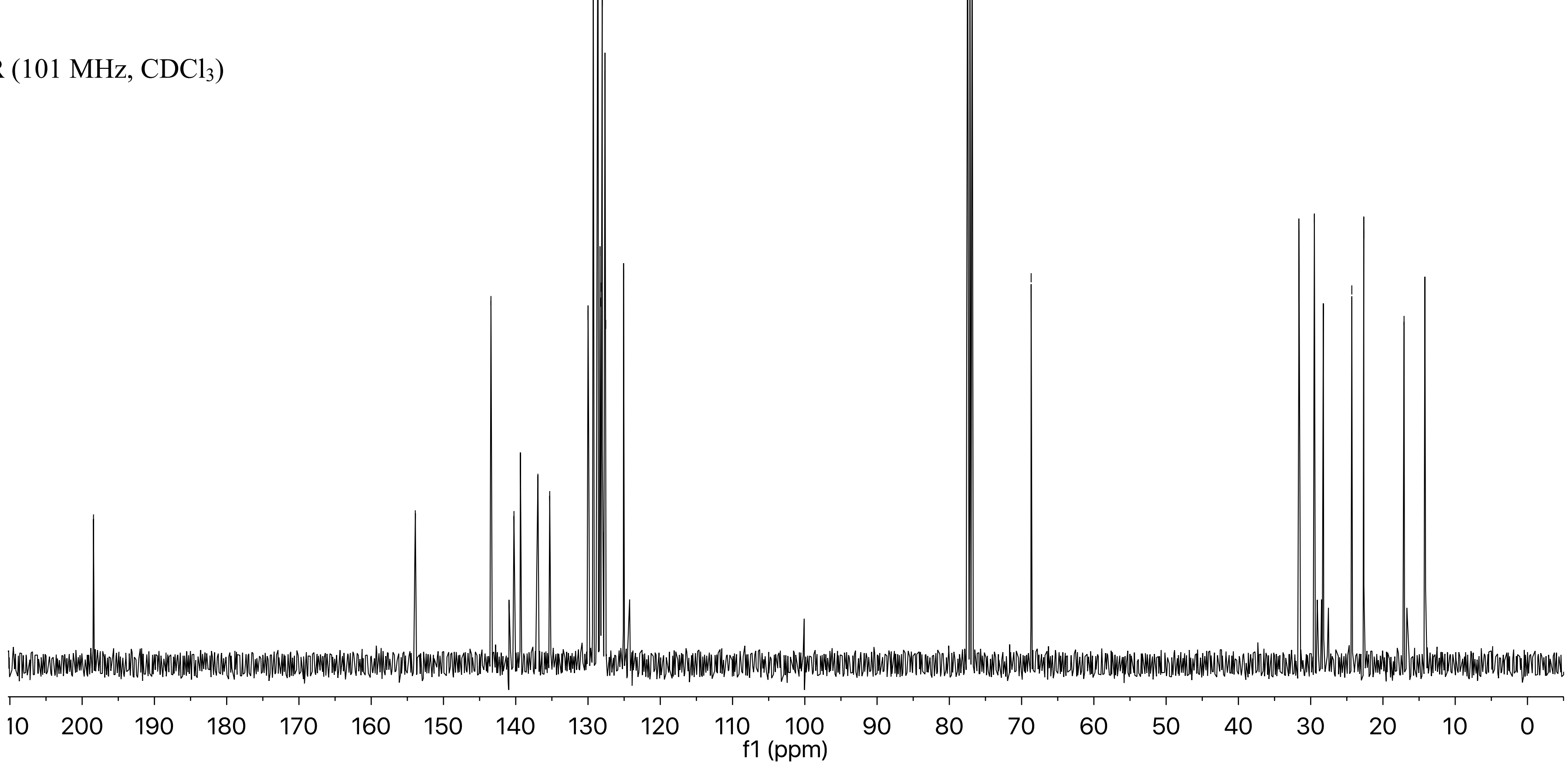

***tert*-Butyl (*E*)-(2-(2-(methylthio)benzoyl)oct-1-en-1-yl)(phenyl)carbamate, 3c**

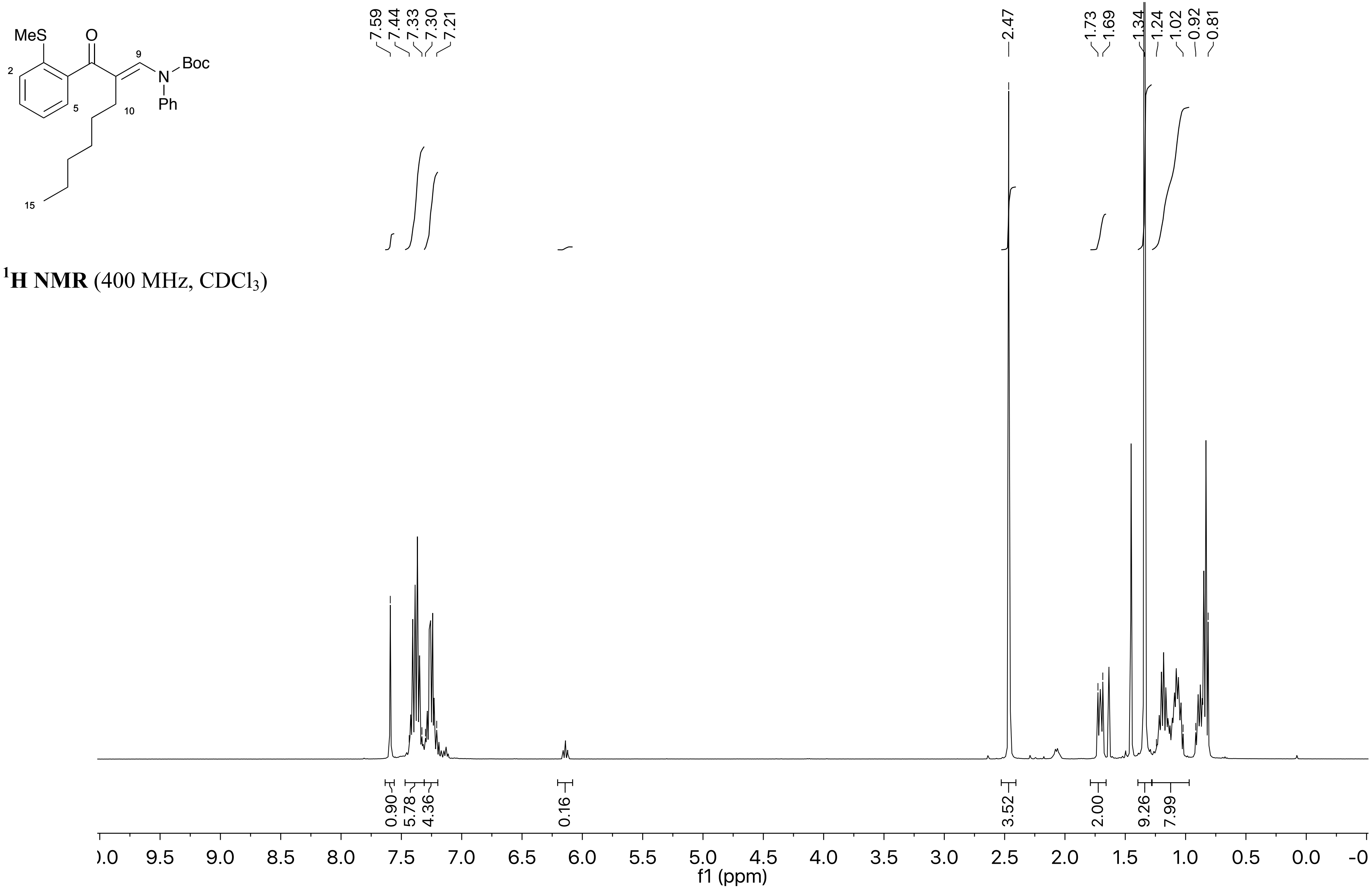

^{1}H NMR (400 MHz, $CDCl_3$)

***tert*-Butyl (*E*)-(2-(2-(methylthio)benzoyl)oct-1-en-1-yl)(phenyl)carbamate, 3c**

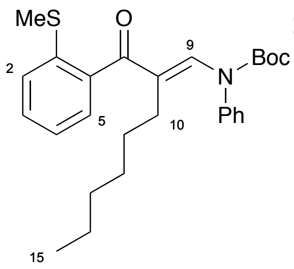

^{13}C NMR (101 MHz, $CDCl_3$)

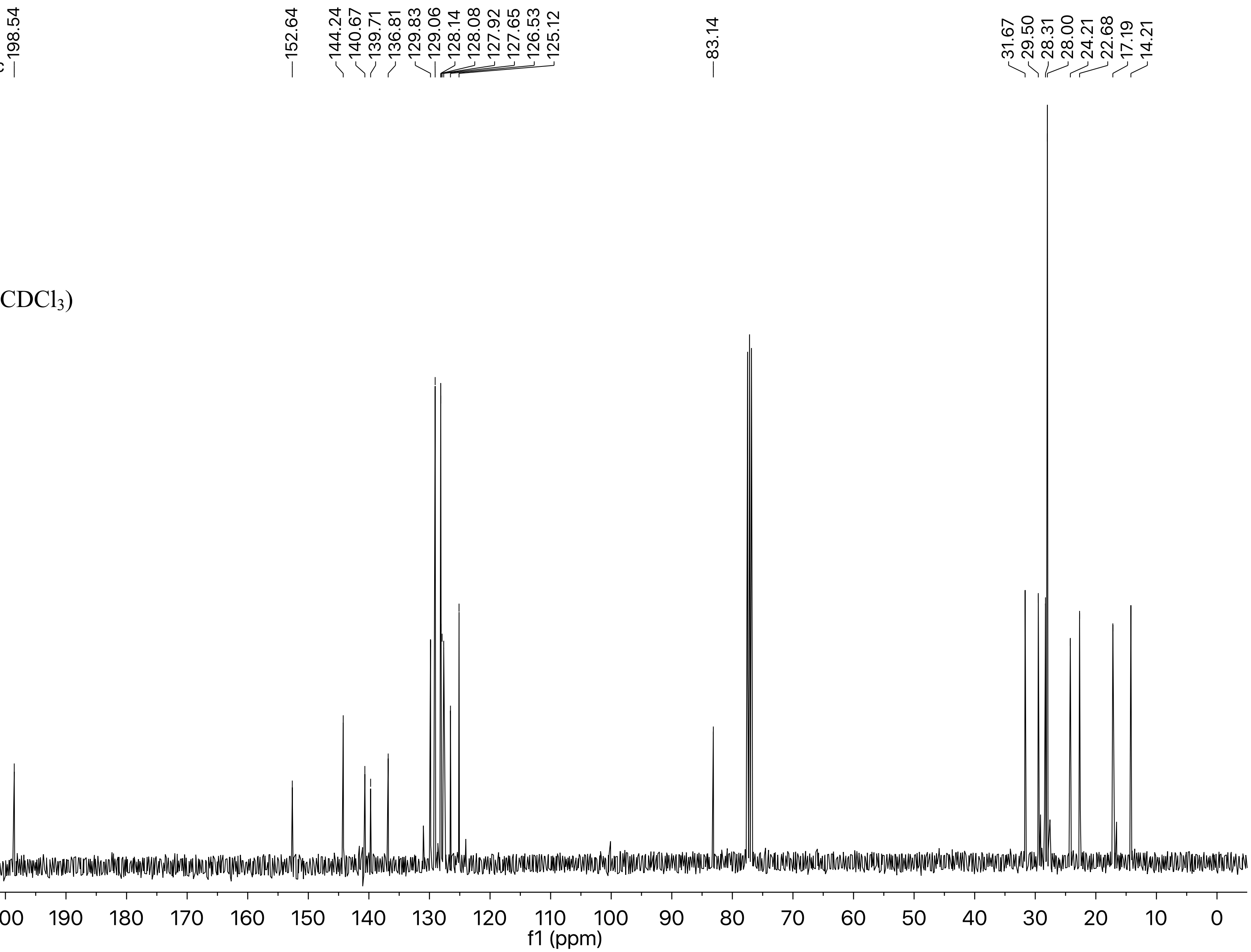

(*E*)-*N*-methyl-*N*-(2-(2-(methylthio)benzoyl)oct-1-en-1-yl)methanesulfonamide, 3d

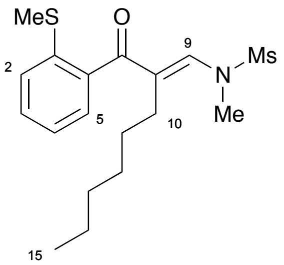

^{1}H NMR (400 MHz, $CDCl_3$)

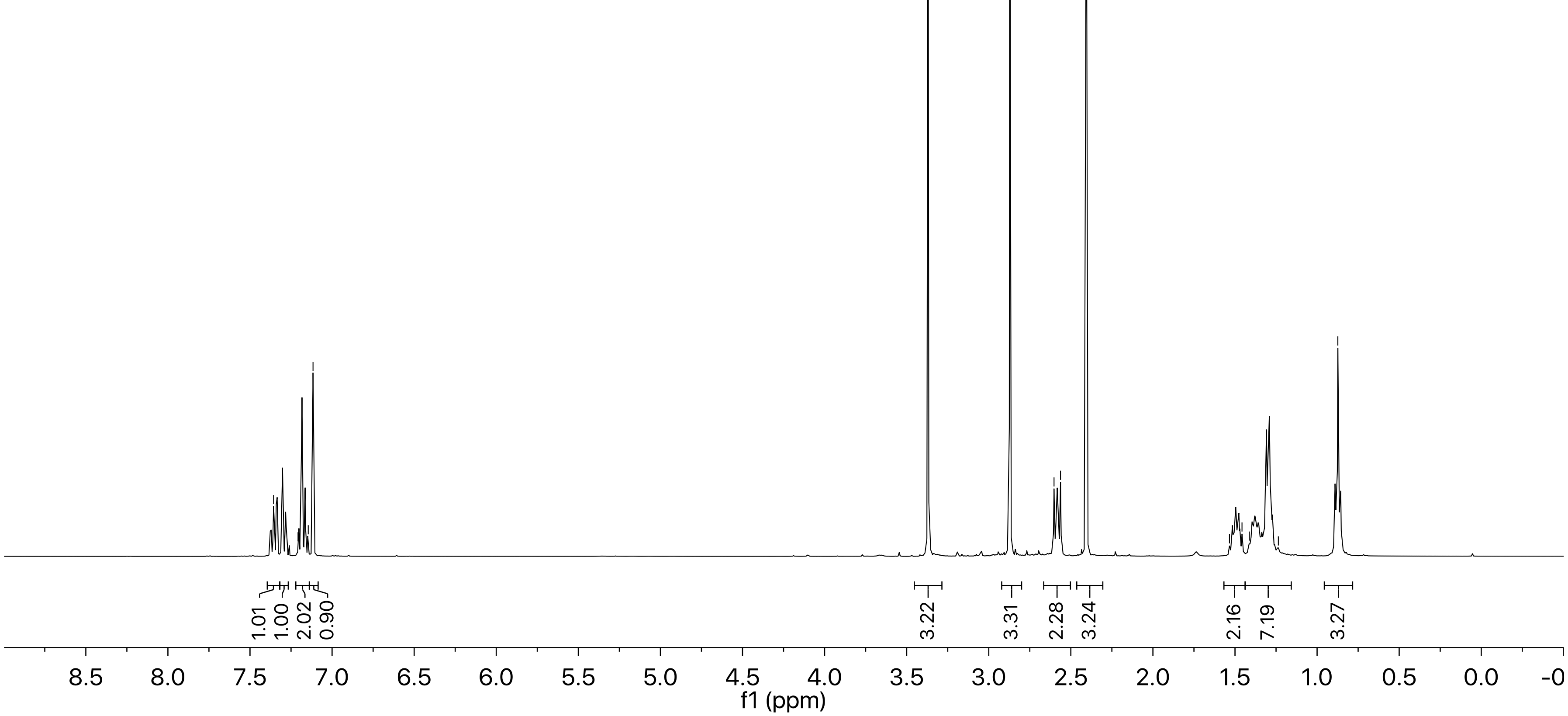

(*E*)-*N*-methyl-*N*-(2-(2-(methylthio)benzoyl)oct-1-en-1-yl)methanesulfonamide, 3d

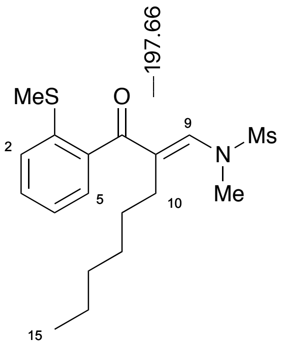

^{13}C NMR (101 MHz, $CDCl_3$)

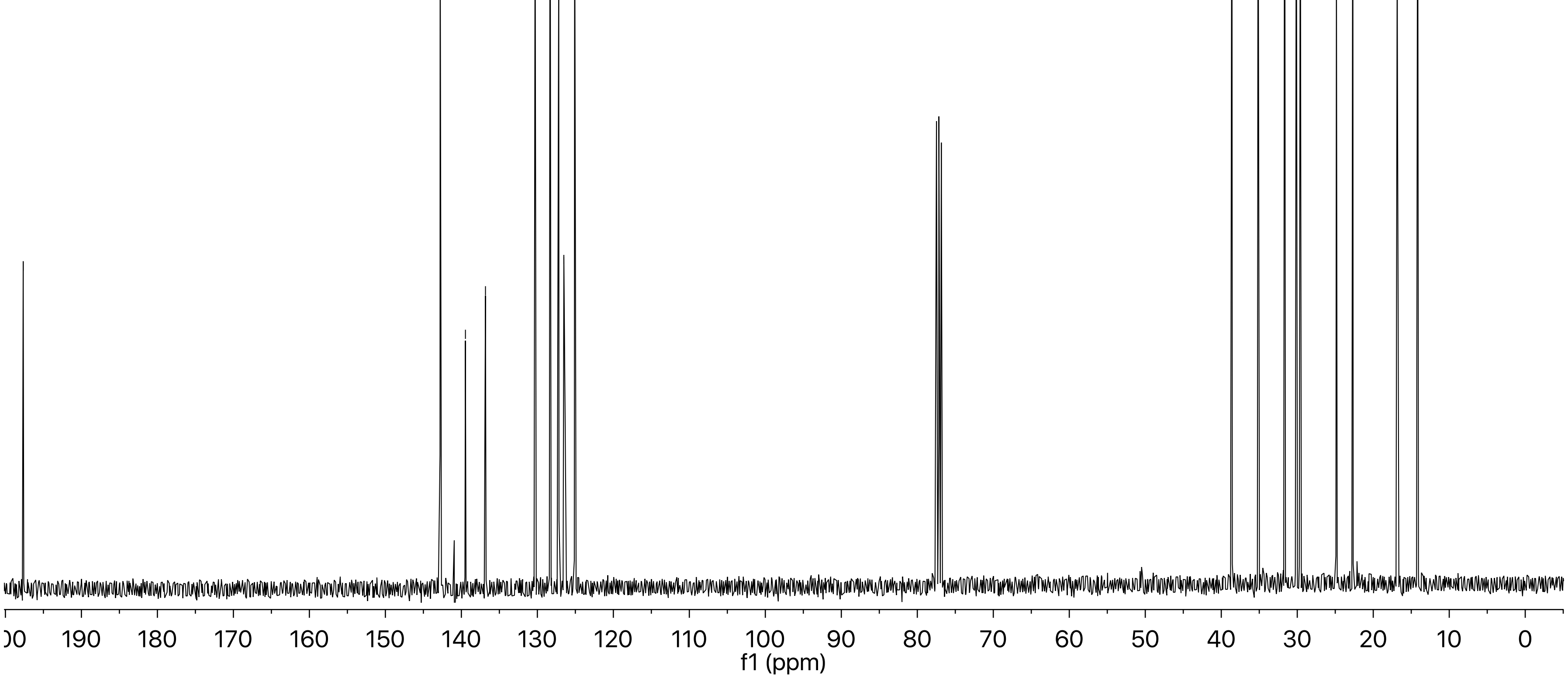

(*Z*)-*N*-Methyl-*N*-(1-(2-(methylthio)phenyl)-1-oxonon-2-en-2-yl)methanesulfonamide, 4d

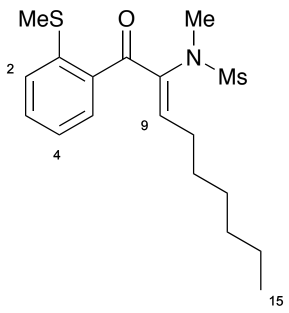

¹H NMR (400 MHz, $CDCl_3$)

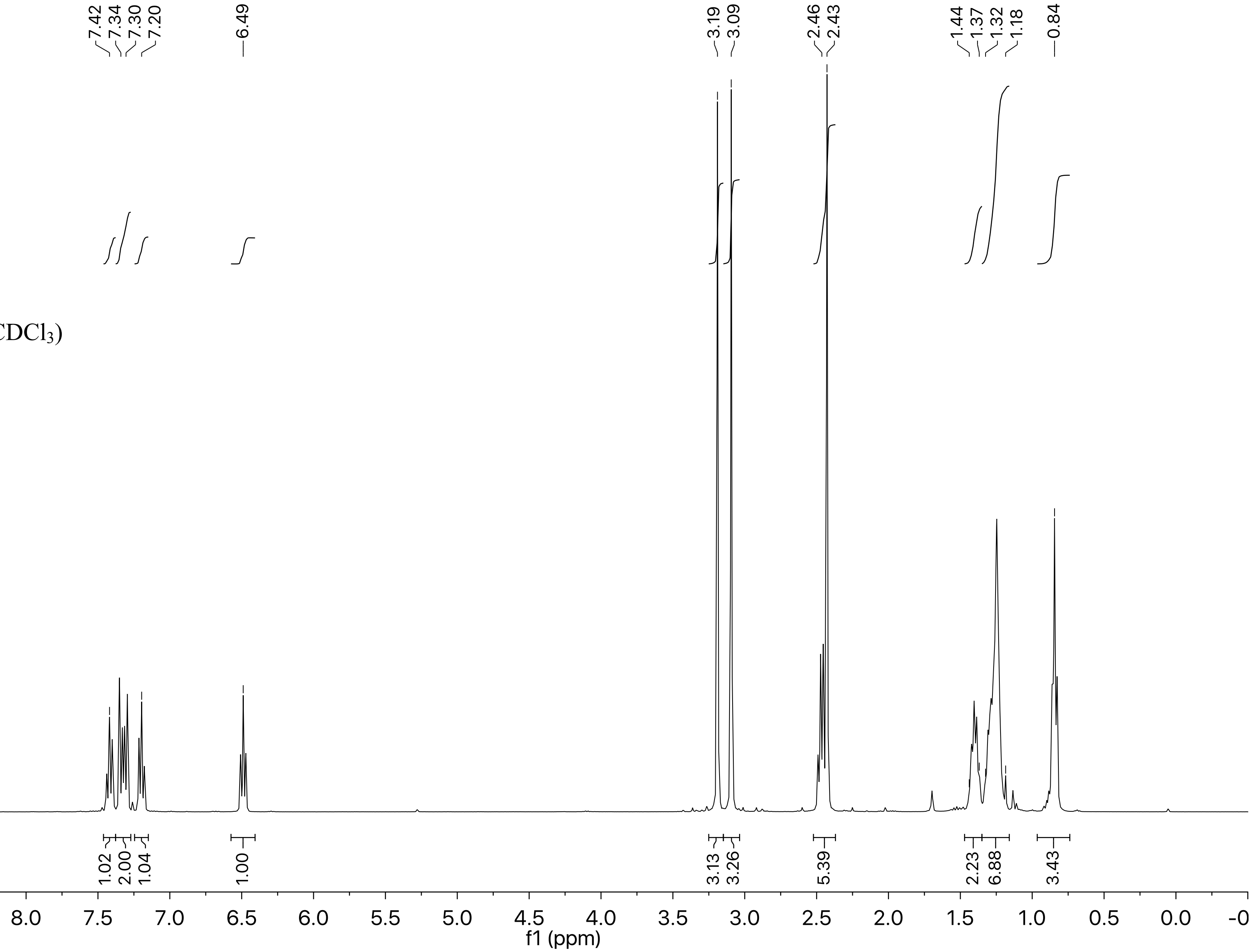

(*Z*)-*N*-Methyl-*N*-(1-(2-(methylthio)phenyl)-1-oxonon-2-en-2-yl)methanesulfonamide, 4d

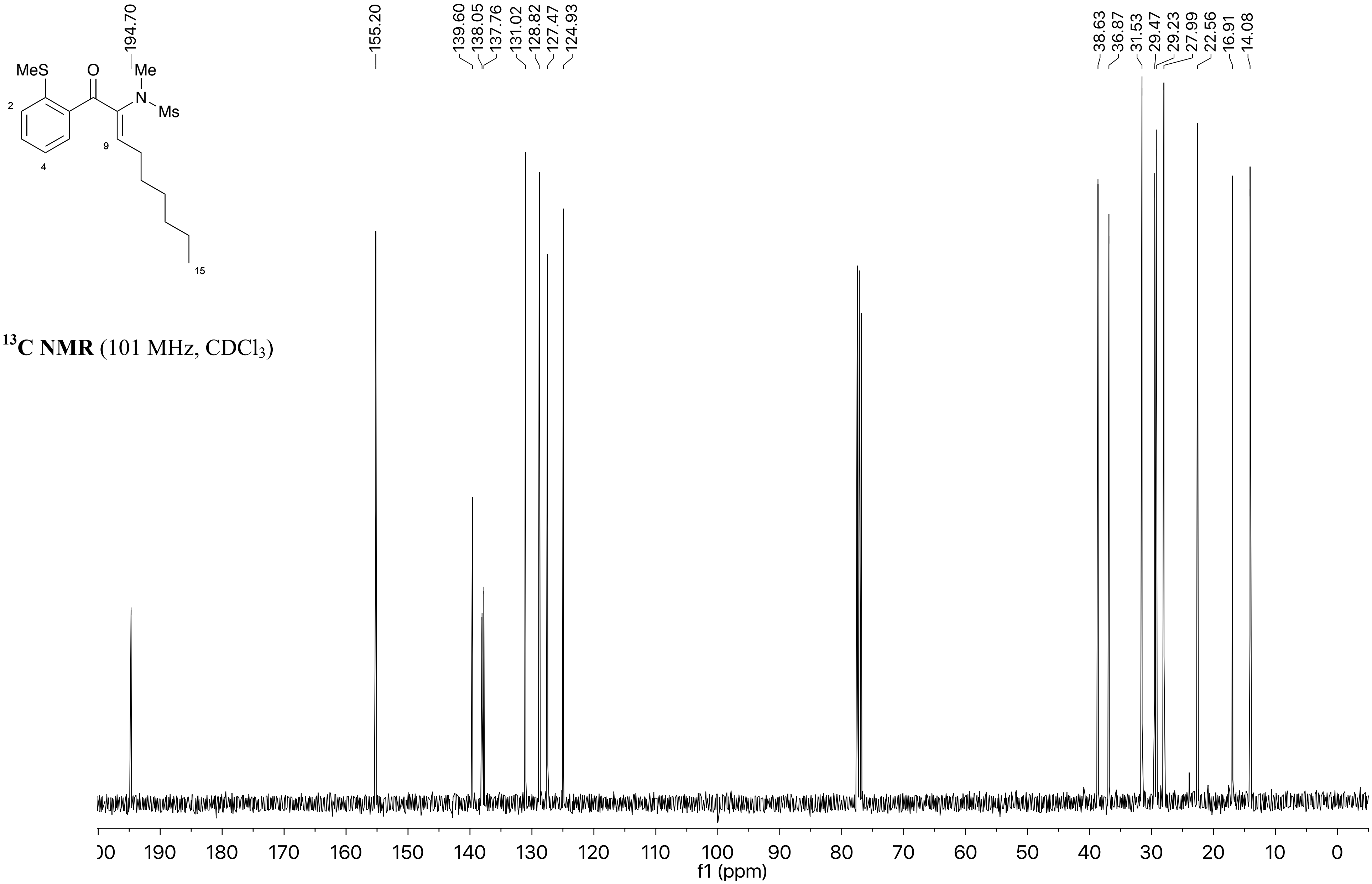

^{13}C NMR (101 MHz, $CDCl_3$)

(*E*)-*N*-(5-Chloro-2-(2-(methylthio)benzoyl)pent-1-en-1-yl)-4-methyl-*N*-phenylbenzenesulfonamide, 3e

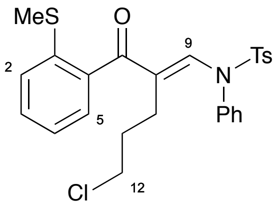

^{1}H NMR (400 MHz, $CDCl_3$)

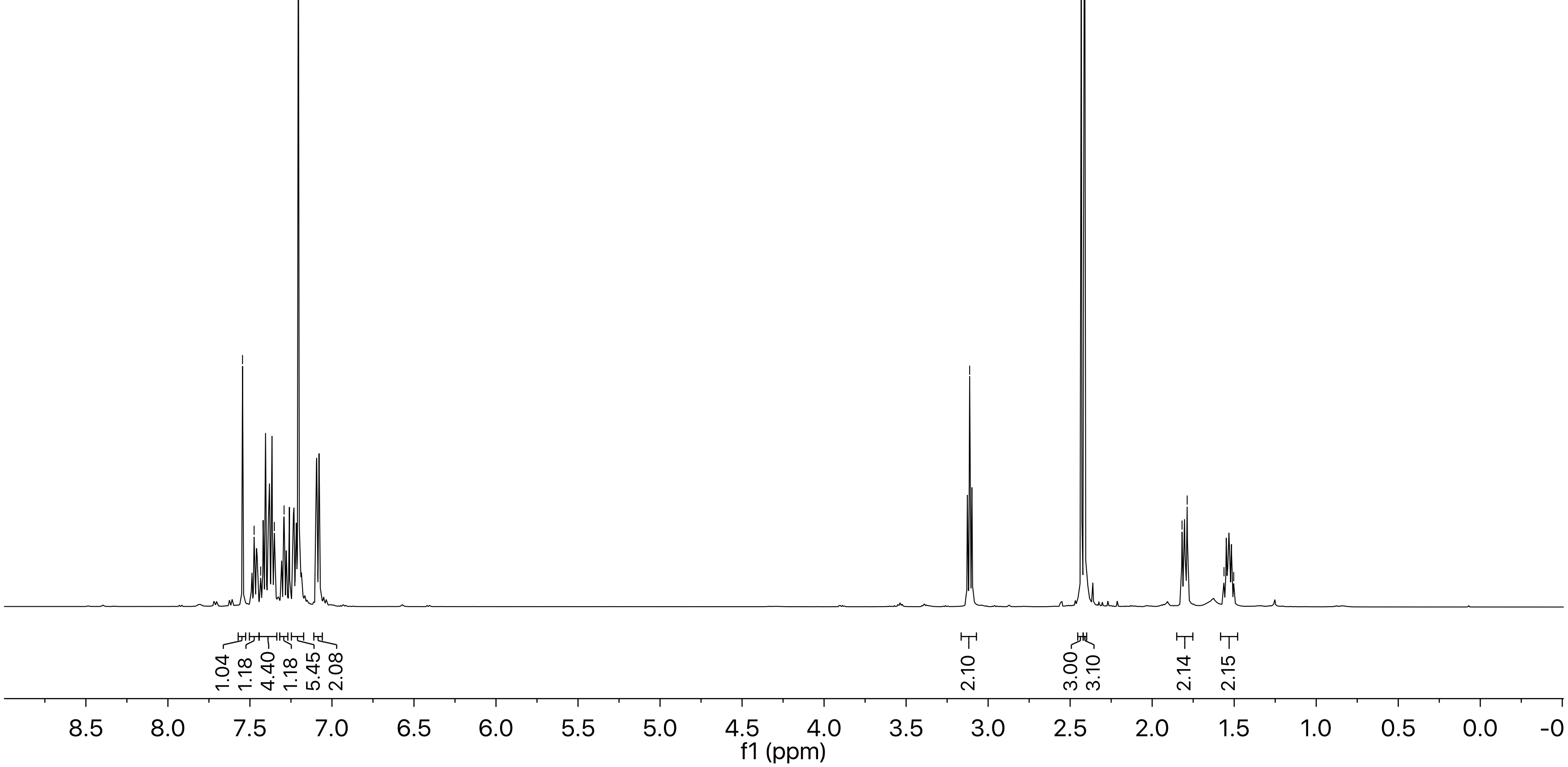

(*E*)-*N*-(5-Chloro-2-(2-(methylthio)benzoyl)pent-1-en-1-yl)-4-methyl-*N*-phenylbenzenesulfonamide, 3e

^{13}C NMR (101 MHz, $CDCl_3$)

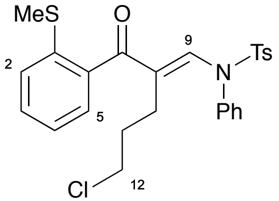

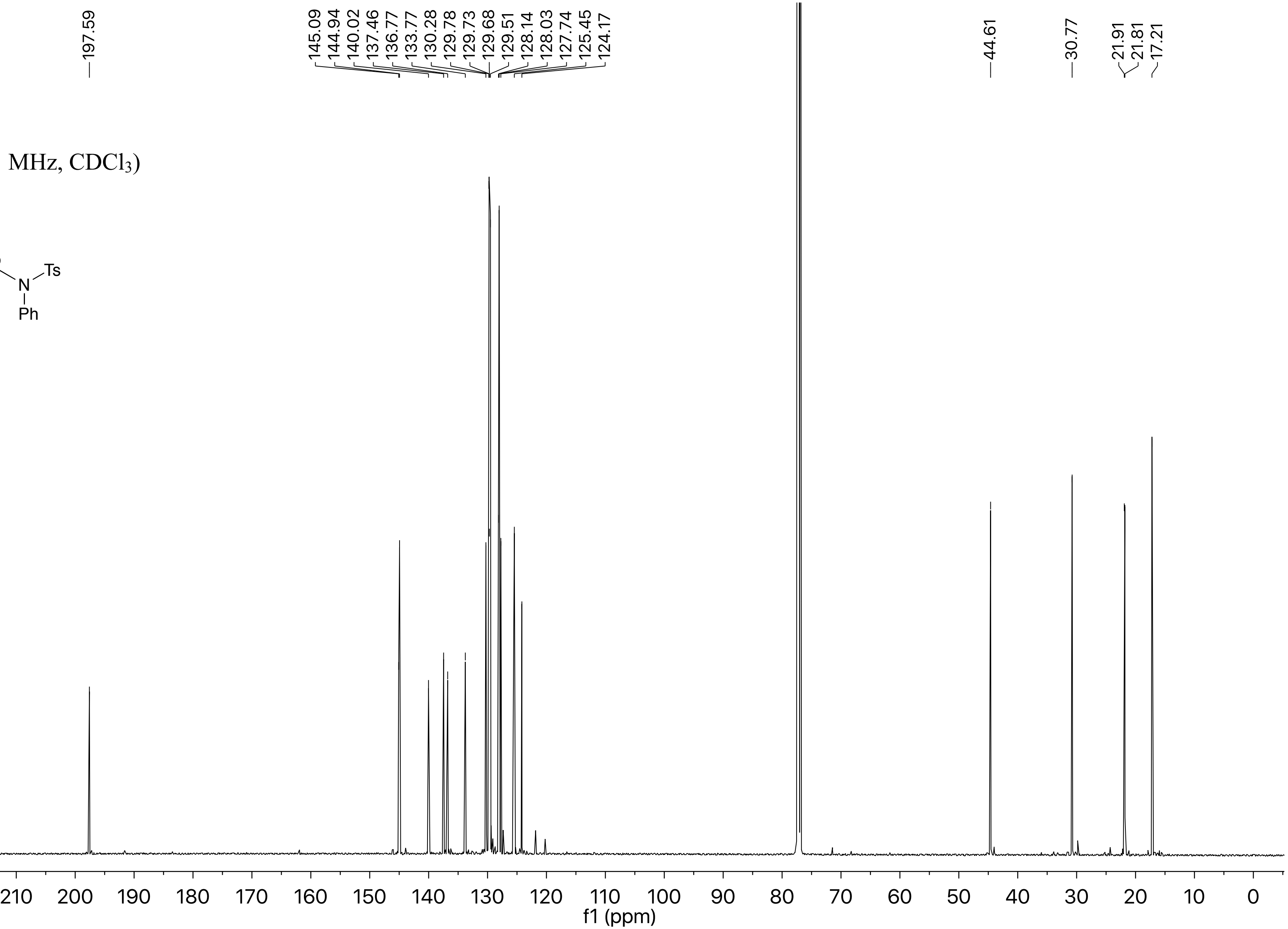

(*Z*)-*N*-(6-Chloro-1-(2-(methylthio)phenyl)-1-oxohex-2-en-2-yl)-4-methyl-*N*-phenylbenzenesulfonamide, 4e

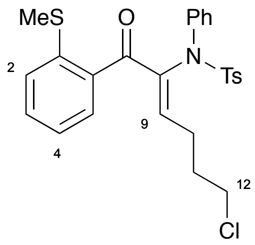

^{1}H NMR (400 MHz, $CDCl_3$)

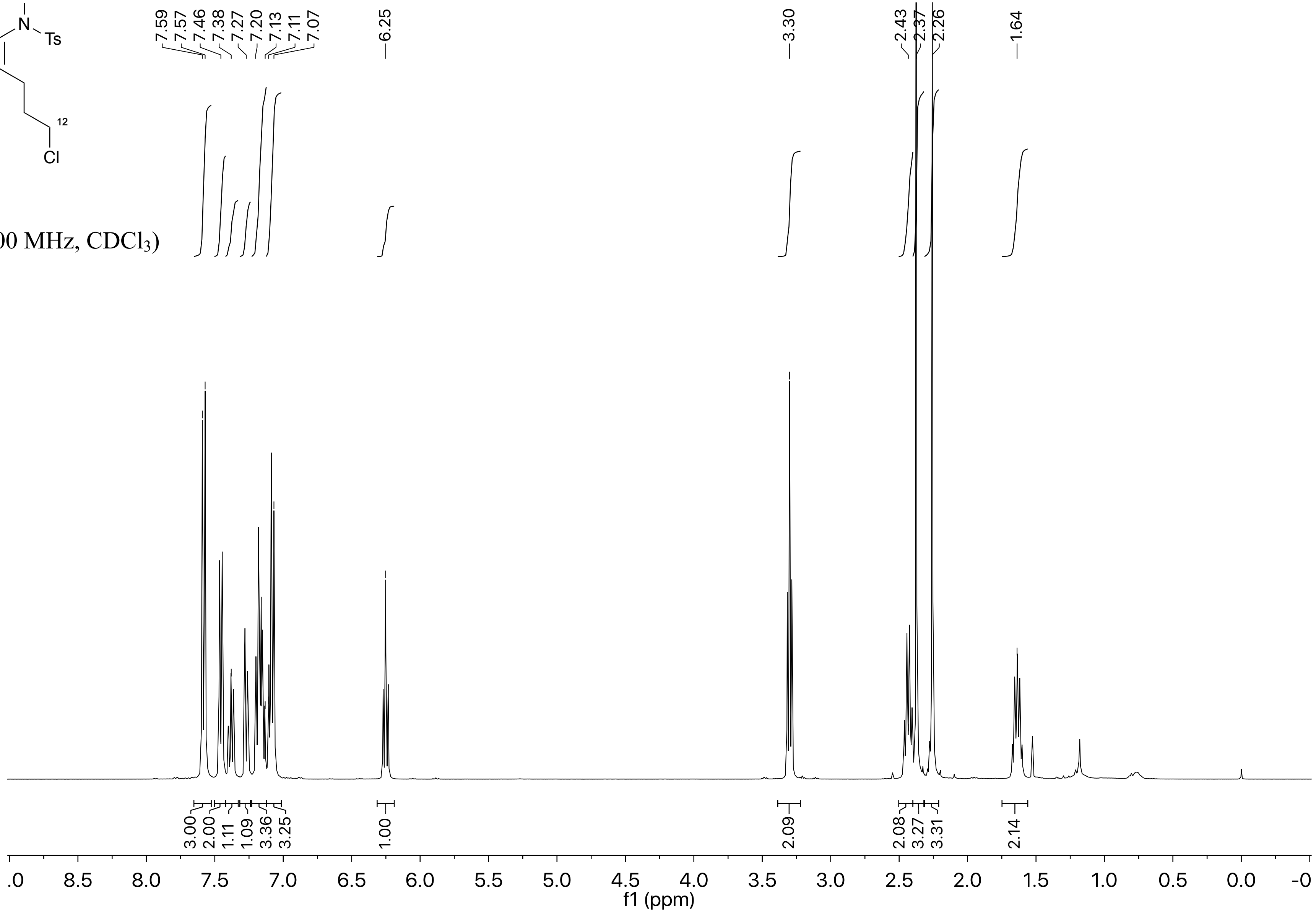

(*Z*)-*N*-(6-Chloro-1-(2-(methylthio)phenyl)-1-oxohex-2-en-2-yl)-4-methyl-*N*-phenylbenzenesulfonamide, 4e

^{13}C NMR (101 MHz, $CDCl_3$)

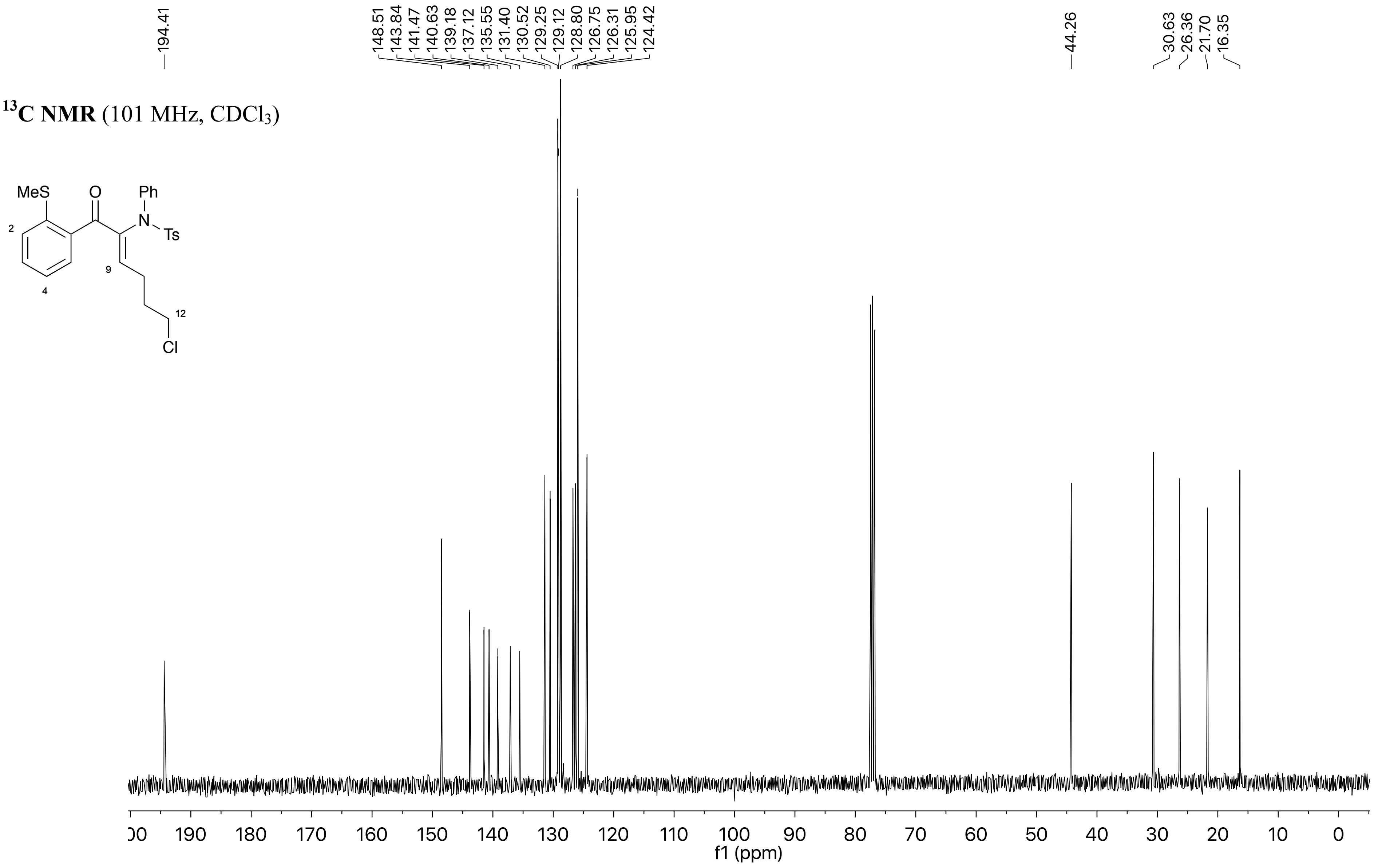

(*E*)-*N*-(5-((*tert*-Butyldimethylsilyl)oxy)-2-(2-(methylthio)benzoyl)pent-1-en-1-yl)-4-methyl-*N*-phenylbenzenesulfonamide, 3f

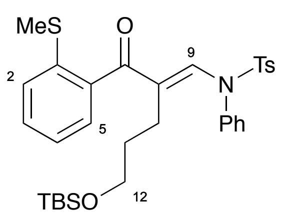

^{1}H NMR (400 MHz, $CDCl_3$)

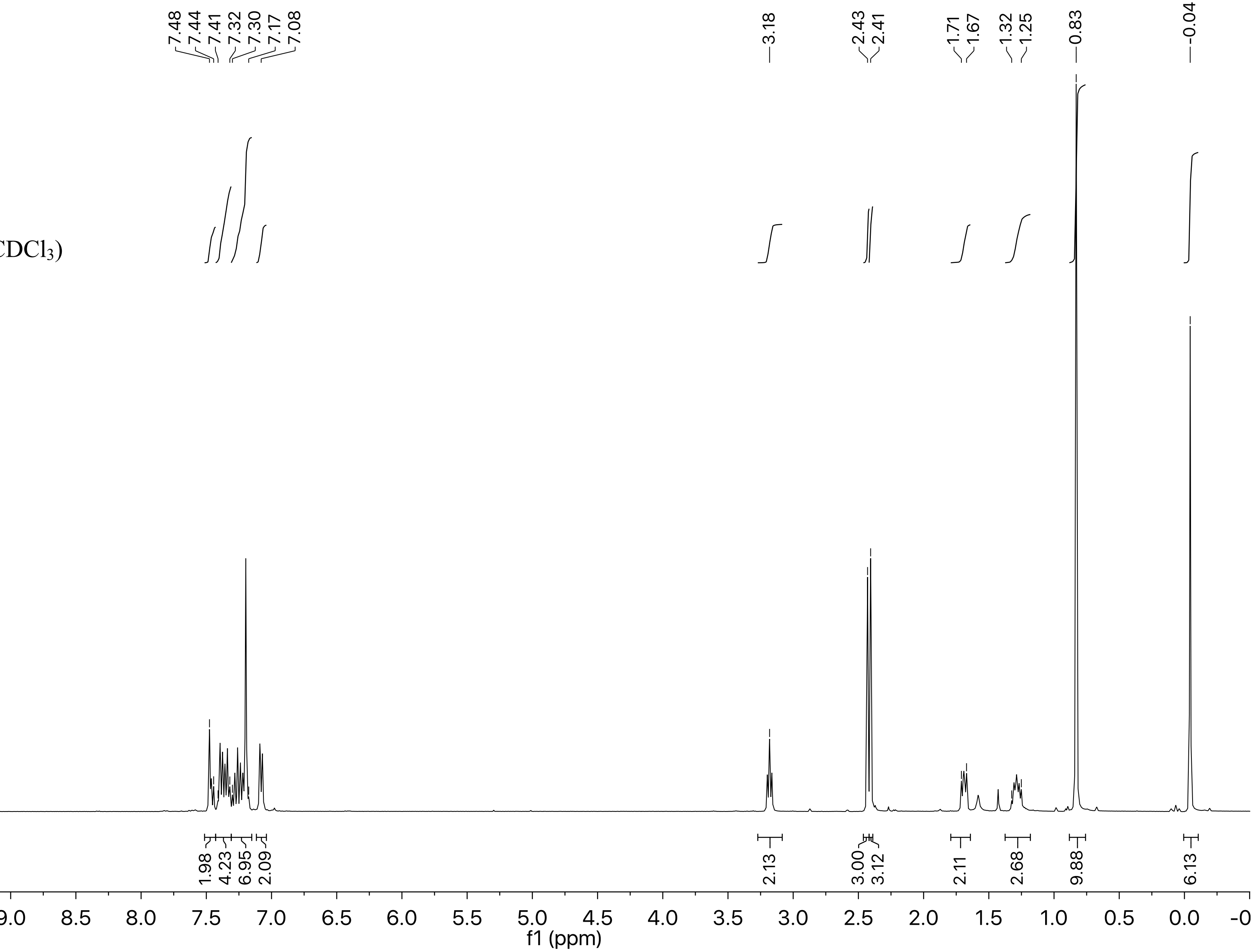

(*E*)-*N*-(5-((*tert*-Butyldimethylsilyl)oxy)-2-(2-(methylthio)benzoyl)pent-1-en-1-yl)-4-methyl-*N*-phenylbenzenesulfonamide, 3f

^{13}**C NMR** (101 MHz, $CDCl_3$)

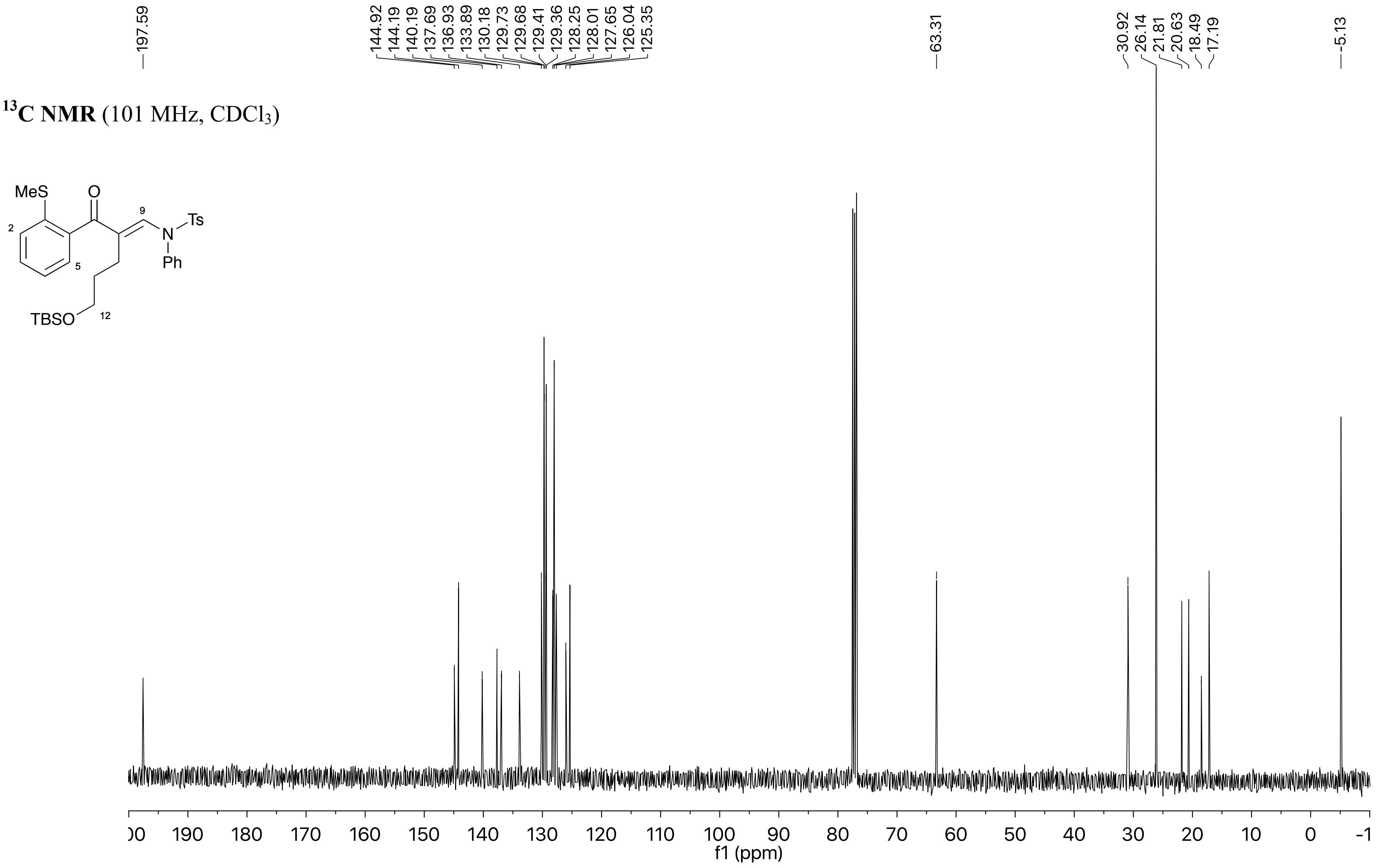

(*Z*)-*N*-(6-((*tert*-Butyldimethylsilyl)oxy)-1-(2-(methylthio)phenyl)-1-oxohex-2-en-2-yl)-4-methyl-*N*-phenylbenzenesulfonamide, 4f

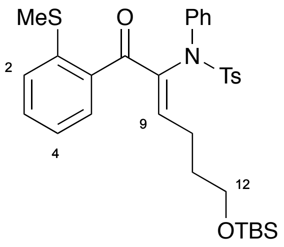

^{1}H NMR (400 MHz, $CDCl_3$)

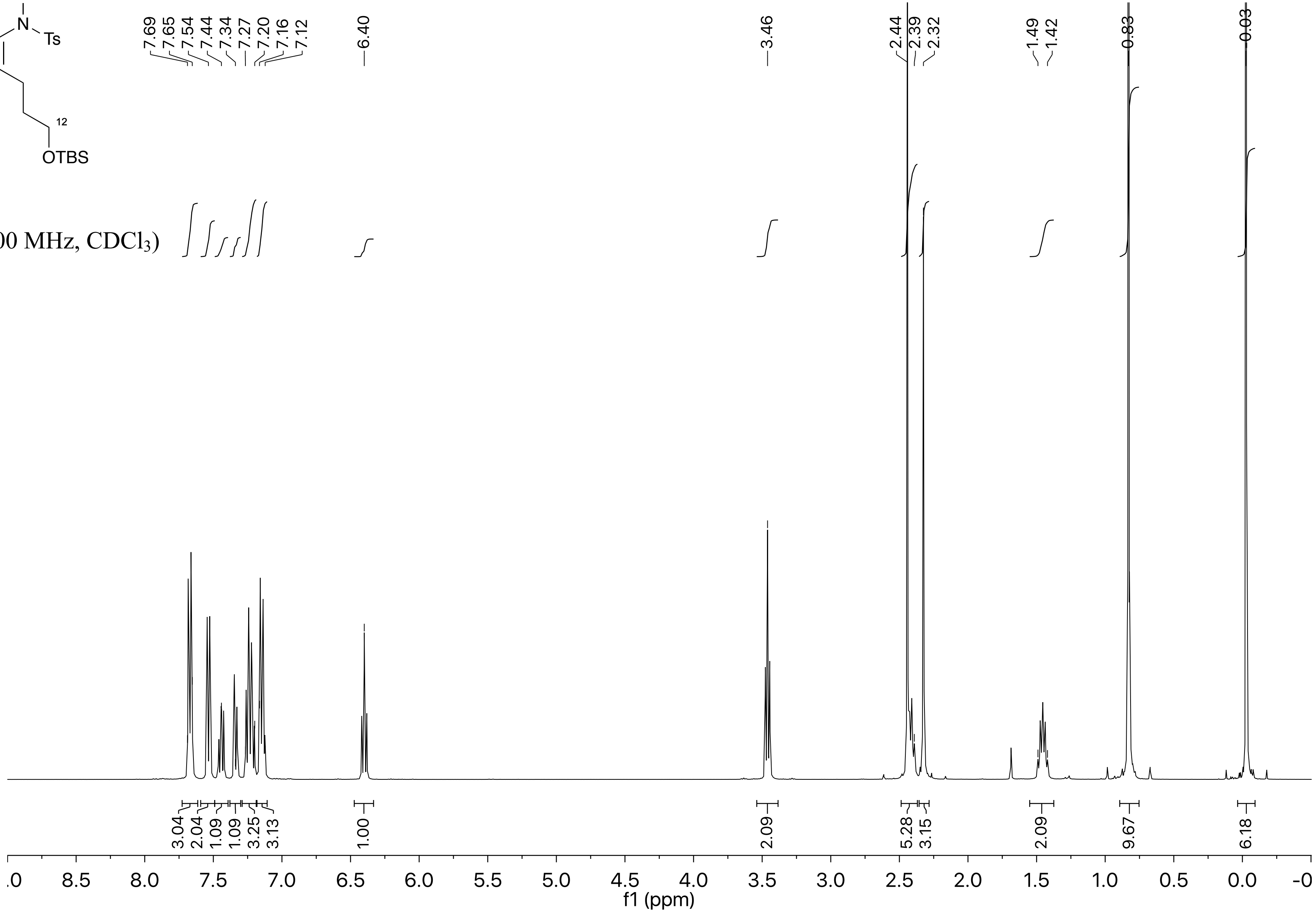

(*Z*)-*N*-(6-((*tert*-Butyldimethylsilyl)oxy)-1-(2-(methylthio)phenyl)-1-oxohex-2-en-2-yl)-4-methyl-*N*-phenylbenzenesulfonamide, 4f

^{13}C NMR (101 MHz, $CDCl_3$)

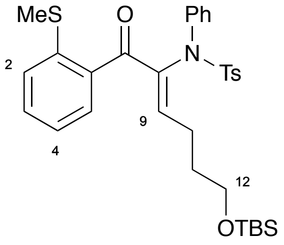

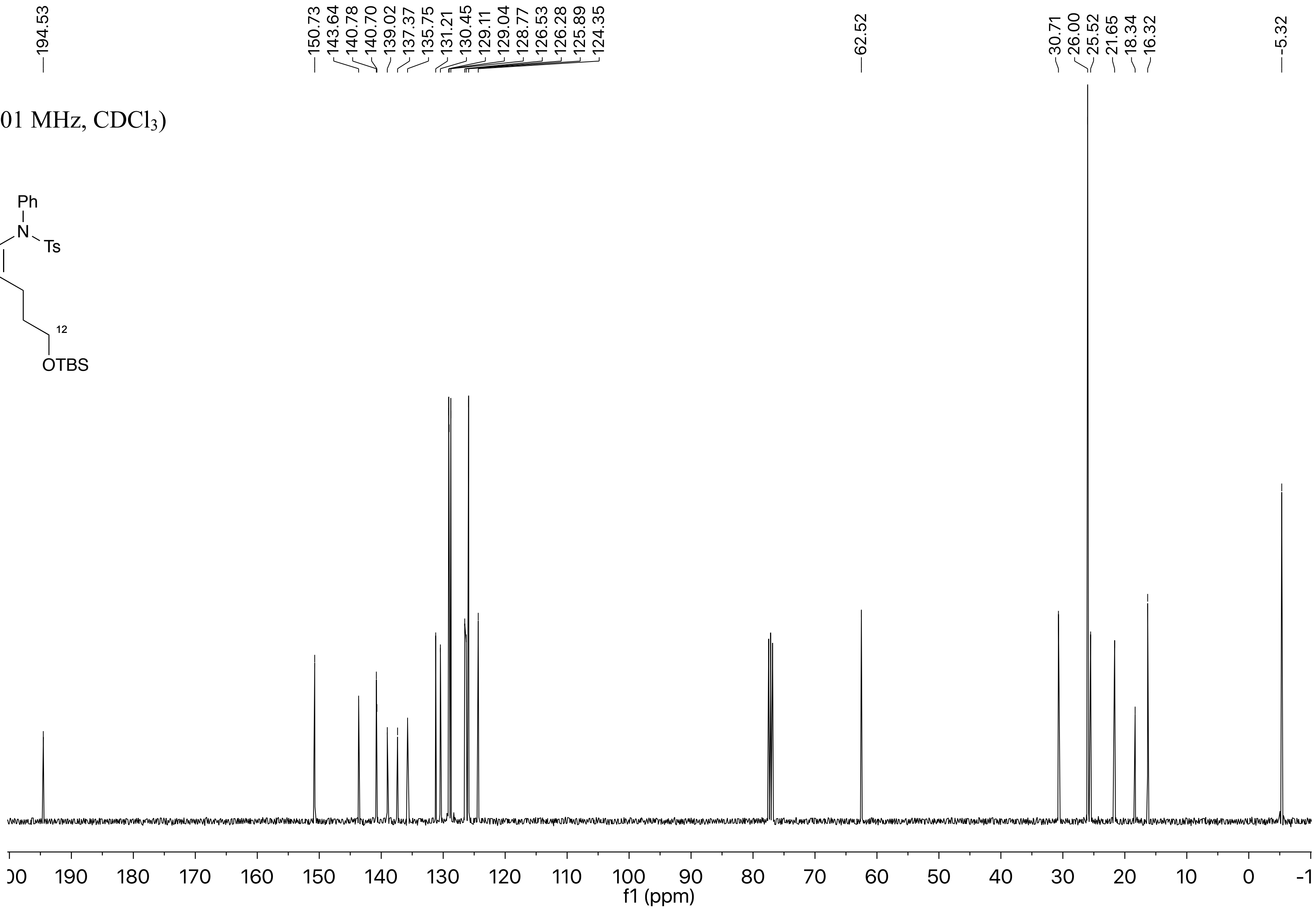

(*E*)-4-Methyl-*N*-(3-(2-(methylthio)phenyl)-3-oxo-2-phenylprop-1-en-1-yl)-*N*-phenylbenzenesulfonamide, 3g

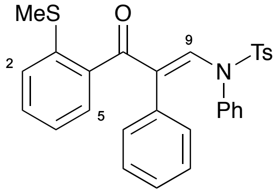

^{1}H NMR (400 MHz, $CDCl_3$)

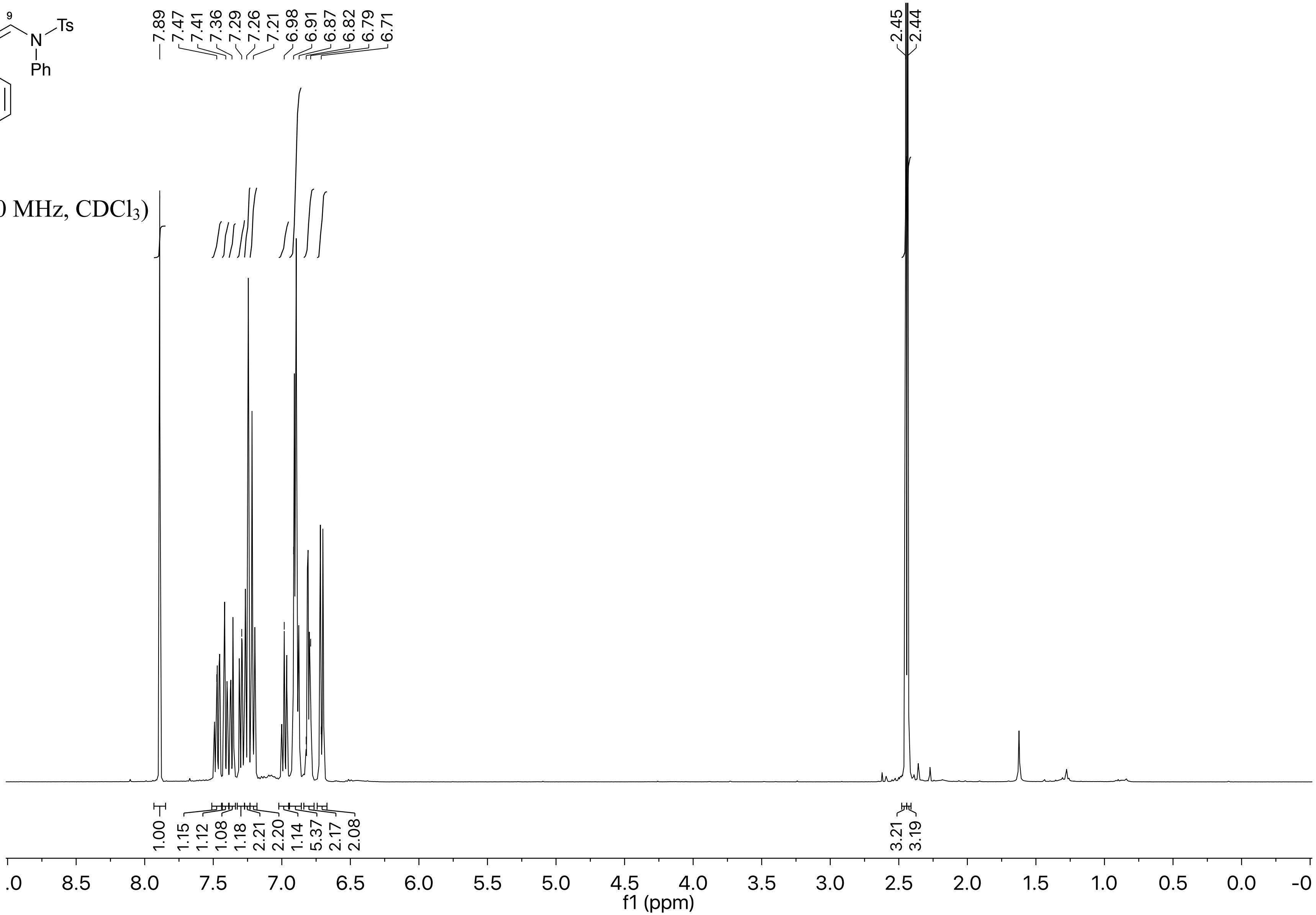

(*E*)-4-Methyl-*N*-(3-(2-(methylthio)phenyl)-3-oxo-2-phenylprop-1-en-1-yl)-*N*-phenylbenzenesulfonamide, 3g

^{13}C NMR (101 MHz, $CDCl_3$)

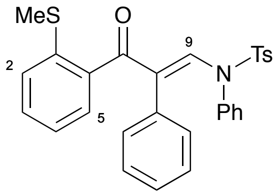

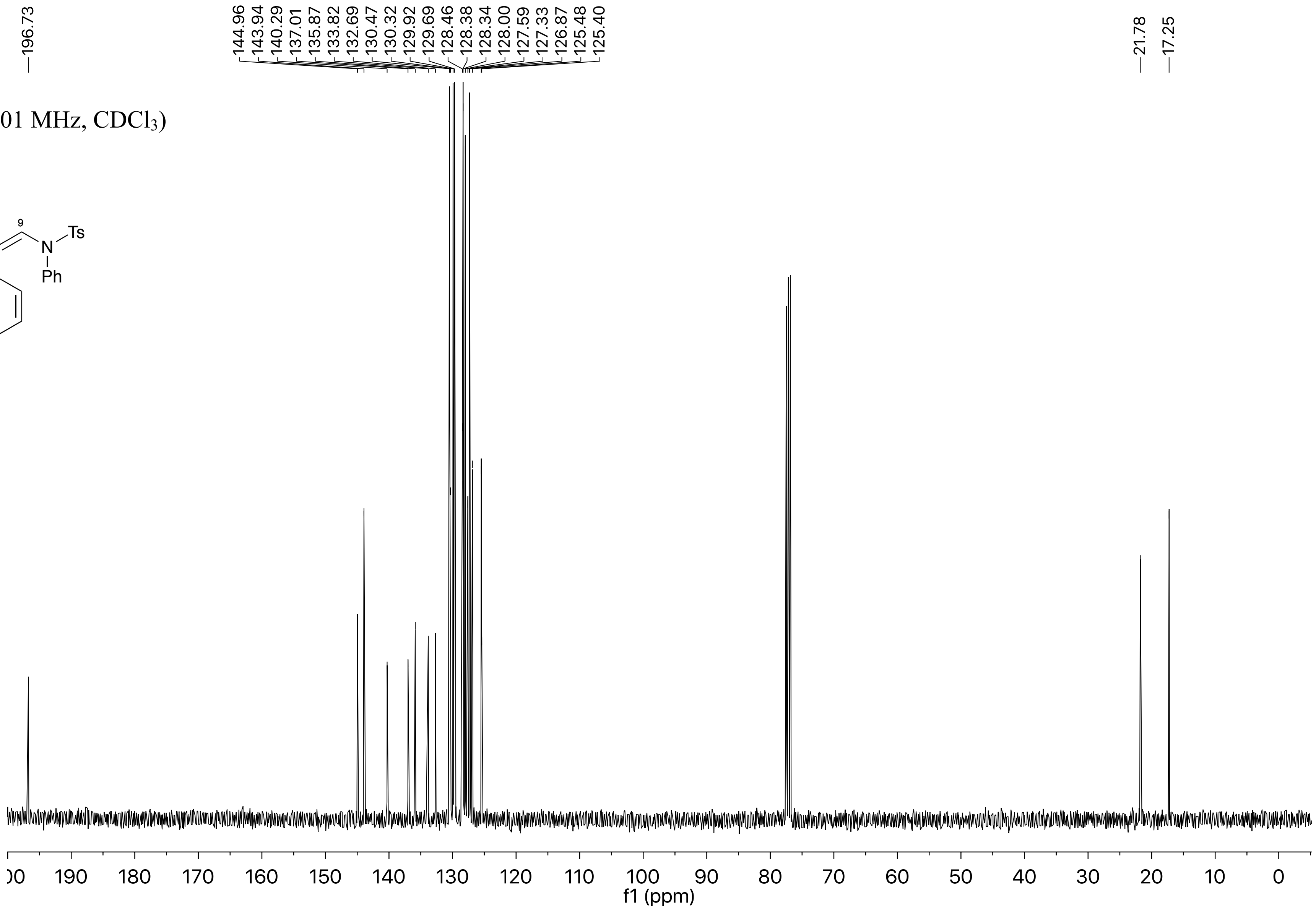

(*Z*)-4-Methyl-*N*-(3-(2-(methylthio)phenyl)-3-oxo-1-phenylprop-1-en-2-yl)-*N*-phenylbenzenesulfonamide, 4g

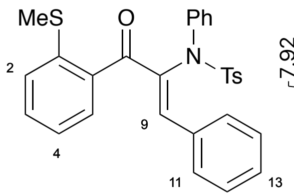

^{1}H NMR (400 MHz, $CDCl_3$)

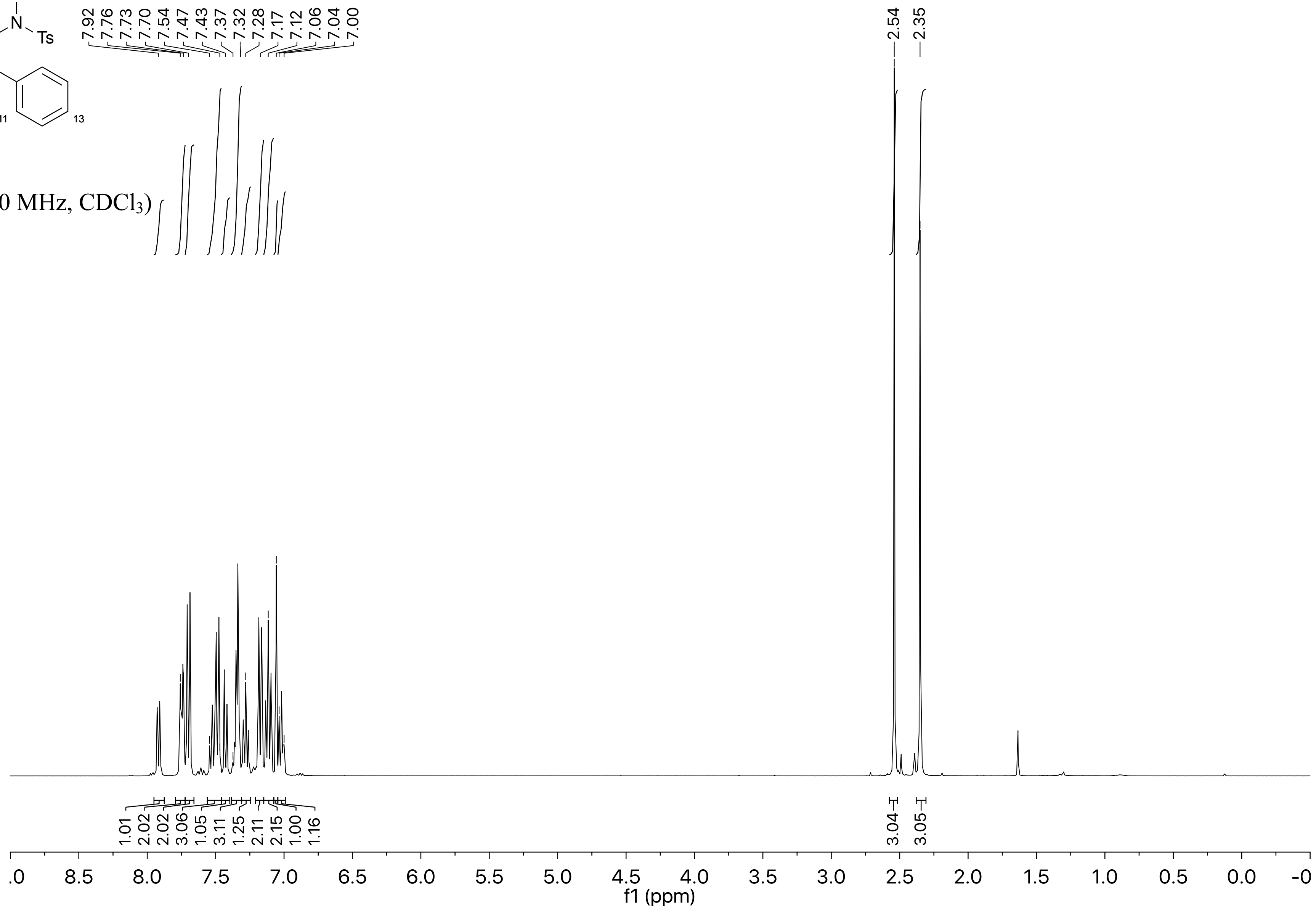

(*Z*)-4-Methyl-*N*-(3-(2-(methylthio)phenyl)-3-oxo-1-phenylprop-1-en-2-yl)-*N*-phenylbenzenesulfonamide, 4g

^{13}C NMR (101 MHz, $CDCl_3$)

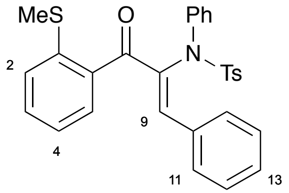

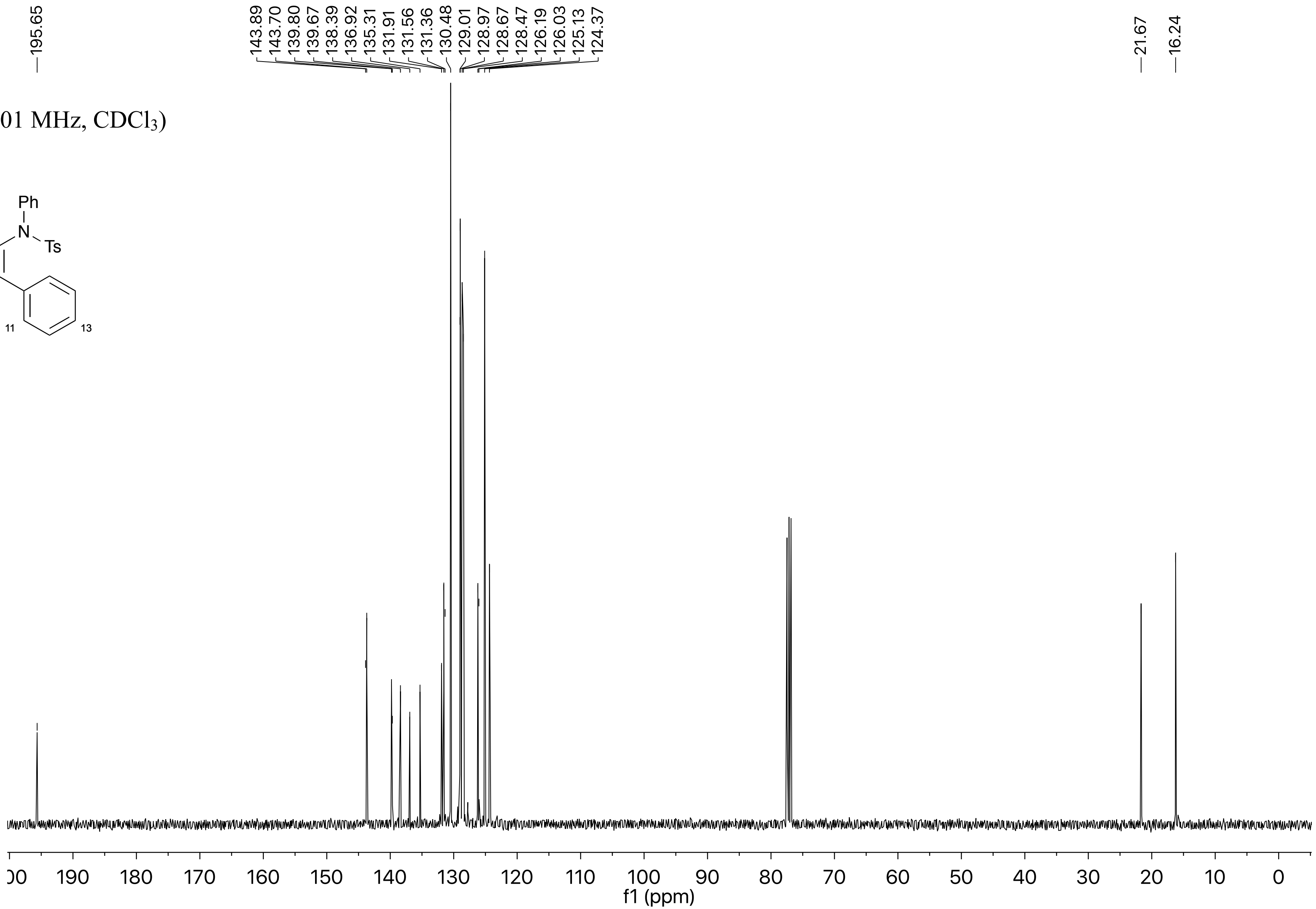

(*E*)-*N*-(2-(4-Methoxyphenyl)-3-(2-(methylthio)phenyl)-3-oxoprop-1-en-1-yl)-4-methyl-*N*-phenylbenzenesulfonamide, 3h

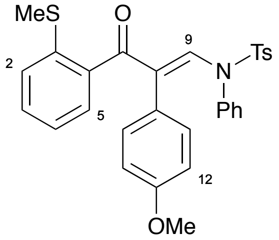

^{1}H NMR (400 MHz, $CDCl_3$)

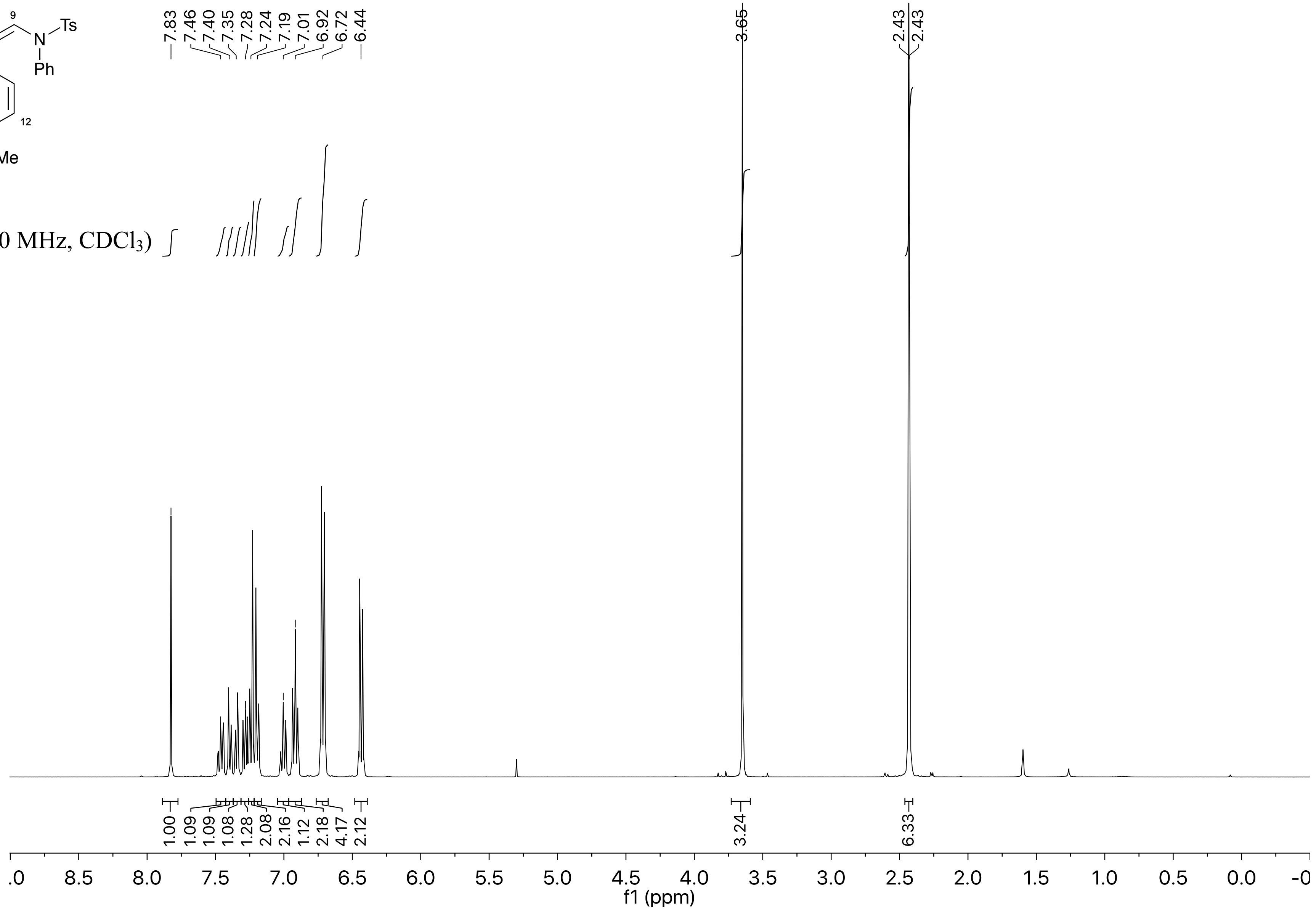

(*E*)-*N*-(2-(4-Methoxyphenyl)-3-(2-(methylthio)phenyl)-3-oxoprop-1-en-1-yl)-4-methyl-*N*-phenylbenzenesulfonamide, 3h

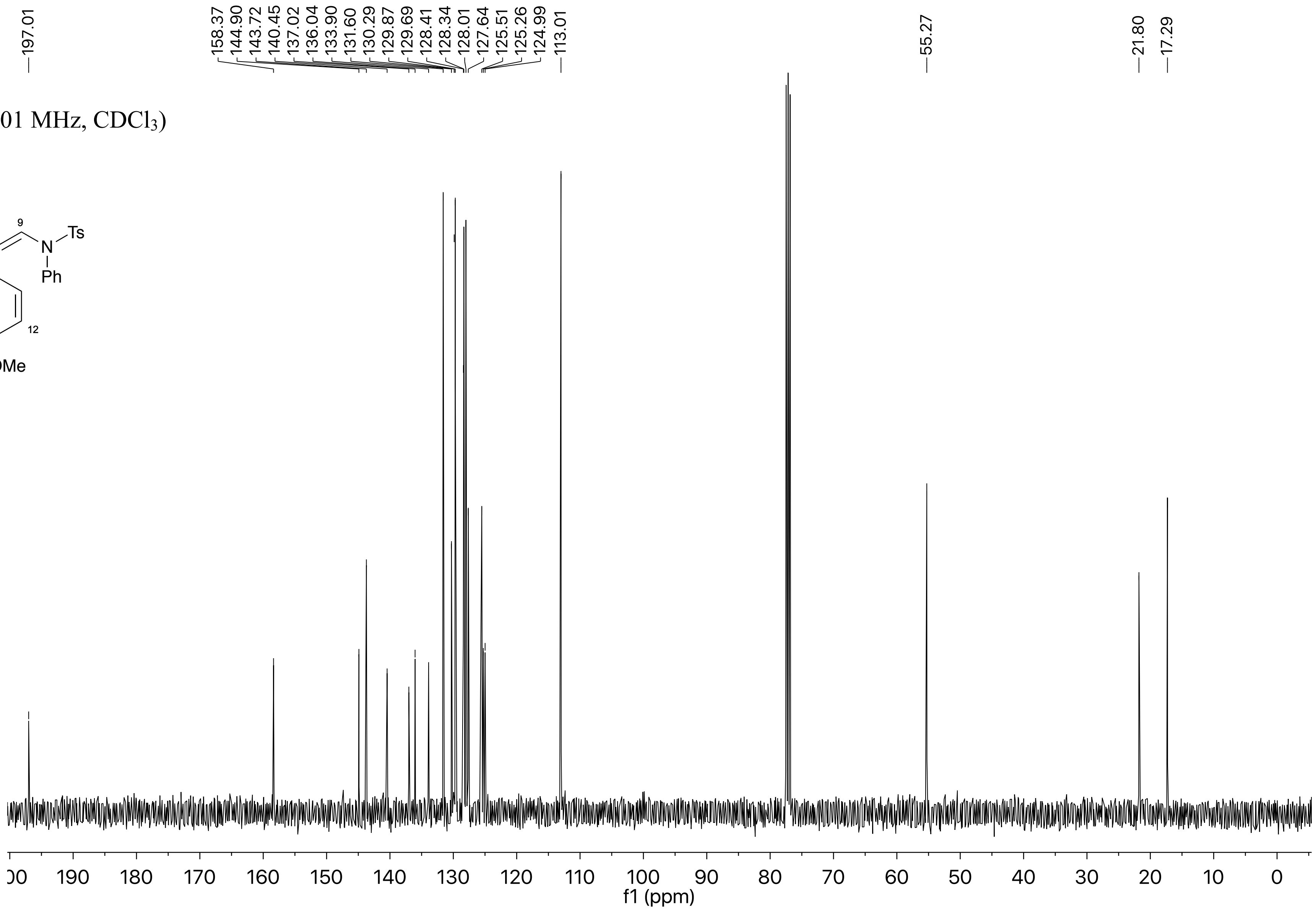

^{13}C NMR (101 MHz, $CDCl_3$)

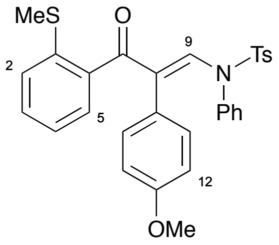

(*E*)-4-Methyl-*N*-(3-(2-(methylthio)phenyl)-3-oxo-2-(4-(trifluoromethyl)phenyl)prop-1-en-1-yl)-*N*-phenylbenzenesulfonamide, 3i

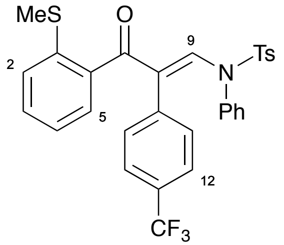

^{1}H NMR (500 MHz, $CDCl_3$)

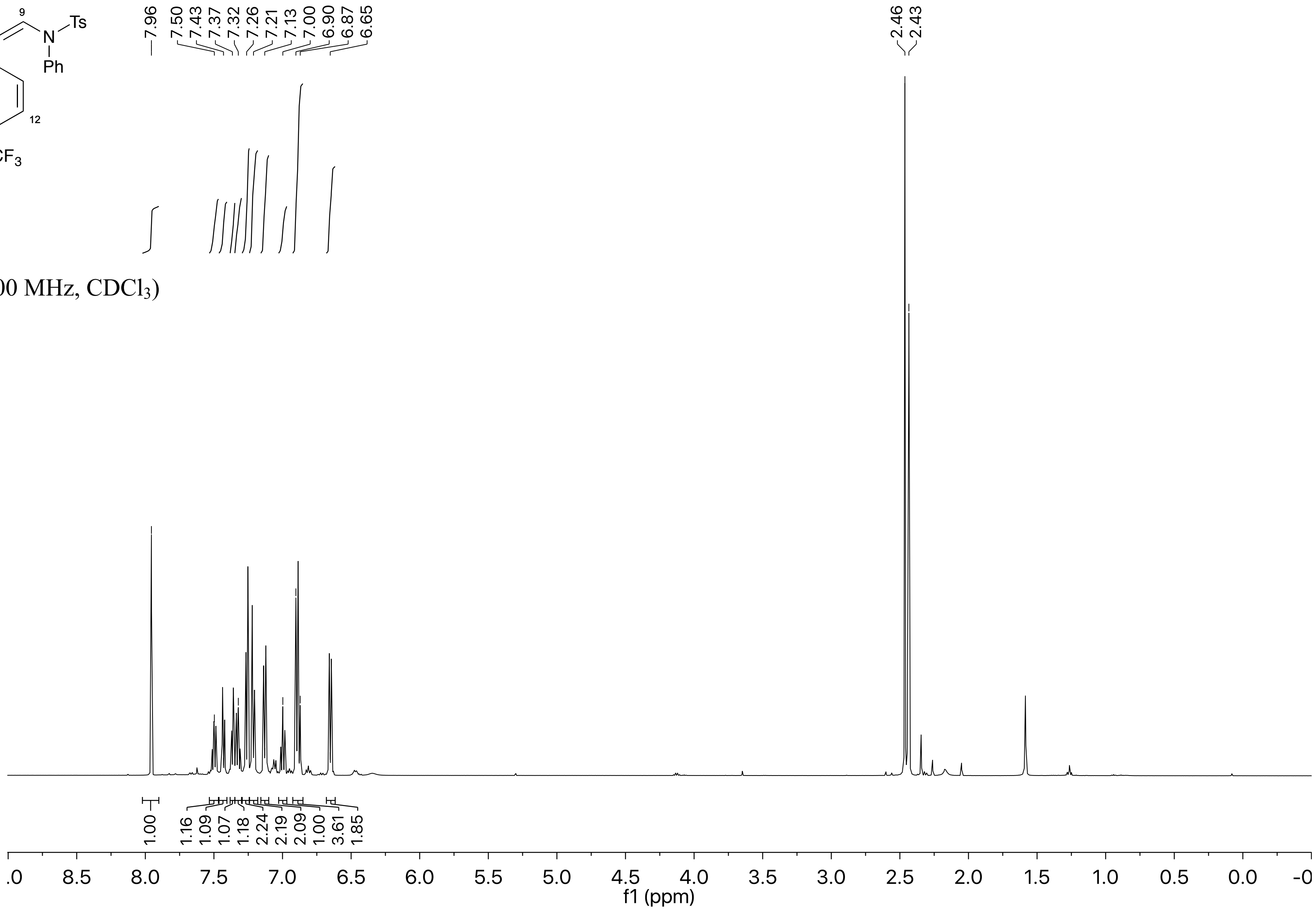

(*E*)-4-Methyl-*N*-(3-(2-(methylthio)phenyl)-3-oxo-2-(4-(trifluoromethyl)phenyl)prop-1-en-1-yl)-*N*-phenylbenzenesulfonamide, 3i

^{13}C NMR (126 MHz, $CDCl_3$)

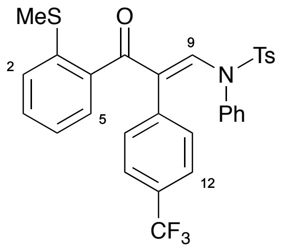

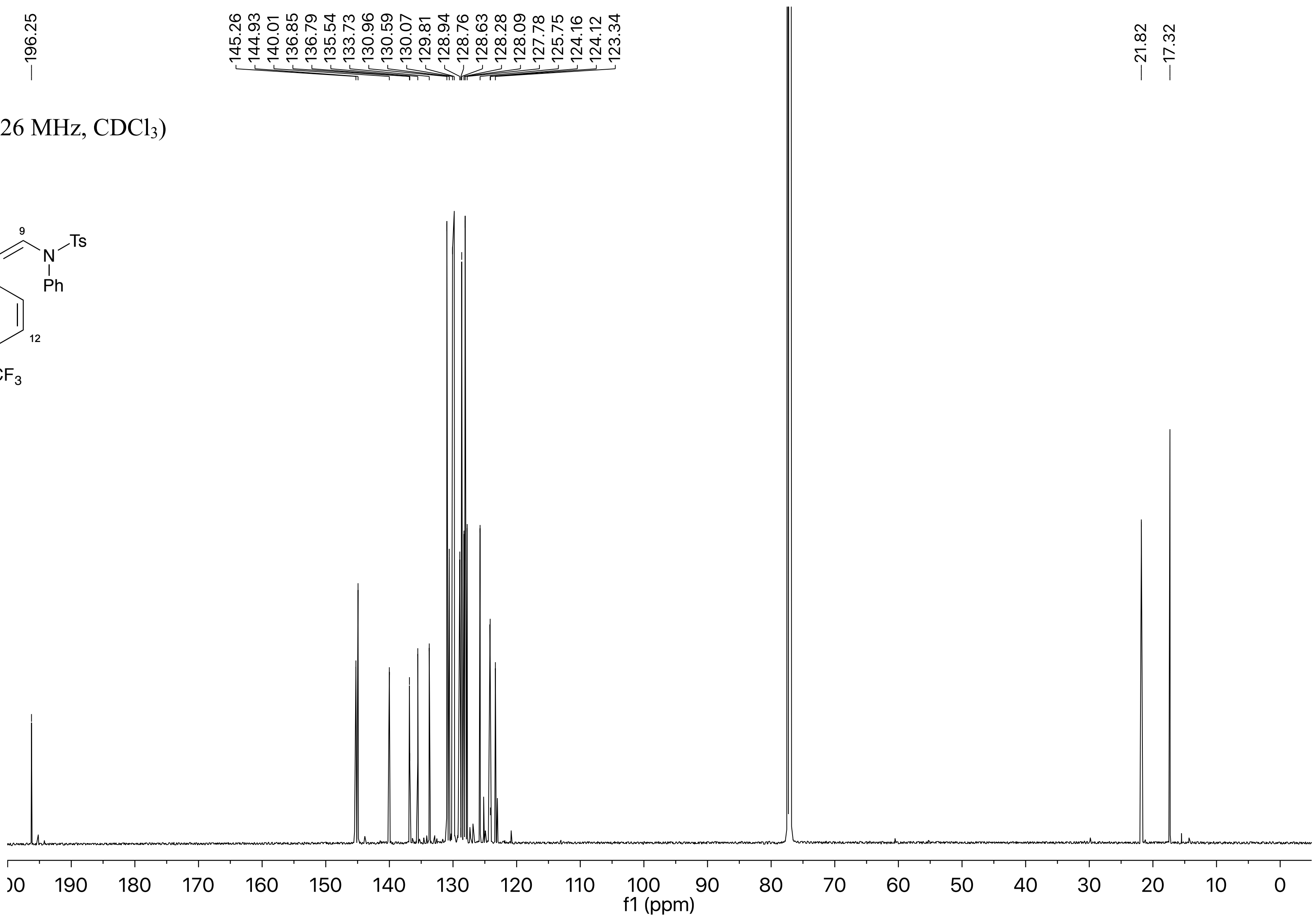

(*E*)-*N*-(2-(3,5-Dimethoxyphenyl)-3-(2-(methylthio)phenyl)-3-oxoprop-1-en-1-yl)-4-methyl-*N*-phenylbenzenesulfonamide, 3j

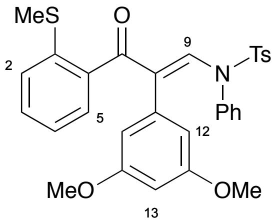

^{1}H NMR (400 MHz, $CDCl_3$)

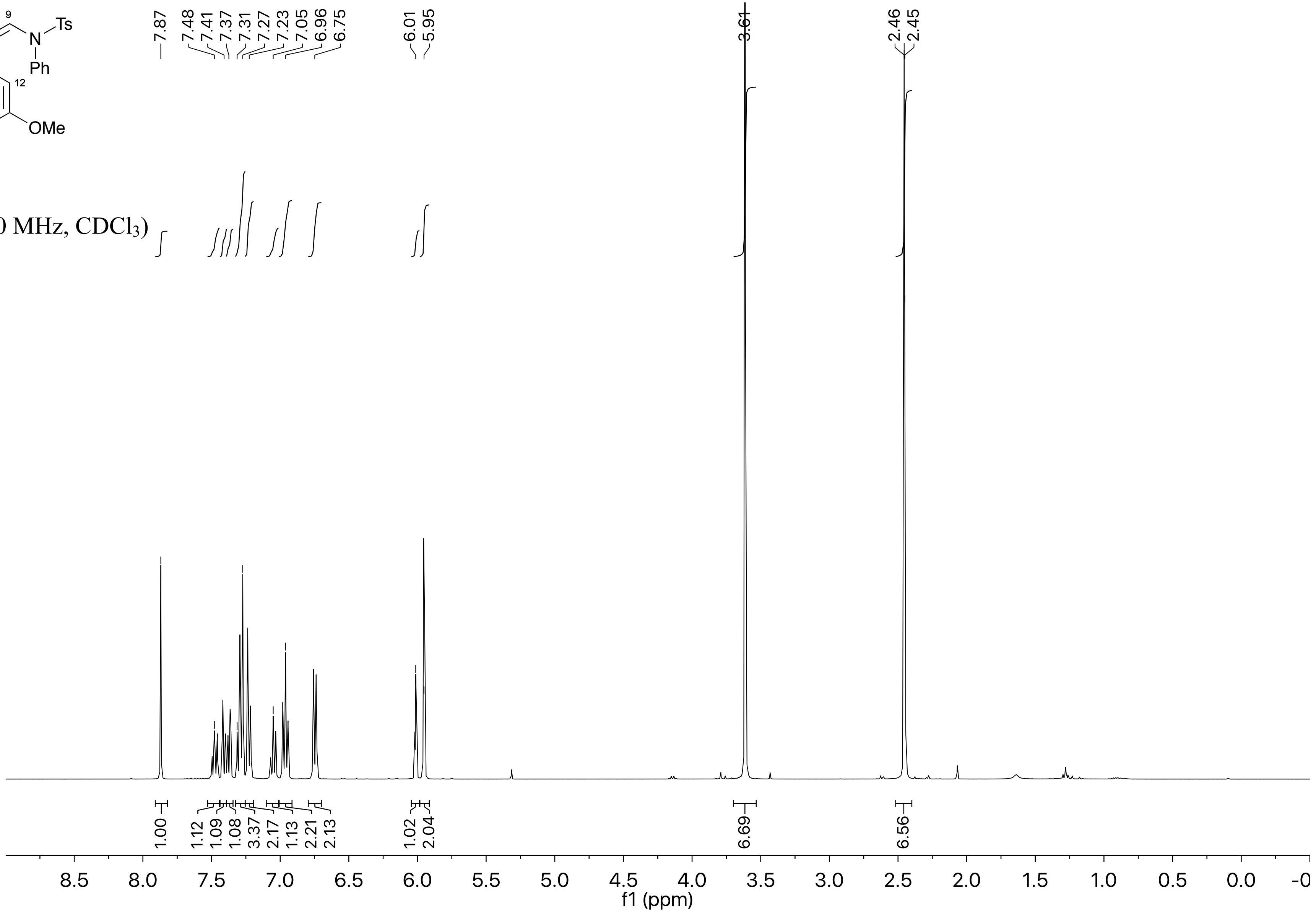

(*E*)-*N*-(2-(3,5-Dimethoxyphenyl)-3-(2-(methylthio)phenyl)-3-oxoprop-1-en-1-yl)-4-methyl-*N*-phenylbenzenesulfonamide, 3j

^{13}C NMR (101 MHz, $CDCl_3$)

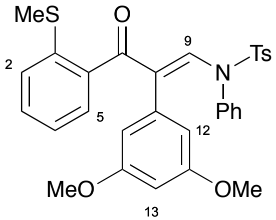

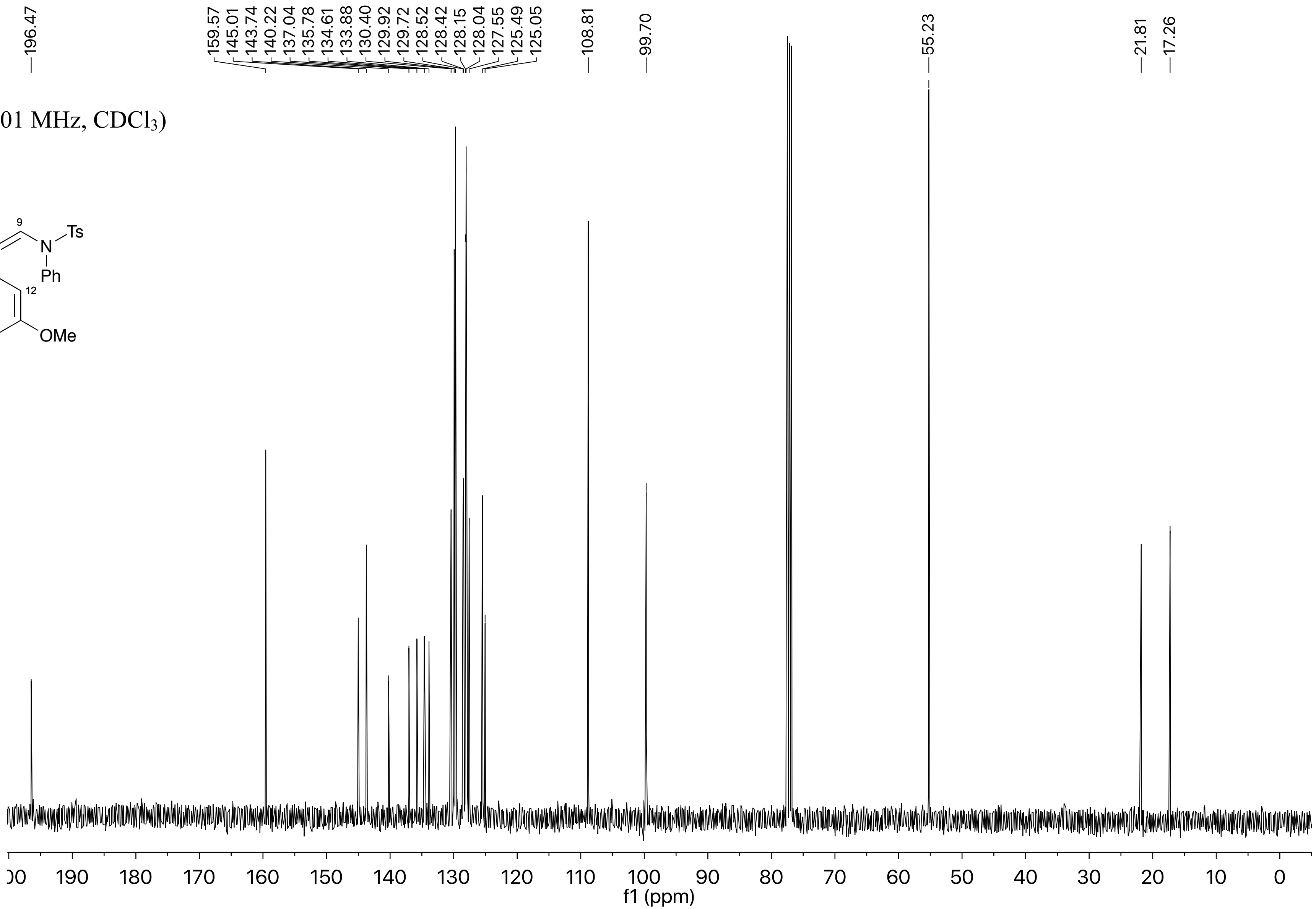

(*E*)-4-Methyl-*N*-(3-(2-(methylthio)phenyl)-3-oxo-2-(thiophen-3-yl)prop-1-en-1-yl)-*N*-phenylbenzenesulfonamide, 3k

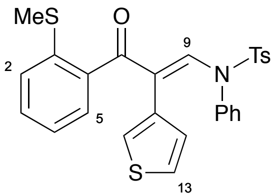

^{1}H NMR (400 MHz, $CDCl_3$)

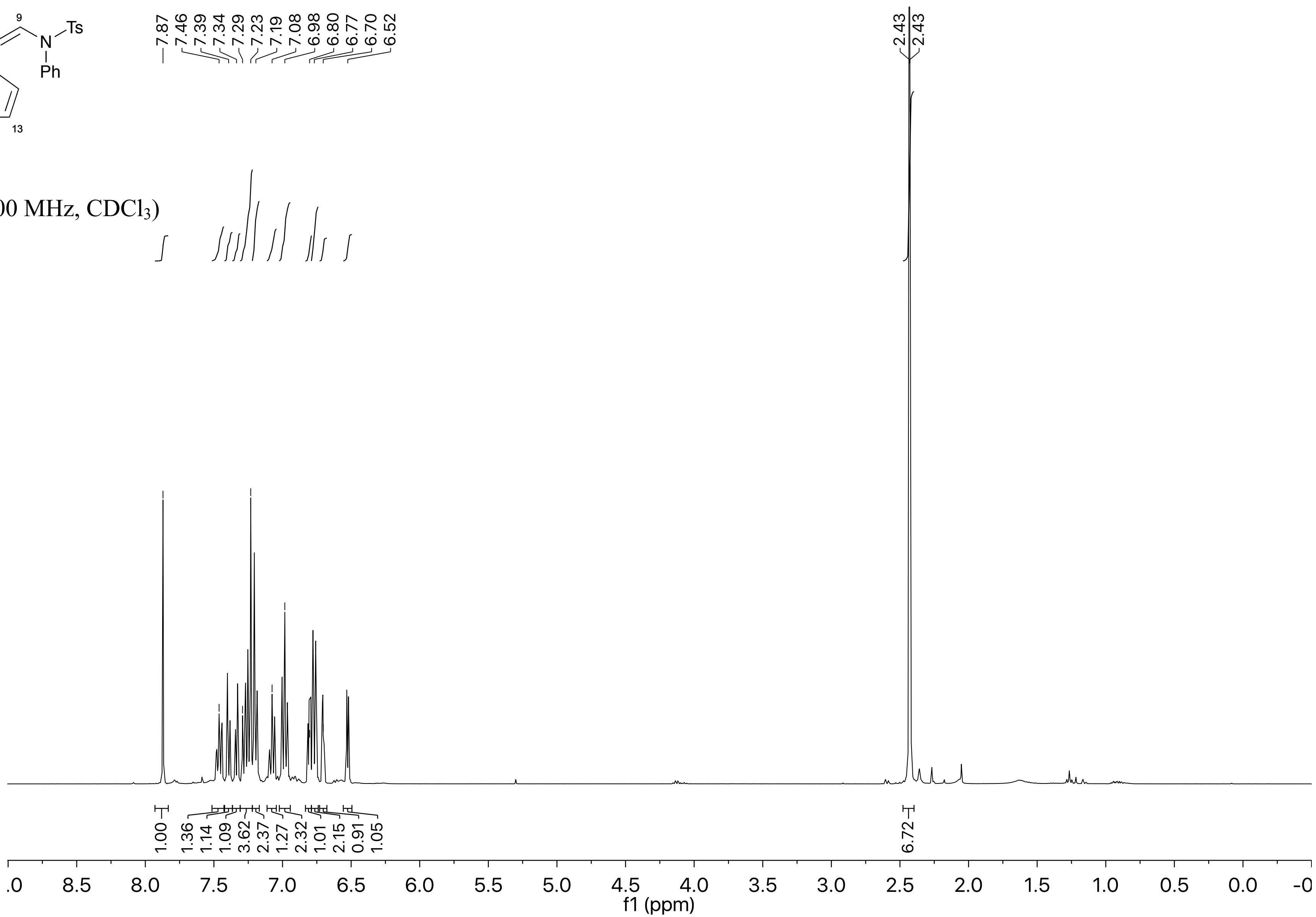

(*E*)-4-Methyl-*N*-(3-(2-(methylthio)phenyl)-3-oxo-2-(thiophen-3-yl)prop-1-en-1-yl)-*N*-phenylbenzenesulfonamide, 3k

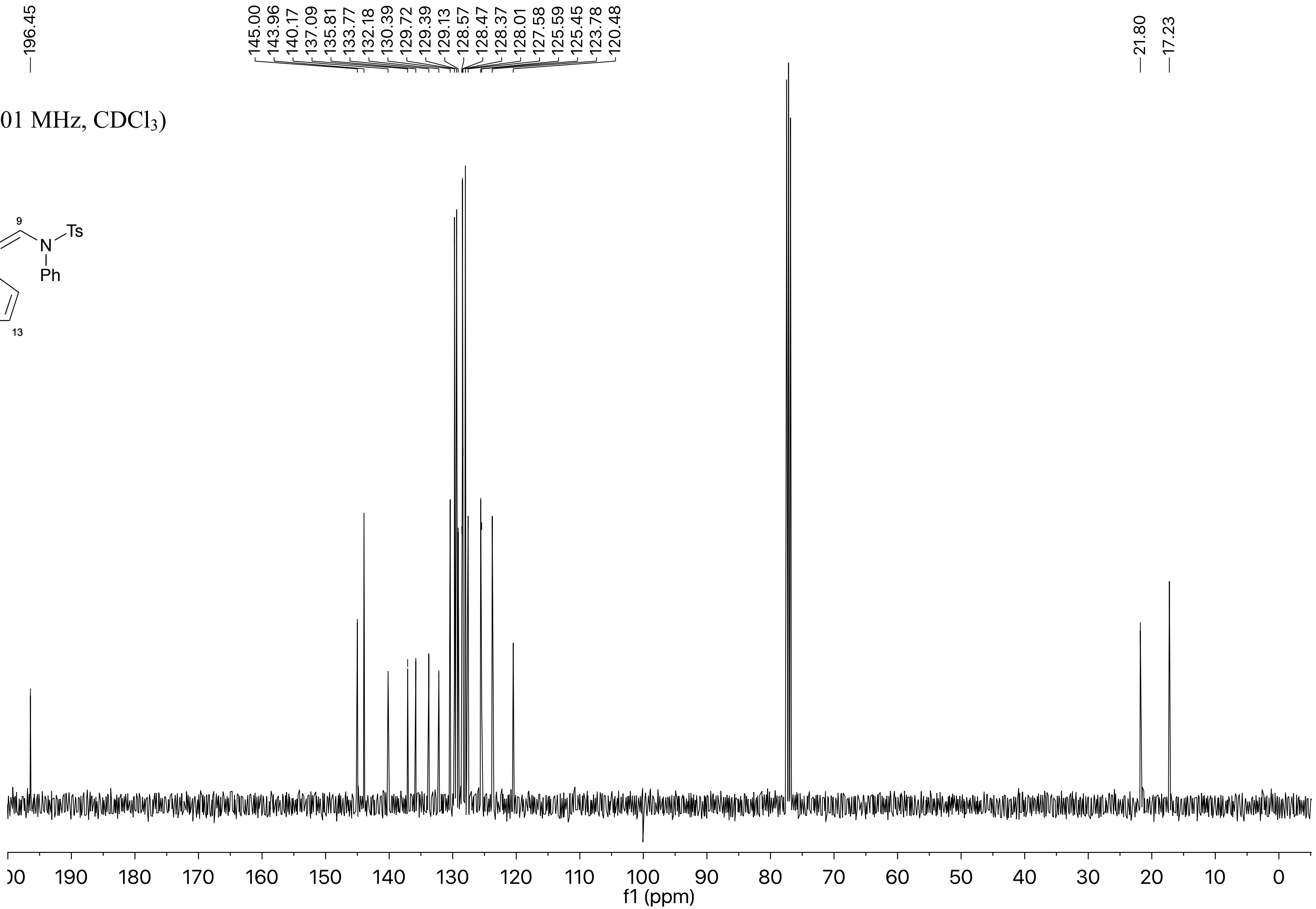

^{13}C NMR (101 MHz, $CDCl_3$)

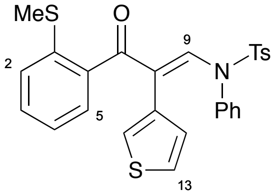

(*E*)-*N*-(2-(Cyclohex-1-en-1-yl)-3-(2-(methylthio)phenyl)-3-oxoprop-1-en-1-yl)-4-methyl-*N*-phenylbenzenesulfonamide, 3l

^{1}H NMR (400 MHz, $CDCl_3$)

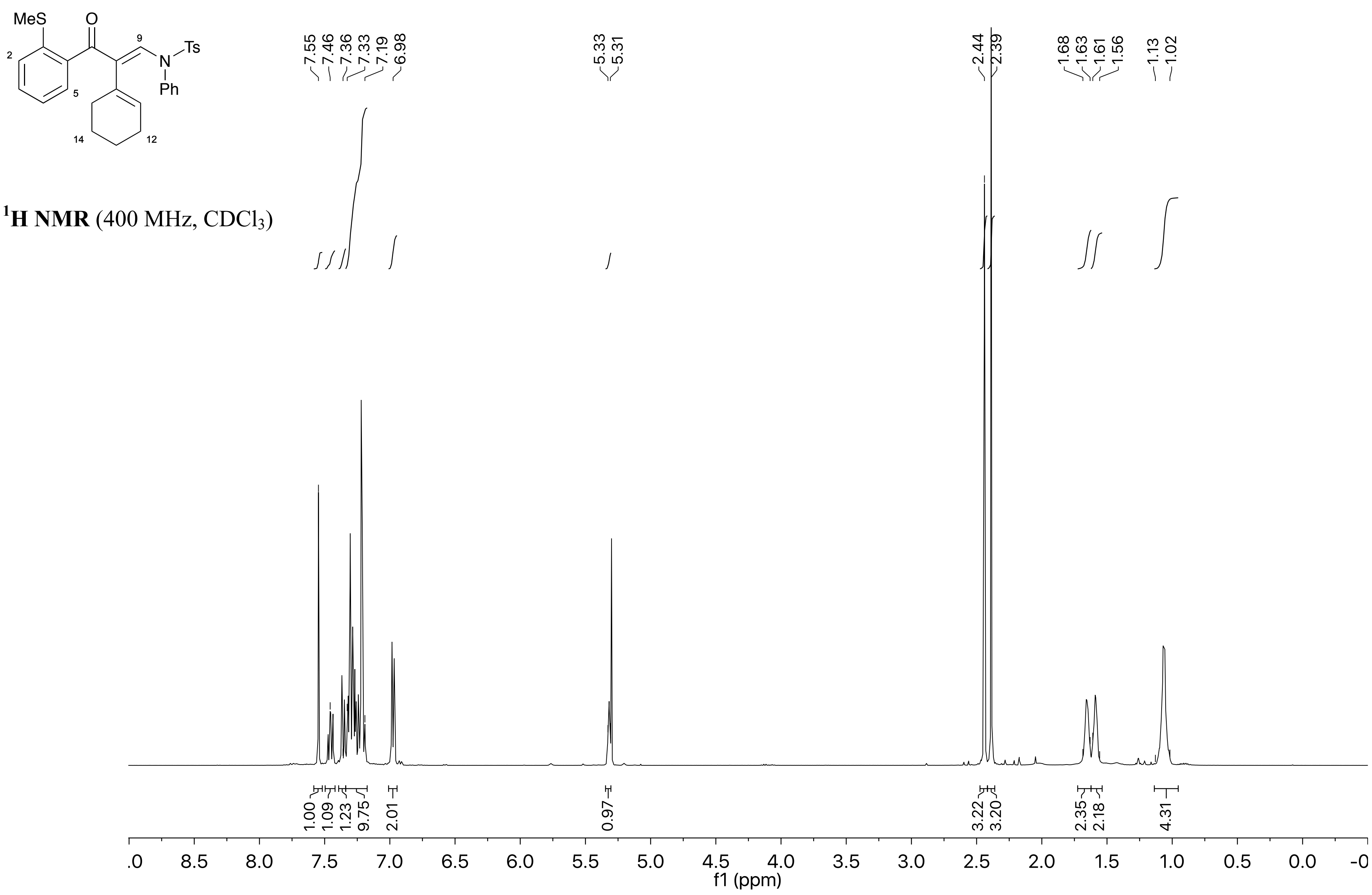

(*E*)-*N*-(2-(Cyclohex-1-en-1-yl)-3-(2-(methylthio)phenyl)-3-oxoprop-1-en-1-yl)-4-methyl-*N*-phenylbenzenesulfonamide, 3l

^{13}C NMR (101 MHz, $CDCl_3$)

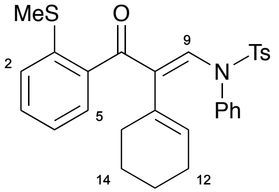

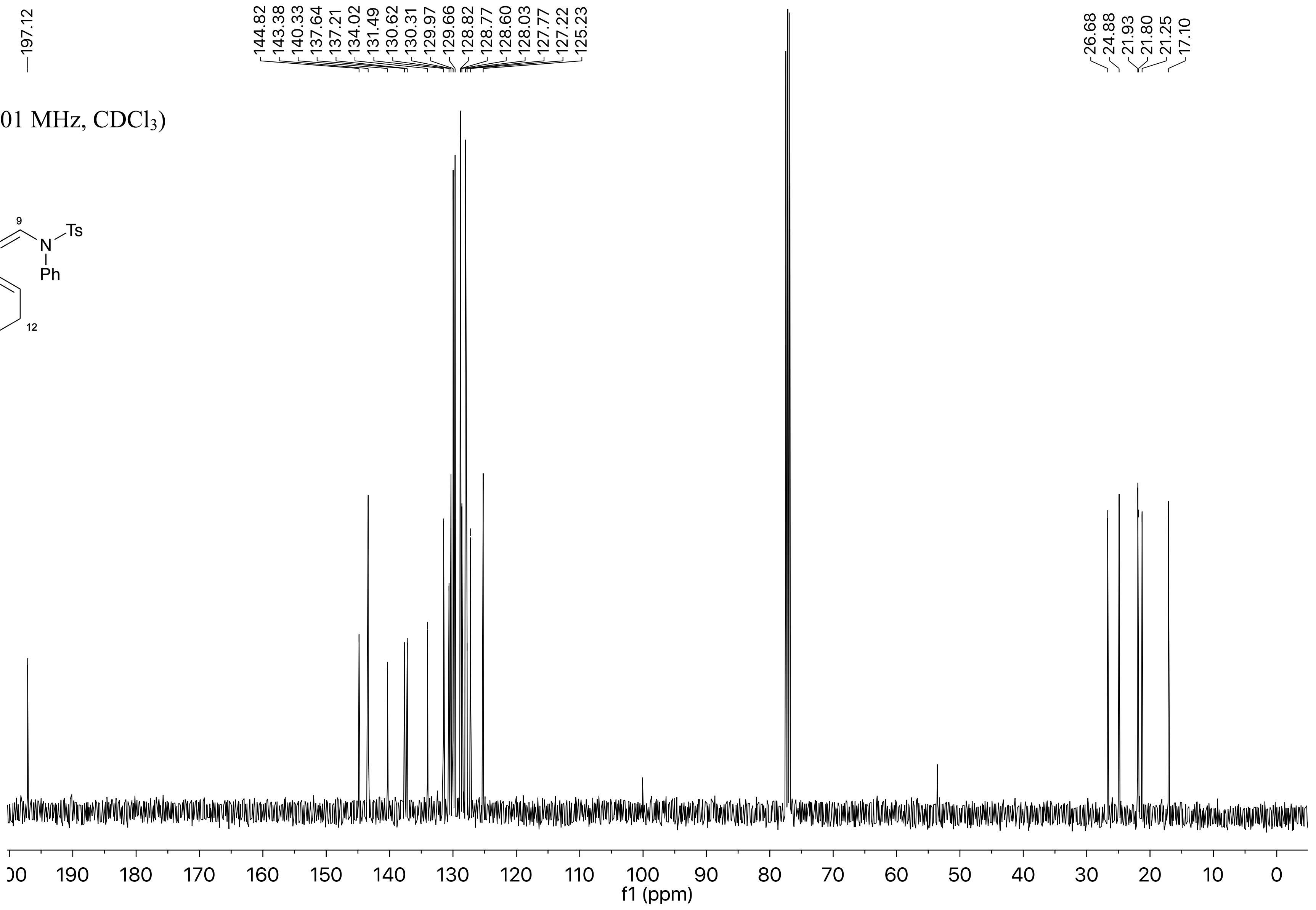

(*E*)-*N*-(2-(2,4-*bis*(Methylthio)benzoyl)oct-1-en-1-yl)-4-methyl-*N*-phenylbenzenesulfonamide, 3m

^{1}H NMR (500 MHz, $CDCl_3$)

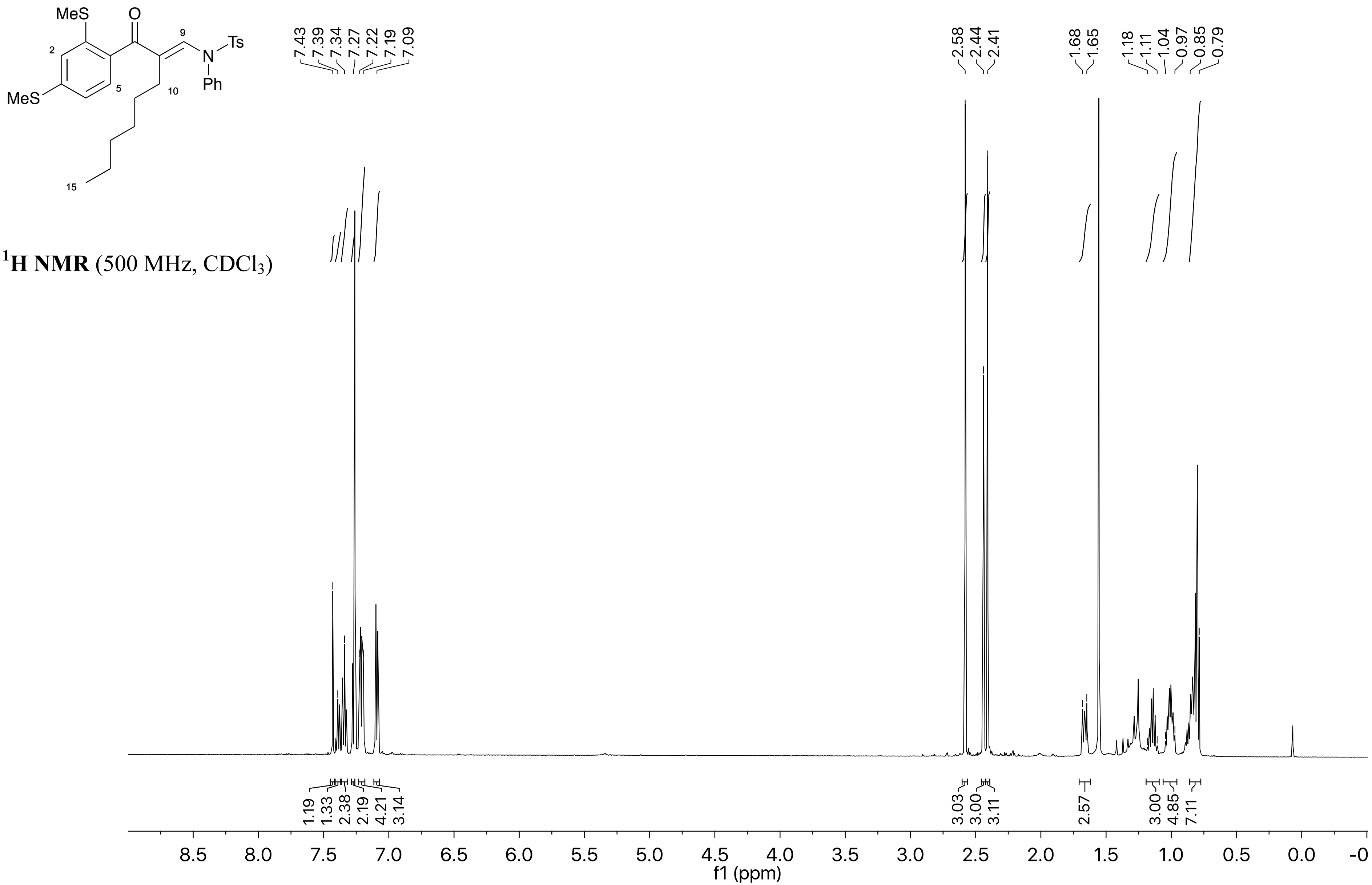

(*E*)-*N*-(2-(2,4-*bis*(Methylthio)benzoyl)oct-1-en-1-yl)-4-methyl-*N*-phenylbenzenesulfonamide, 3m

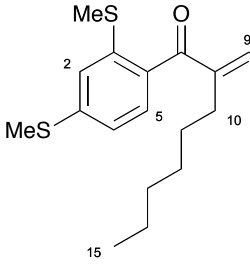

^{13}C NMR (126 MHz, $CDCl_3$)

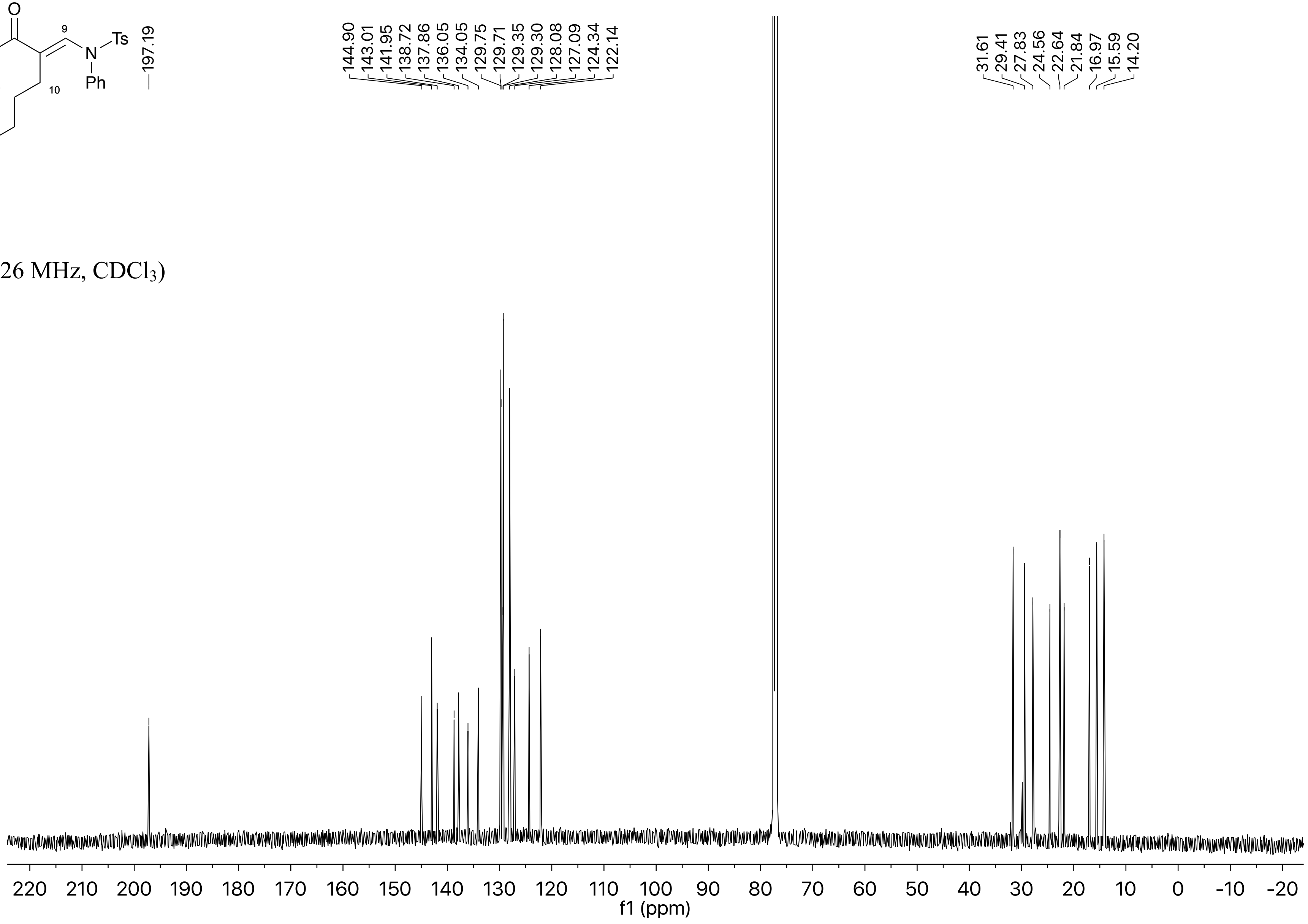

(*Z*)-*N*-(1-(2,4-*bis*(Methylthio)phenyl)-1-oxonon-2-en-2-yl)-4-methyl-*N*-phenylbenzenesulfonamide, 4m

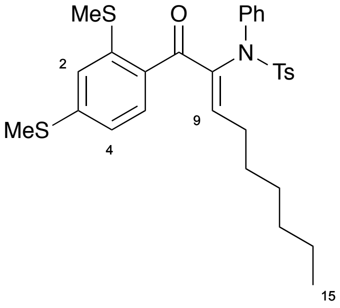

^{1}H NMR (400 MHz, $CDCl_3$)

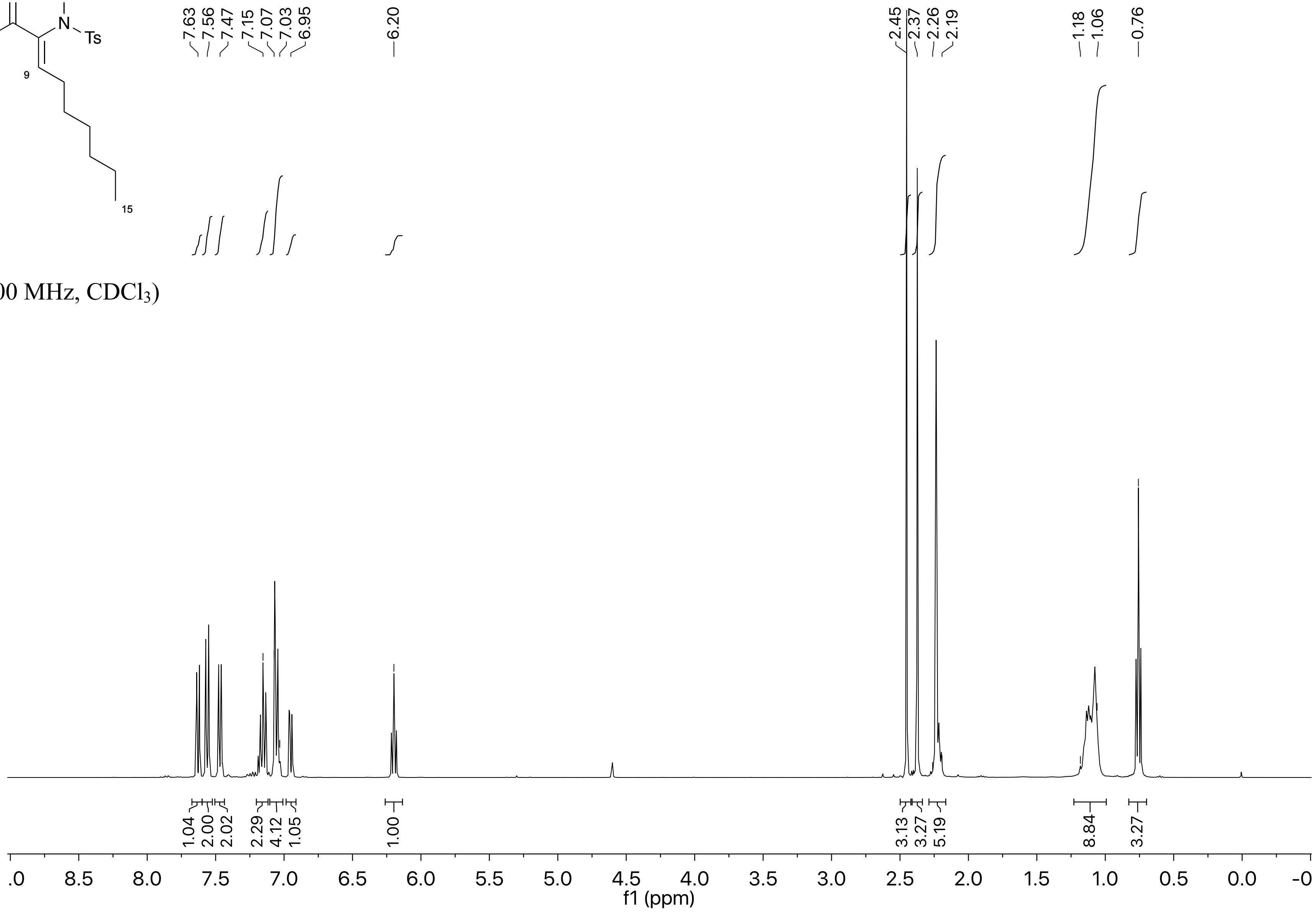

(*Z*)-*N*-(1-(2,4-*bis*(Methylthio)phenyl)-1-oxonon-2-en-2-yl)-4-methyl-*N*-phenylbenzenesulfonamide, 4m

^{13}C NMR (101 MHz, $CDCl_3$)

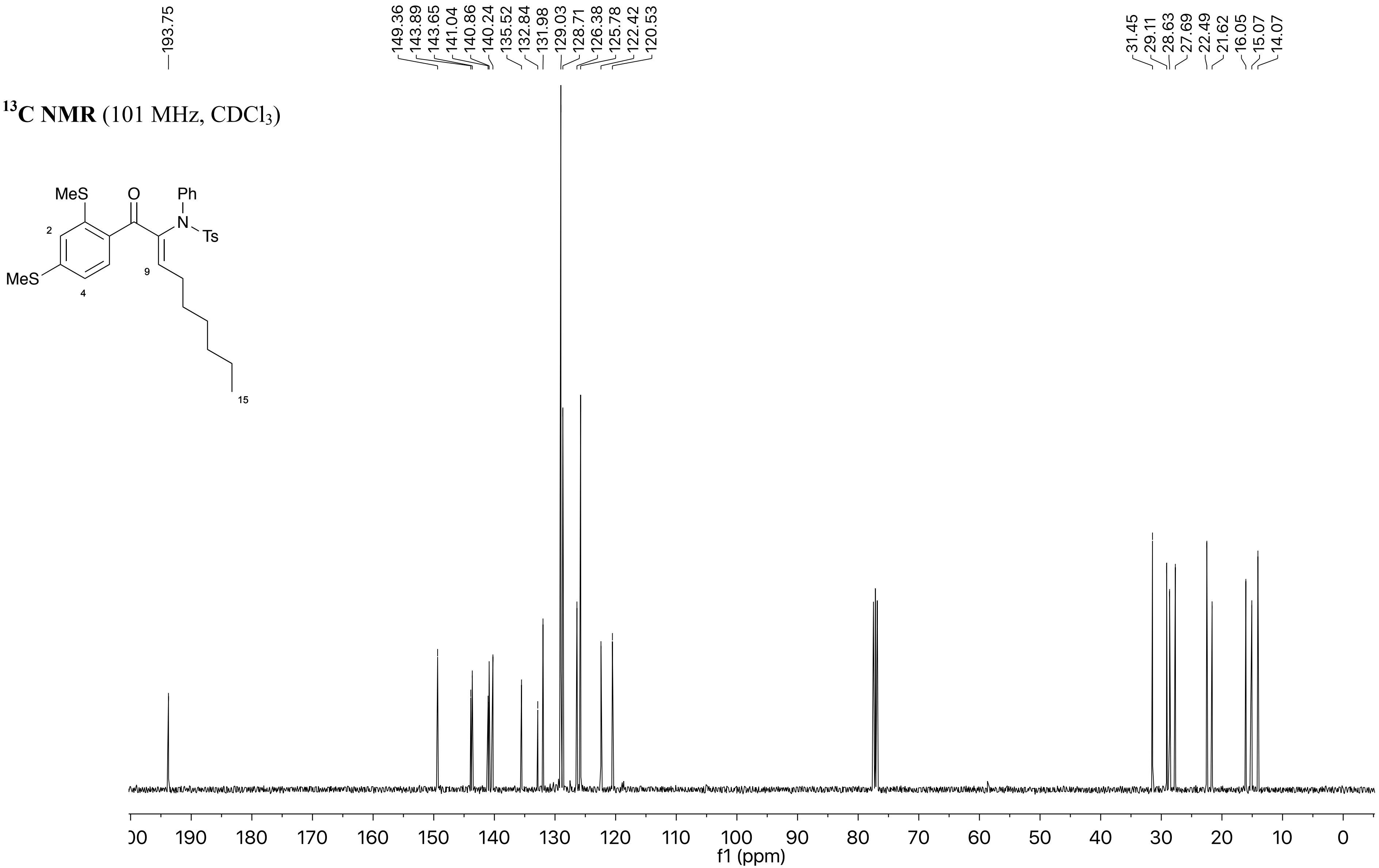

(*E*)-*N*-(2-(4-Methoxy-2-(methylthio)benzoyl)oct-1-en-1-yl)-4-methyl-*N*-phenylbenzenesulfonamide, 3n

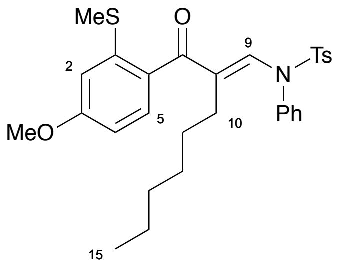

^{1}H NMR (400 MHz, $CDCl_3$)

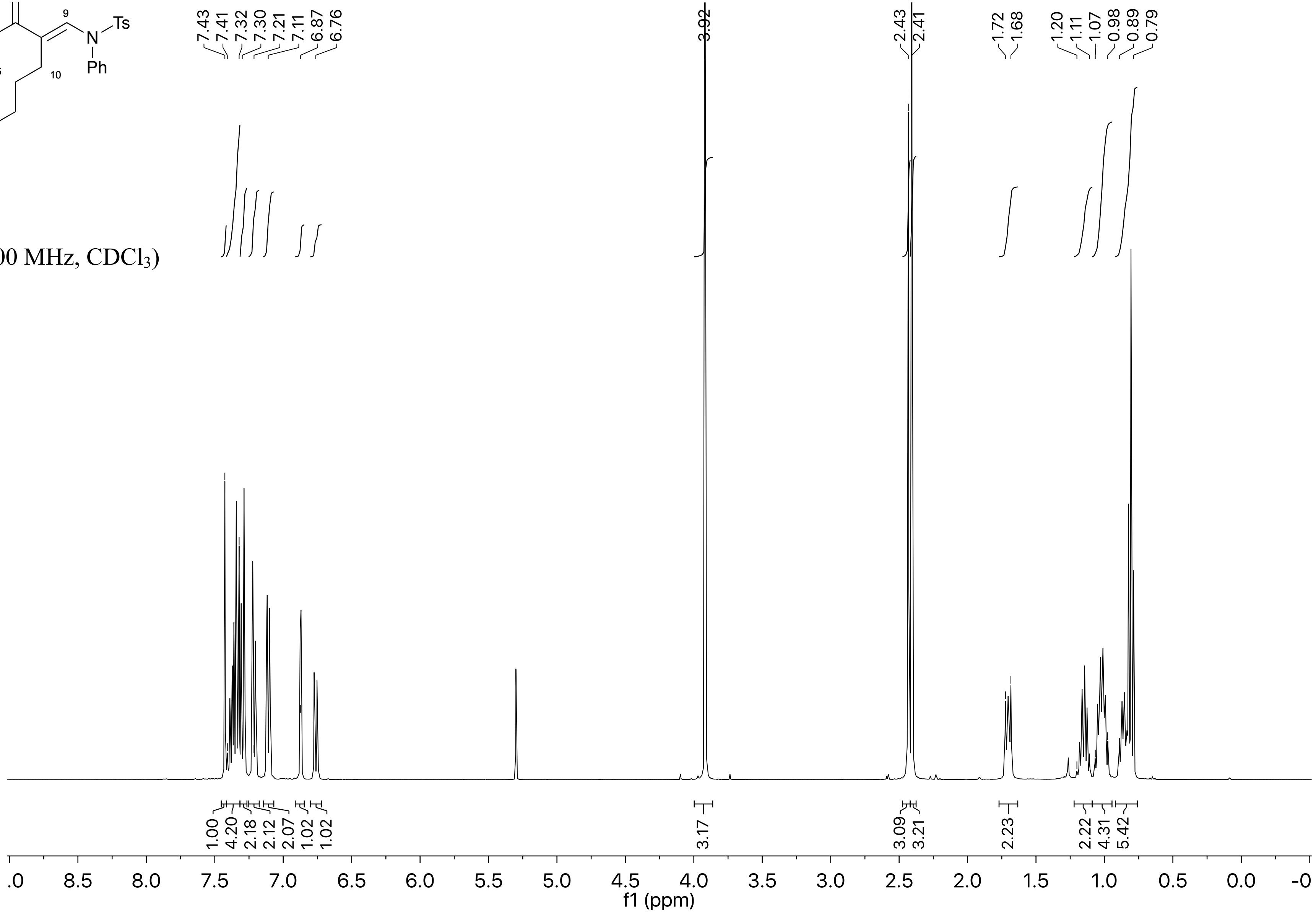

(*E*)-*N*-(2-(4-Methoxy-2-(methylthio)benzoyl)oct-1-en-1-yl)-4-methyl-*N*-phenylbenzenesulfonamide, 3n

^{13}C NMR (101 MHz, $CDCl_3$)

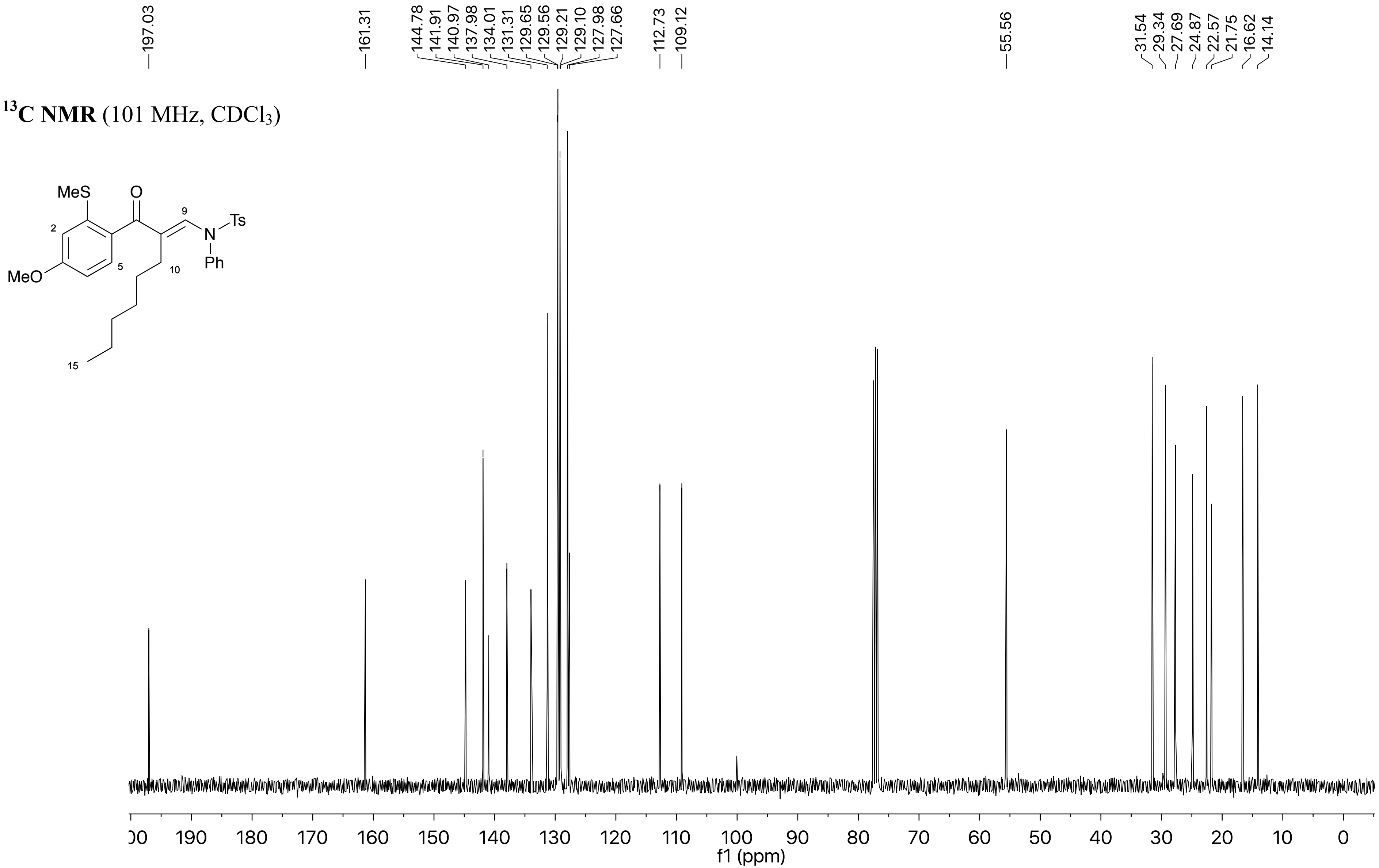

(*E*)-4-Methyl-*N*-(2-(2-(methylthio)-4-(trifluoromethyl)benzoyl)oct-1-en-1-yl)-*N*-phenylbenzenesulfonamide, 3o

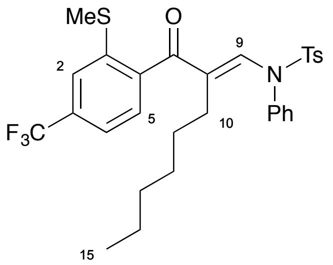

^{1}H NMR (500 MHz, $CDCl_3$)

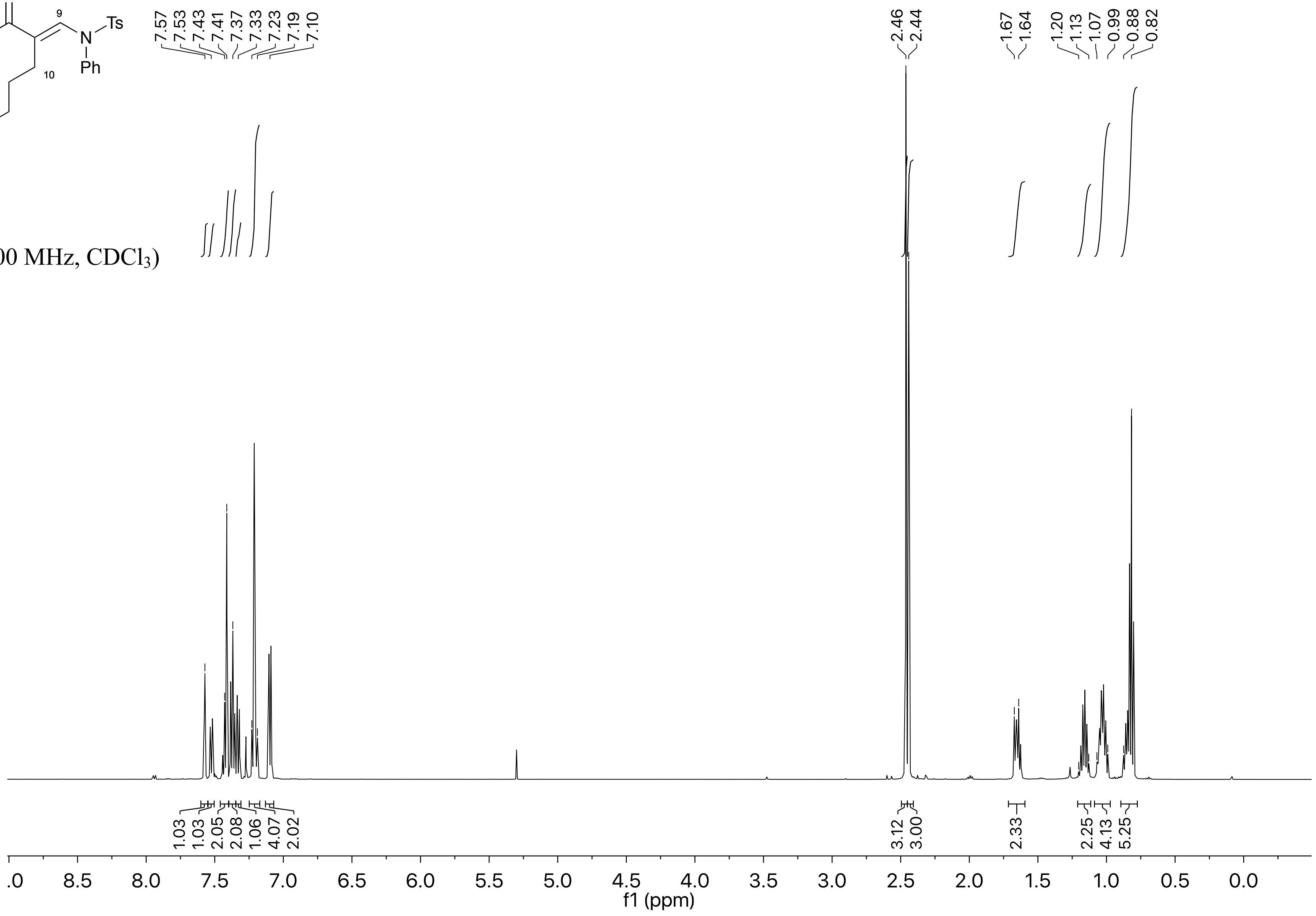

(*E*)-4-Methyl-*N*-(2-(2-(methylthio)-4-(trifluoromethyl)benzoyl)oct-1-en-1-yl)-*N*-phenylbenzenesulfonamide, 3o

^{13}C NMR (126 MHz, $CDCl_3$)

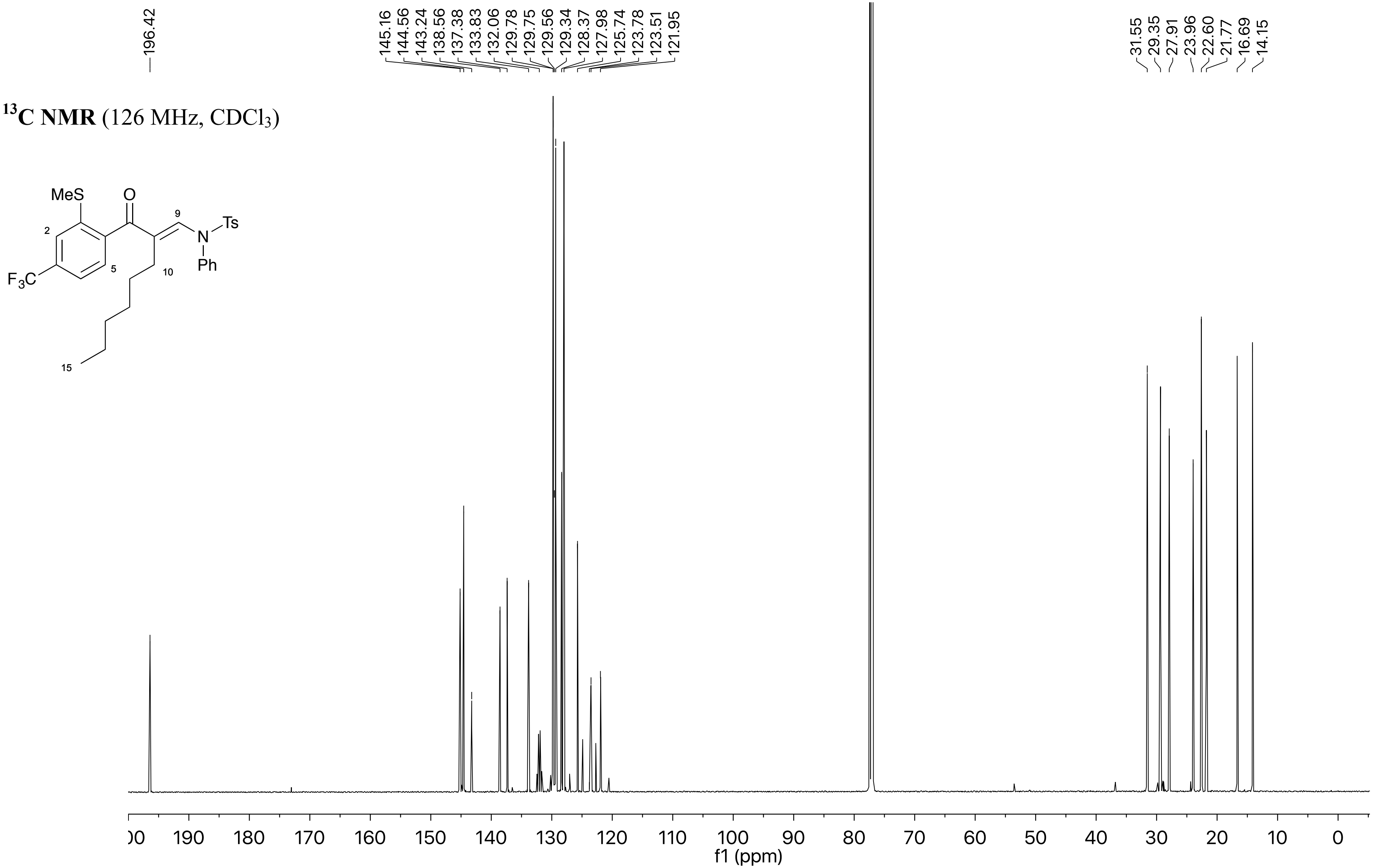

(*E*)-*N*-(2-(4-Bromo-2-(methylthio)benzoyl)oct-1-en-1-yl)-4-methyl-*N*-phenylbenzenesulfonamide, 3p

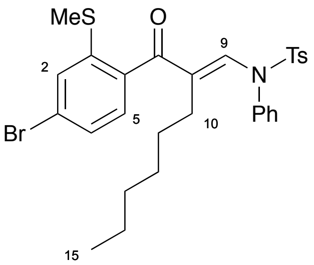

^{1}H NMR (400 MHz, $CDCl_3$)

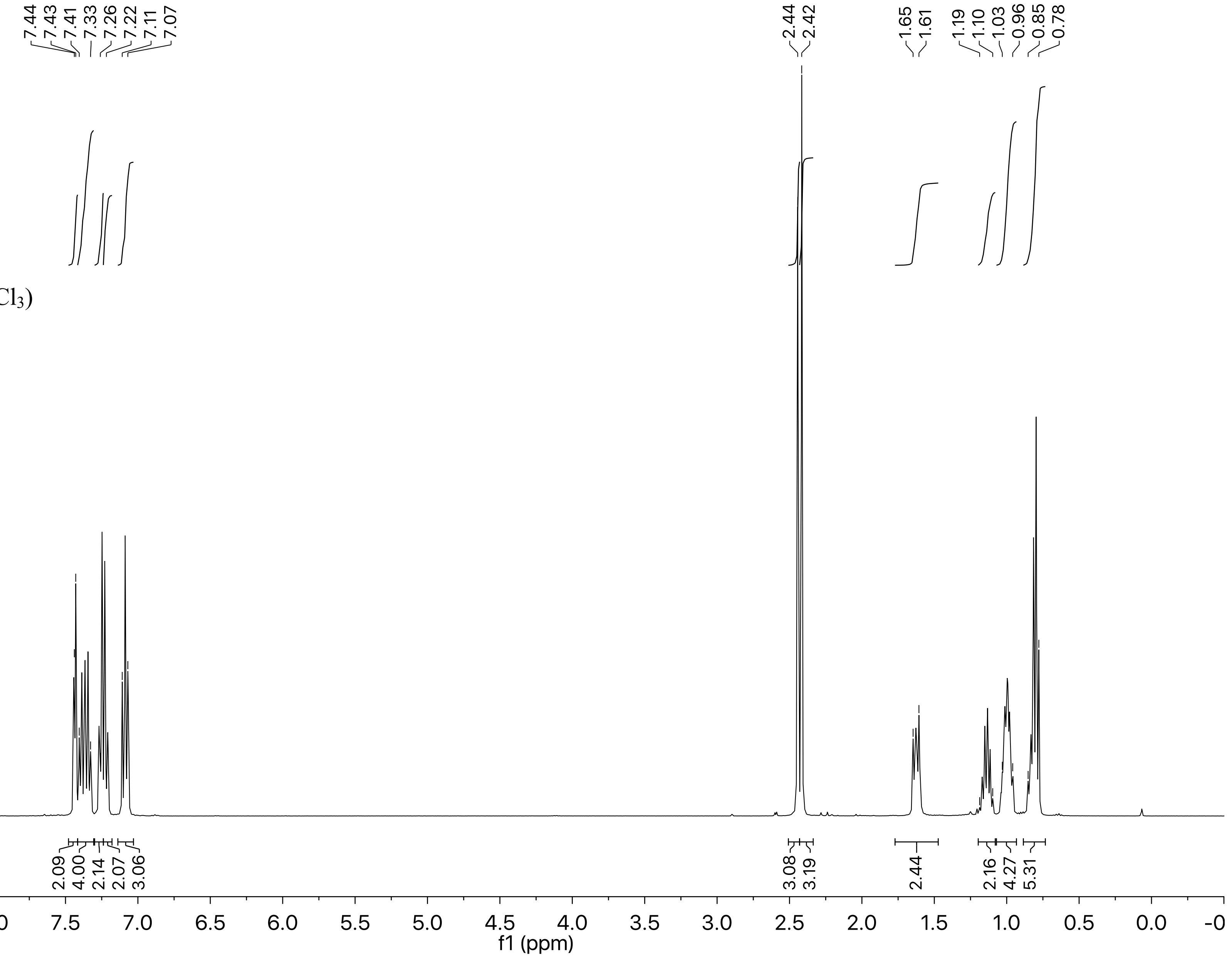

(*E*)-*N*-(2-(4-Bromo-2-(methylthio)benzoyl)oct-1-en-1-yl)-4-methyl-*N*-phenylbenzenesulfonamide, 3p

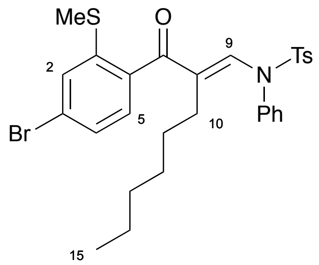

^{13}C NMR (101 MHz, $CDCl_3$)

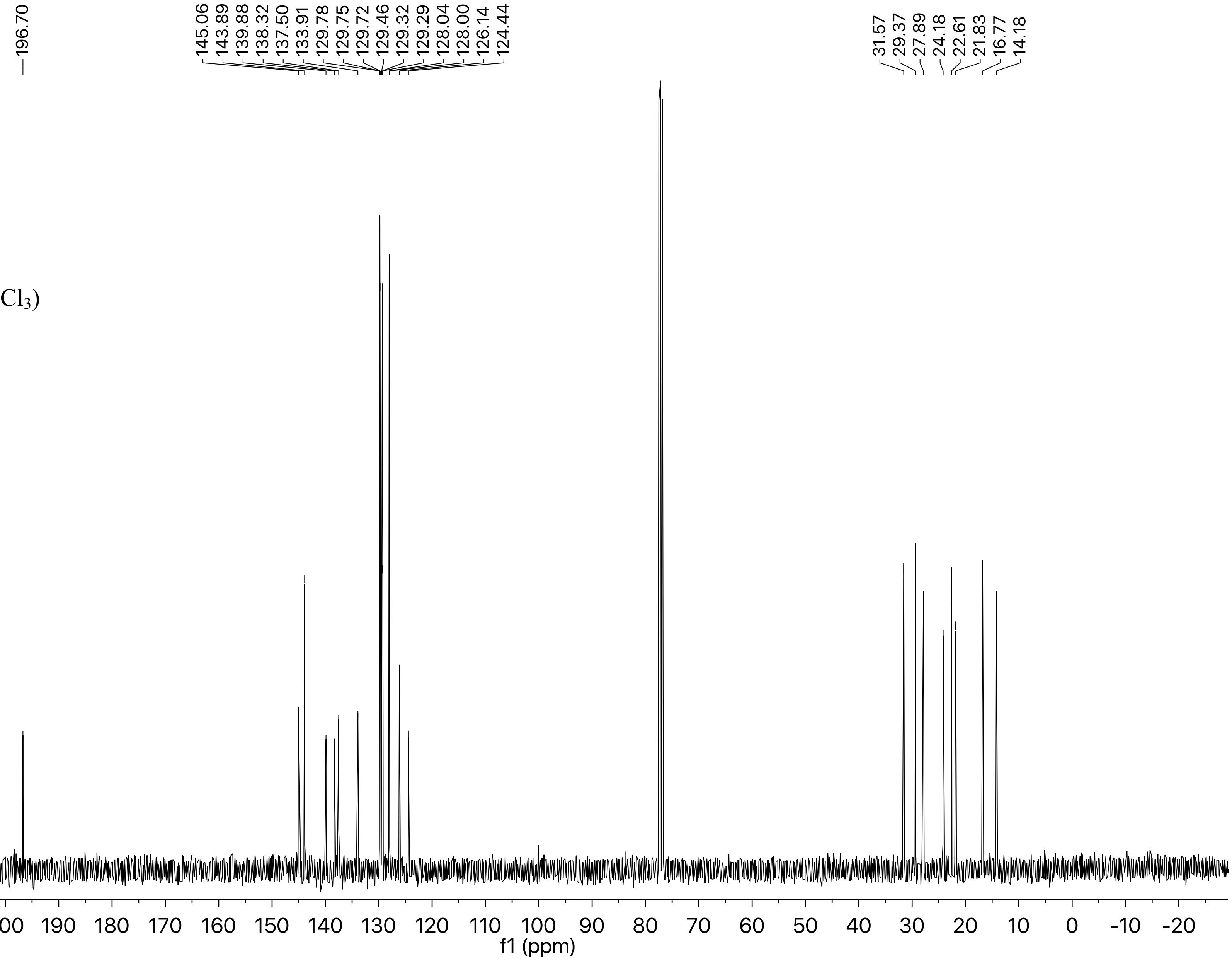

(*E*)-4-Methyl-*N*-(2-(3-(methylthio)thiophene-2-carbonyl)oct-1-en-1-yl)-*N*-phenylbenzenesulfonamide, 3q

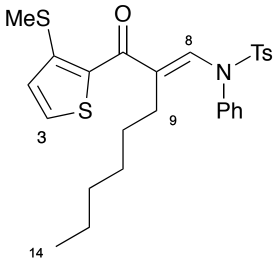

¹H NMR (400 MHz, $CDCl_3$)

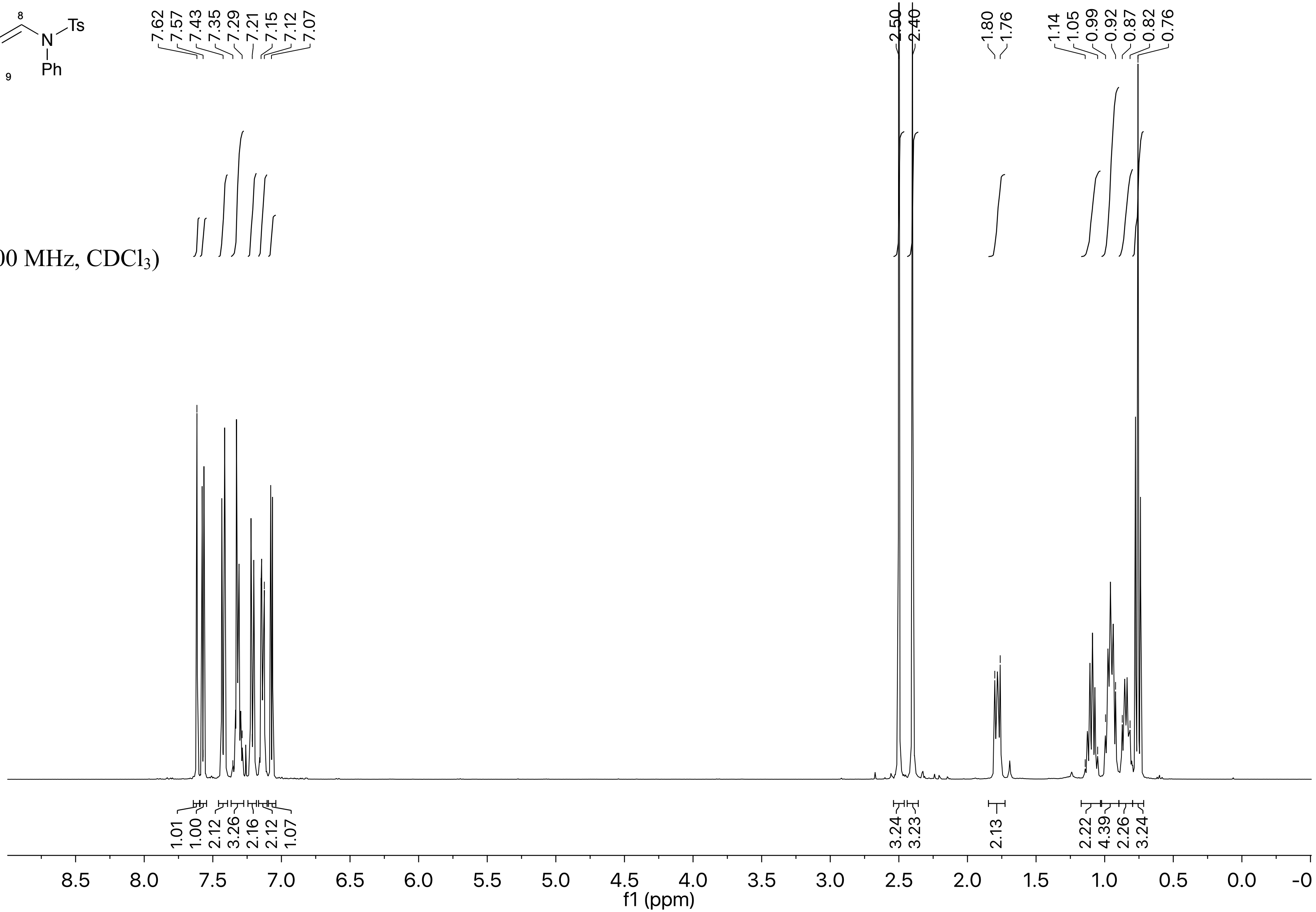

(*E*)-4-Methyl-*N*-(2-(3-(methylthio)thiophene-2-carbonyl)oct-1-en-1-yl)-*N*-phenylbenzenesulfonamide, 3q

^{13}C NMR (101 MHz, $CDCl_3$)

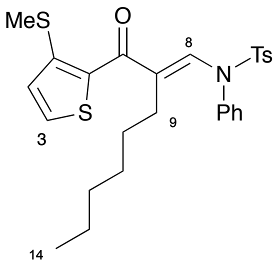

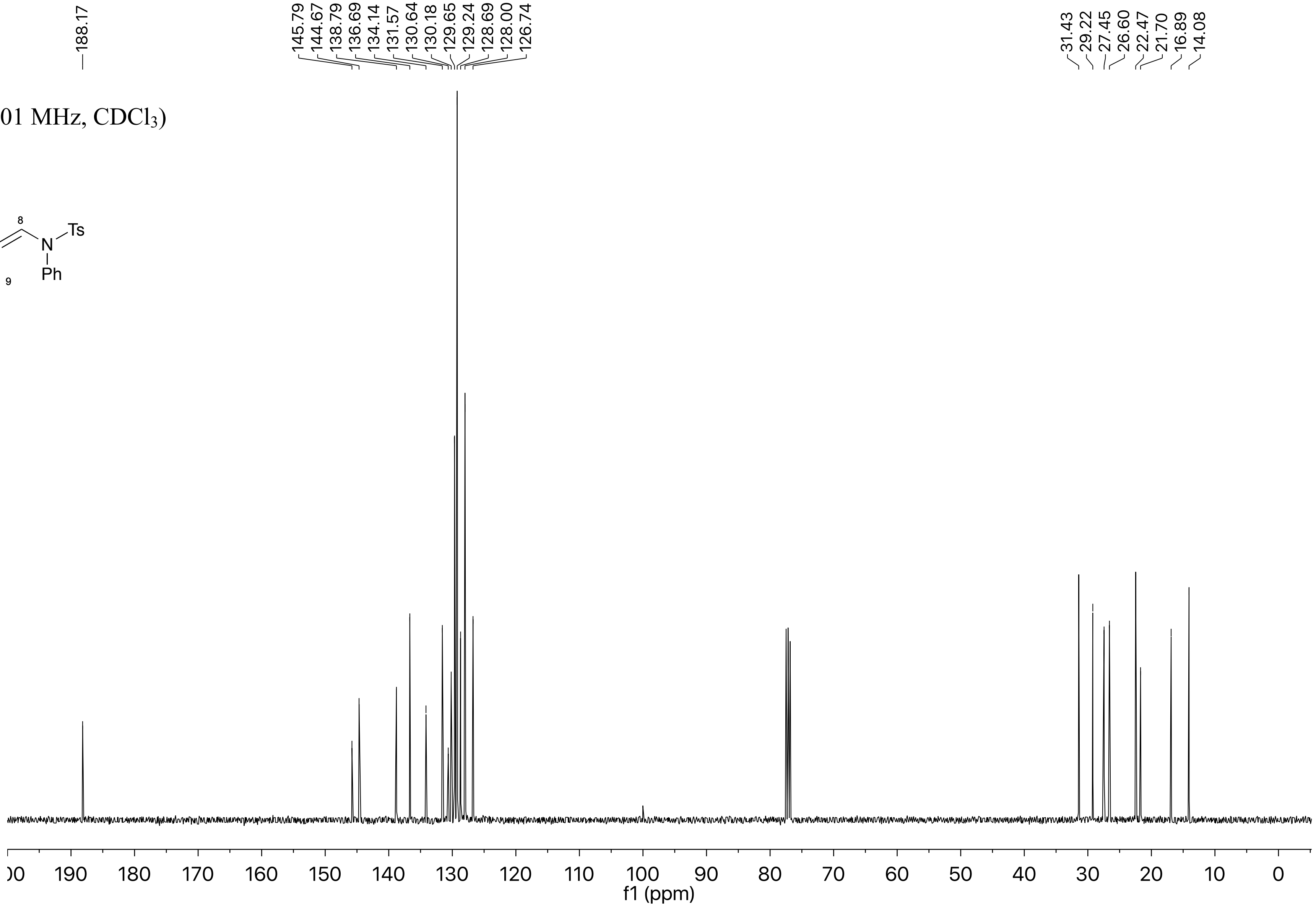

(*E*)-4-Methyl-*N*-(2-(4-(methylthio)-5,6-dihydro-2*H*-pyran-3-carbonyl)oct-1-en-1-yl)-*N*-phenylbenzenesulfonamide, 3r

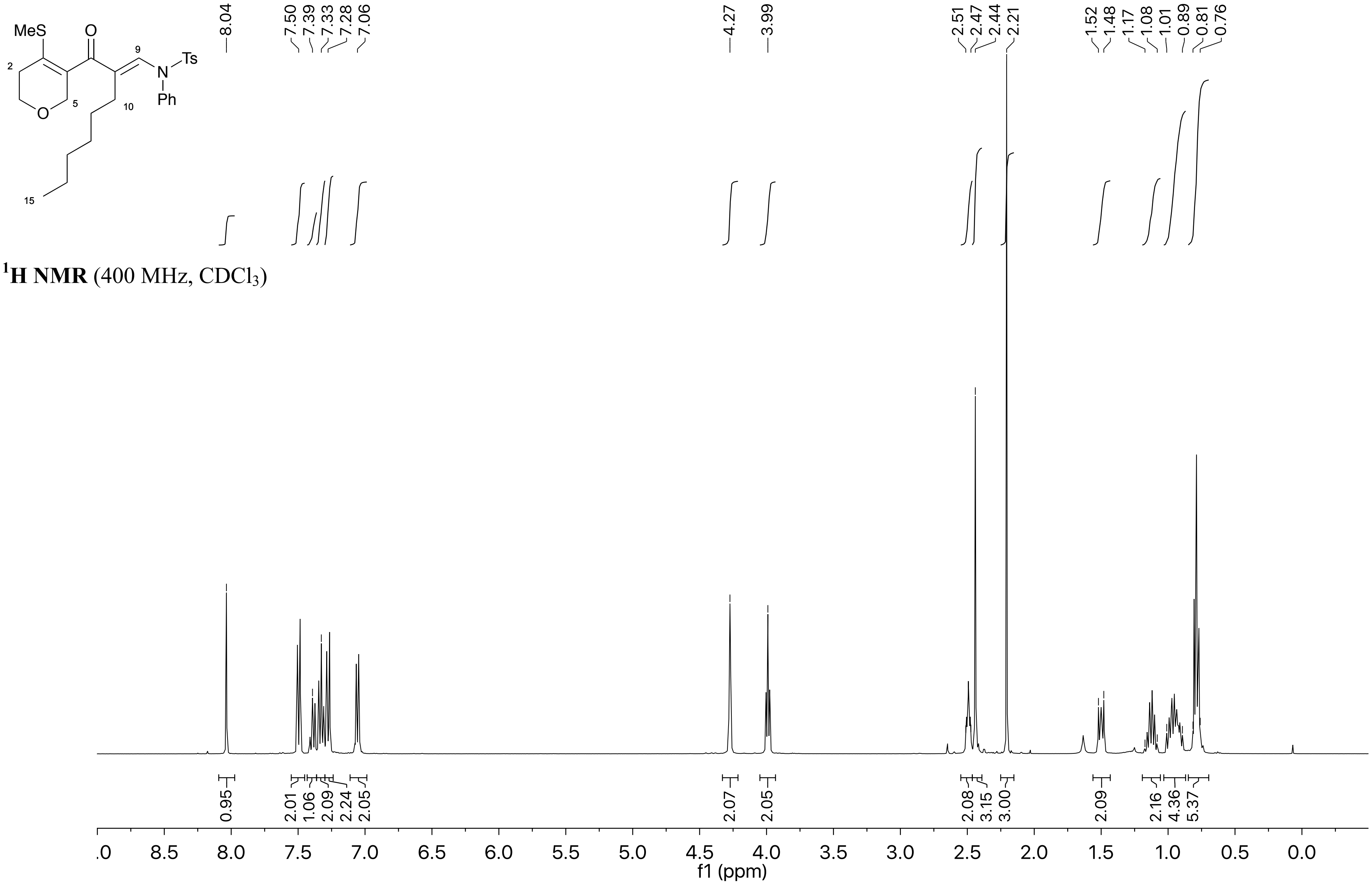

^{1}H NMR (400 MHz, $CDCl_3$)

(*E*)-4-Methyl-*N*-(2-(4-(methylthio)-5,6-dihydro-2*H*-pyran-3-carbonyl)oct-1-en-1-yl)-*N*-phenylbenzenesulfonamide, 3r

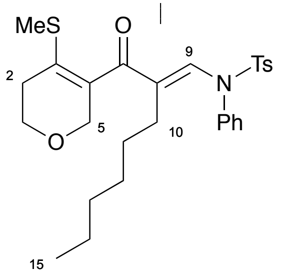

^{13}C NMR (101 MHz, $CDCl_3$)

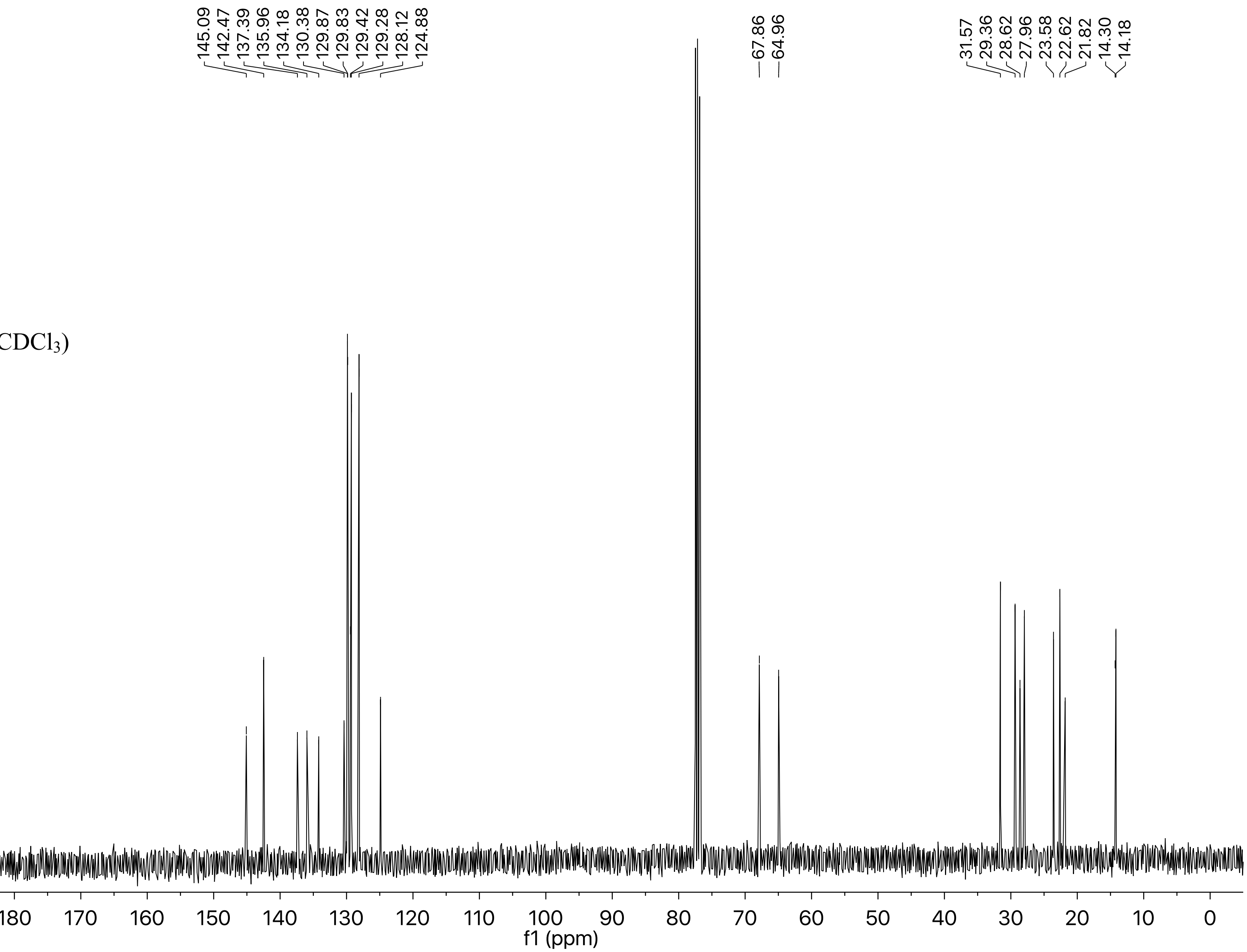

(*E*)-*N*-(2-(2-Benzyl-3-morpholino-3-oxopropanoyl)oct-1-en-1-yl)-4-methyl-*N*-phenylbenzenesulfonamide, 3s

^{1}H NMR (400 MHz, $CDCl_3$)

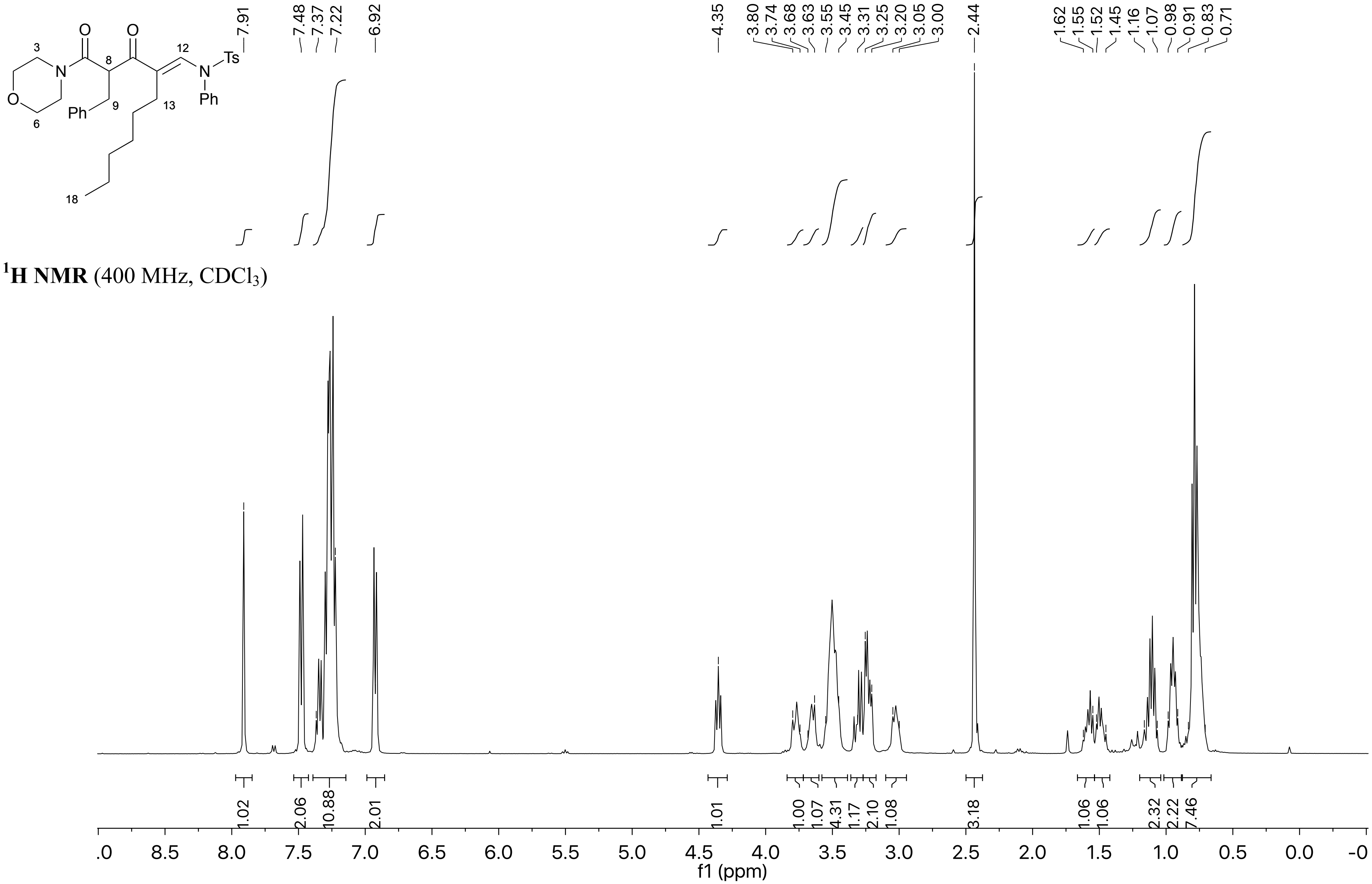

(*E*)-*N*-(2-(2-Benzyl-3-morpholino-3-oxopropanoyl)oct-1-en-1-yl)-4-methyl-*N*-phenylbenzenesulfonamide, 3s

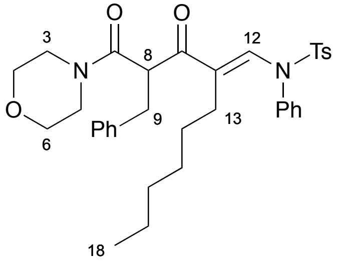

^{13}C NMR (101 MHz, $CDCl_3$)

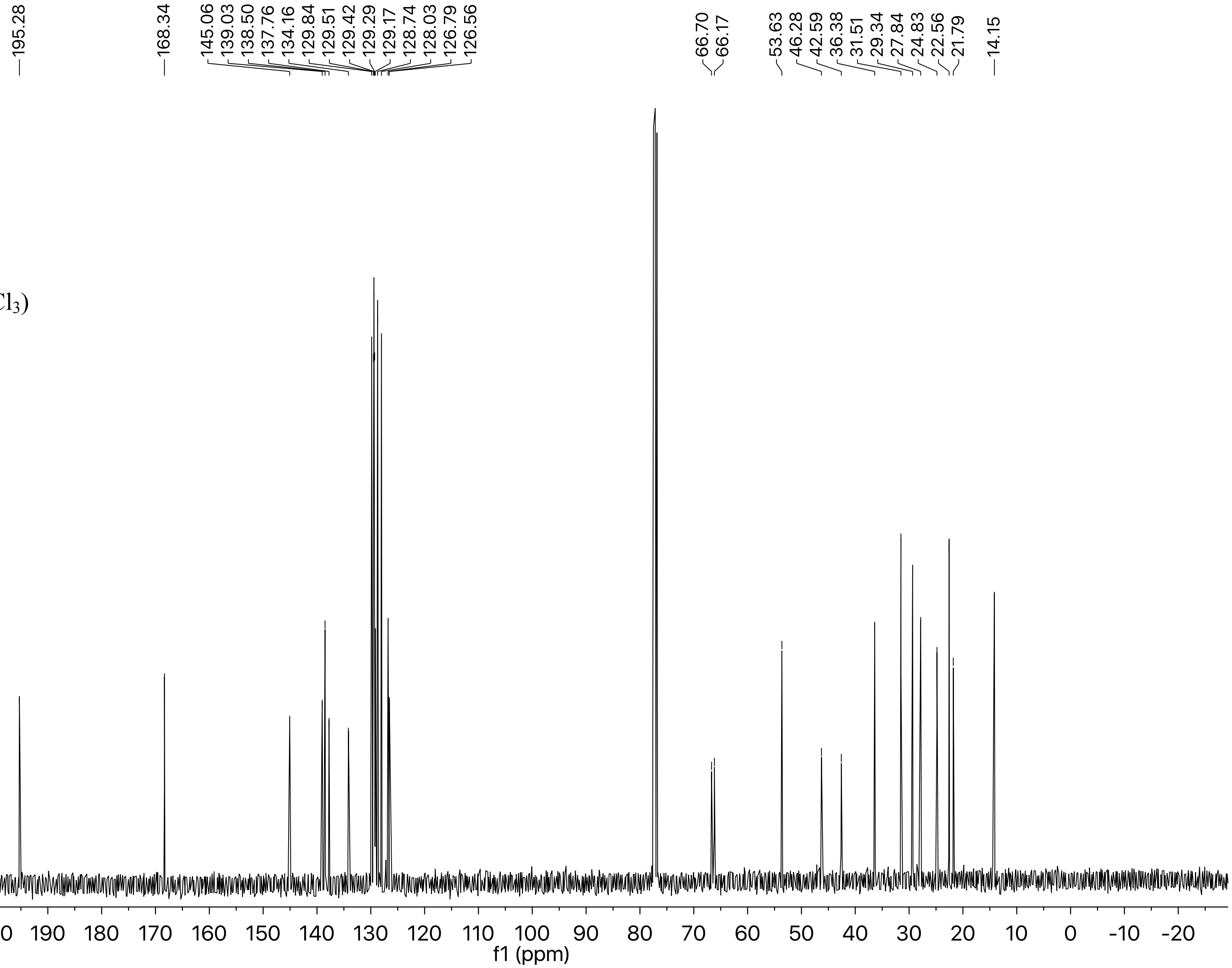

(*E*)-*N*-(4-((Ethylthio)methyl)-2-hexyl-3-oxodec-1-en-1-yl)-4-methyl-*N*-phenylbenzenesulfonamide, 3t

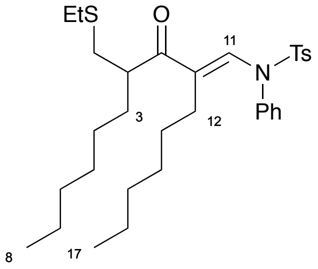

^{1}H NMR (400 MHz, $CDCl_3$)

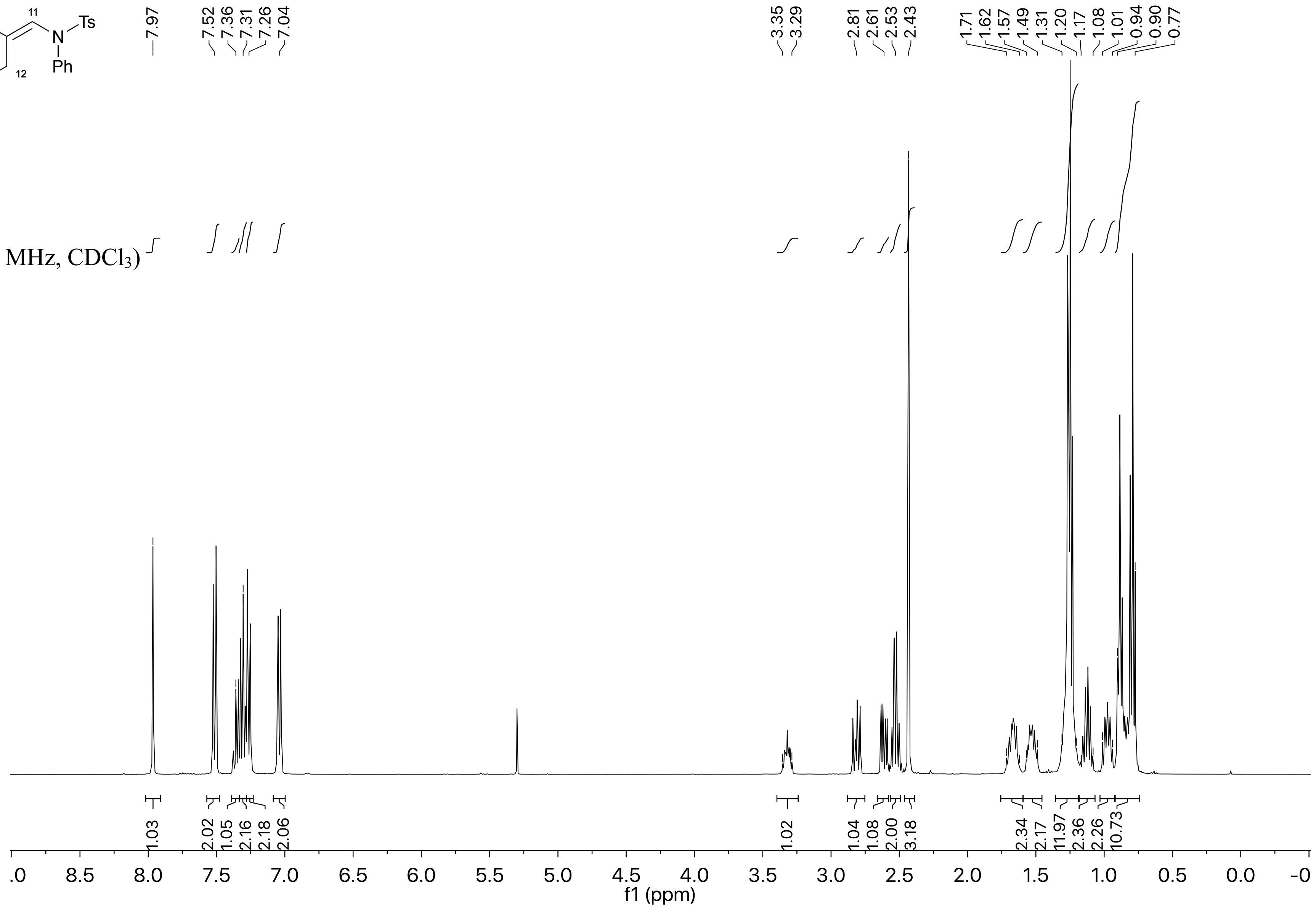

(*E*)-*N*-(4-((Ethylthio)methyl)-2-hexyl-3-oxodec-1-en-1-yl)-4-methyl-*N*-phenylbenzenesulfonamide, 3t

^{13}C NMR (101 MHz, $CDCl_3$)

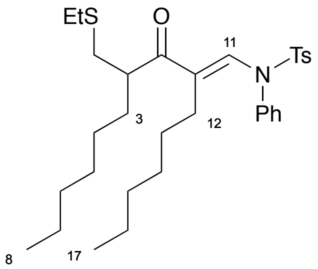

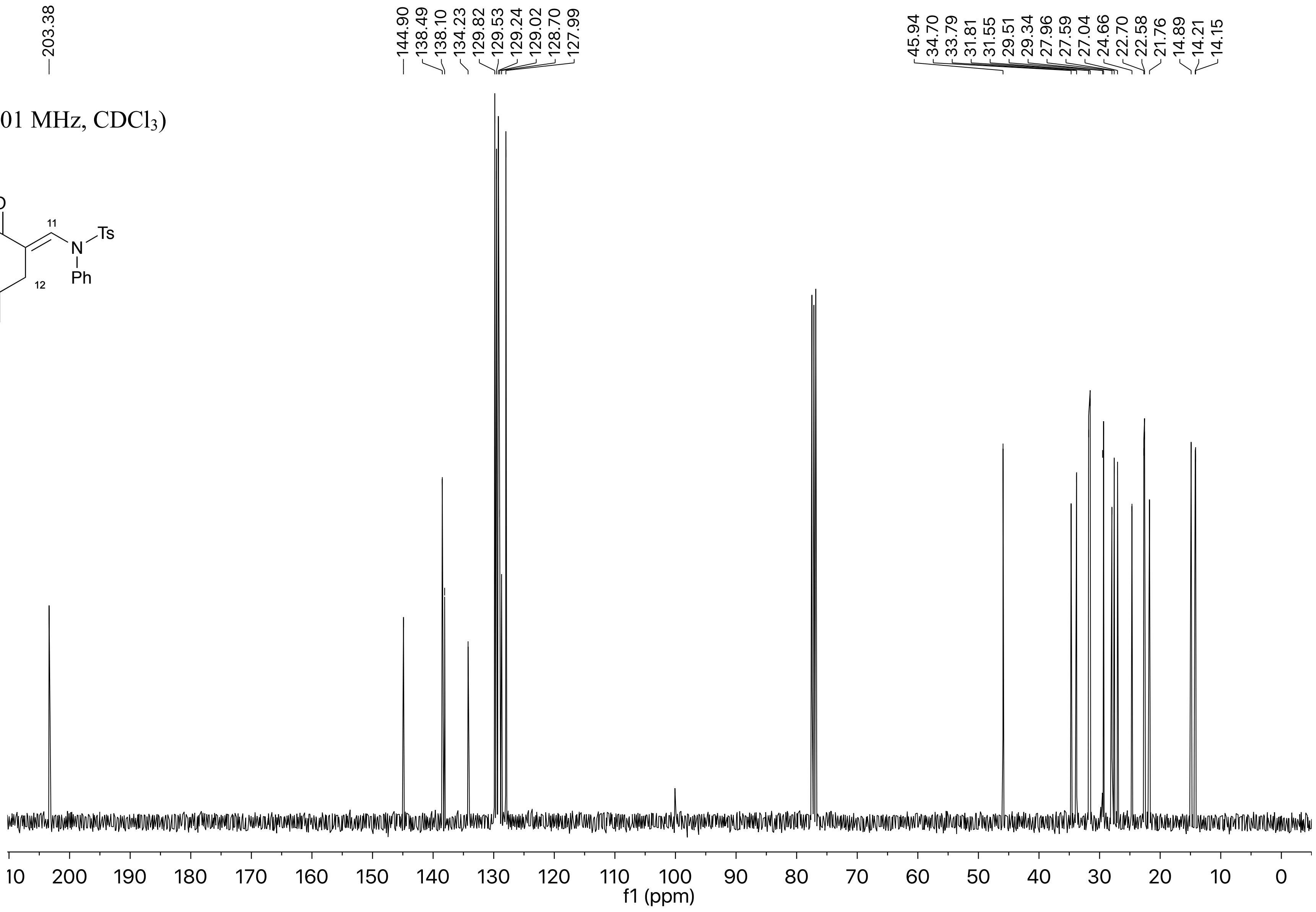

(*E*)-*N*-(2-(2-Benzyl-3-(ethylthio)propanoyl)oct-1-en-1-yl)-4-methyl-*N*-phenylbenzenesulfonamide, 3u

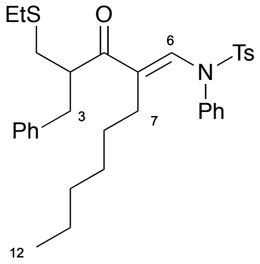

^{1}H NMR (400 MHz, $CDCl_3$)

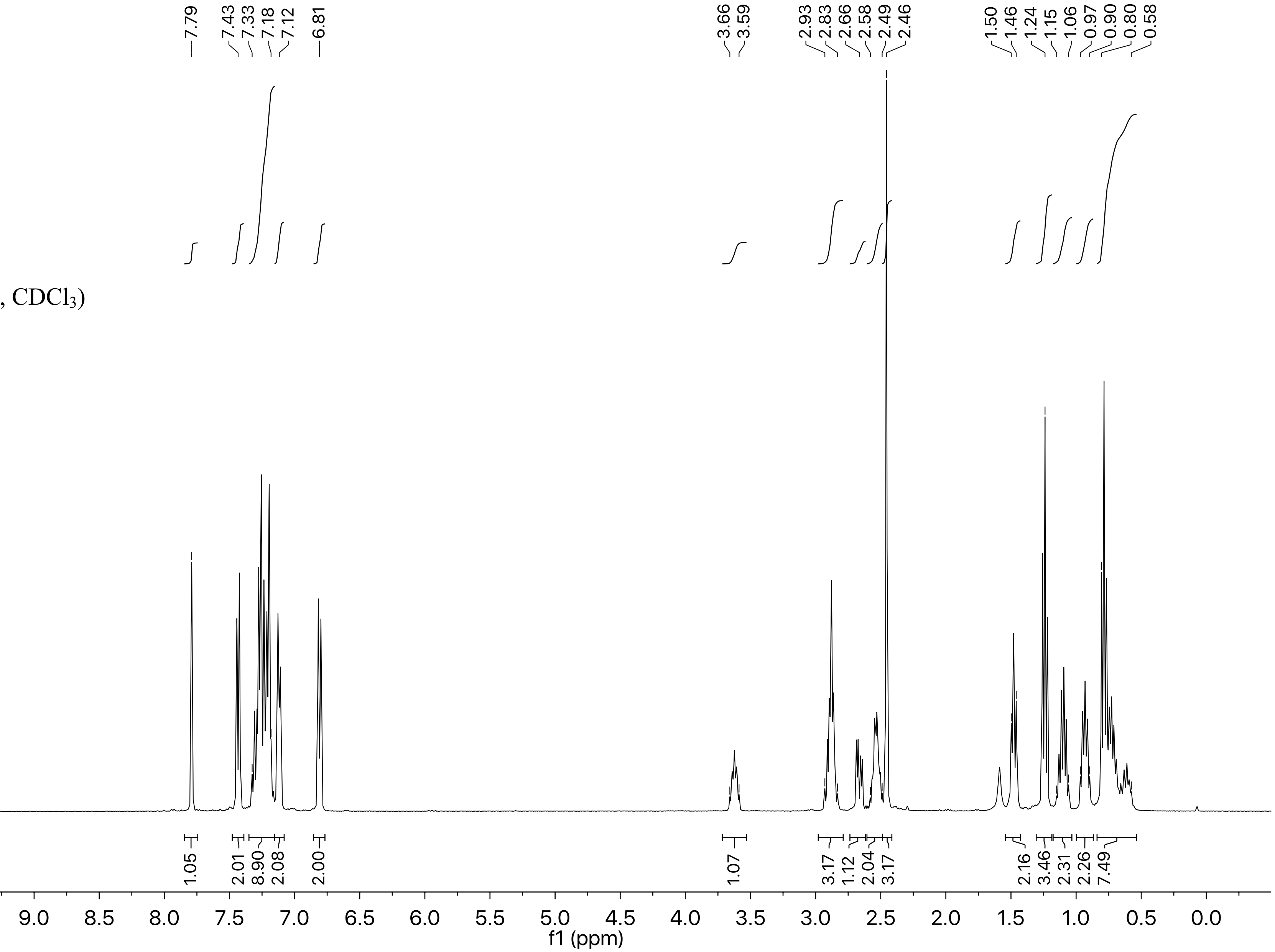

(*E*)-*N*-(2-(2-Benzyl-3-(ethylthio)propanoyl)oct-1-en-1-yl)-4-methyl-*N*-phenylbenzenesulfonamide, 3u

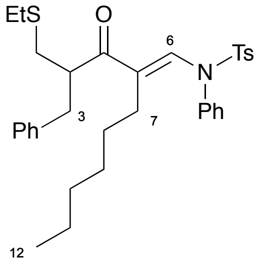

^{13}C NMR (126 MHz, $CDCl_3$)

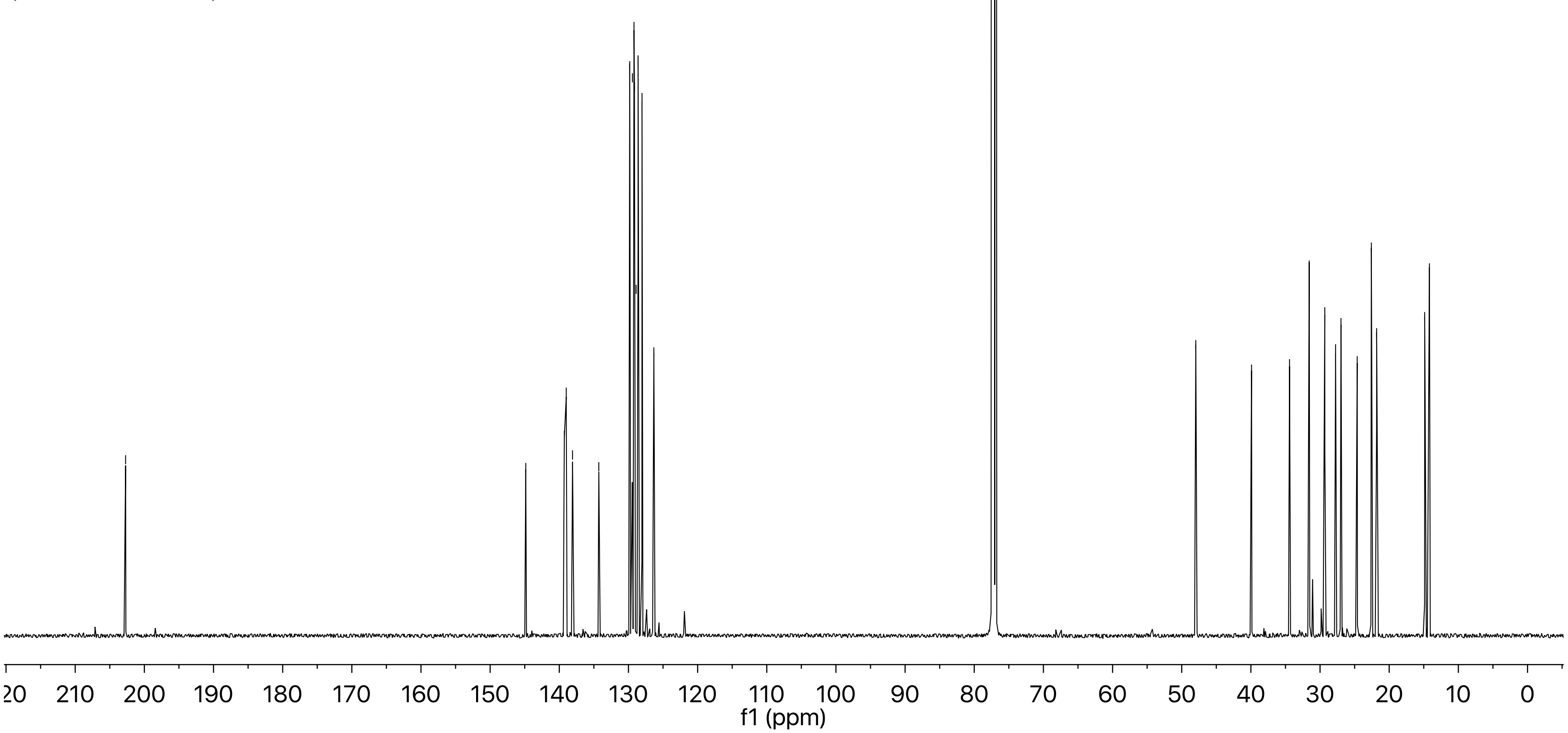

(*E*)-4-Methyl-*N*-(2-(2-(methylthio)cyclohex-1-ene-1-carbonyl)oct-1-en-1-yl)-*N*-phenylbenzenesulfonamide, 3v

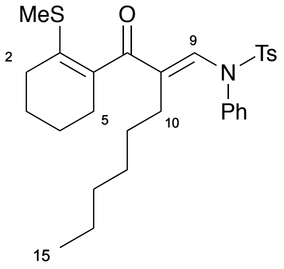

^{1}H NMR (400 MHz, $CDCl_3$)

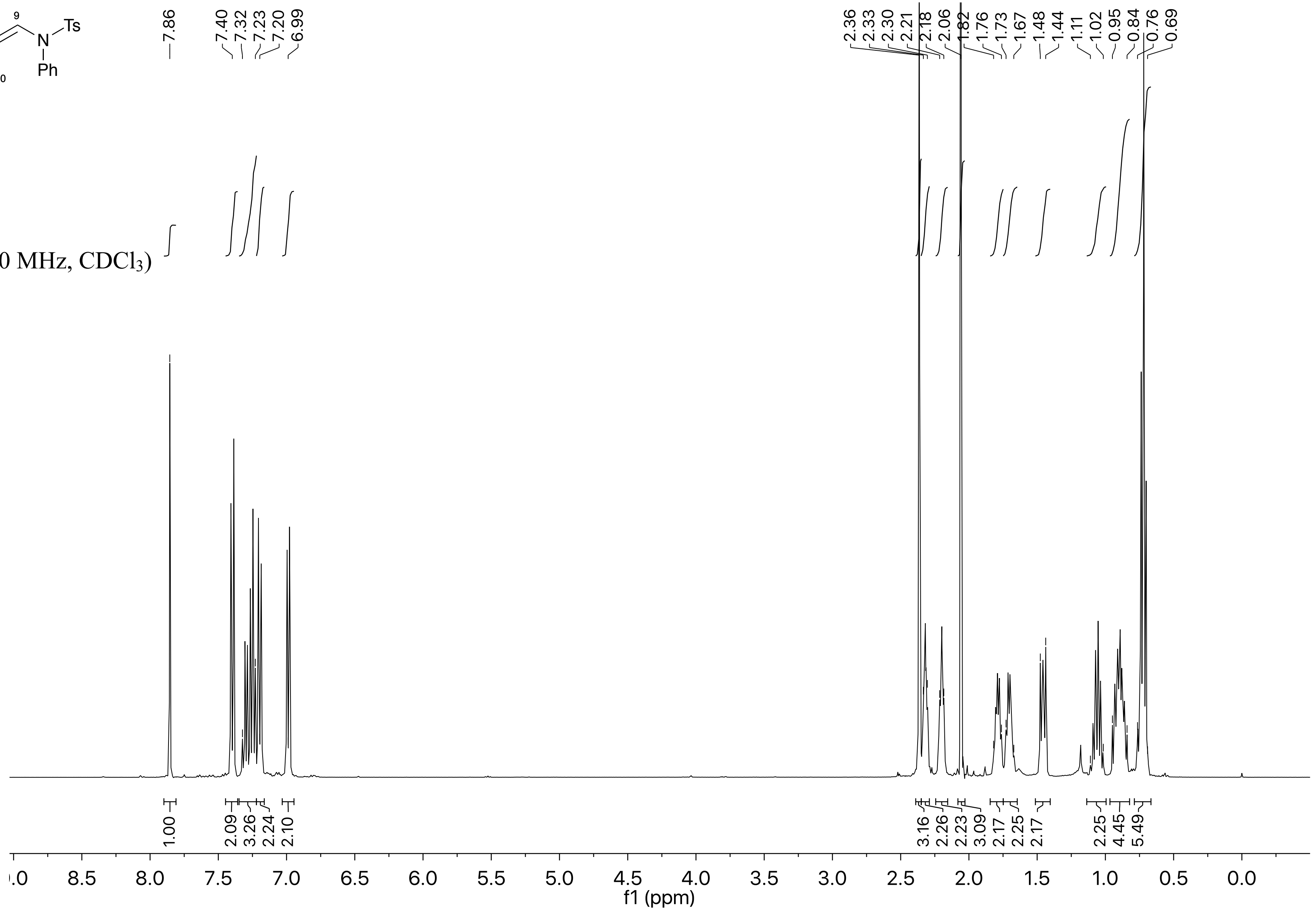

(*E*)-4-Methyl-*N*-(2-(2-(methylthio)cyclohex-1-ene-1-carbonyl)oct-1-en-1-yl)-*N*-phenylbenzenesulfonamide, 3v

^{13}C NMR (101 MHz, $CDCl_3$)

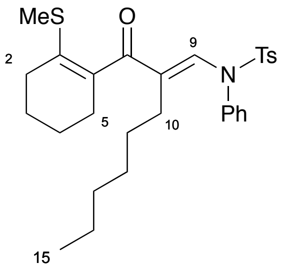

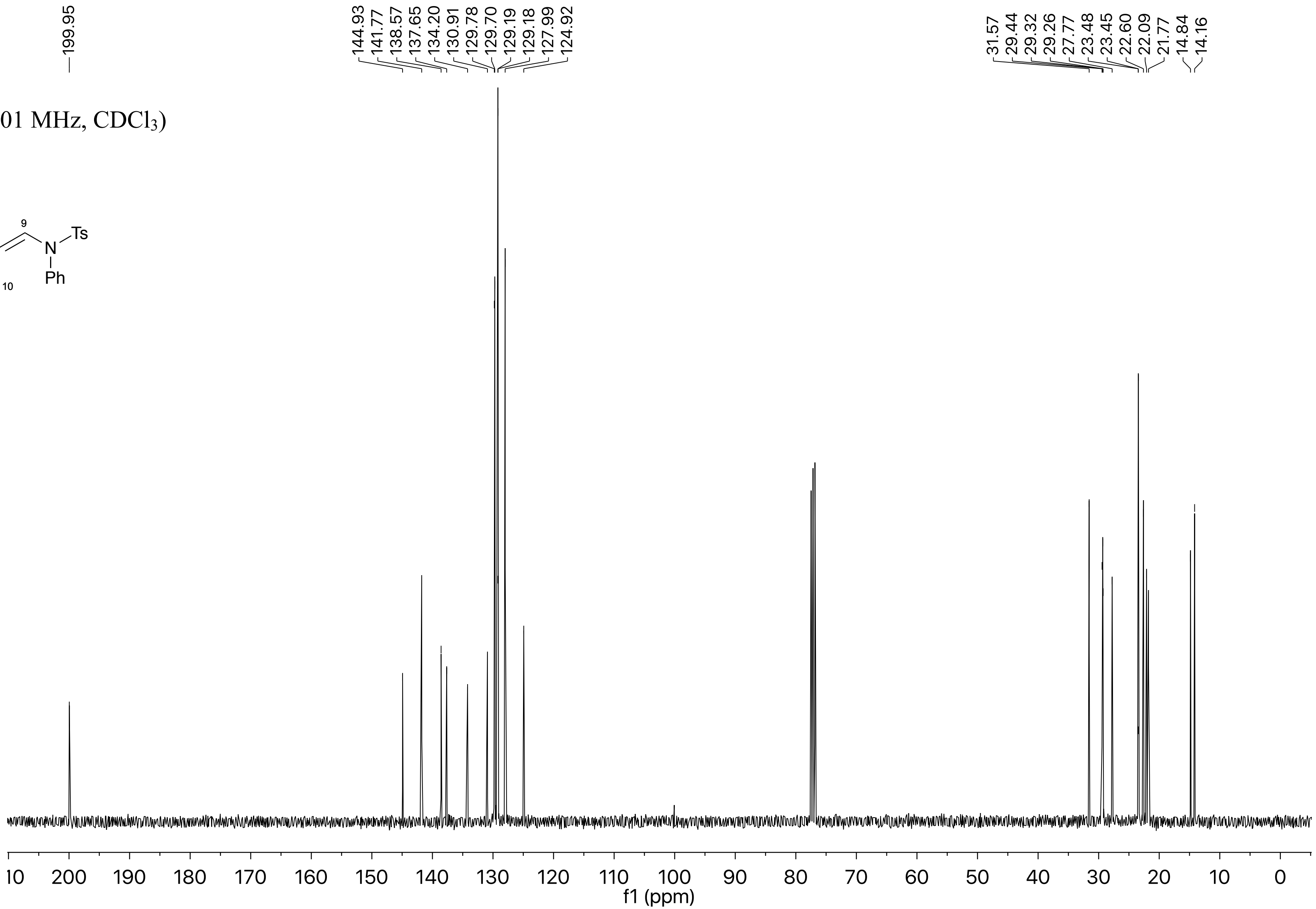

(*Z*)-4-Methyl-*N*-(1-(2-(methylthio)cyclohex-1-en-1-yl)-1-oxonon-2-en-2-yl)-*N*-phenylbenzenesulfonamide, 4v

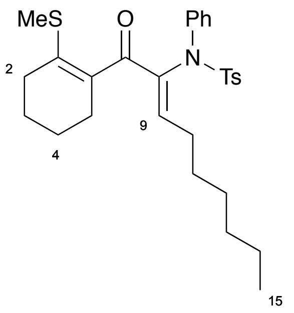

^{1}H NMR (400 MHz, $CDCl_3$)

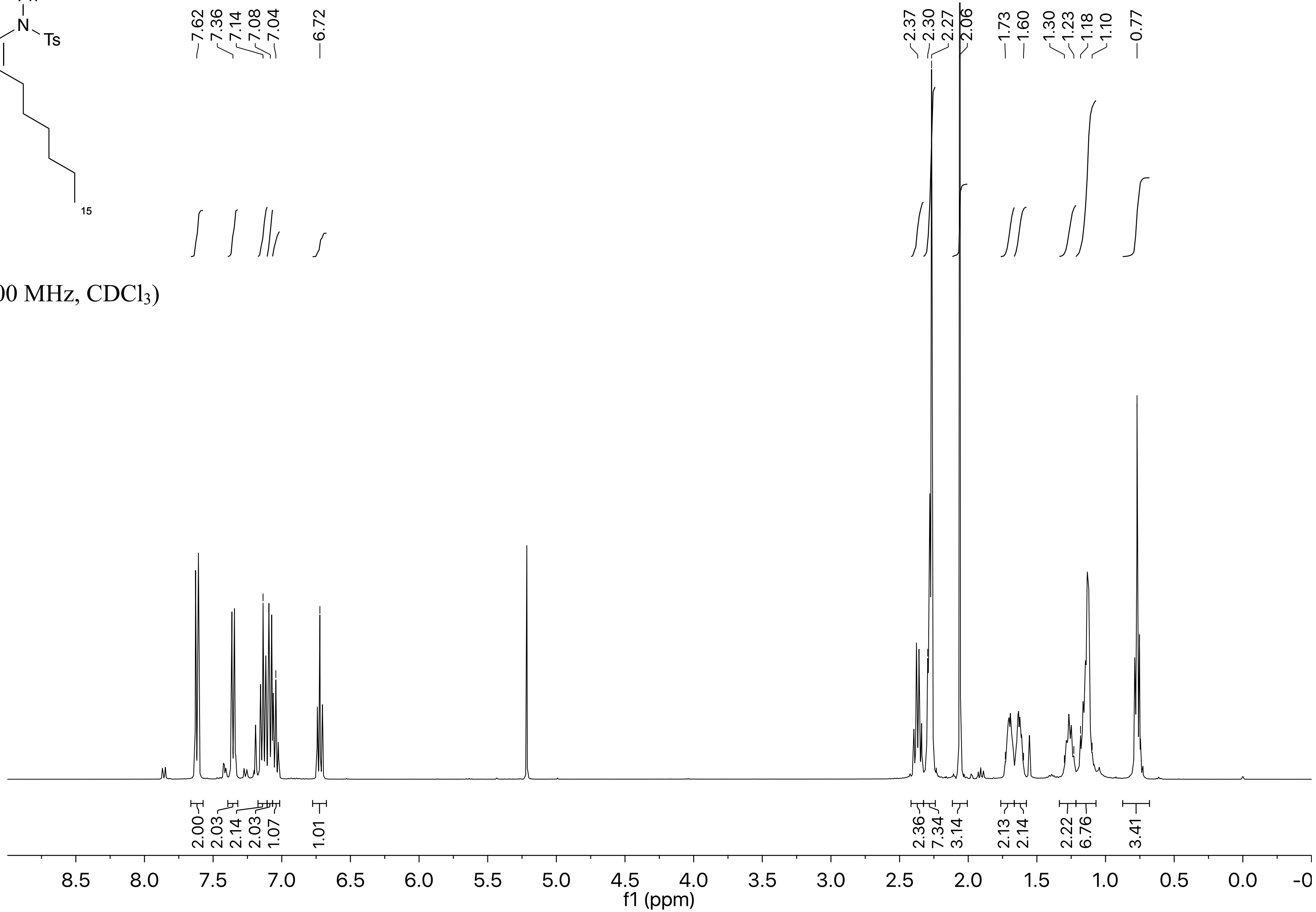

(*Z*)-4-Methyl-*N*-(1-(2-(methylthio)cyclohex-1-en-1-yl)-1-oxonon-2-en-2-yl)-*N*-phenylbenzenesulfonamide, 4v

^{13}C NMR (101 MHz, $CDCl_3$)

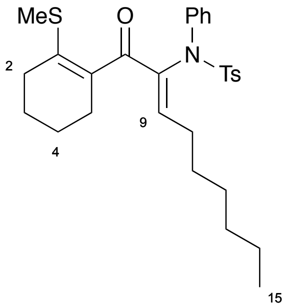

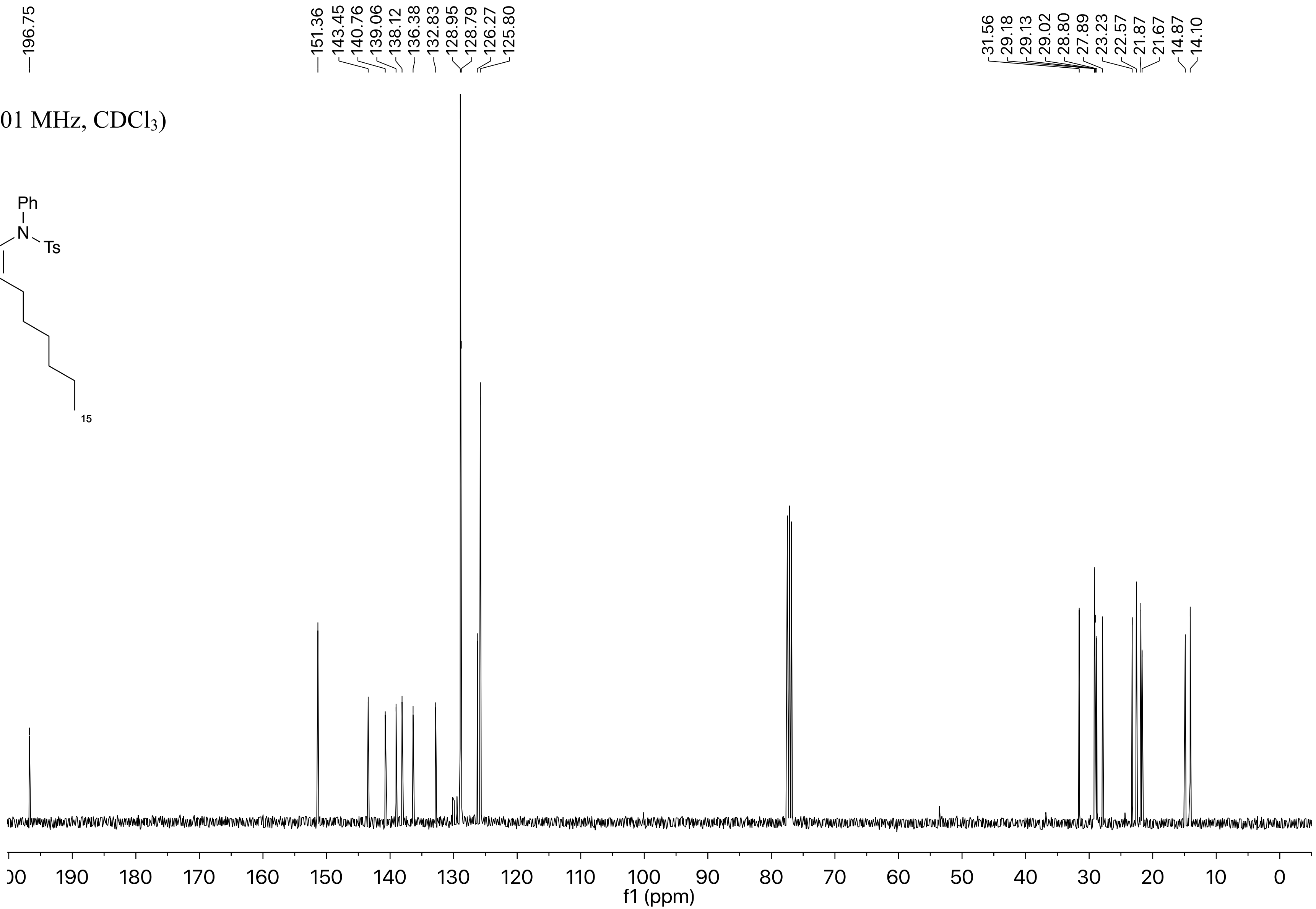

(*E*)-4-Methyl-*N*-(2-(4'-methyl-[1,1'-biphenyl]-2-carbonyl)oct-1-en-1-yl)-*N*-phenylbenzenesulfonamide, 5a

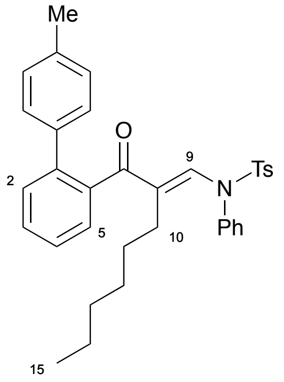

^{1}H NMR (400 MHz, $CDCl_3$)

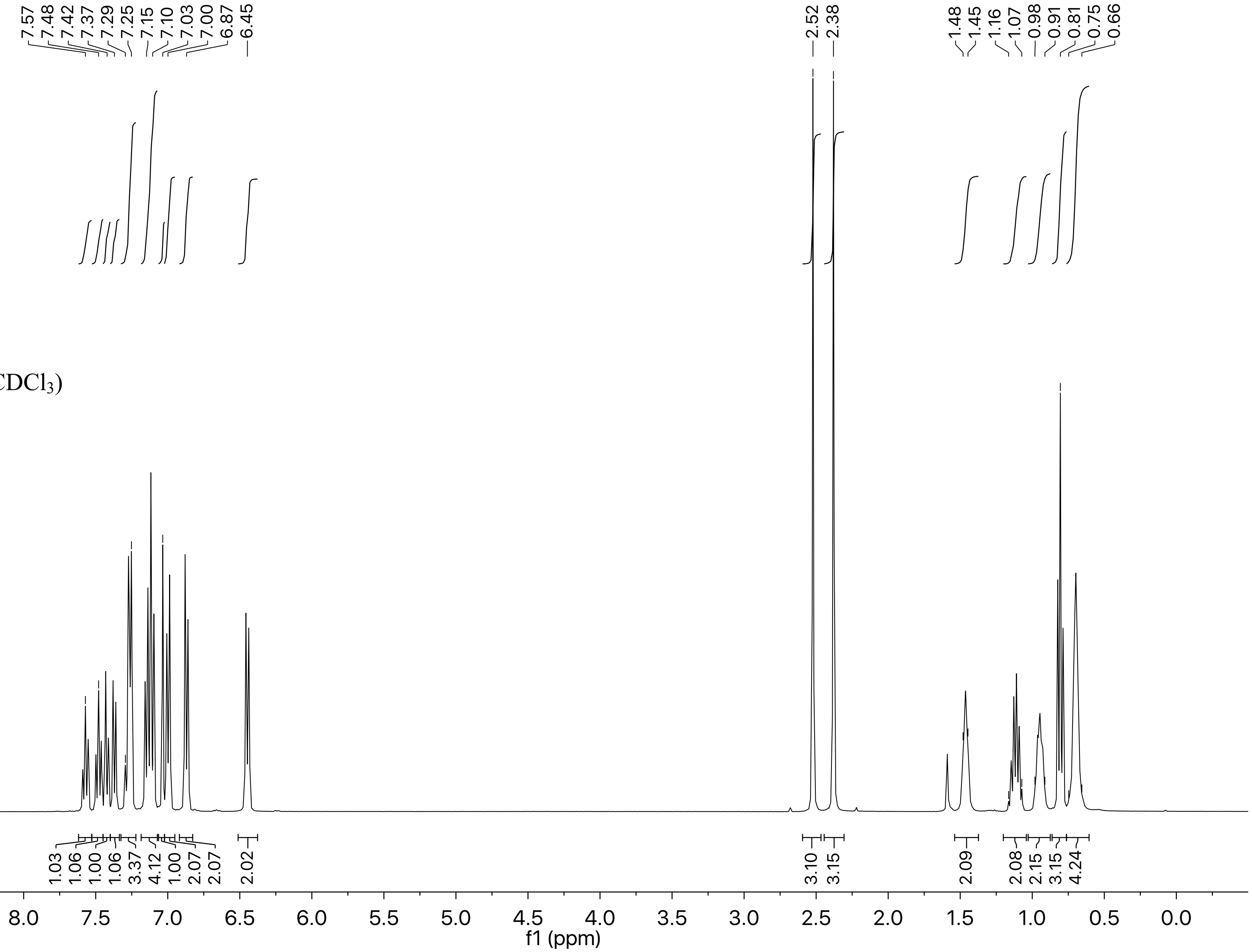

(*E*)-4-Methyl-*N*-(2-(4'-methyl-[1,1'-biphenyl]-2-carbonyl)oct-1-en-1-yl)-*N*-phenylbenzenesulfonamide, 5a

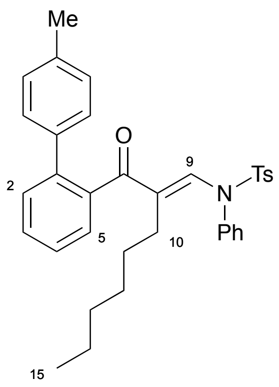

^{13}C NMR (101 MHz, $CDCl_3$)

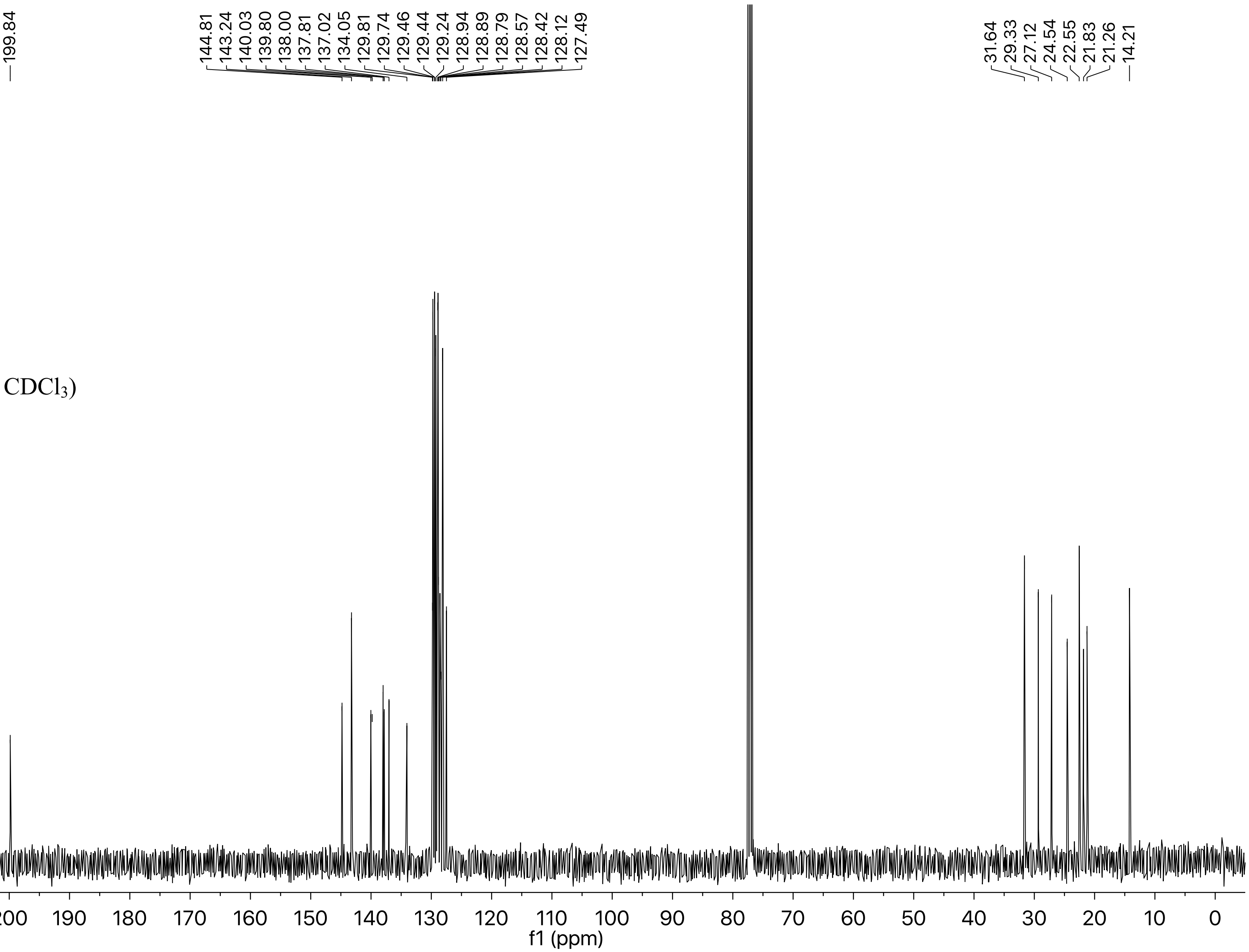

(*E*)-*N*-(2-(4'-Methoxy-[1,1'-biphenyl]-2-carbonyl)oct-1-en-1-yl)-4-methyl-*N*-phenylbenzenesulfonamide, 5b

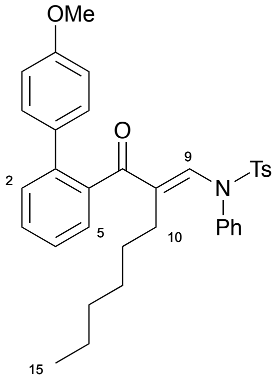

^{1}H NMR (400 MHz, $CDCl_3$)

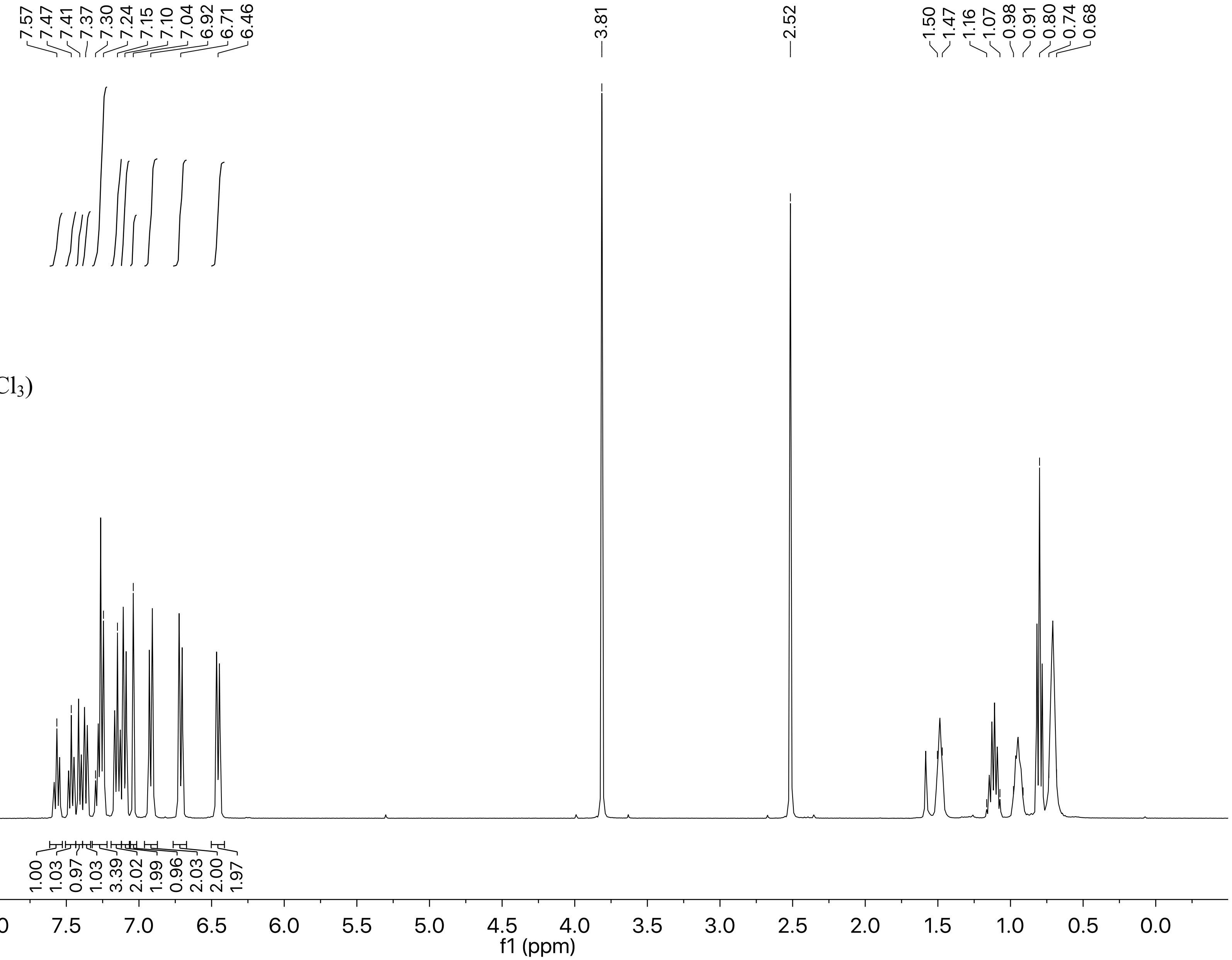

(*E*)-*N*-(2-(4'-Methoxy-[1,1'-biphenyl]-2-carbonyl)oct-1-en-1-yl)-4-methyl-*N*-phenylbenzenesulfonamide, 5b

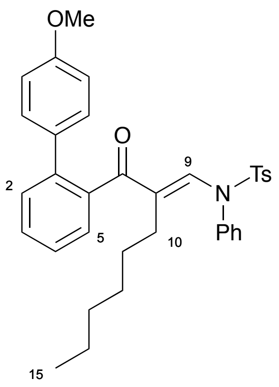

^{13}C NMR (101 MHz, $CDCl_3$)

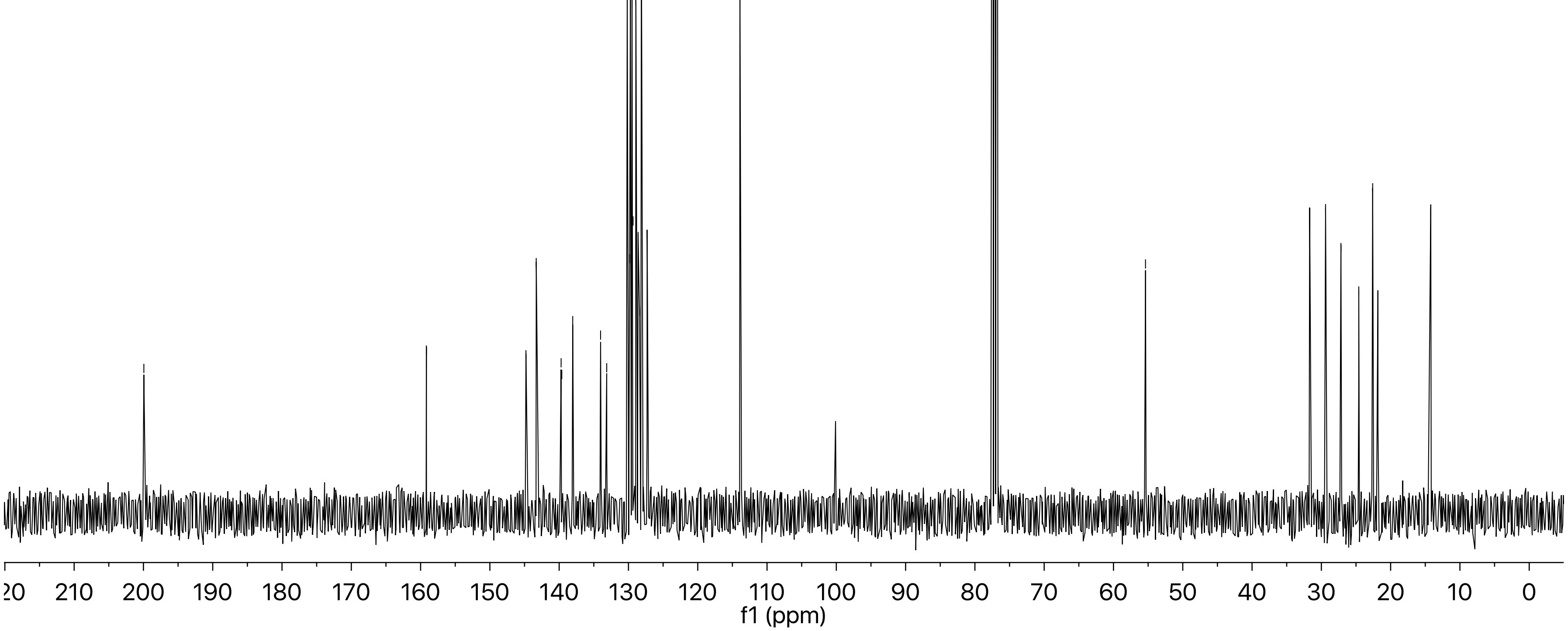

(*E*)-*N*-(2-(4'-Acetyl-[1,1'-biphenyl]-2-carbonyl)oct-1-en-1-yl)-4-methyl-*N*-phenylbenzenesulfonamide, 5c

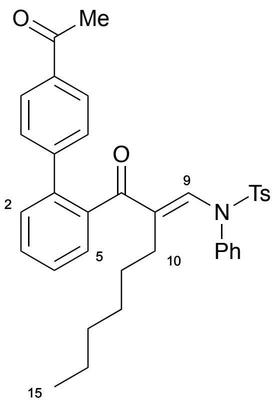

^{1}H NMR (500 MHz, $CDCl_3$)

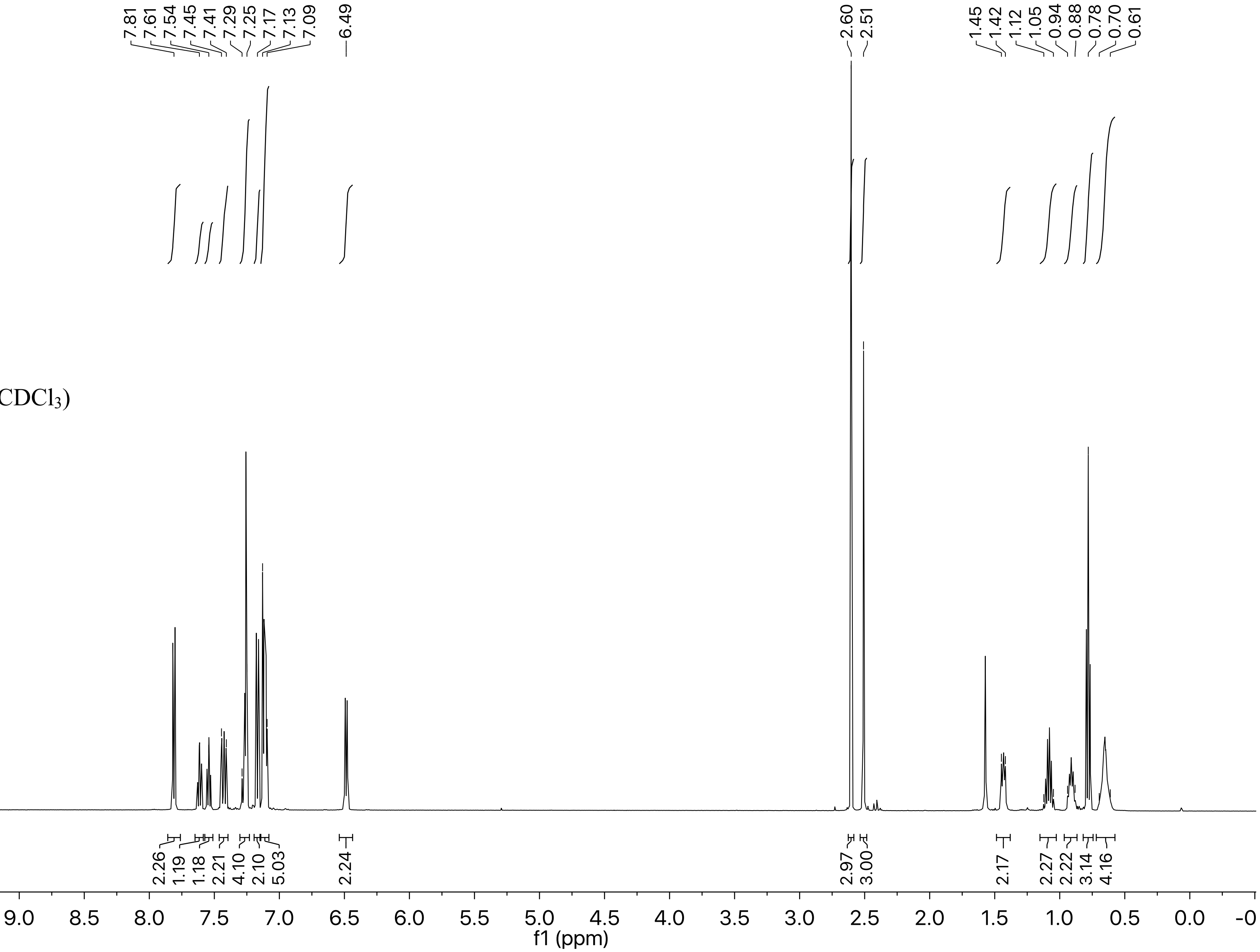

(*E*)-*N*-(2-(4'-Acetyl-[1,1'-biphenyl]-2-carbonyl)oct-1-en-1-yl)-4-methyl-*N*-phenylbenzenesulfonamide, 5c

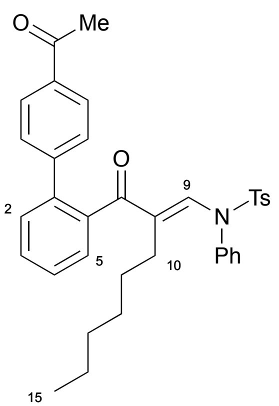

^{13}C NMR (126 MHz, $CDCl_3$)

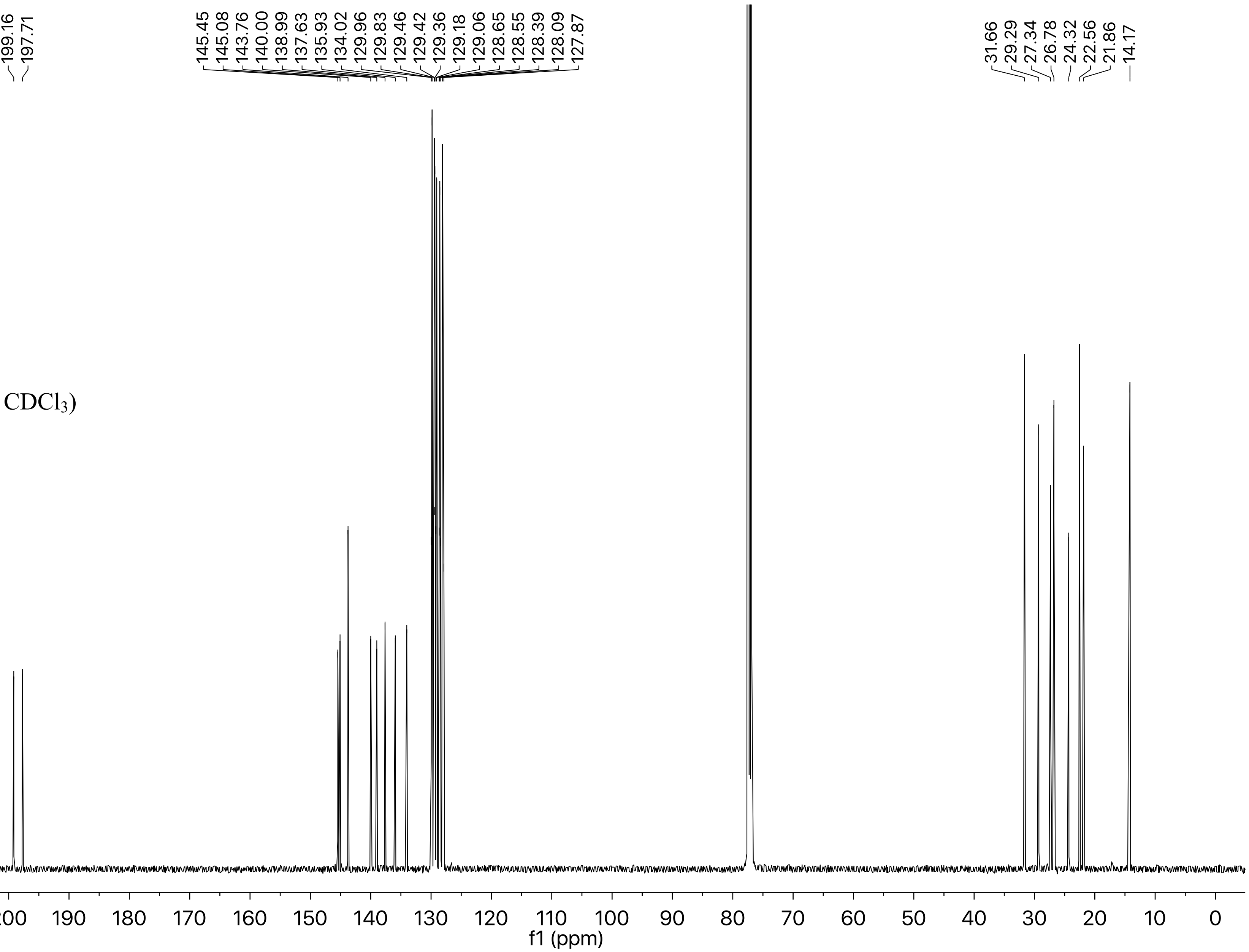

4-Hexyl-5-(2-(methylthio)phenyl)isoxazole, 6a

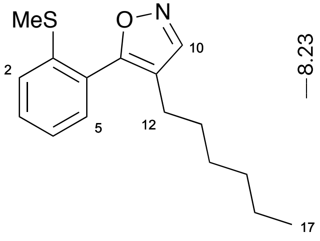

^{1}H NMR (400 MHz, $CDCl_3$)

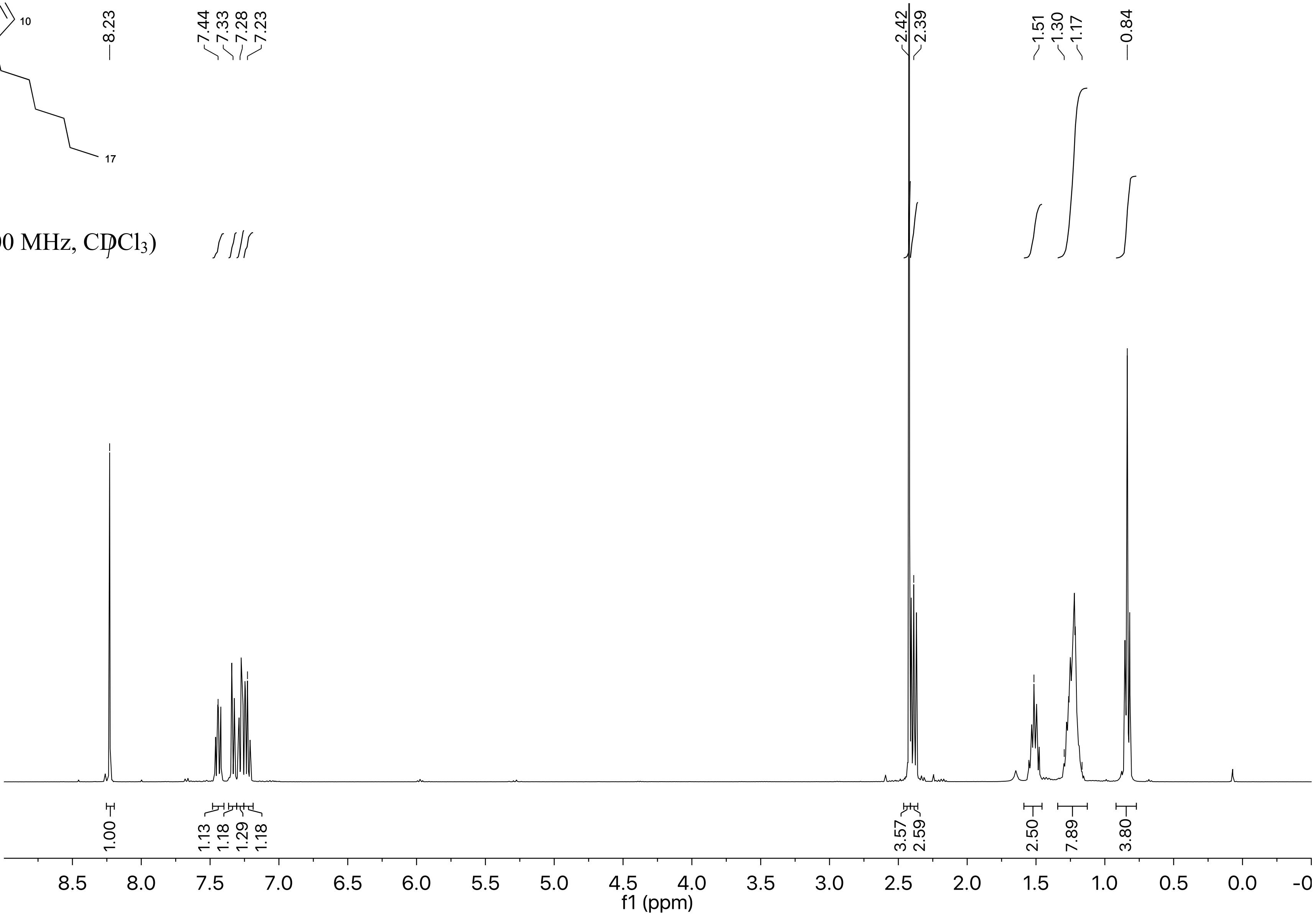

4-Hexyl-5-(2-(methylthio)phenyl)isoxazole, 6a

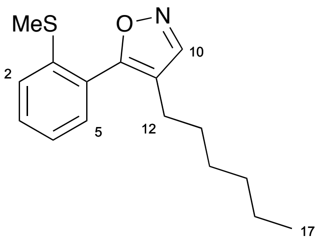

^{13}C NMR (101 MHz, $CDCl_3$)

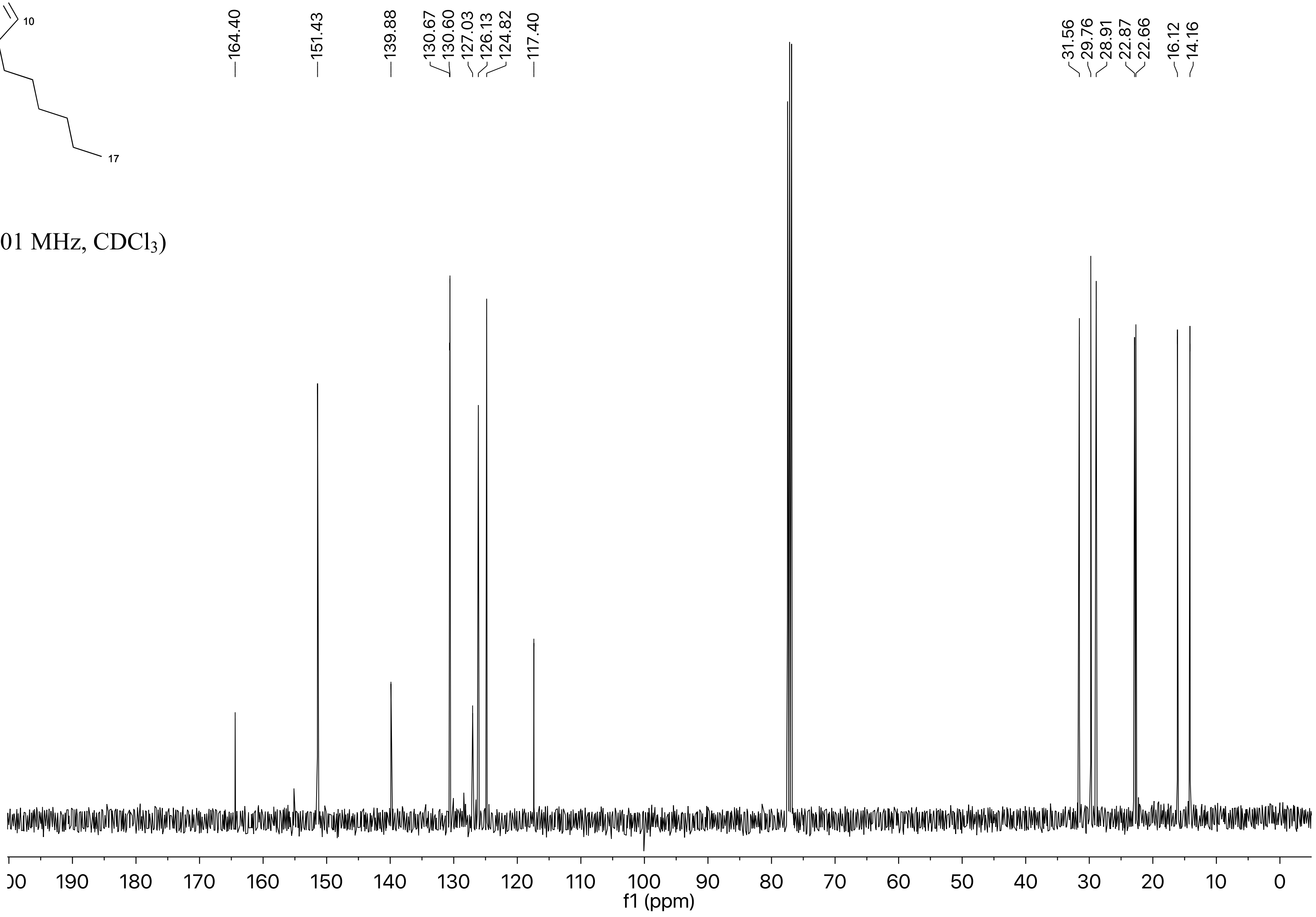

4-(3-Chloropropyl)-5-(2-(methylthio)phenyl)isoxazole, 6e

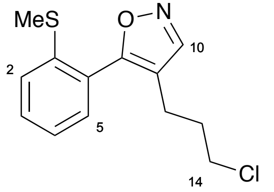

^{1}H NMR (400 MHz, $CDCl_3$)

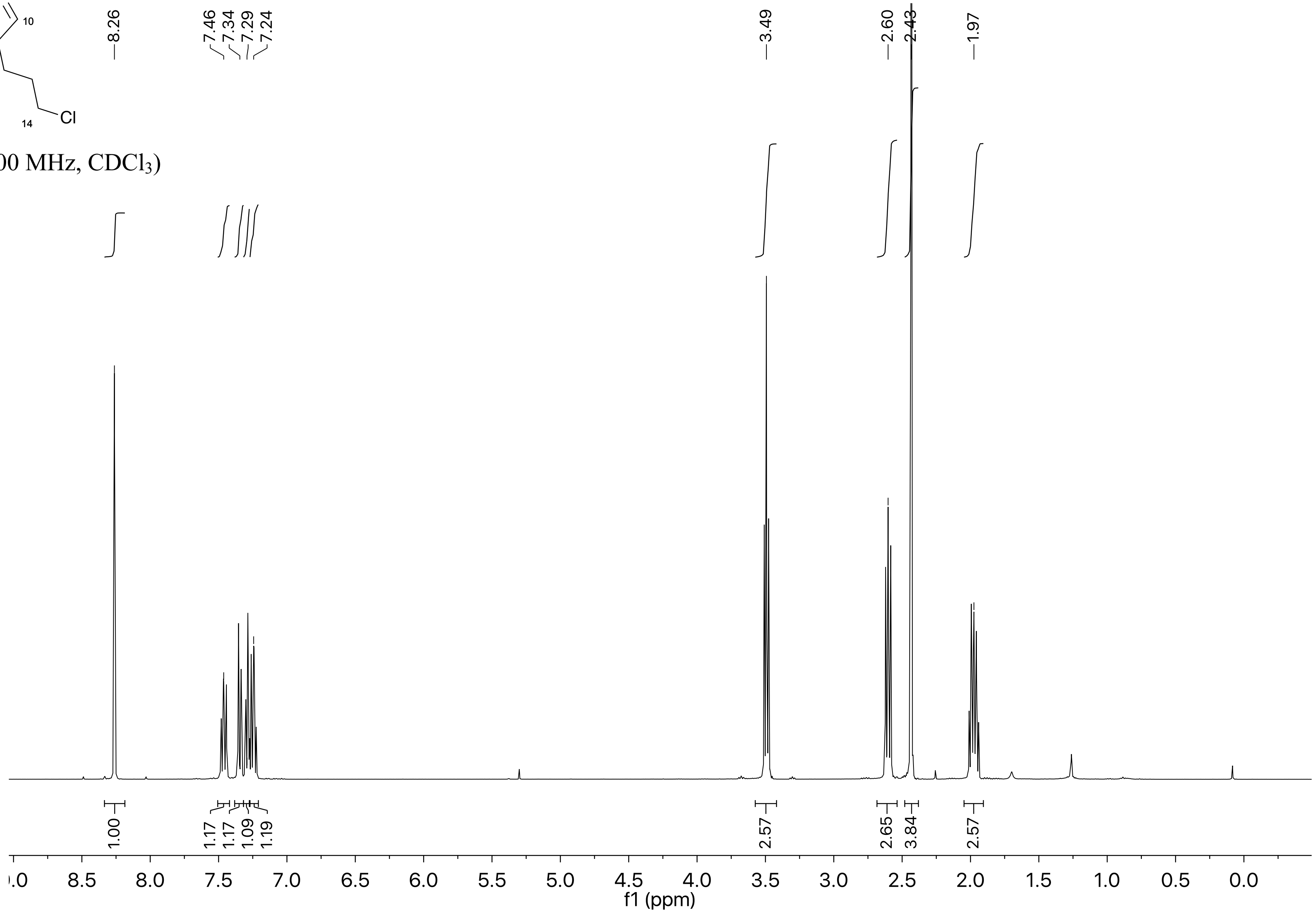

4-(3-Chloropropyl)-5-(2-(methylthio)phenyl)isoxazole, 6e

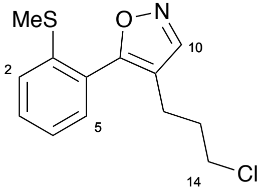

^{13}C NMR (101 MHz, $CDCl_3$)

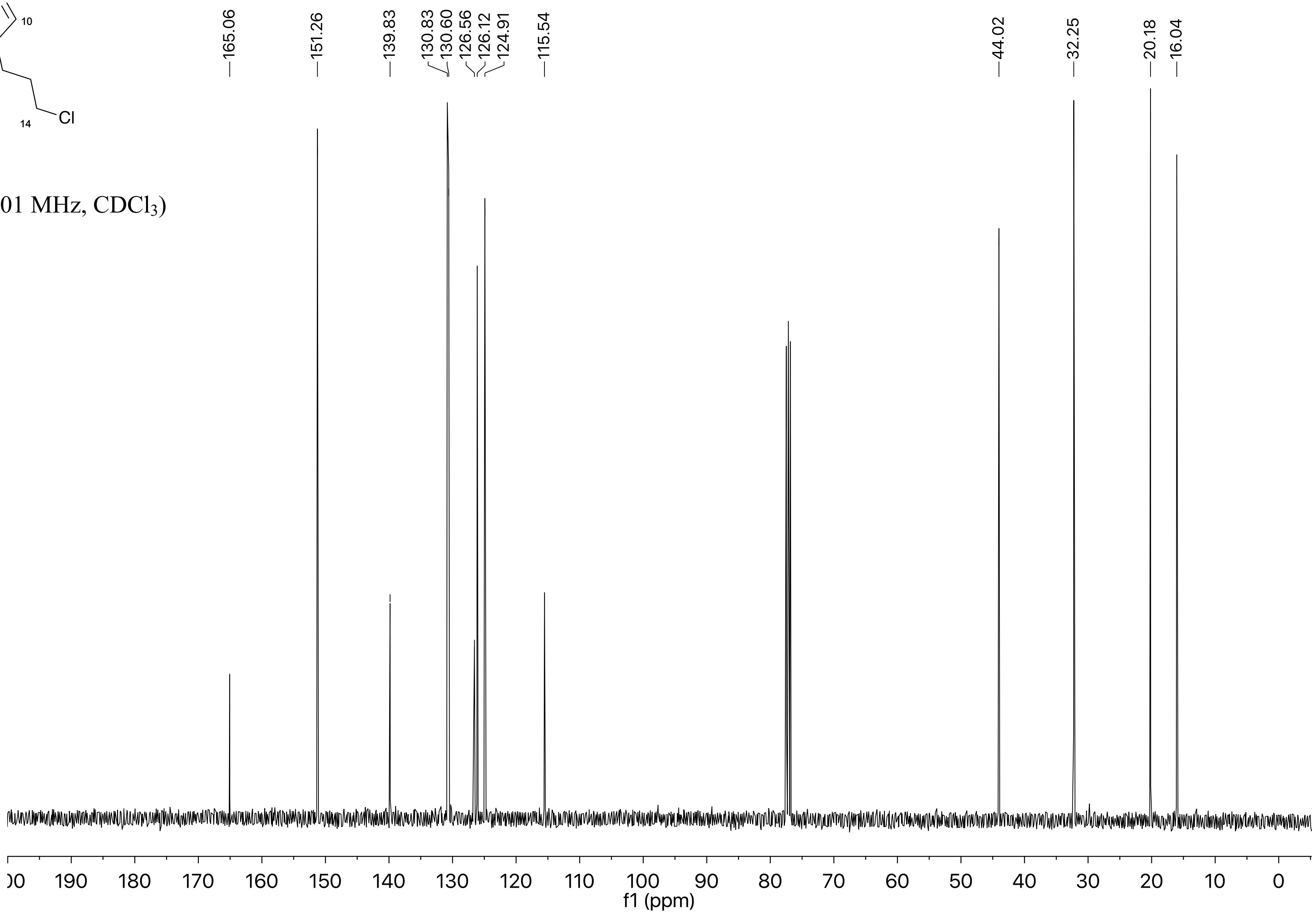

3-(5-(2-(Methylthio)phenyl)isoxazol-4-yl)propan-1-ol, 6f

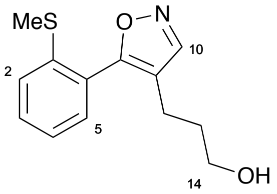

^{1}H NMR (500 MHz, $CDCl_3$)

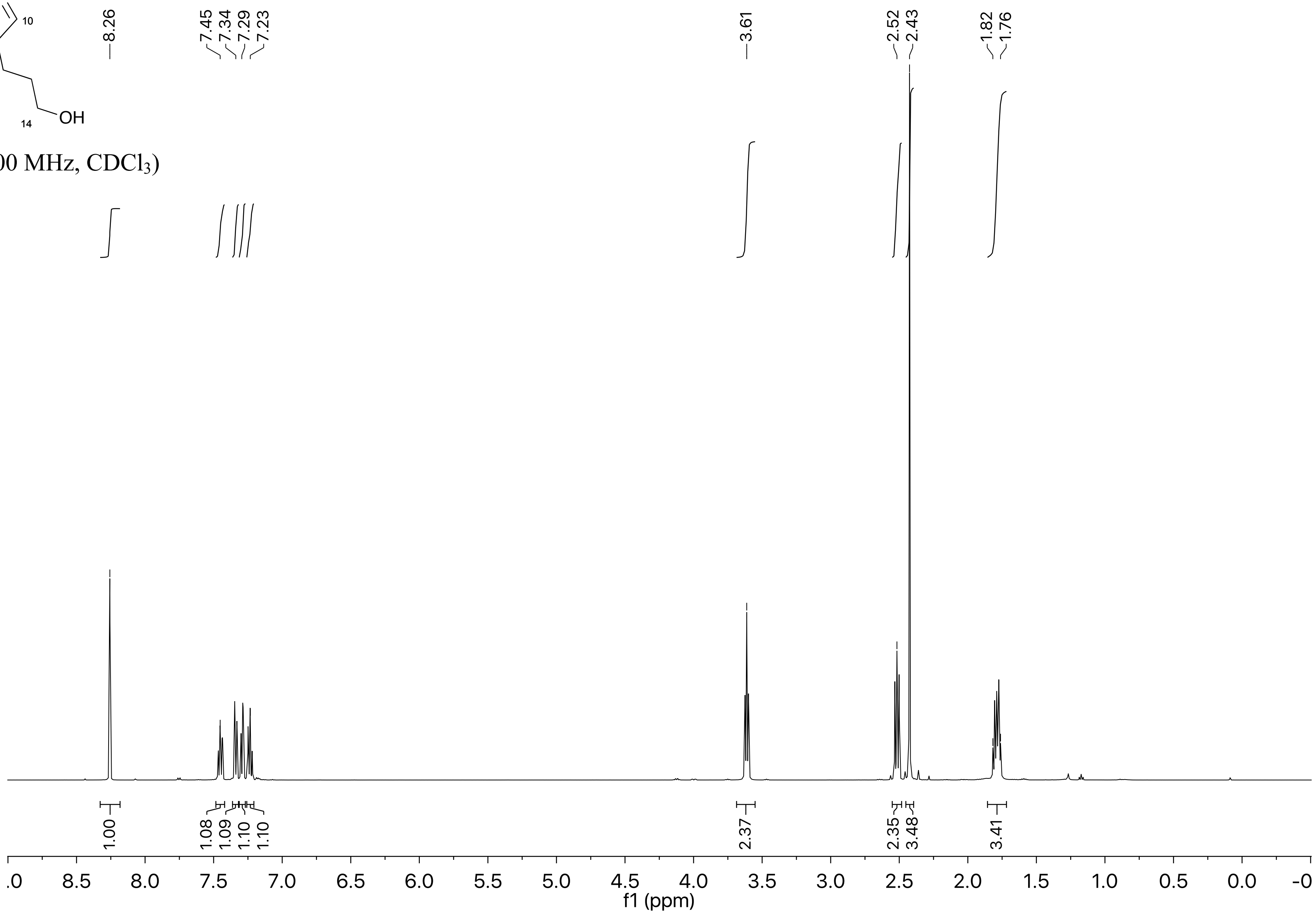

3-(5-(2-(Methylthio)phenyl)isoxazol-4-yl)propan-1-ol, 6f

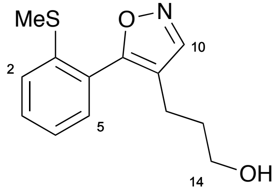

^{13}**C NMR** (126 MHz, $CDCl_3$)

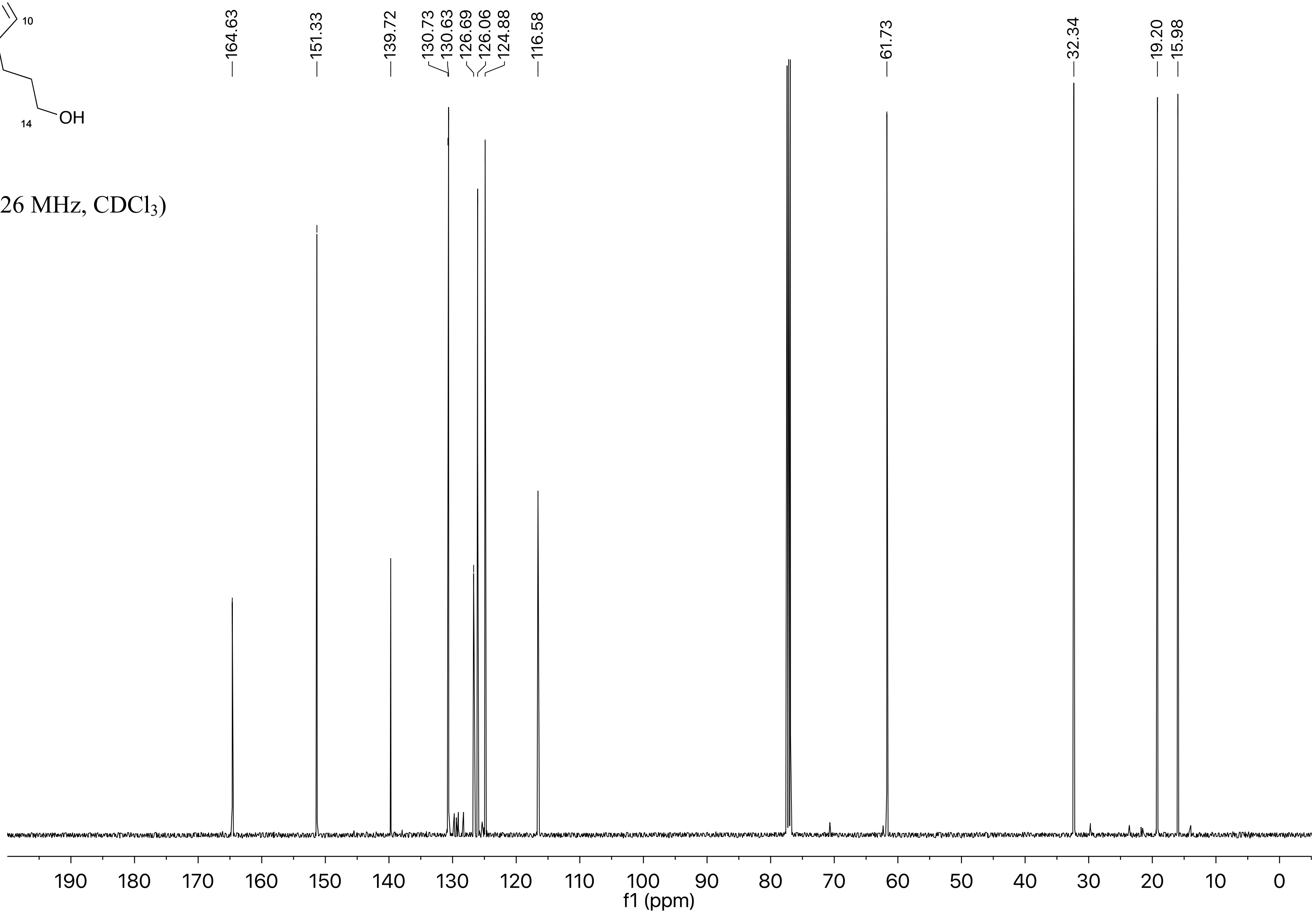

5-(2-(Methylthio)phenyl)-4-phenylisoxazole, 6g

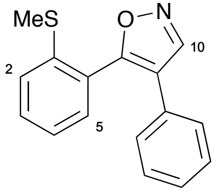

^{1}H NMR (400 MHz, $CDCl_3$)

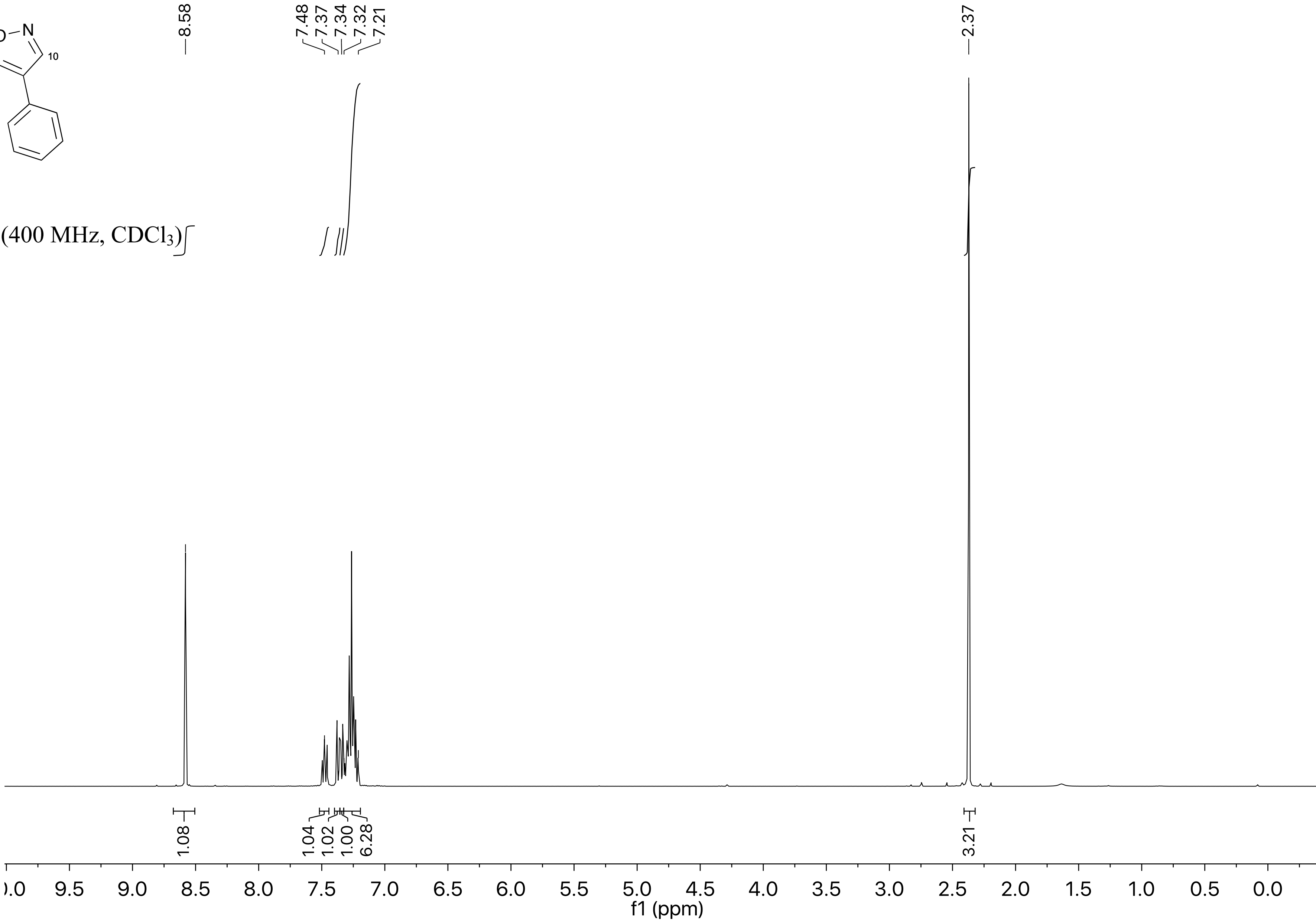

5-(2-(Methylthio)phenyl)-4-phenylisoxazole, 6g

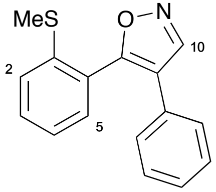

^{13}C NMR (101 MHz, $CDCl_3$)

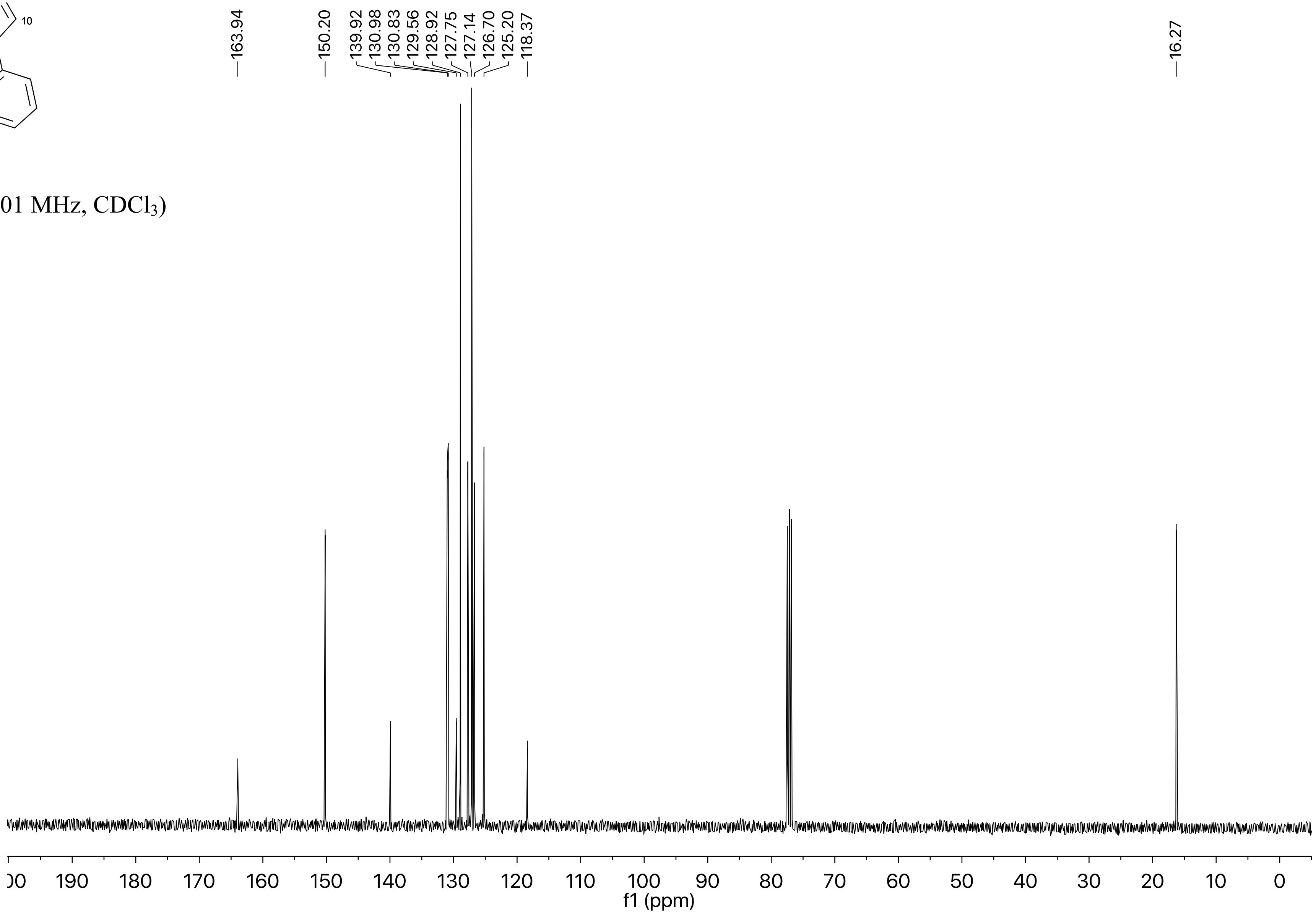

5-(2-(Dimethylsulfonio)phenyl)-2-methyl-4-phenylisoxazol-2-ium *bis*-tetrafluoroborate, S1

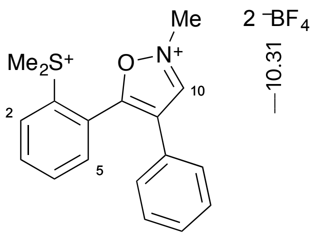

^{1}H NMR (500 MHz, $CDCl_3$)

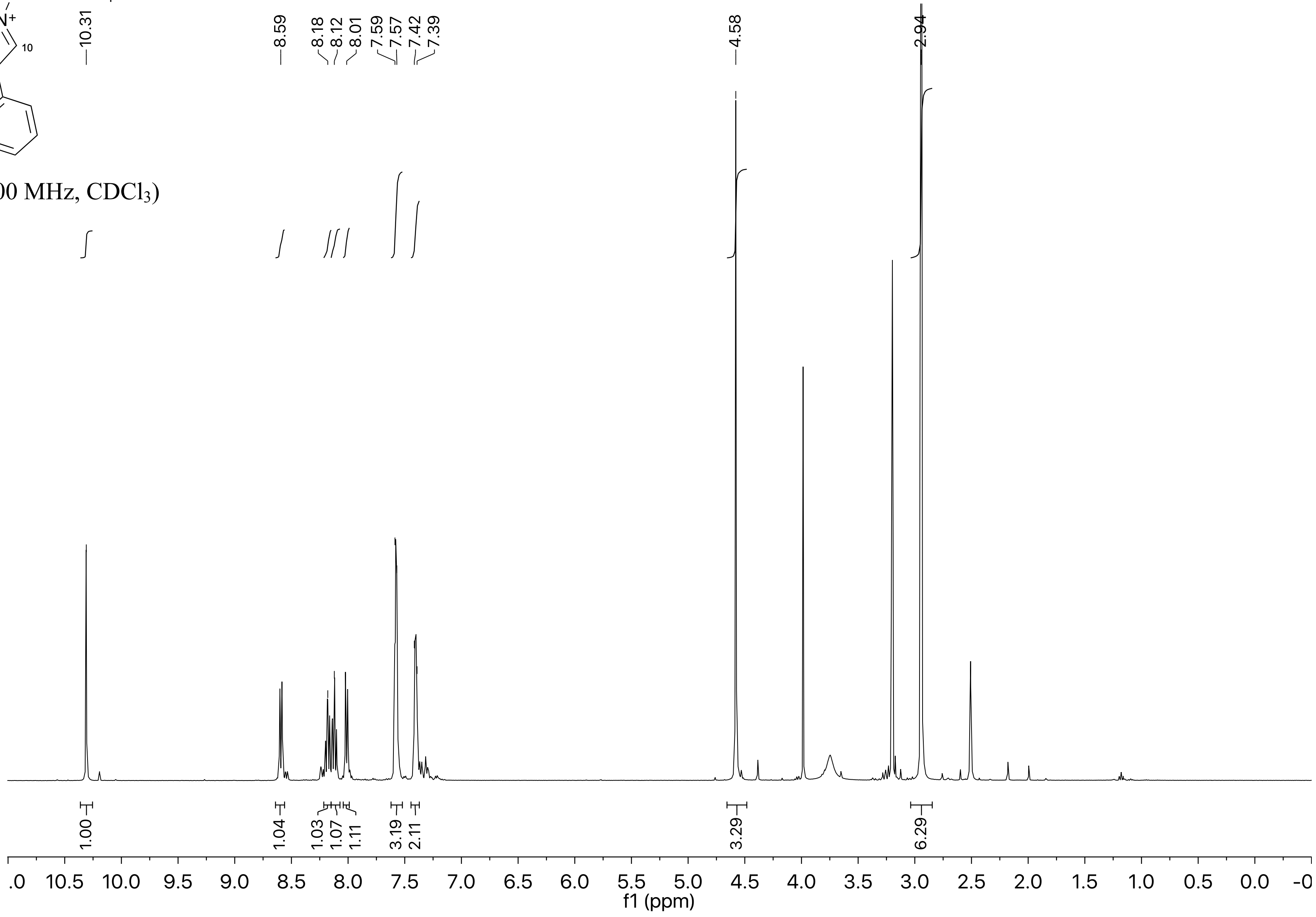

5-(2-(Dimethylsulfonio)phenyl)-2-methyl-4-phenylisoxazol-2-ium *bis*-tetrafluoroborate, S1

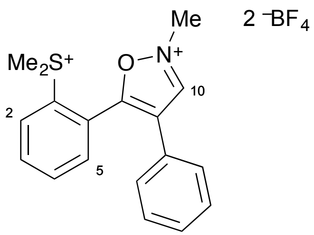

^{13}C NMR (125 MHz, $CDCl_3$)

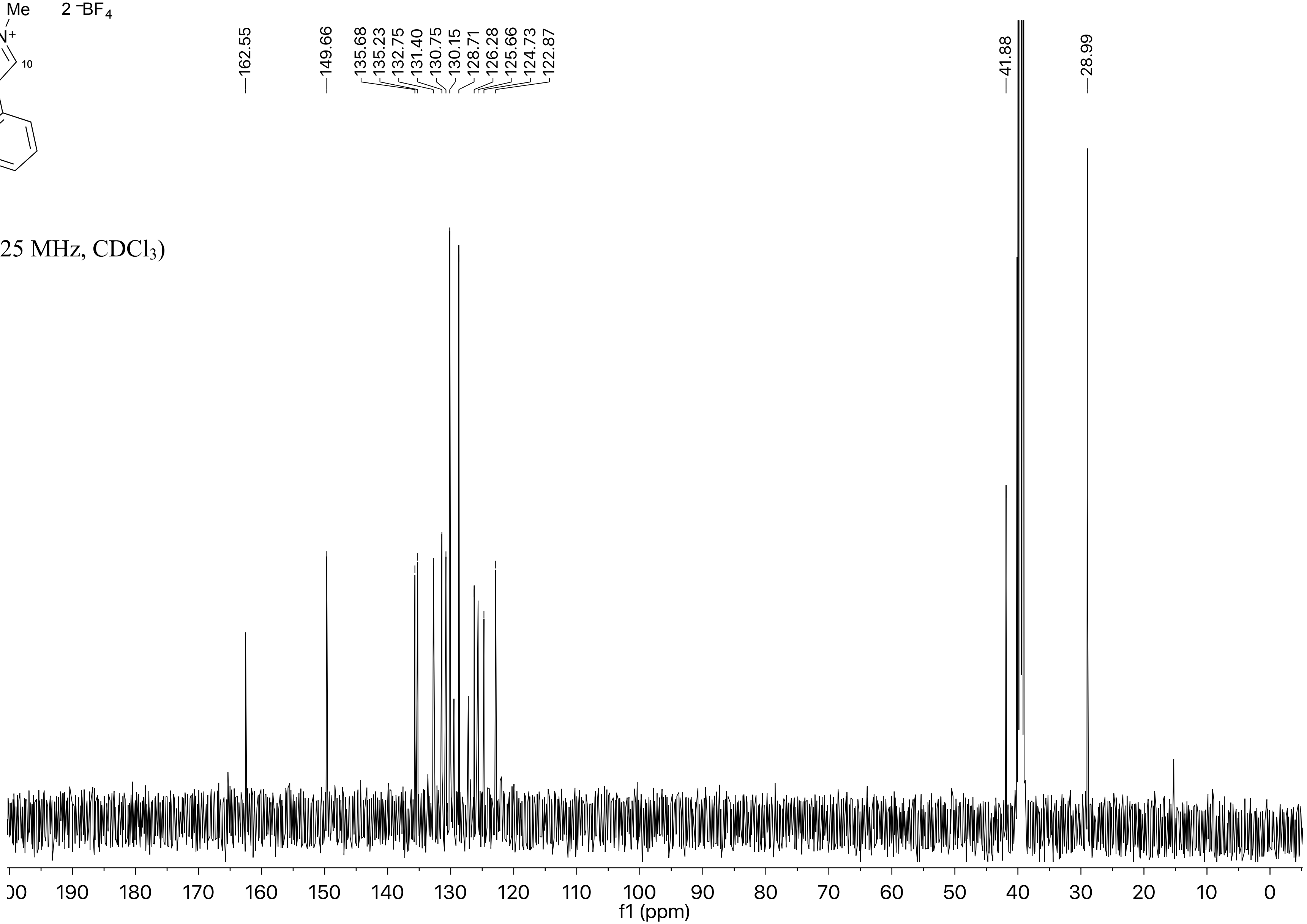

5-(2-(Dimethylsulfonio)phenyl)-2-methyl-4-phenylisoxazol-2-ium *bis*-tetrafluoroborate, S1

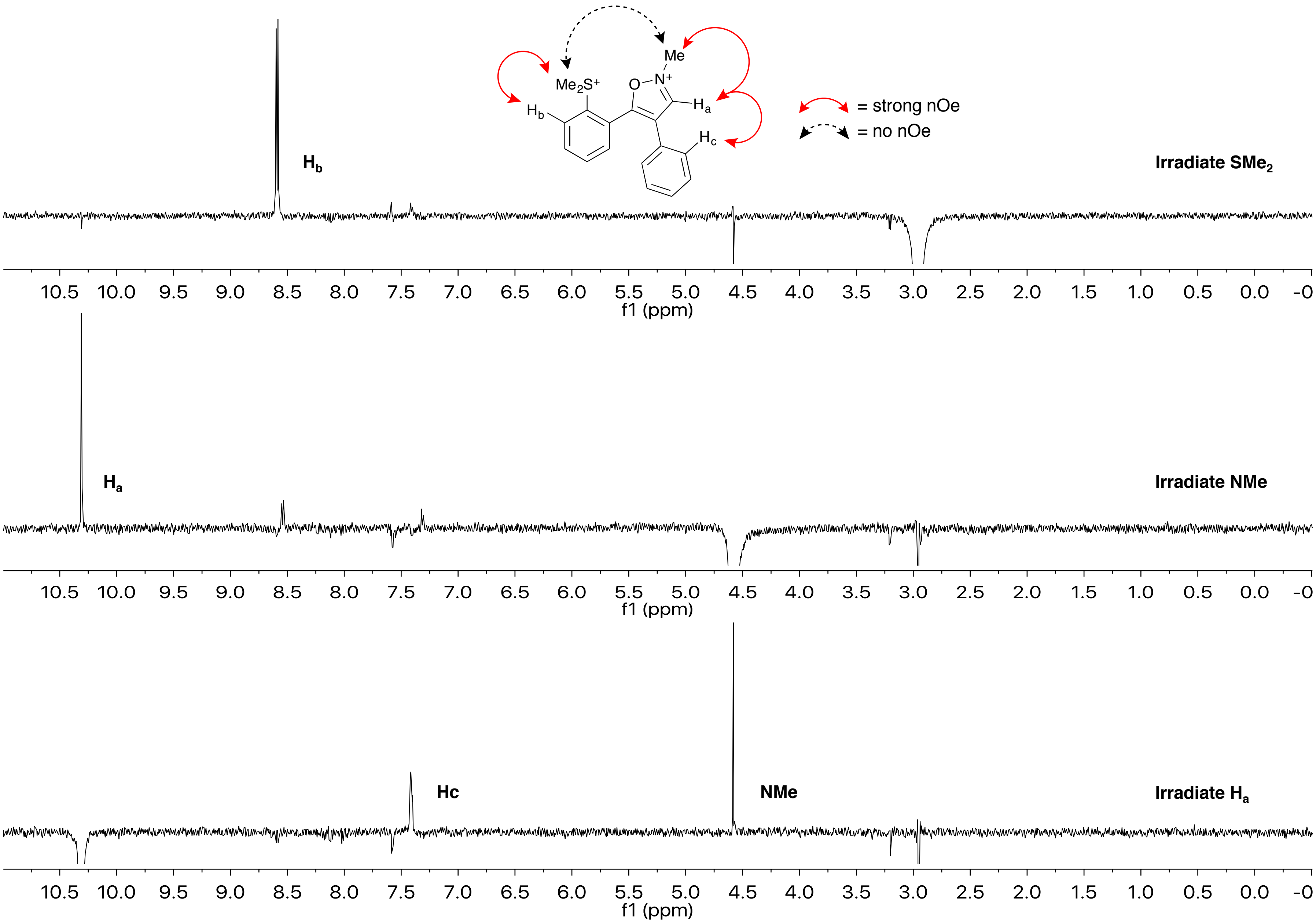

4-(4-Methoxyphenyl)-5-(2-(methylthio)phenyl)isoxazole, 6h

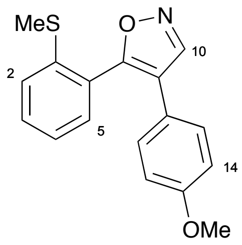

^{1}H NMR (400 MHz, $CDCl_3$)

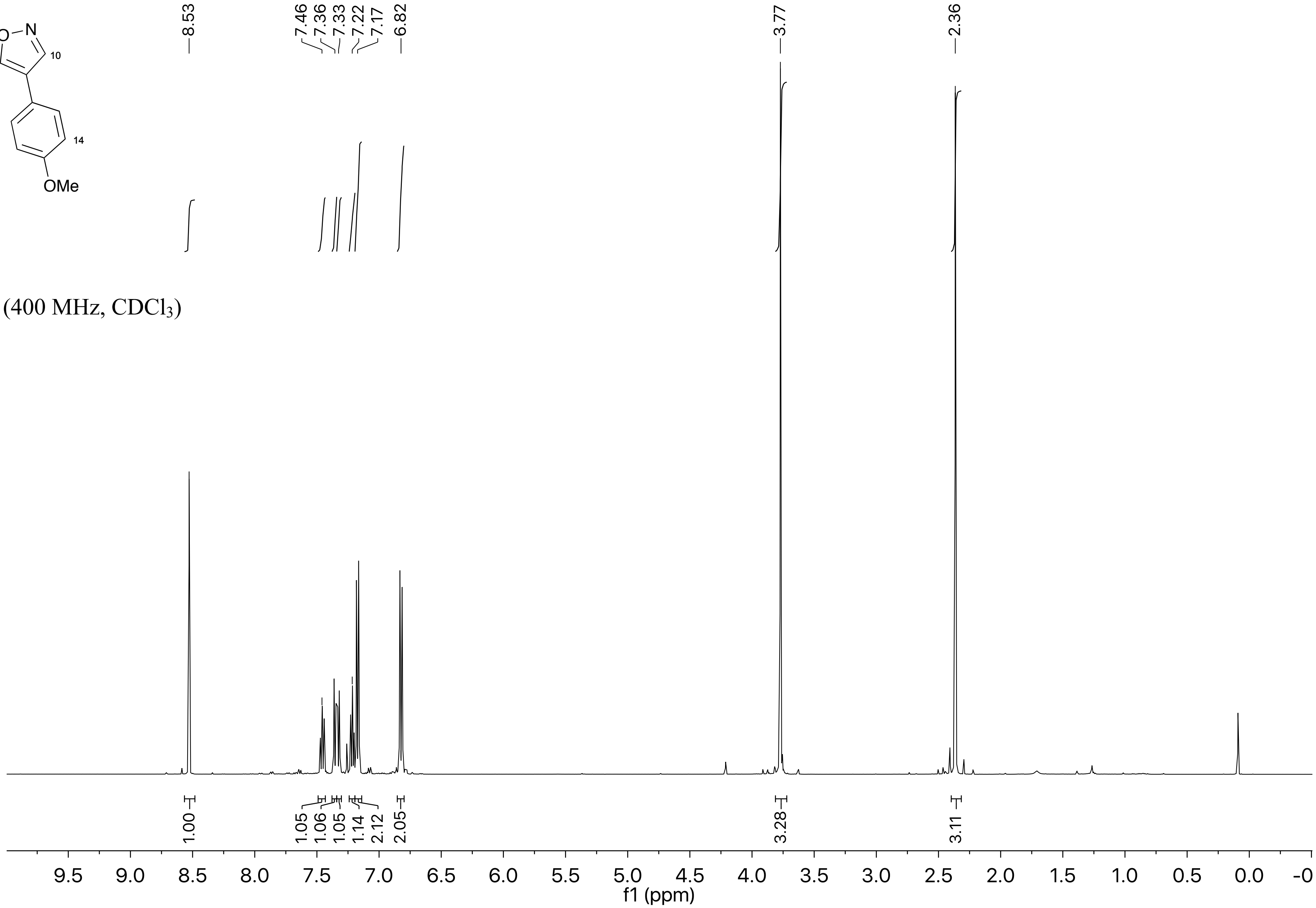

4-(4-Methoxyphenyl)-5-(2-(methylthio)phenyl)isoxazole, 6h

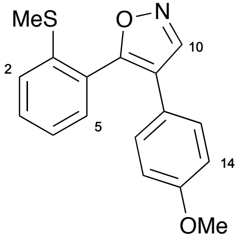

^{13}C NMR (101 MHz, $CDCl_3$)

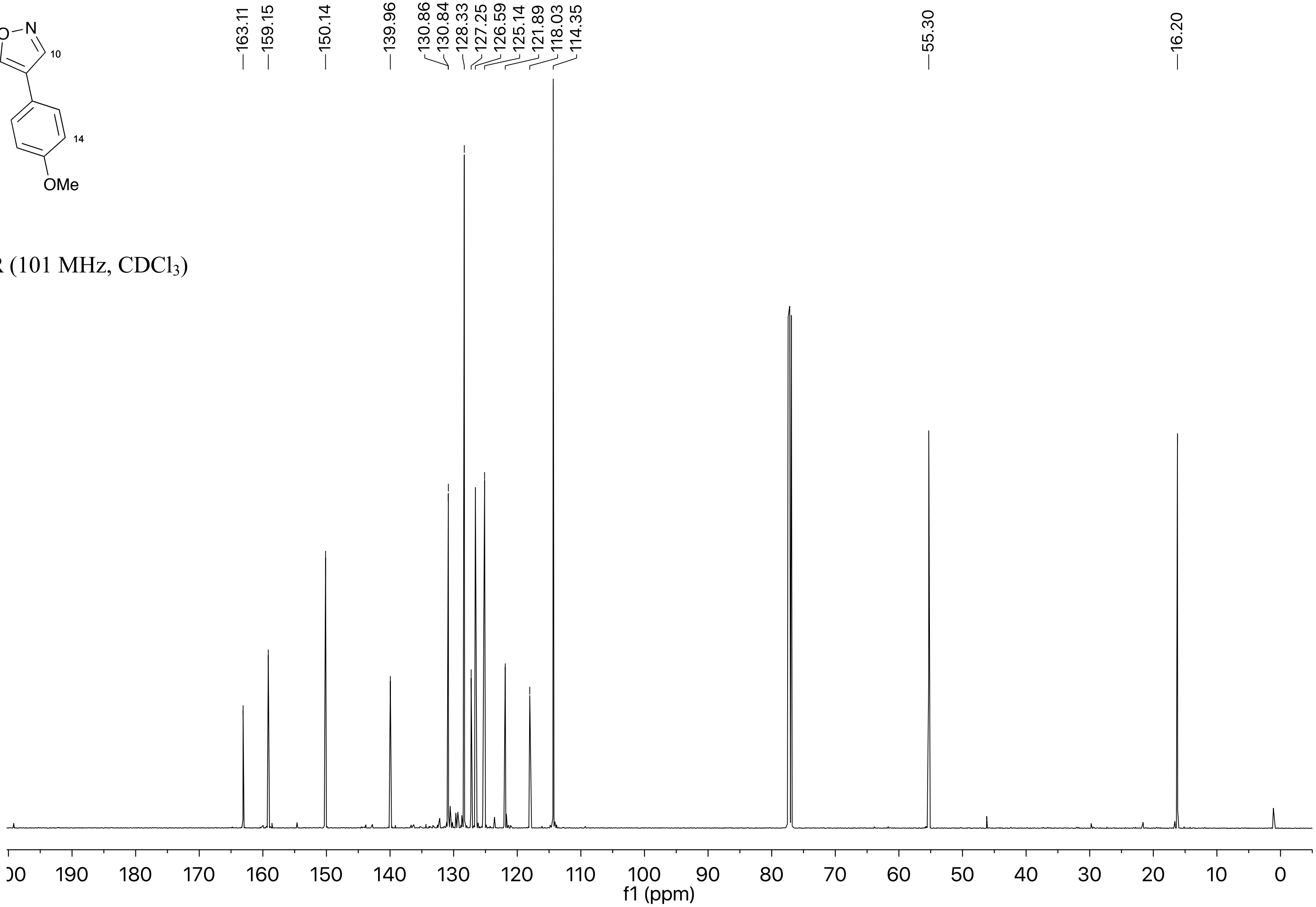

5-(2-(Methylthio)phenyl)-4-(4-(trifluoromethyl)phenyl)isoxazole, 6i

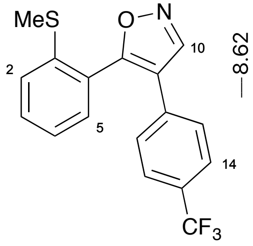

^{1}H NMR (500 MHz, $CDCl_3$)

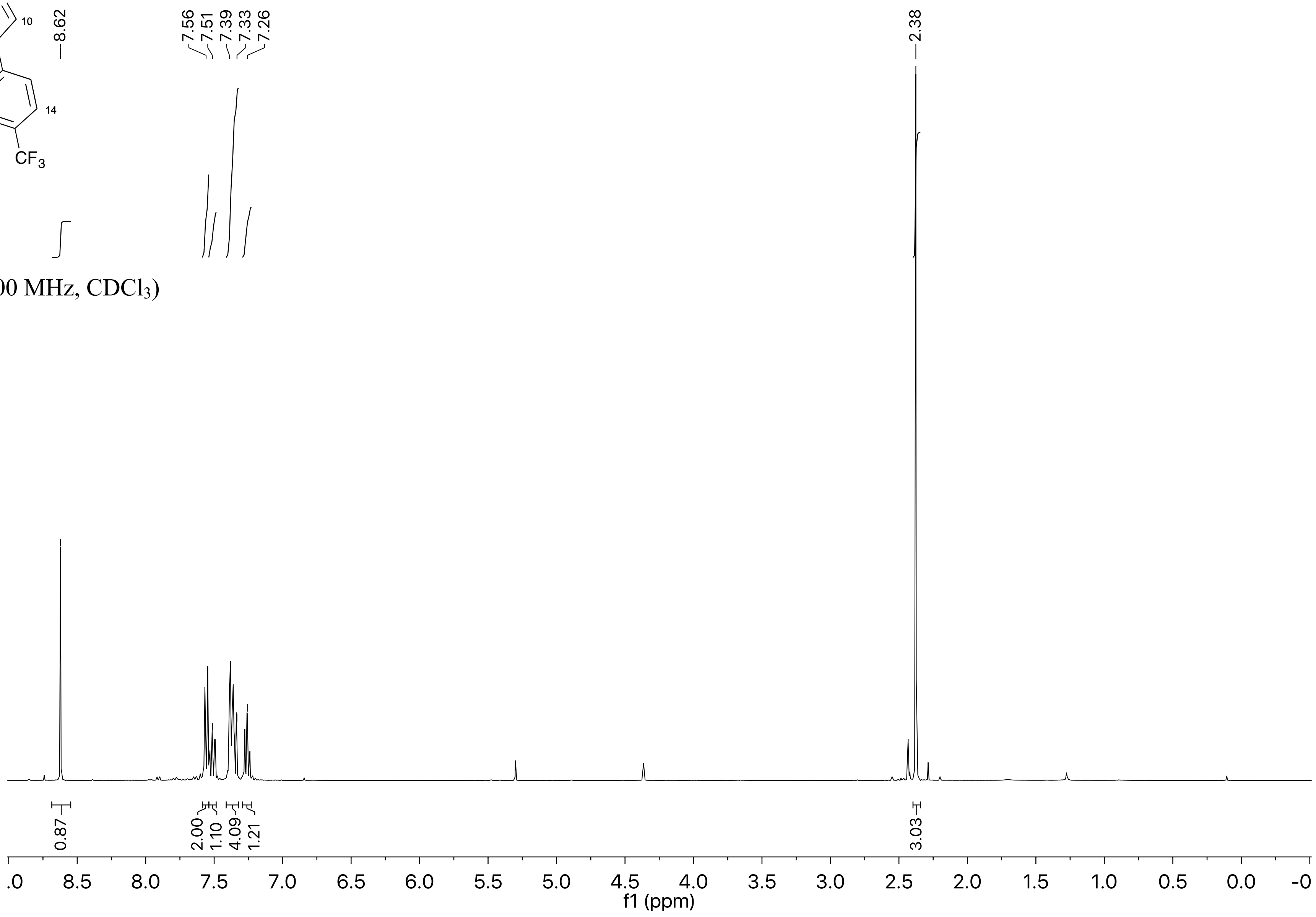

5-(2-(Methylthio)phenyl)-4-(4-(trifluoromethyl)phenyl)isoxazole, 6i

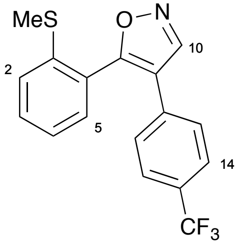

^{13}C NMR (126 MHz, $CDCl_3$)

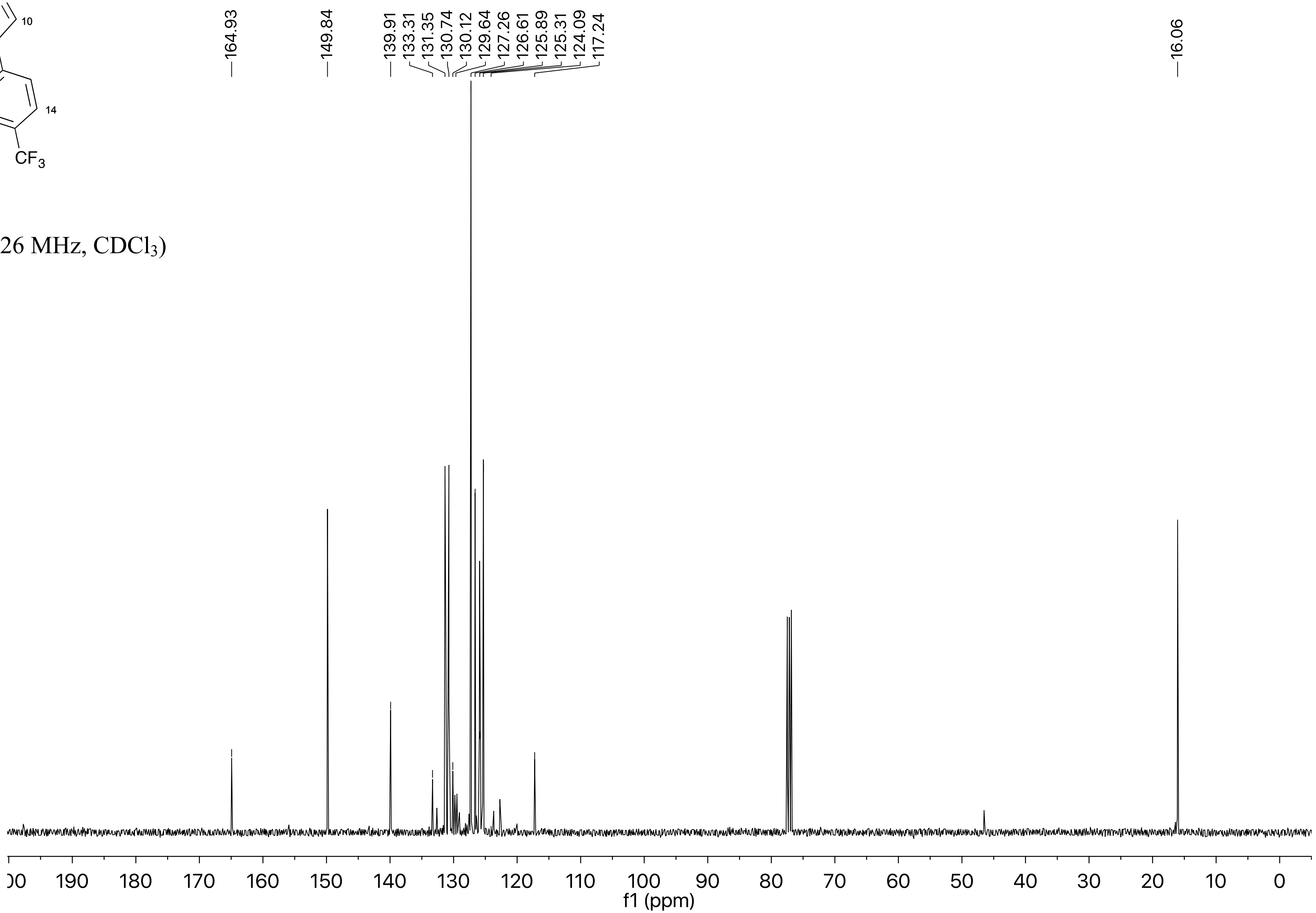

4-(3,5-Dimethoxyphenyl)-5-(2-(methylthio)phenyl)isoxazole, 6j

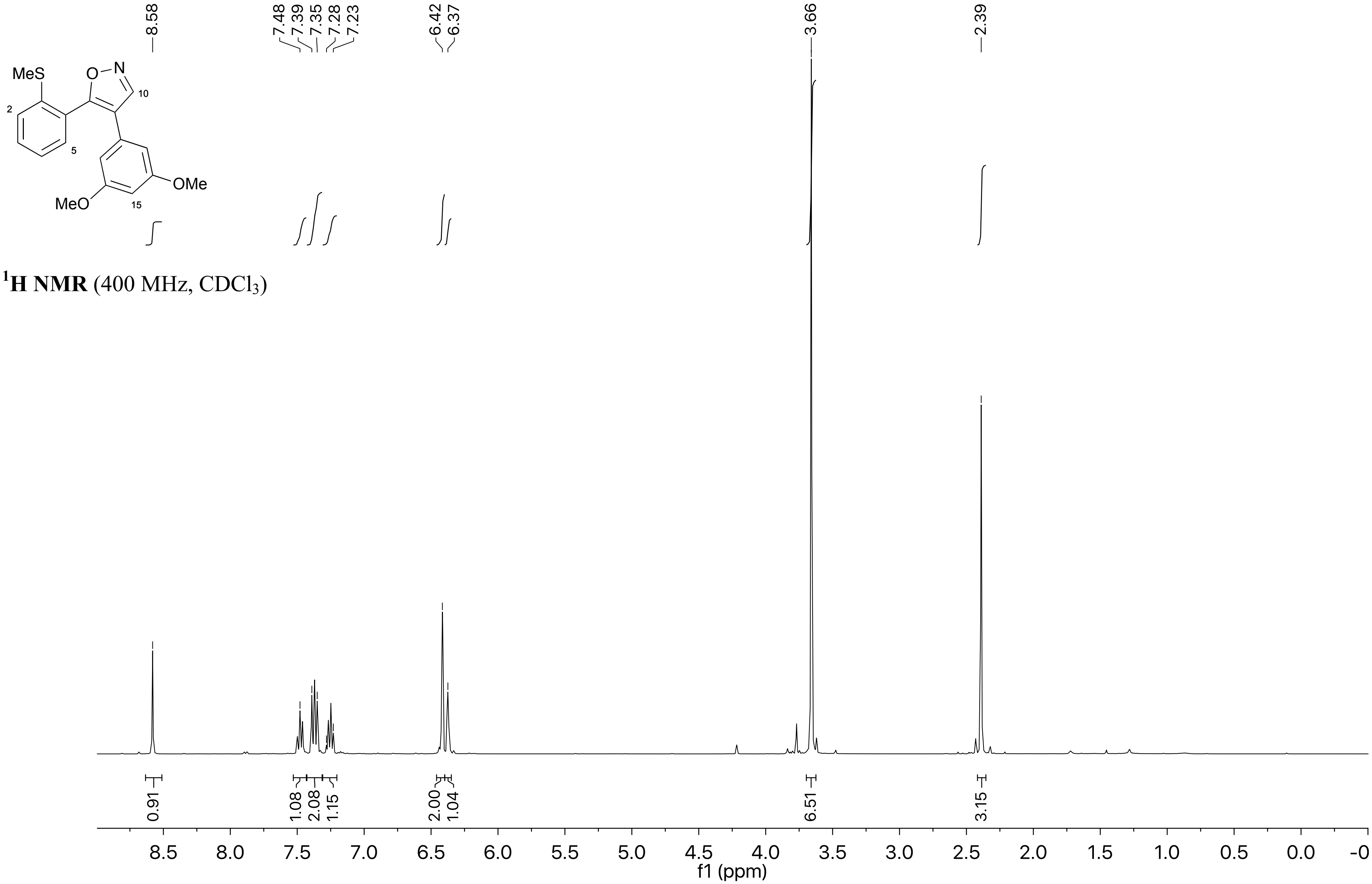

^{1}H NMR (400 MHz, $CDCl_3$)

4-(3,5-Dimethoxyphenyl)-5-(2-(methylthio)phenyl)isoxazole, 6j

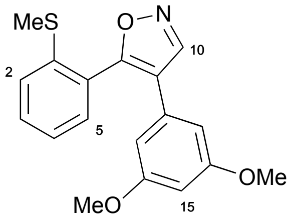

^{13}C NMR (101 MHz, $CDCl_3$)

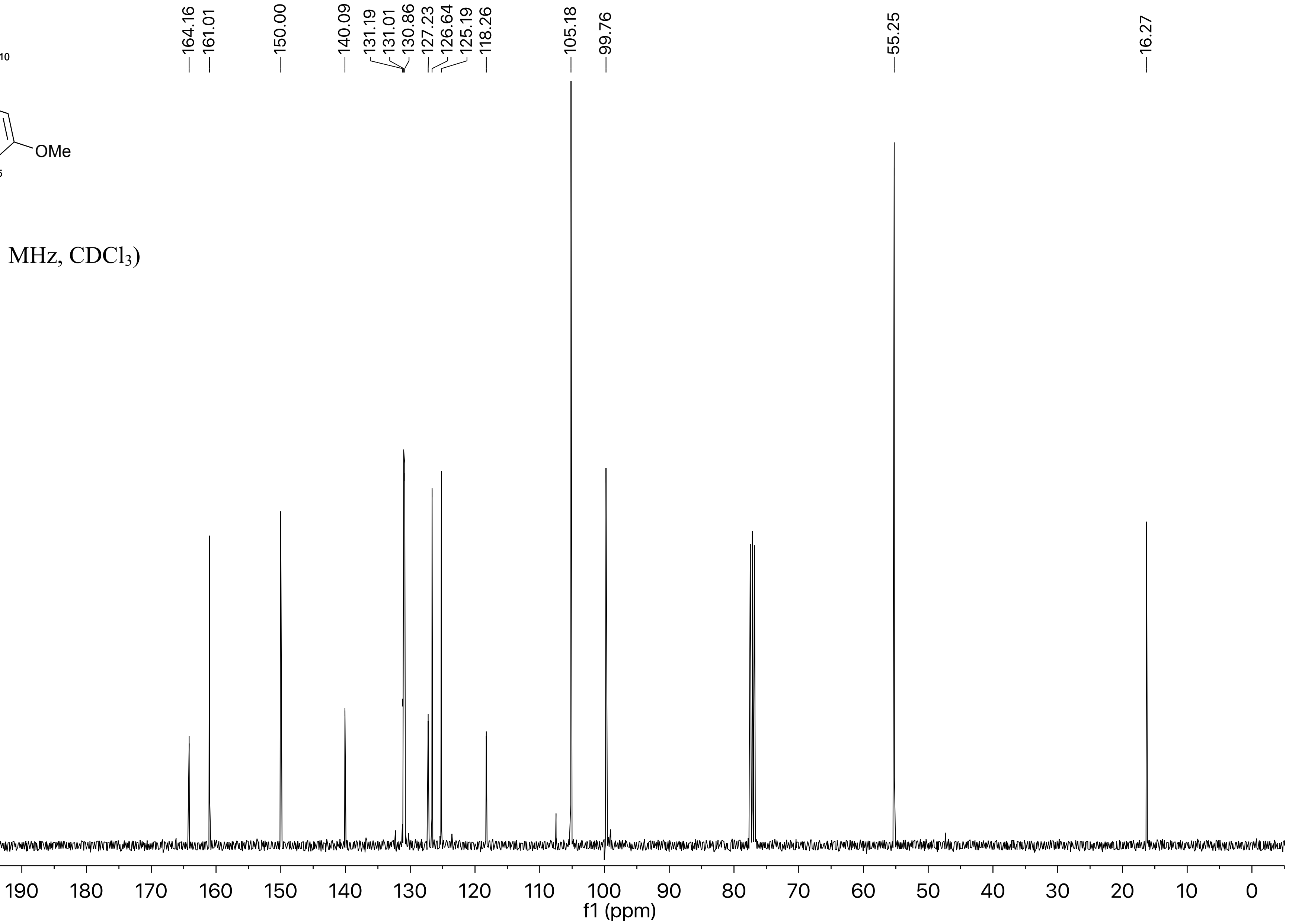

5-(2-(Methylthio)phenyl)-4-(thiophen-3-yl)isoxazole, 6k

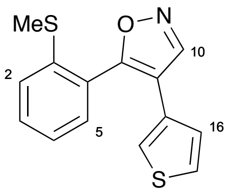

^{1}H NMR (400 MHz, $CDCl_3$)

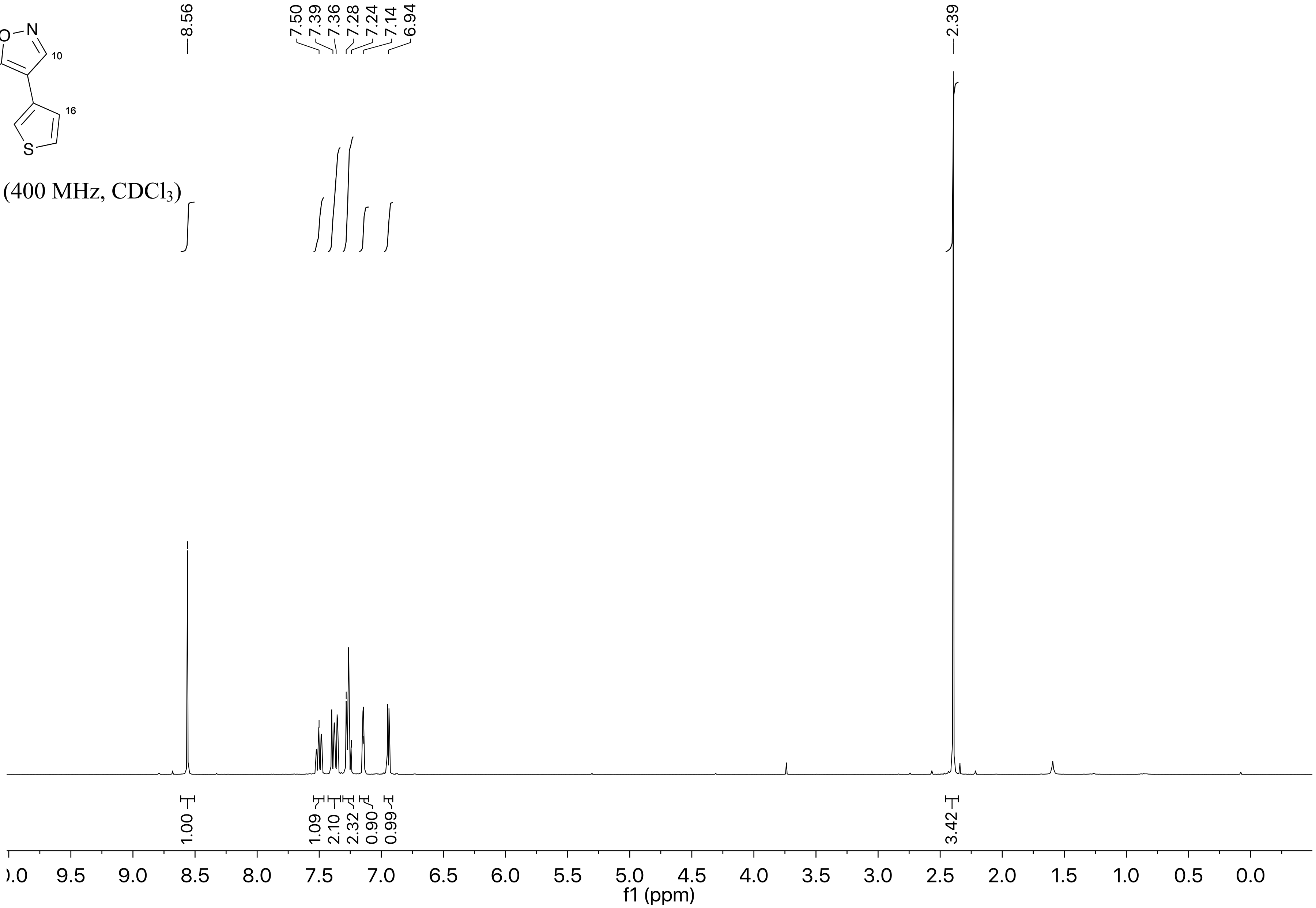

5-(2-(Methylthio)phenyl)-4-(thiophen-3-yl)isoxazole, 6k

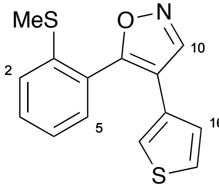

^{13}C NMR (101 MHz, $CDCl_3$)

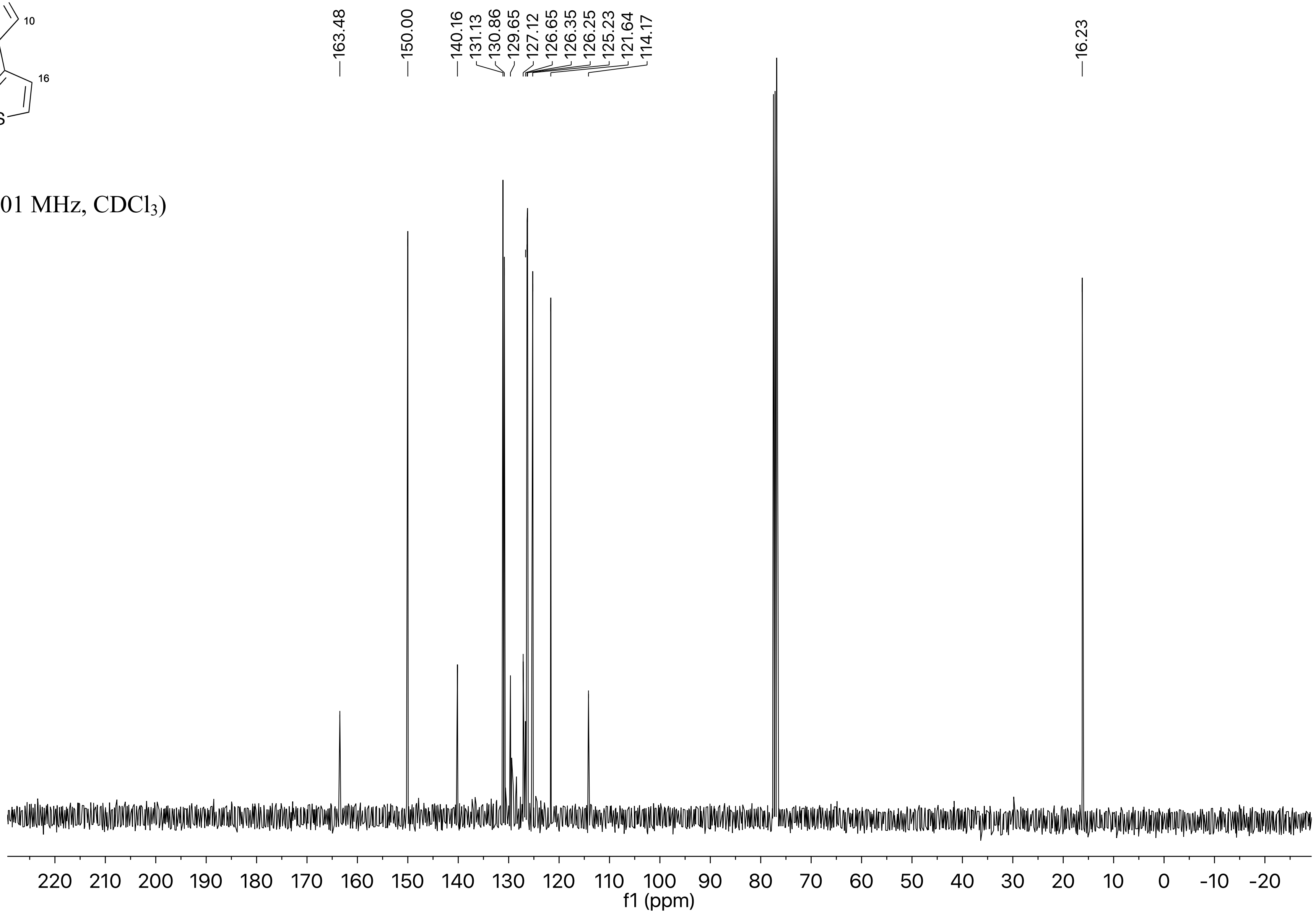

4-(Cyclohex-1-en-1-yl)-5-(2-(methylthio)phenyl)isoxazole, 6l

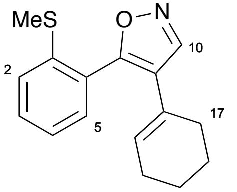

^{1}H NMR (500 MHz, $CDCl_3$)

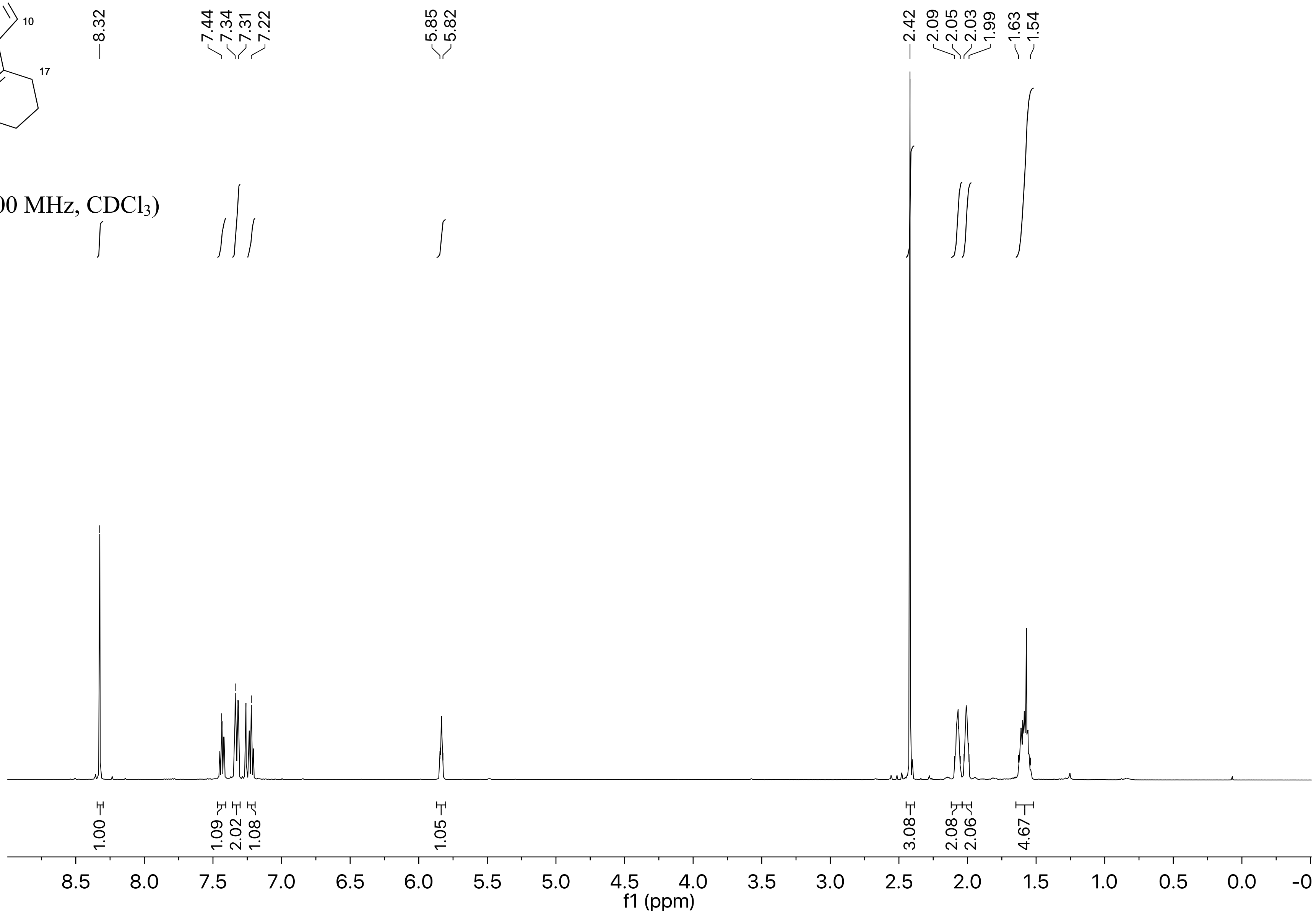

4-(Cyclohex-1-en-1-yl)-5-(2-(methylthio)phenyl)isoxazole, 6l

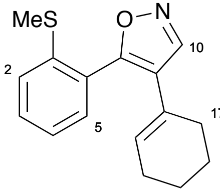

^{13}C NMR (126 MHz, $CDCl_3$)

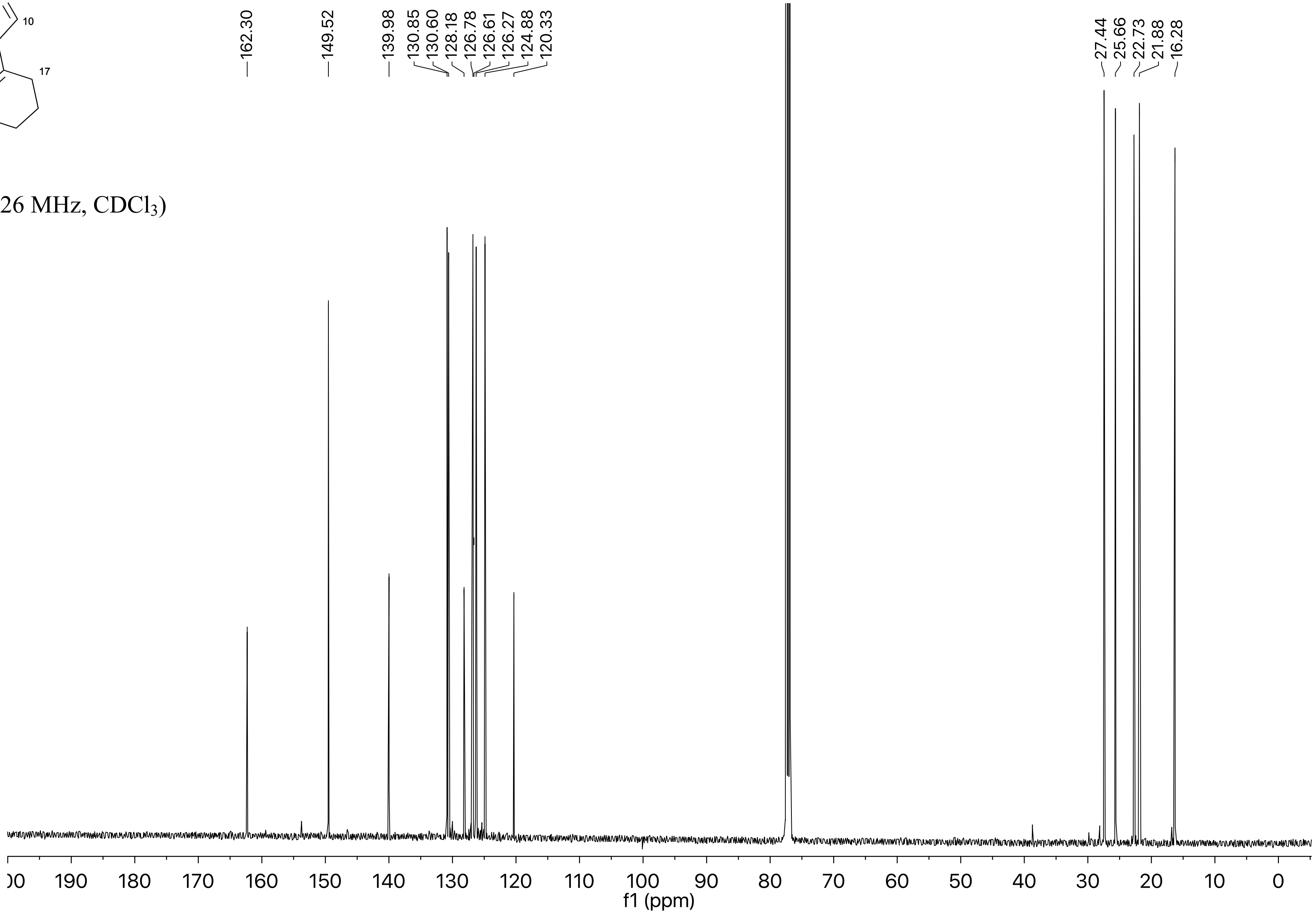

4-Hexyl-5-(4-methoxy-2-(methylthio)phenyl)isoxazole, 6n

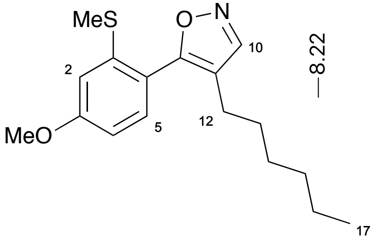

^{1}H NMR (400 MHz, $CDCl_3$)

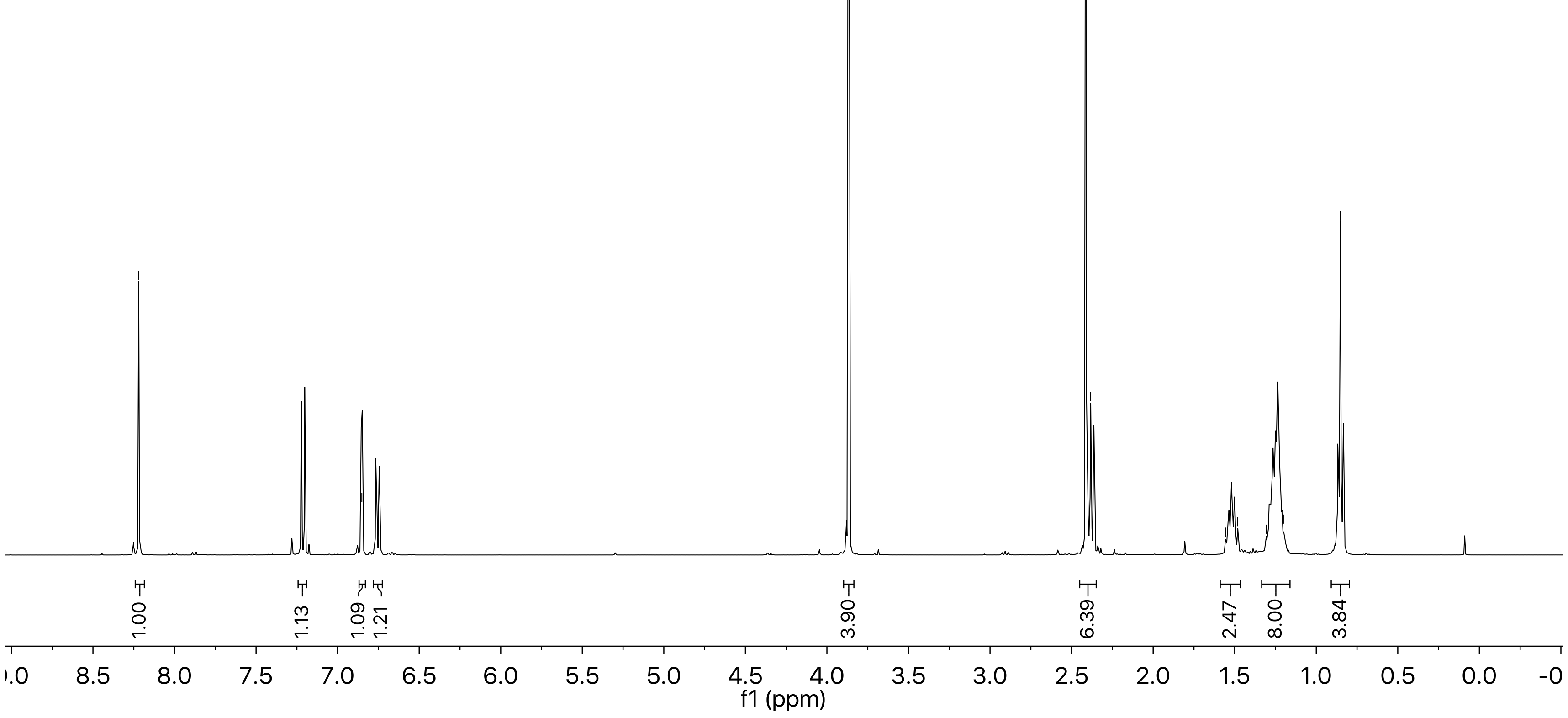

4-Hexyl-5-(4-methoxy-2-(methylthio)phenyl)isoxazole, 6n

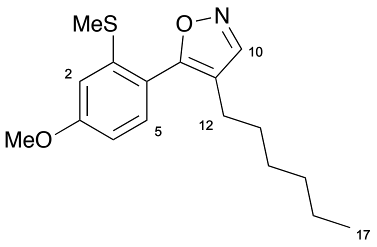

^{13}C NMR (101 MHz, $CDCl_3$)

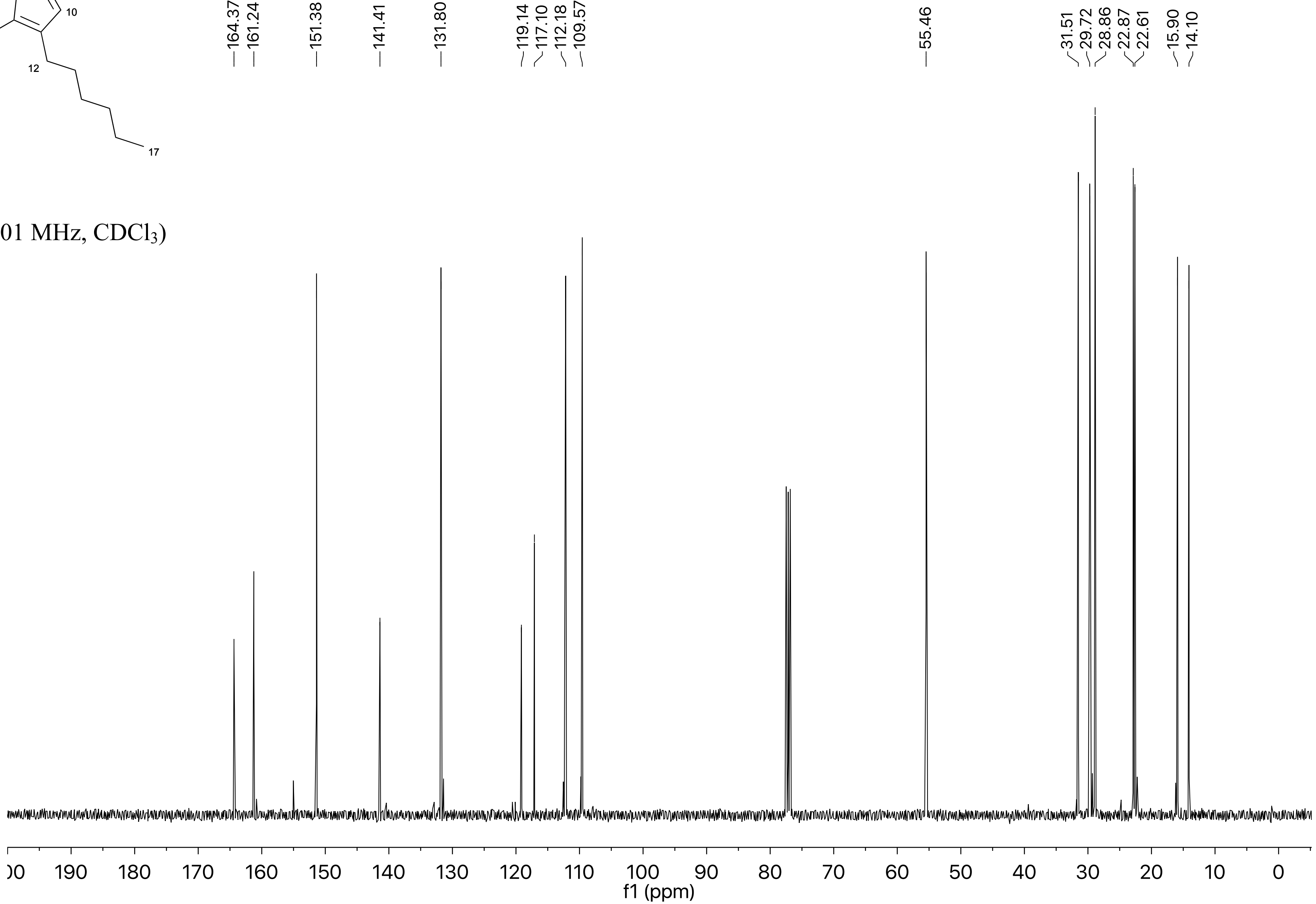

4-Hexyl-5-(2-(methylthio)-4-(trifluoromethyl)phenyl)isoxazole, 6o

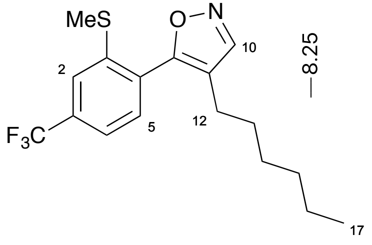

^{1}H NMR (500 MHz, $CDCl_3$)

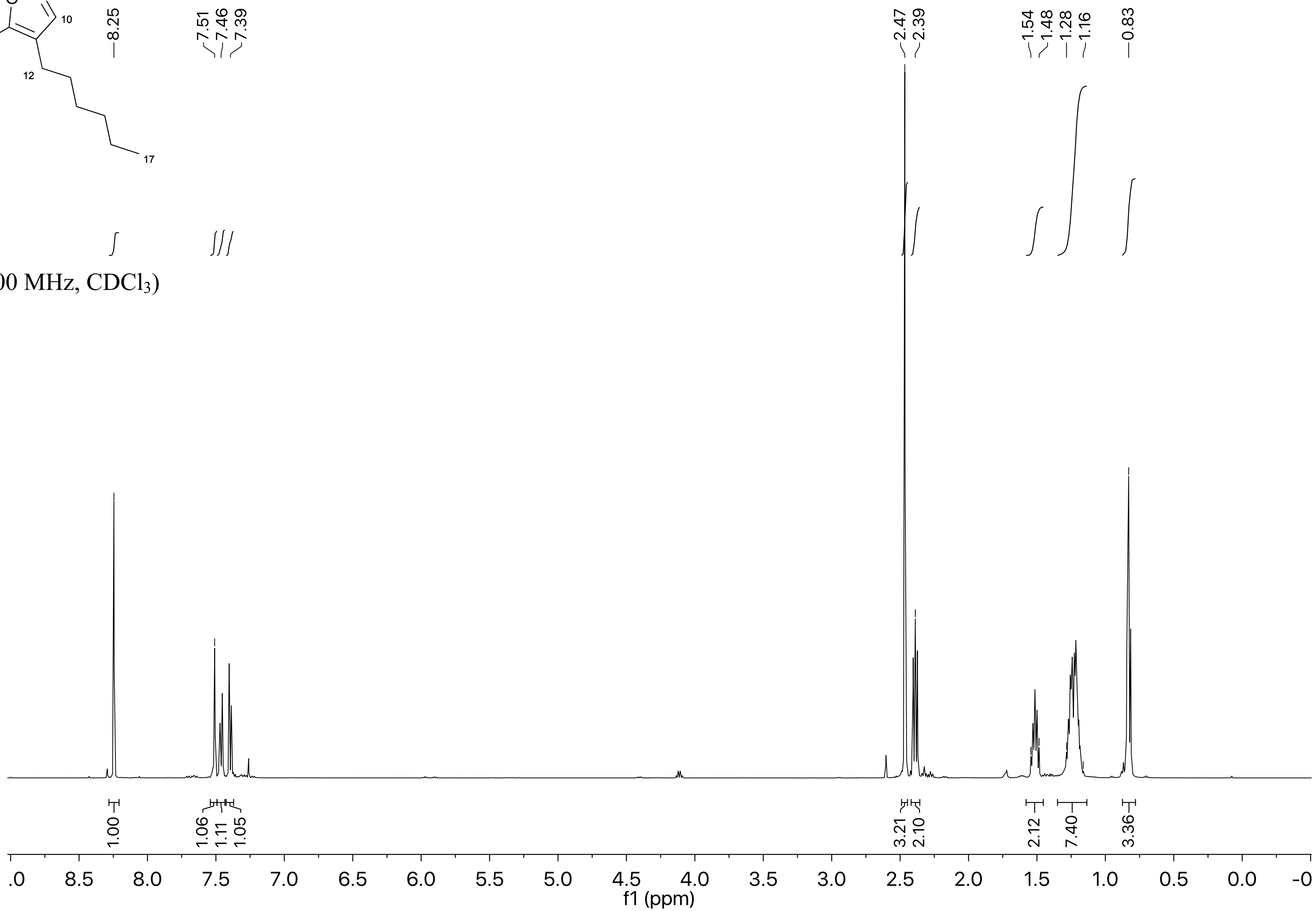

4-Hexyl-5-(2-(methylthio)-4-(trifluoromethyl)phenyl)isoxazole, 6o

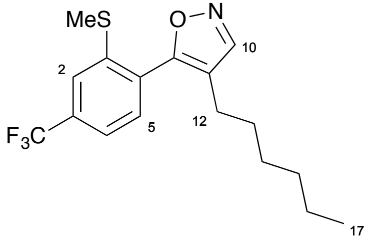

^{13}C NMR (126 MHz, $CDCl_3$)

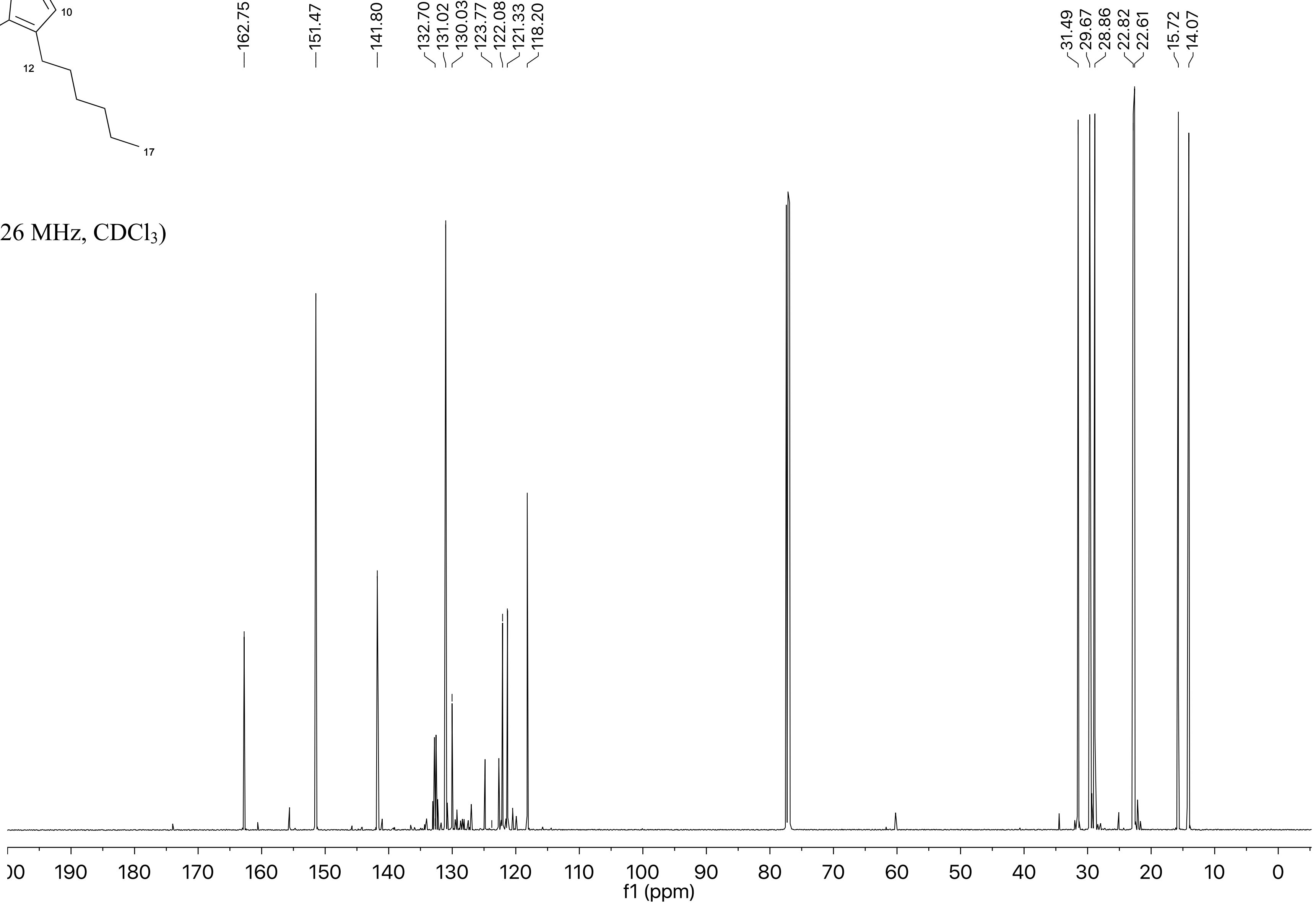

5-(4-Bromo-2-(methylthio)phenyl)-4-hexylisoxazole, 6p

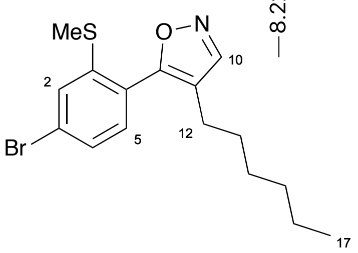

^{1}H NMR (400 MHz, $CDCl_3$)

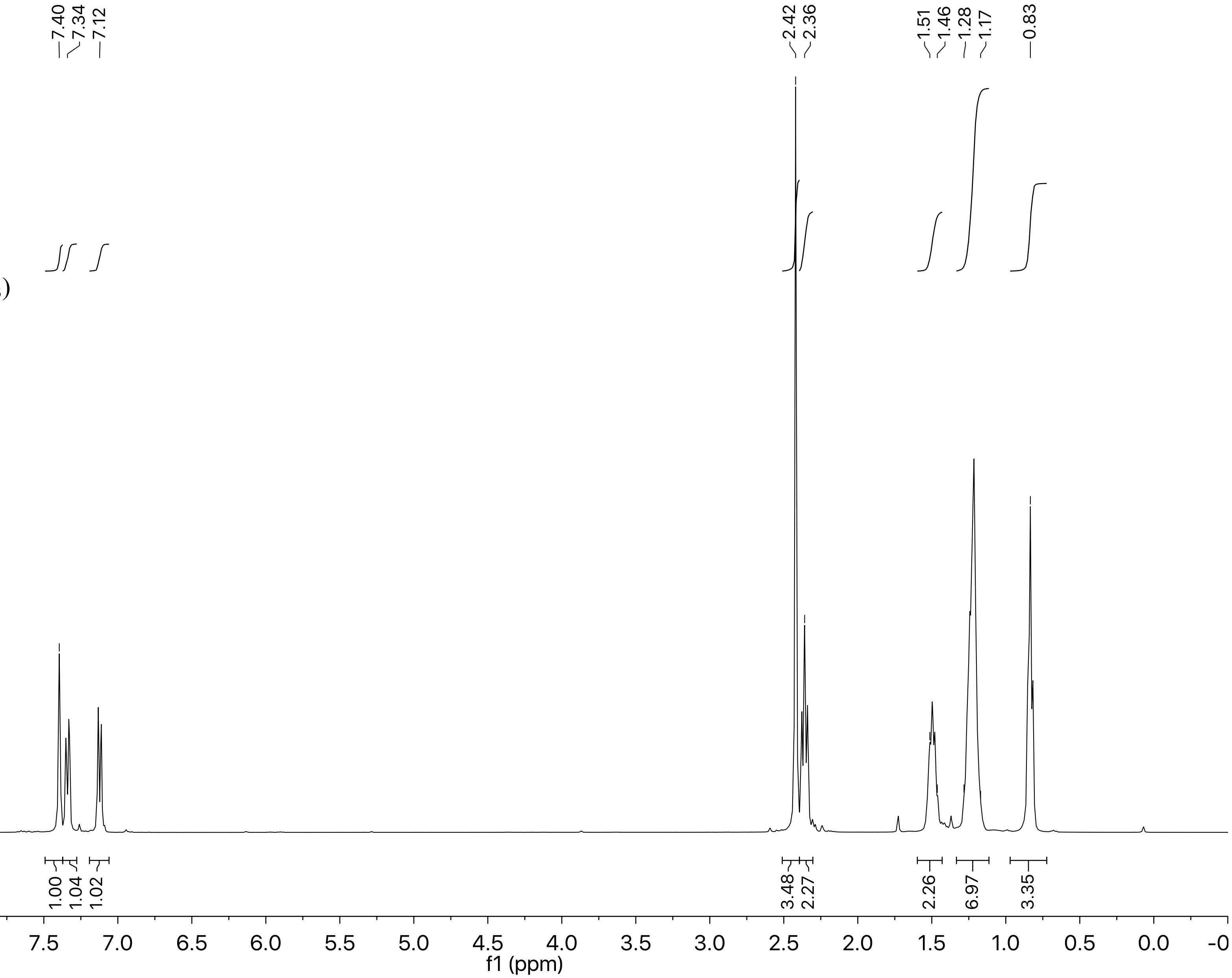

5-(4-Bromo-2-(methylthio)phenyl)-4-hexylisoxazole, 6p

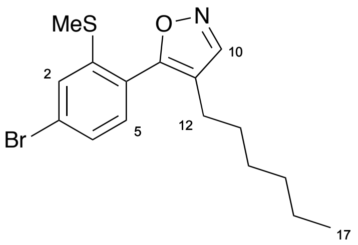

^{13}C NMR (101 MHz, $CDCl_3$)

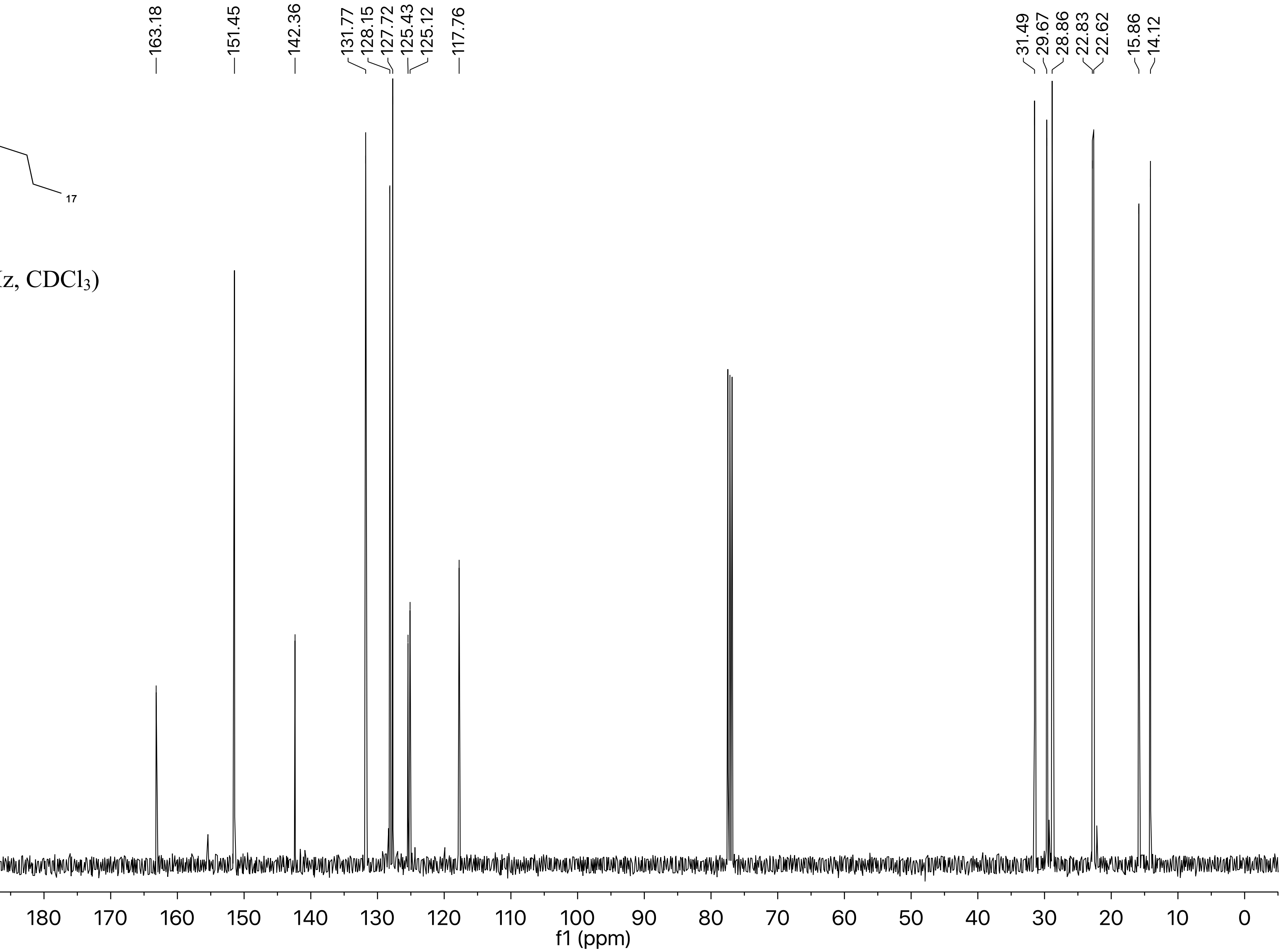

4-Hexyl-5-(3-(methylthio)thiophen-2-yl)isoxazole, 6q

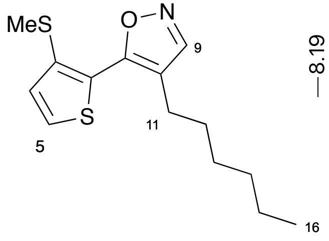

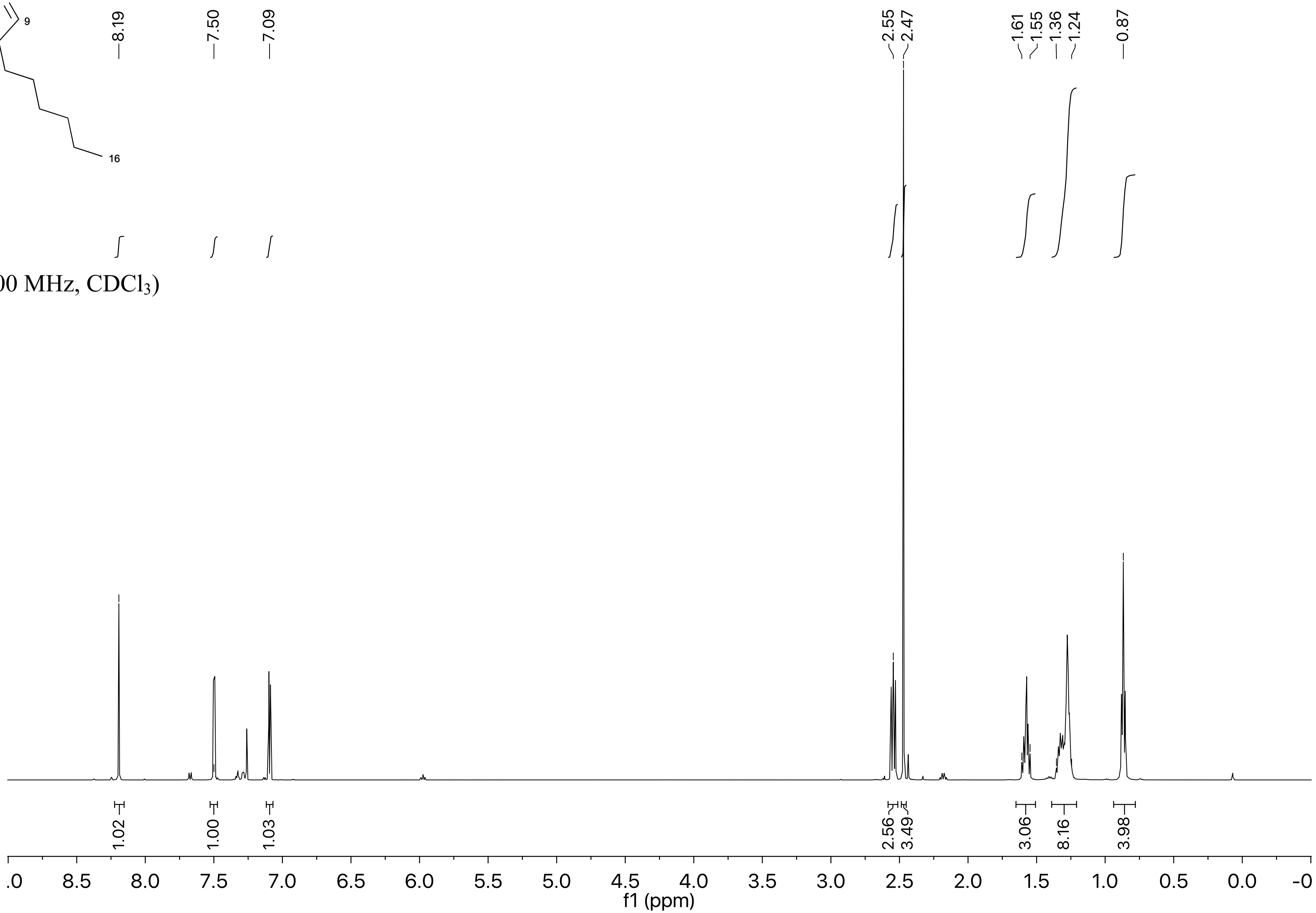

^{1}H NMR (500 MHz, $CDCl_3$)

4-Hexyl-5-(3-(methylthio)thiophen-2-yl)isoxazole, 6q

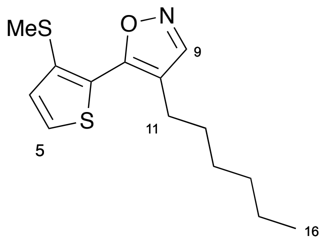

^{13}C NMR (126 MHz, $CDCl_3$)

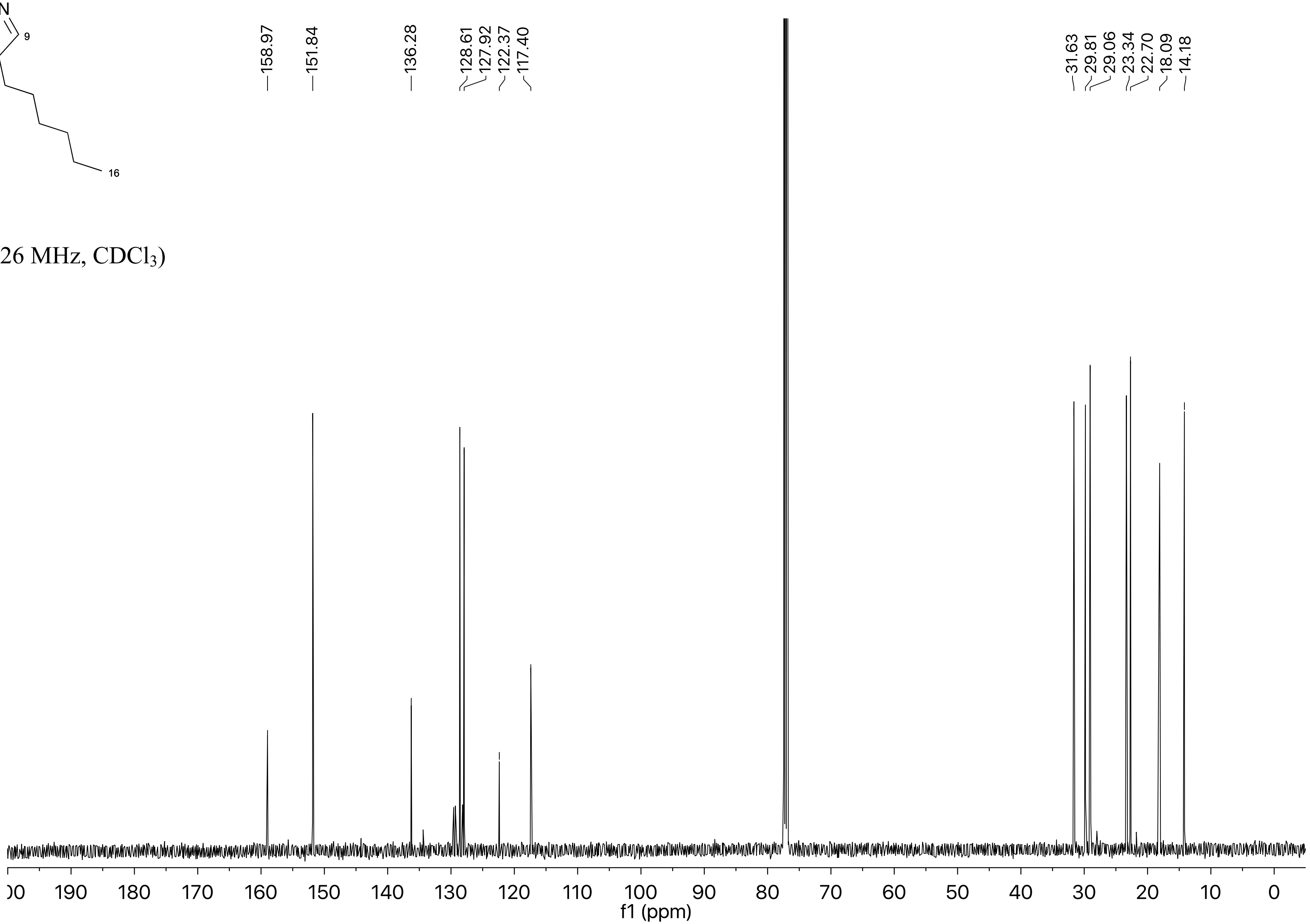

5-(1-(Ethylthio)octan-2-yl)-4-hexylisoxazole, 6t

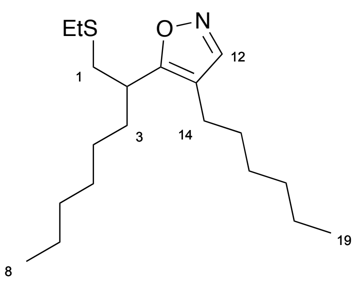

^{1}H NMR (400 MHz, $CDCl_3$)

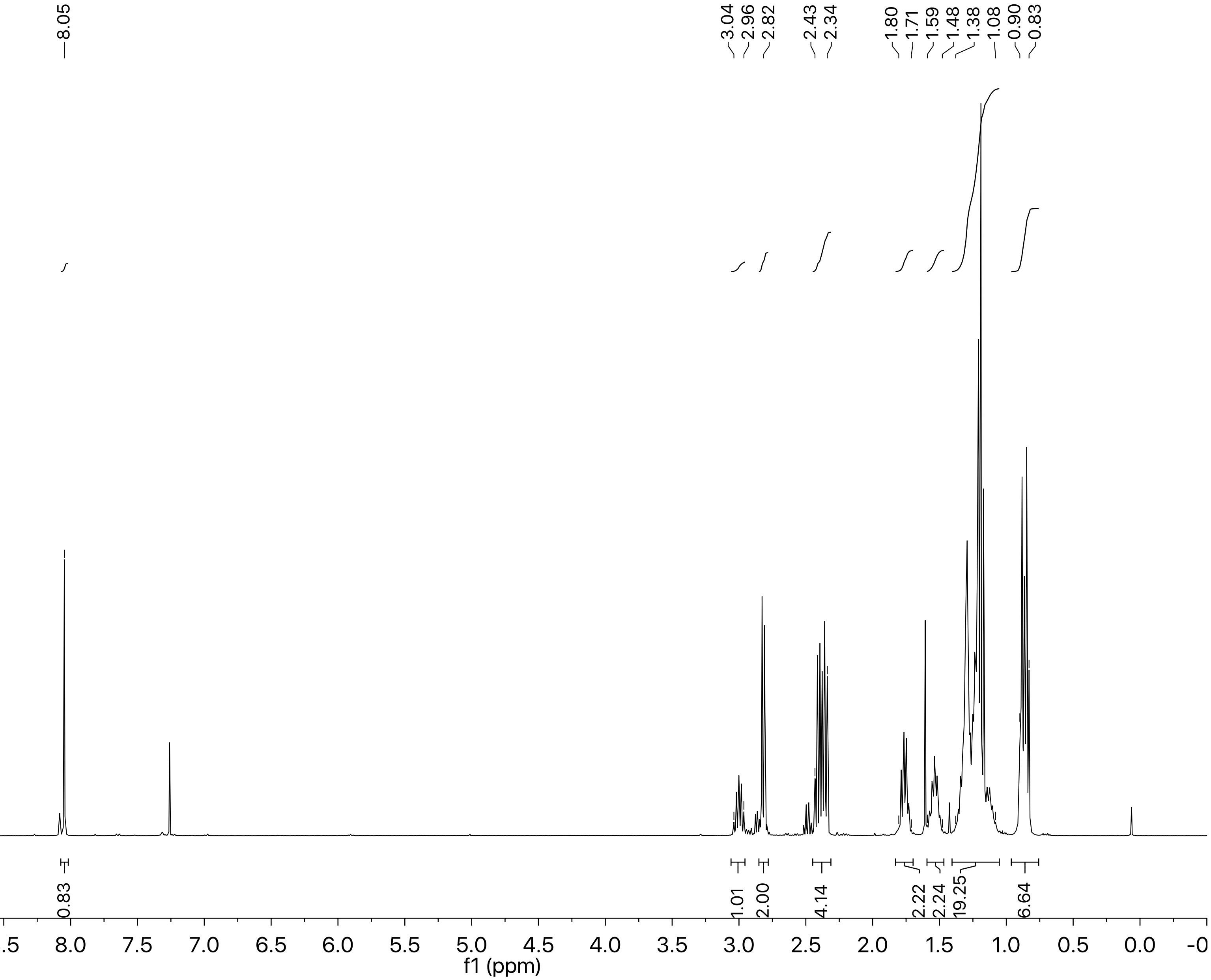

5-(1-(Ethylthio)octan-2-yl)-4-hexylisoxazole, 6t

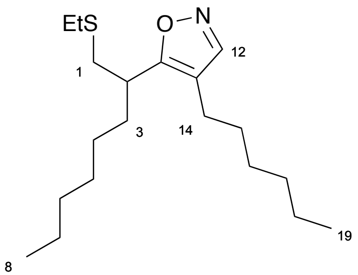

^{13}C NMR (101 MHz, $CDCl_3$)

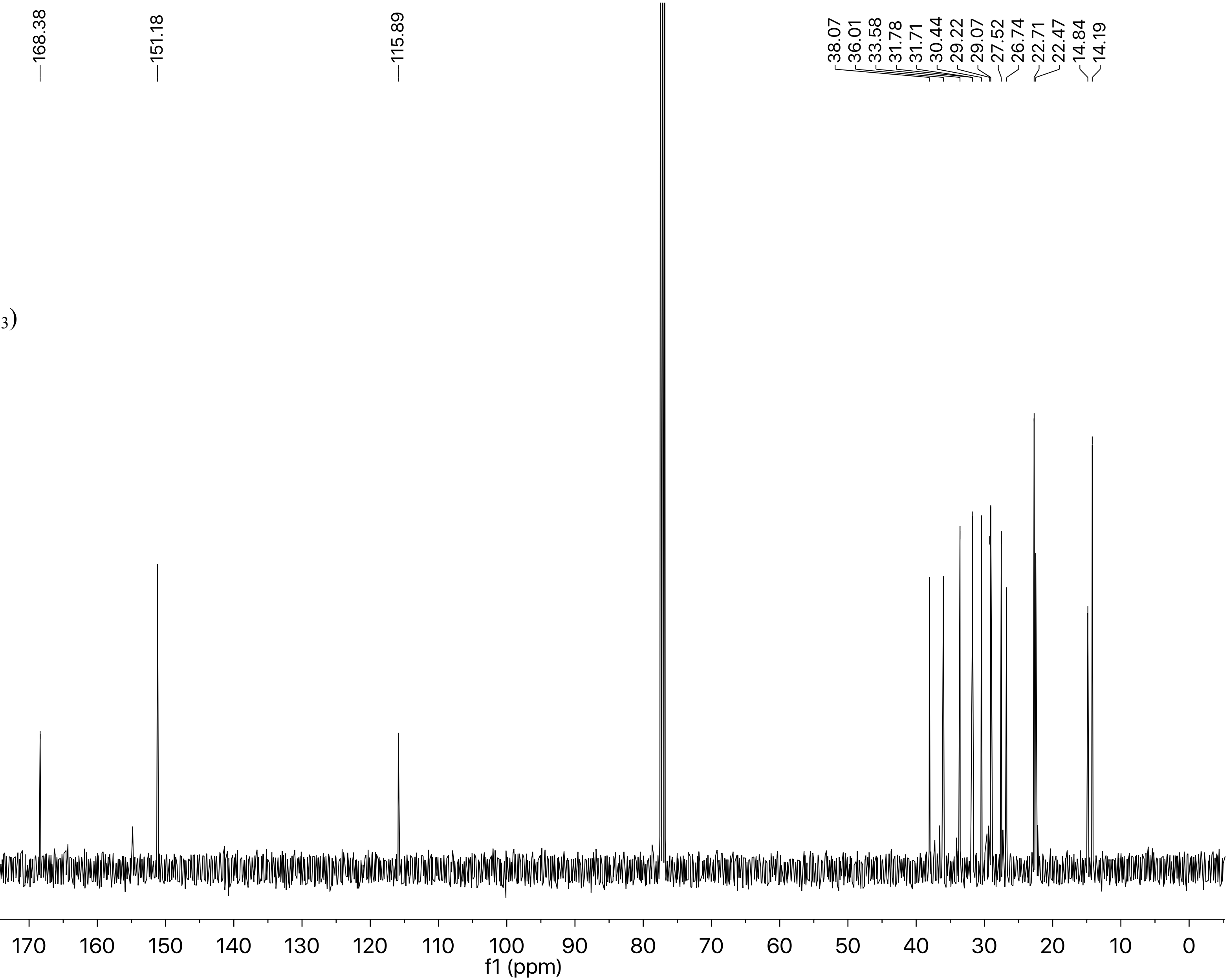

5-(1-(Ethylthio)-3-phenylpropan-2-yl)-4-hexylisoxazole, 6u

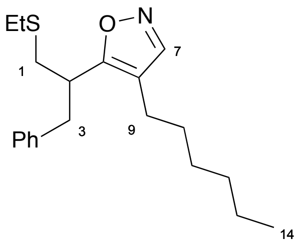

^{1}H NMR (400 MHz, $CDCl_3$)

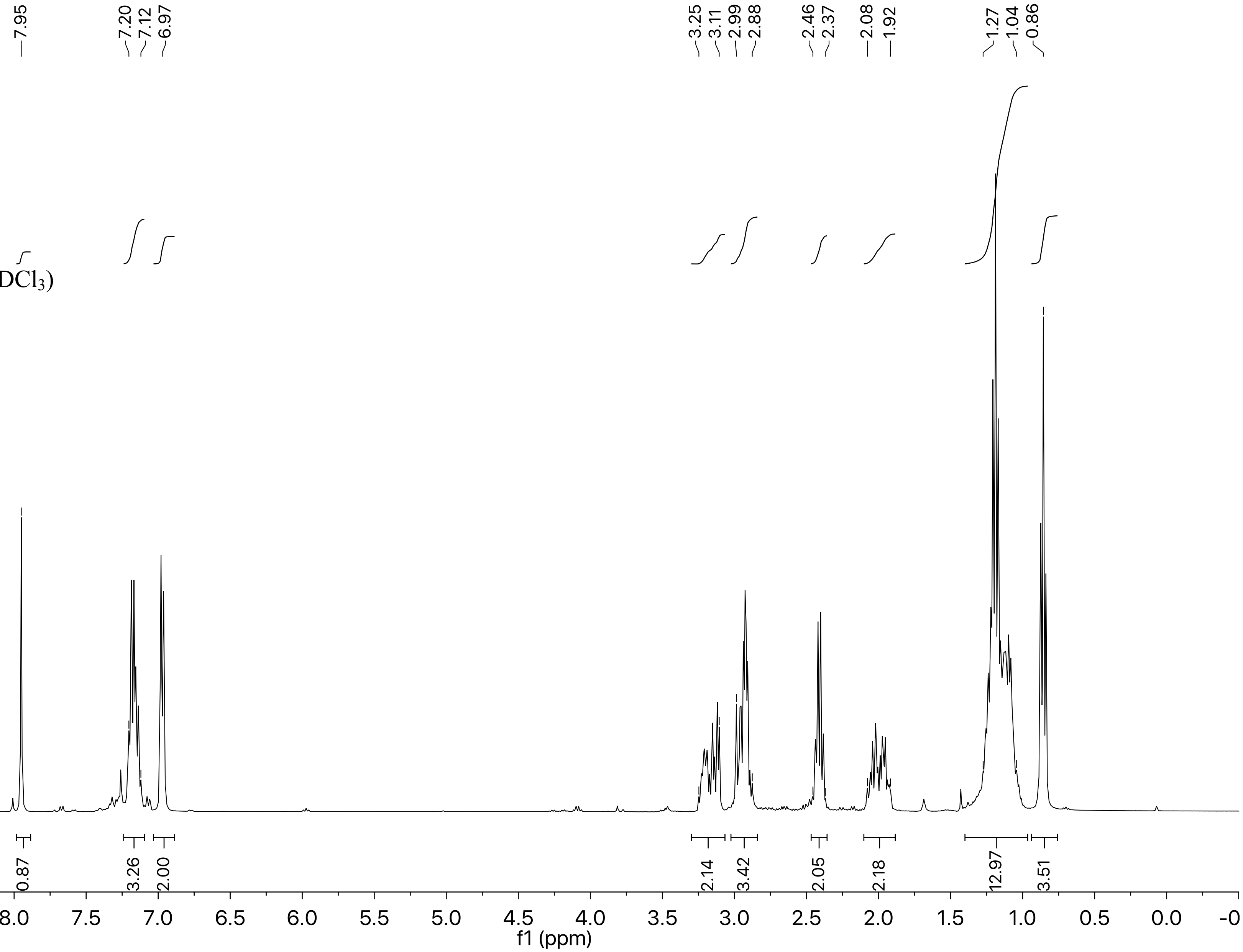

5-(1-(Ethylthio)-3-phenylpropan-2-yl)-4-hexylisoxazole, 6u

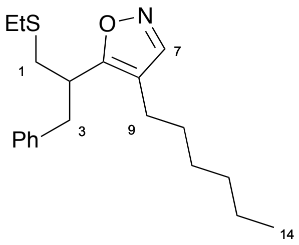

^{13}C NMR (101 MHz, $CDCl_3$)

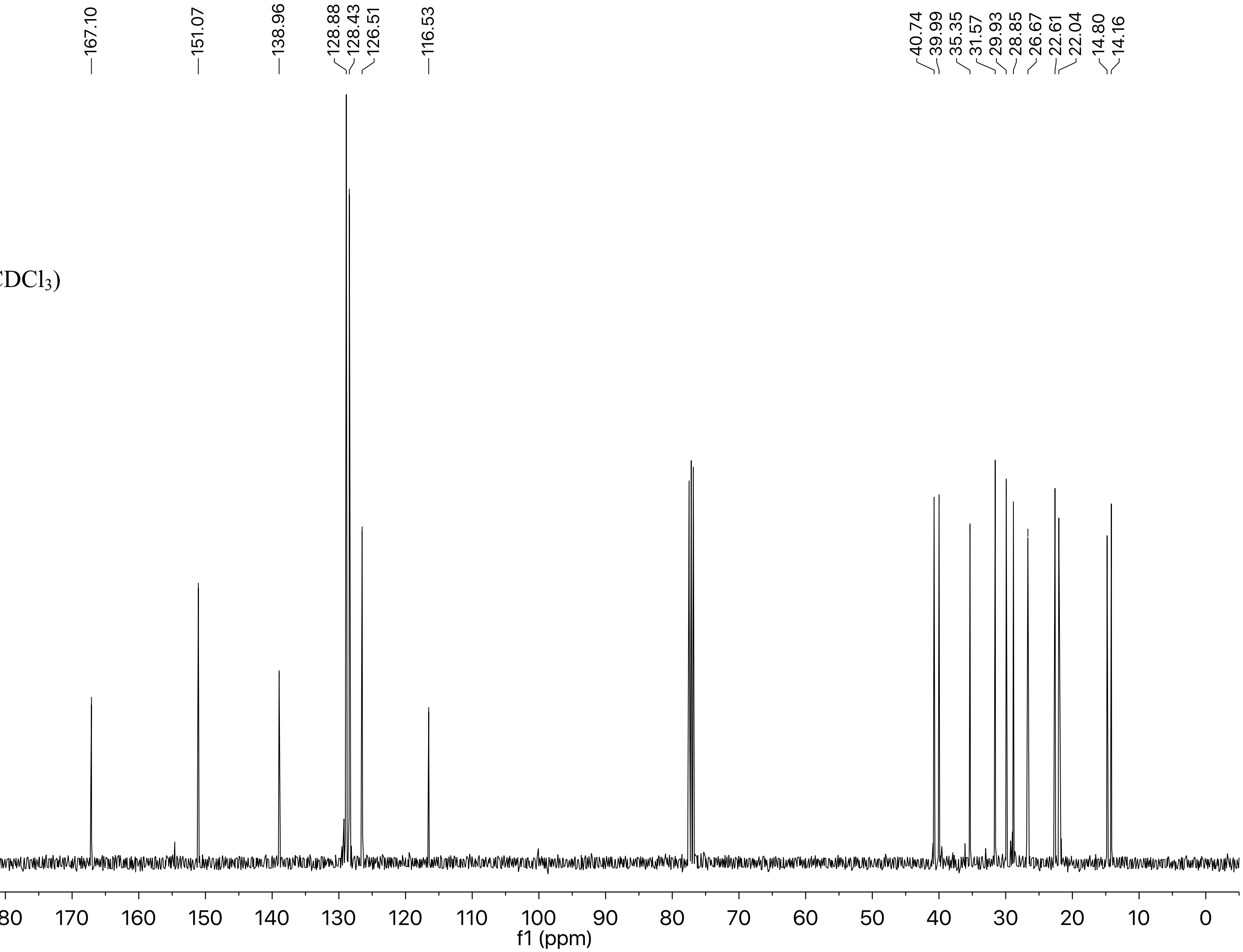

1-Cyclohexyl-4-hexyl-5-(2-(methylthio)phenyl)-1*H*-pyrazole, 7a

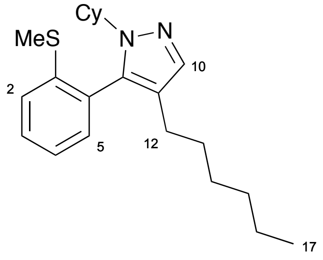

^{1}H NMR (400 MHz, $CDCl_3$)

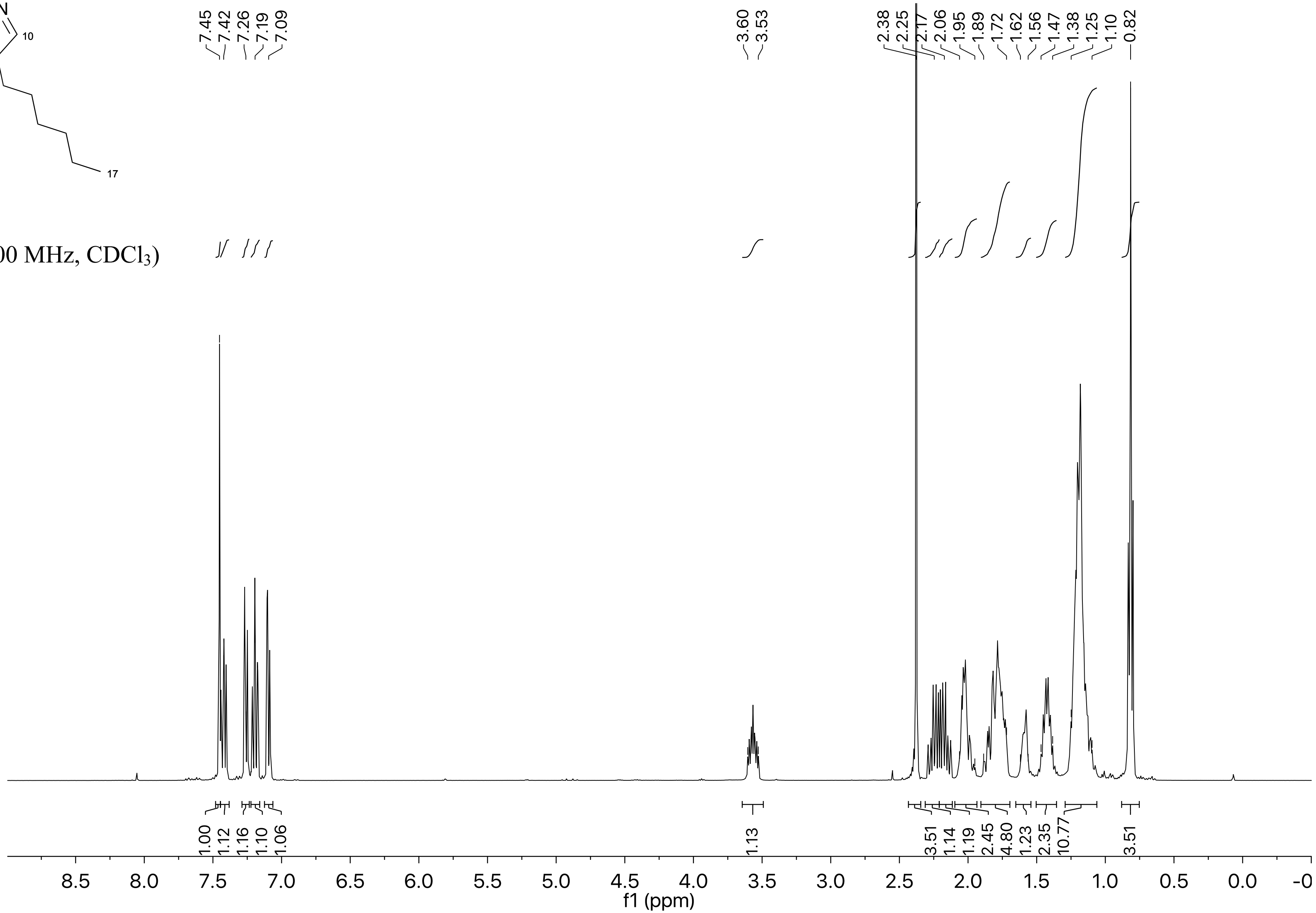

1-Cyclohexyl-4-hexyl-5-(2-(methylthio)phenyl)-1*H*-pyrazole, 7a

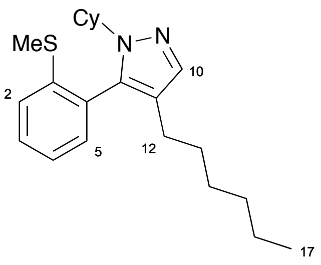

^{13}C NMR (101 MHz, $CDCl_3$)

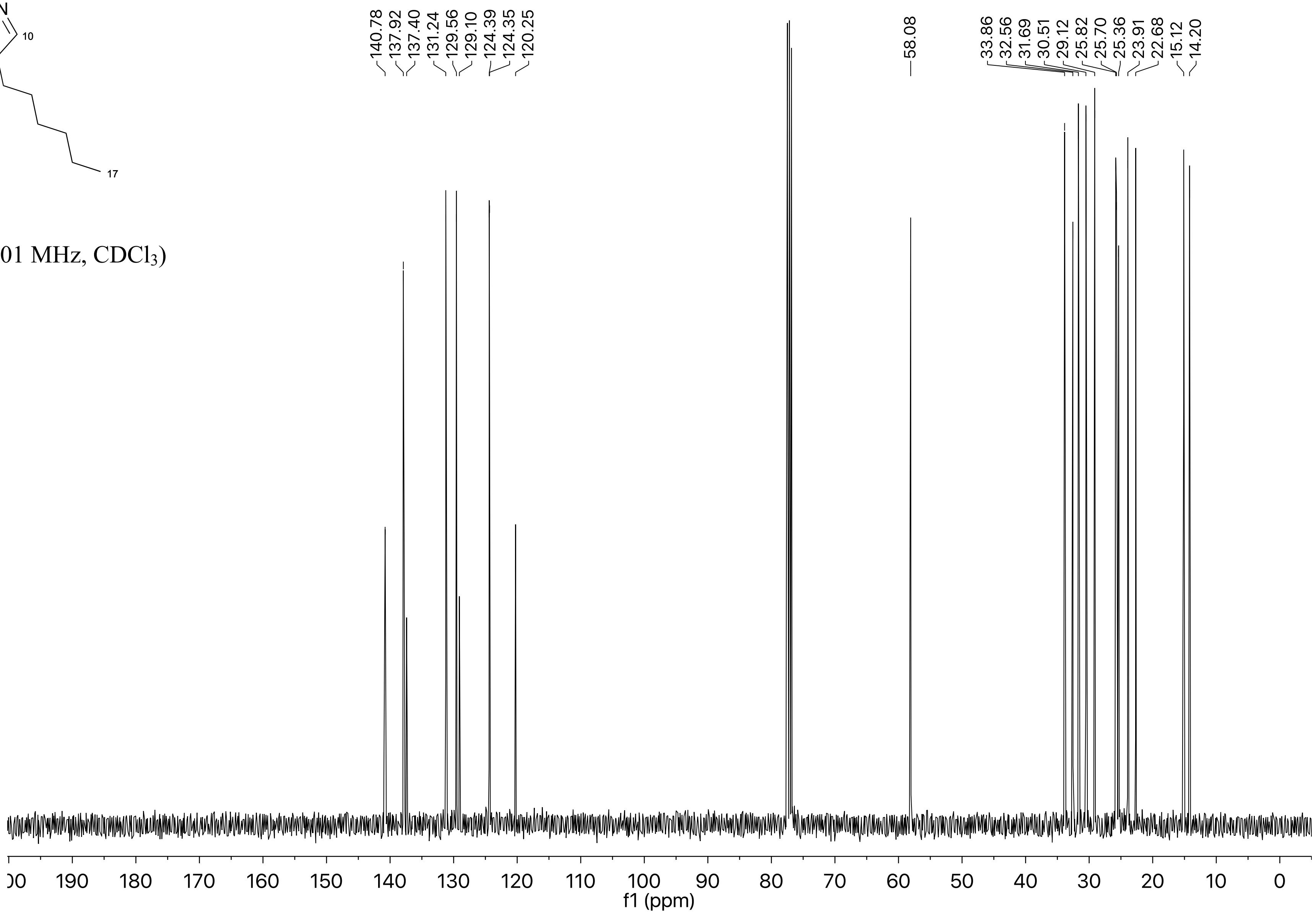

1-(*tert*-Butyl)-4-hexyl-5-(2-(methylthio)phenyl)-1*H*-pyrazole, 7b

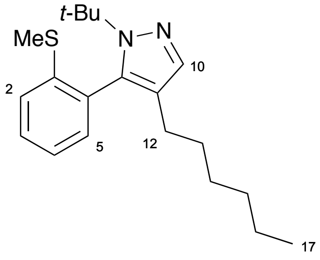

^{1}H NMR (400 MHz, $CDCl_3$)

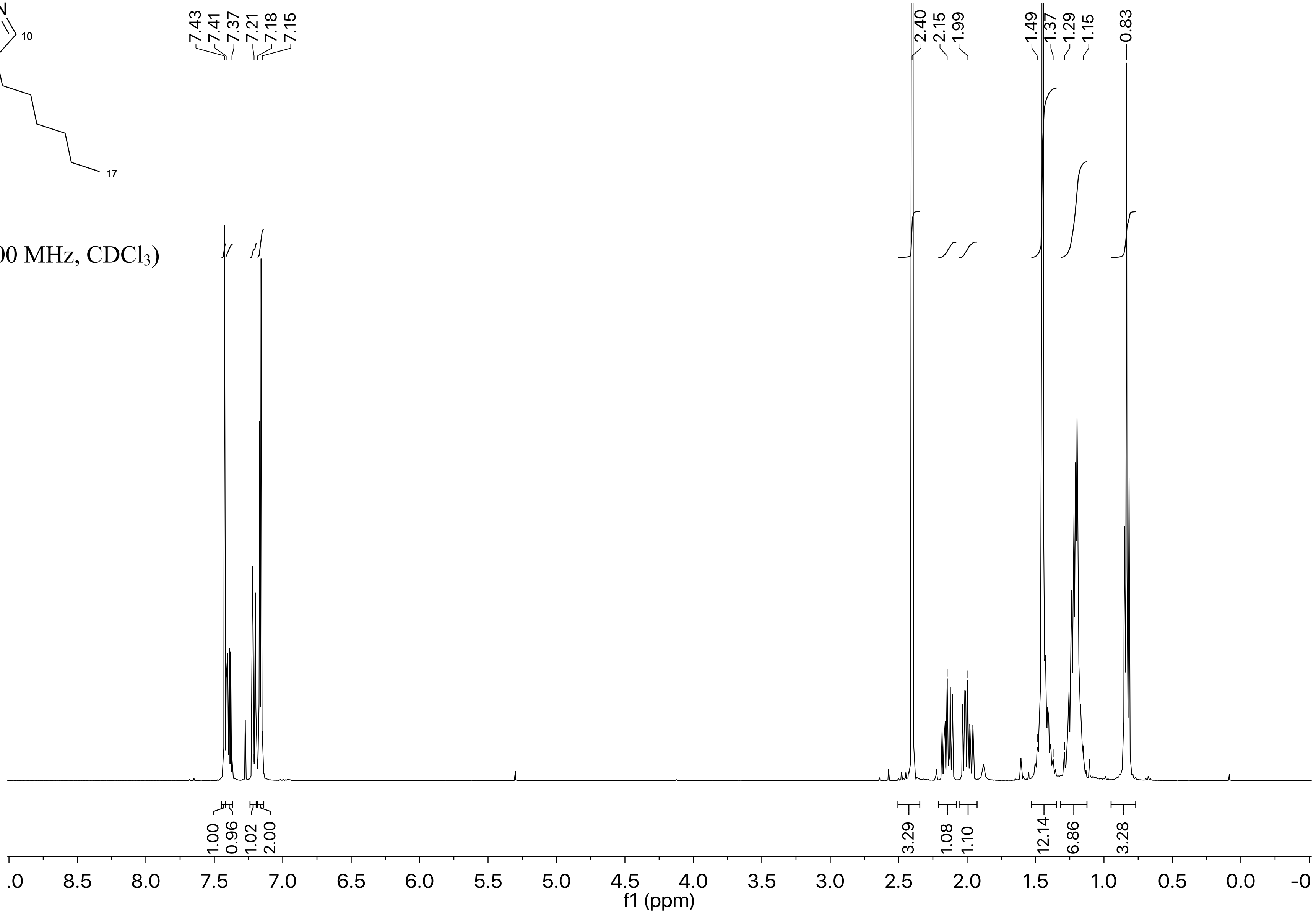

1-(*tert*-Butyl)-4-hexyl-5-(2-(methylthio)phenyl)-1*H*-pyrazole, 7b

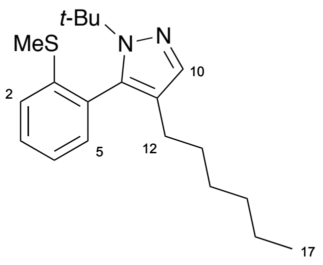

^{13}C NMR (101 MHz, $CDCl_3$)

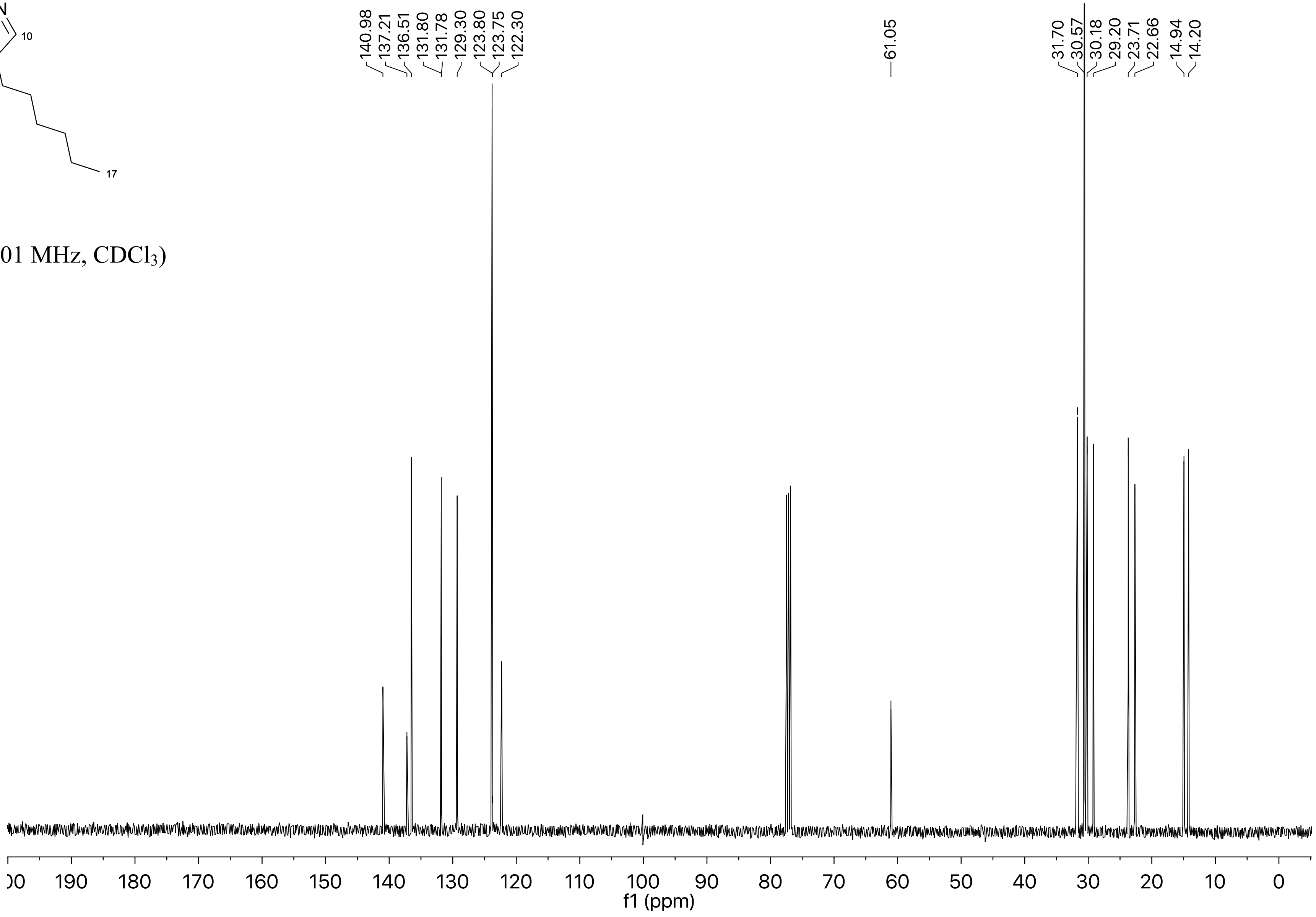

1-(*tert*-Butyl)-4-hexyl-5-(2-(methylthio)phenyl)-1*H*-pyrazole, 7b

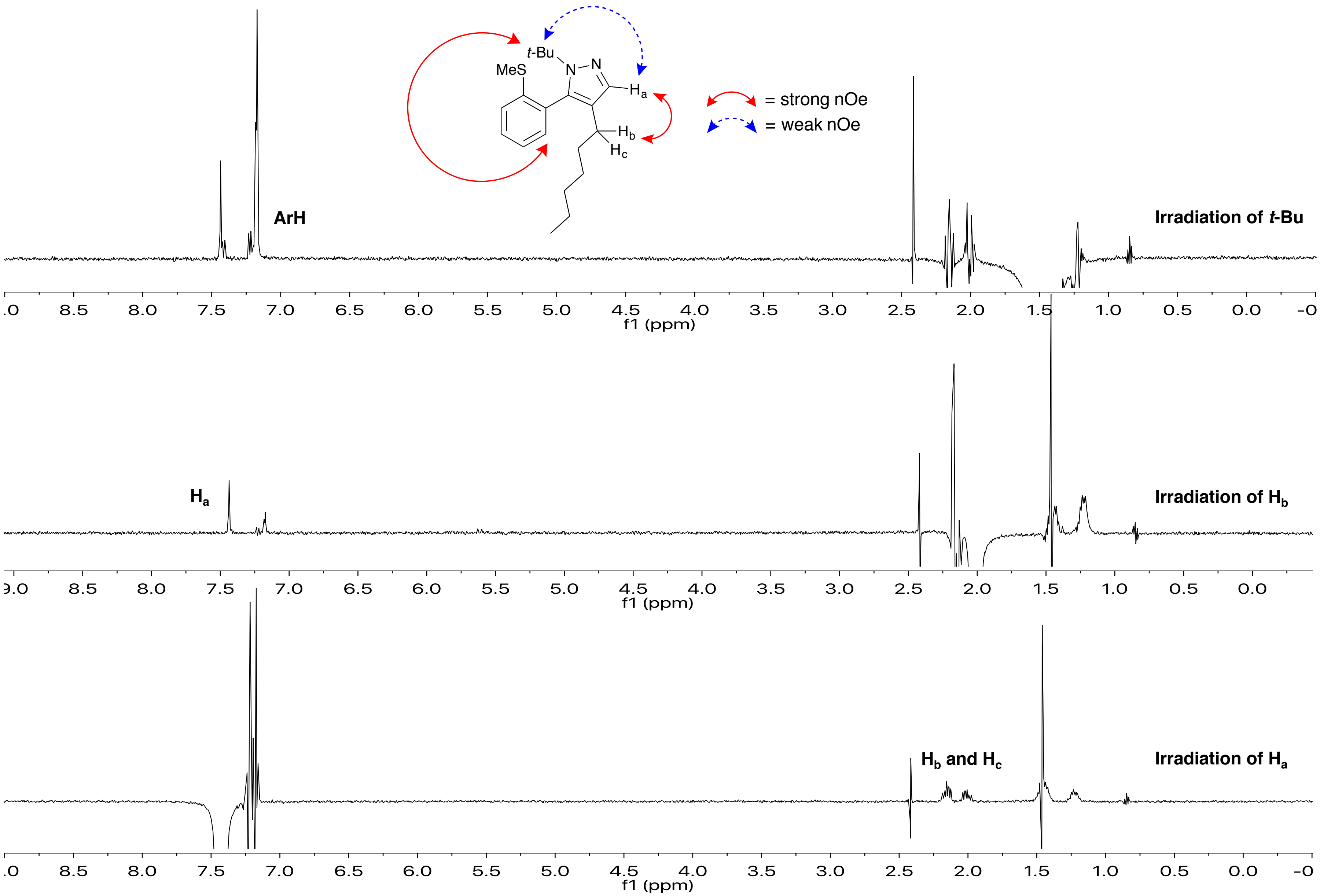

1-Benzyl-4-hexyl-5-(2-(methylthio)phenyl)-1*H*-pyrazole, 7c

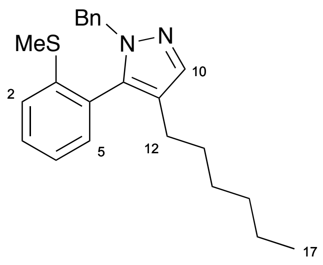

^{1}H NMR (400 MHz, $CDCl_3$)

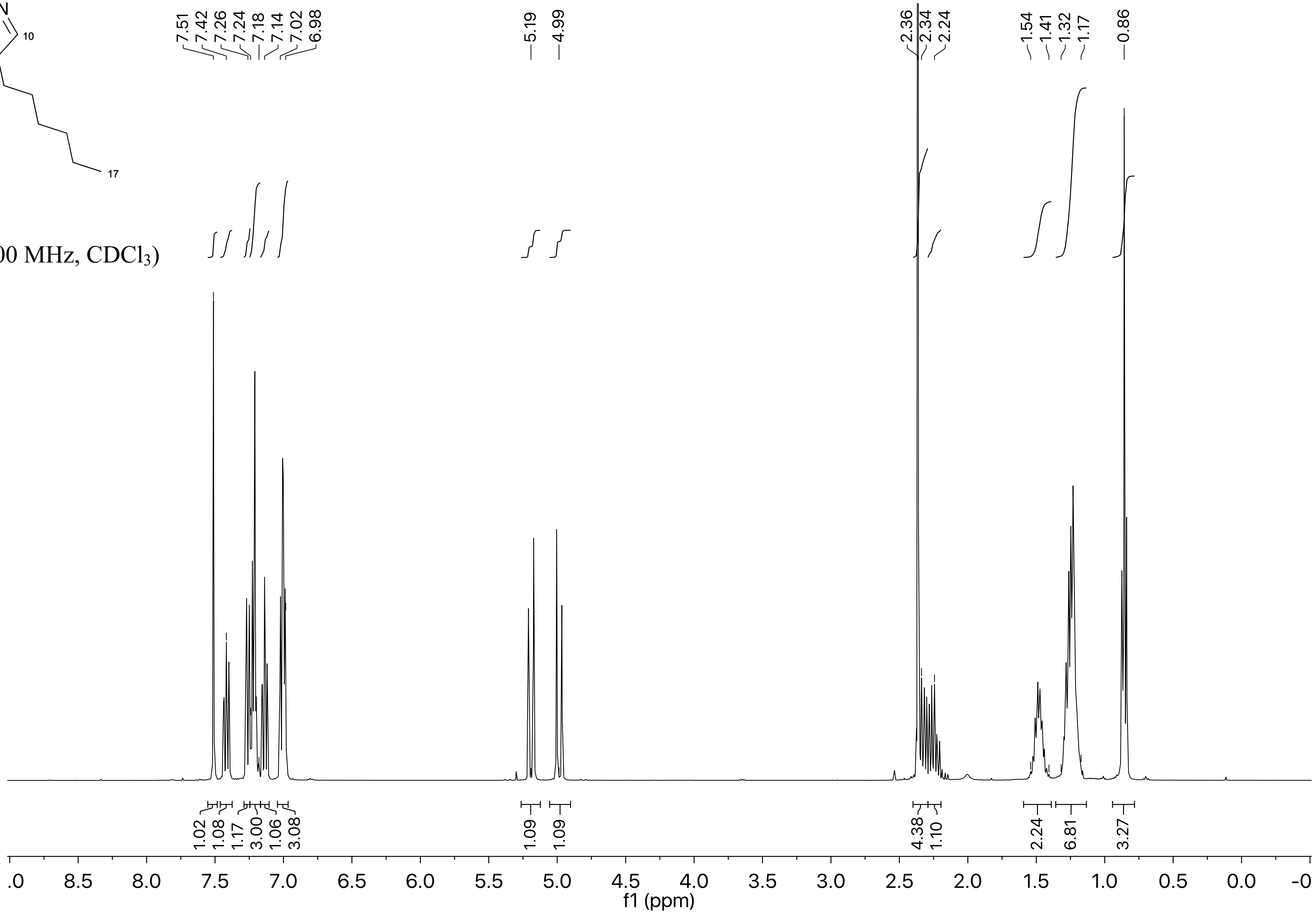

1-Benzyl-4-hexyl-5-(2-(methylthio)phenyl)-1*H*-pyrazole, 7c

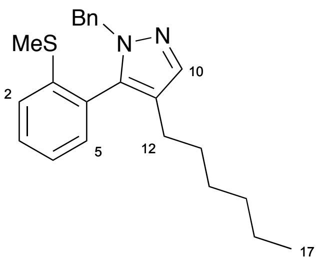

^{13}C NMR (101 MHz, $CDCl_3$)

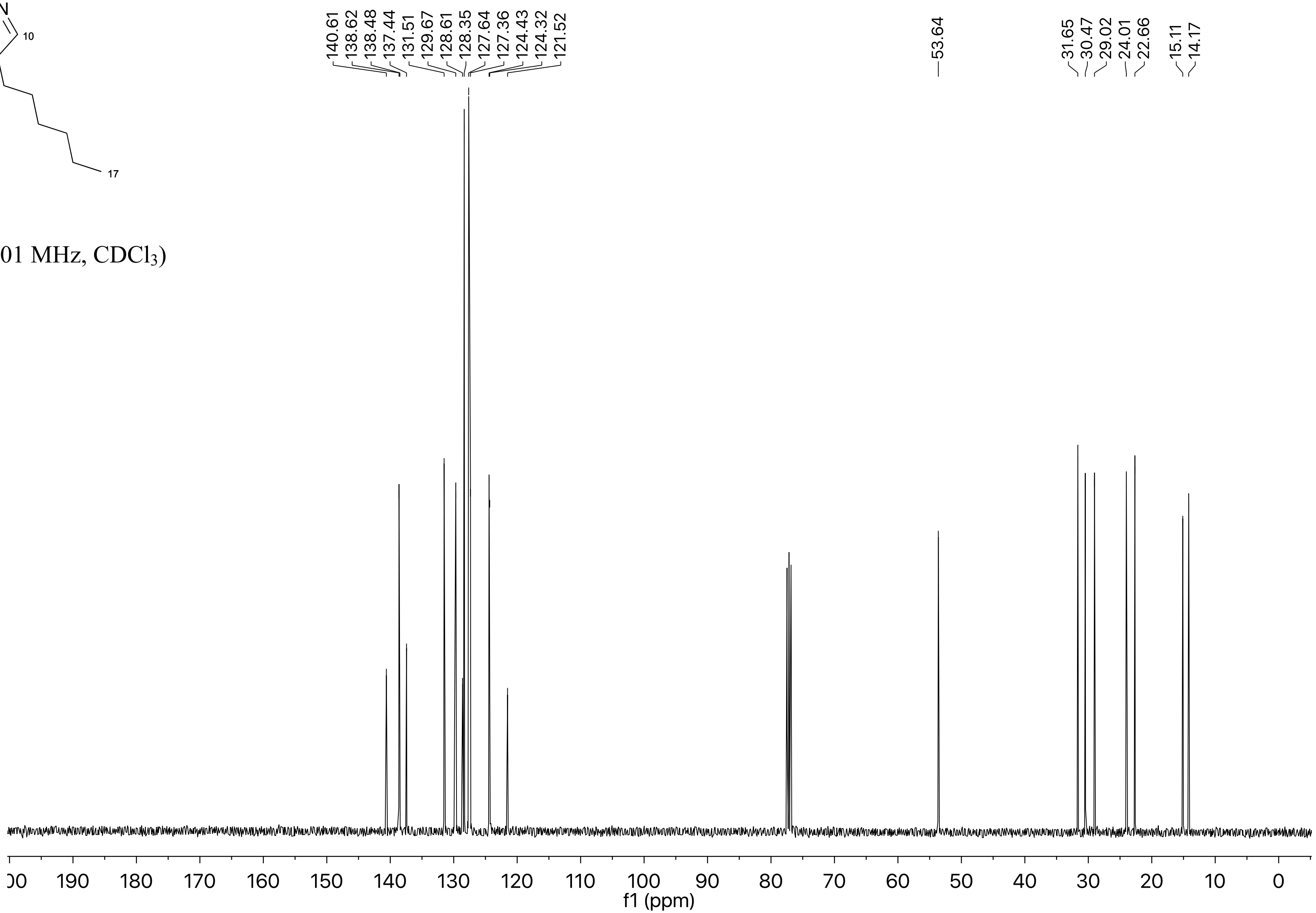

5-Hexyl-4-(2-(methylthio)phenyl)-2-phenylpyrimidine, 8a

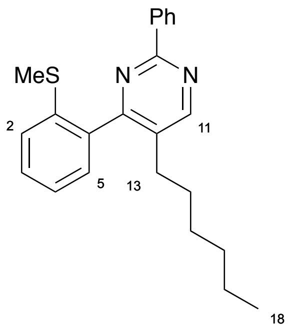

^{1}H NMR (400 MHz, $CDCl_3$)

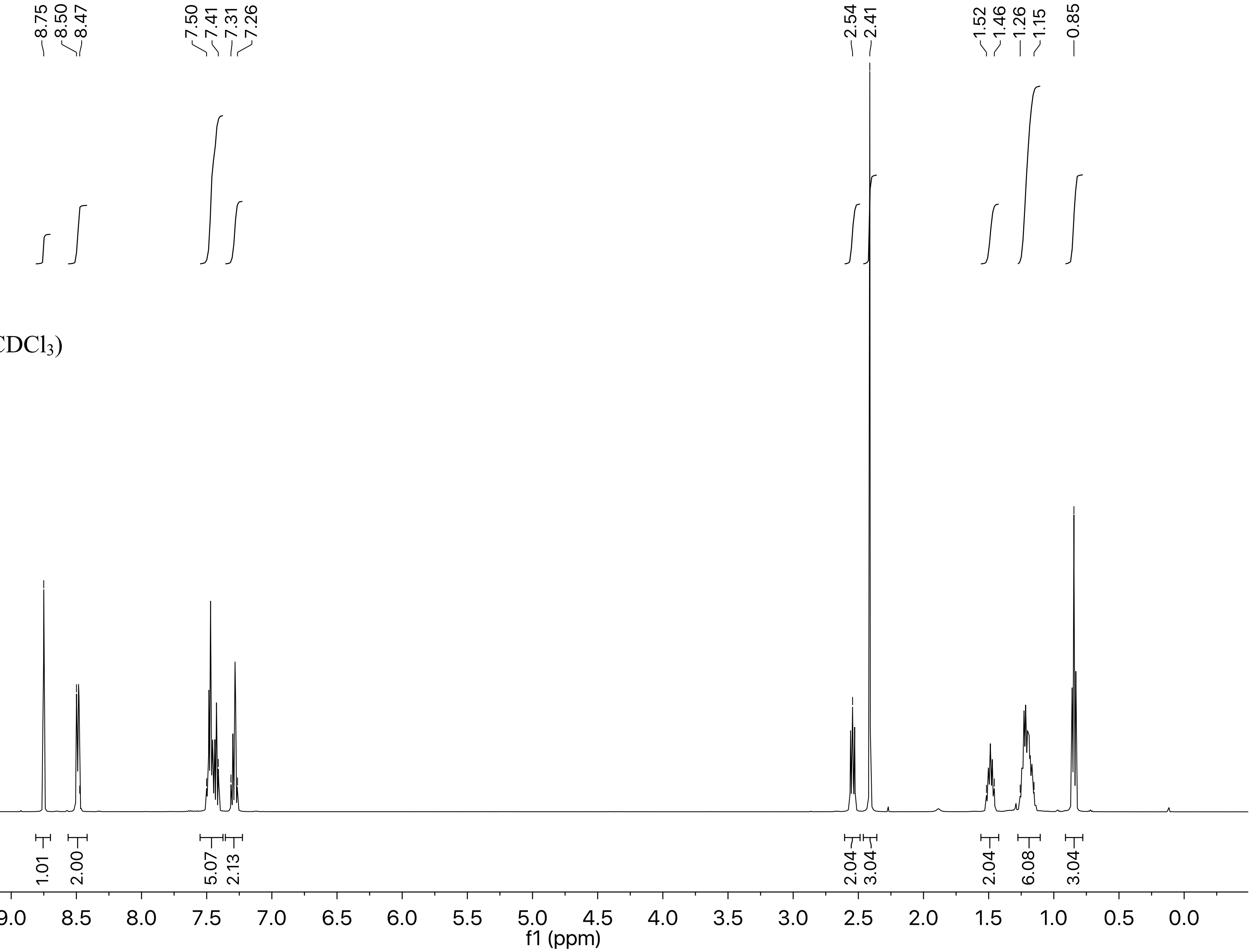

5-Hexyl-4-(2-(methylthio)phenyl)-2-phenylpyrimidine, 8a

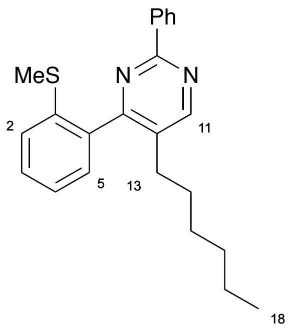

^{13}C NMR (101 MHz, $CDCl_3$)

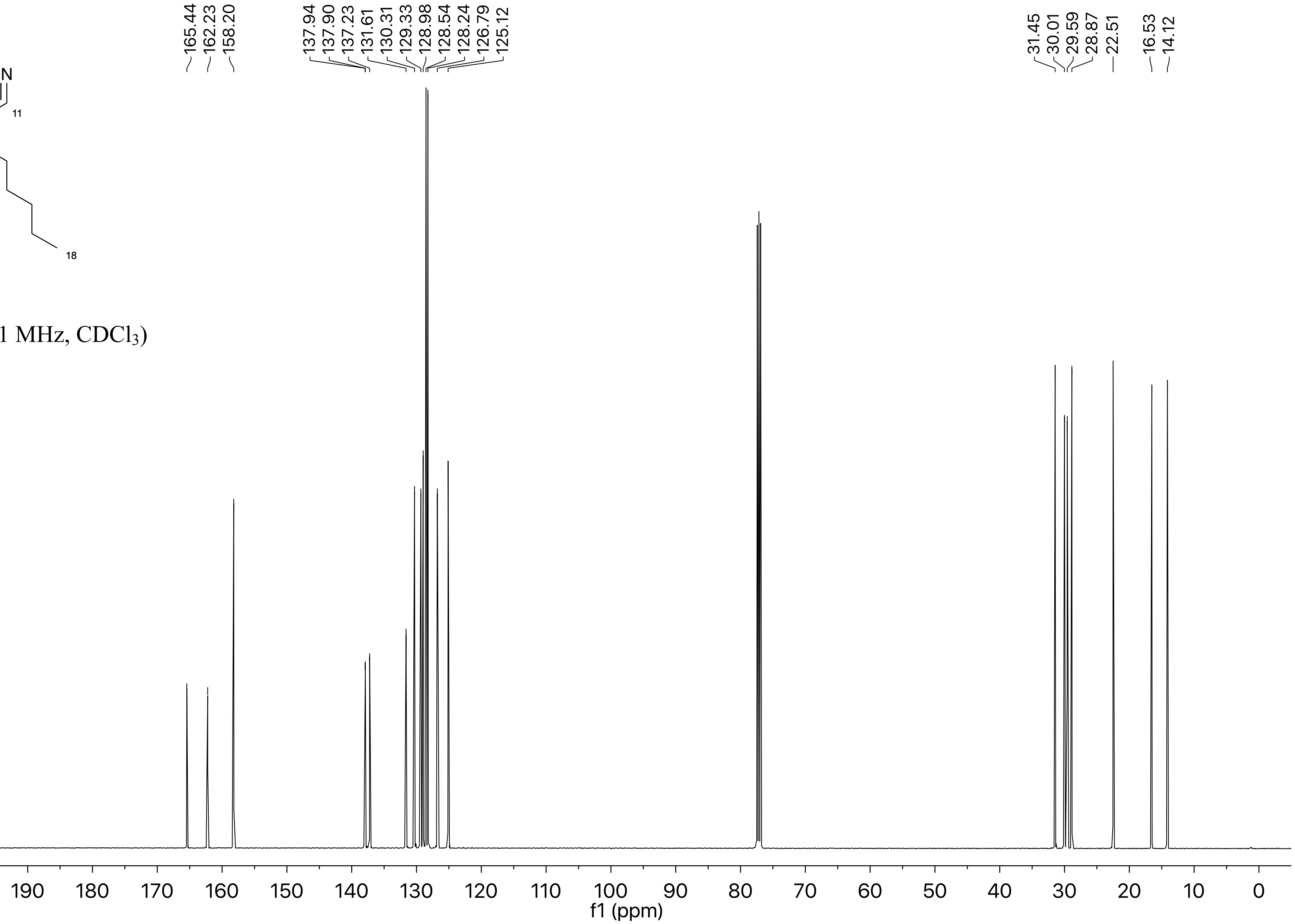

5-Hexyl-4-(2-(methylthio)phenyl)pyrimidin-2-amine, 8b

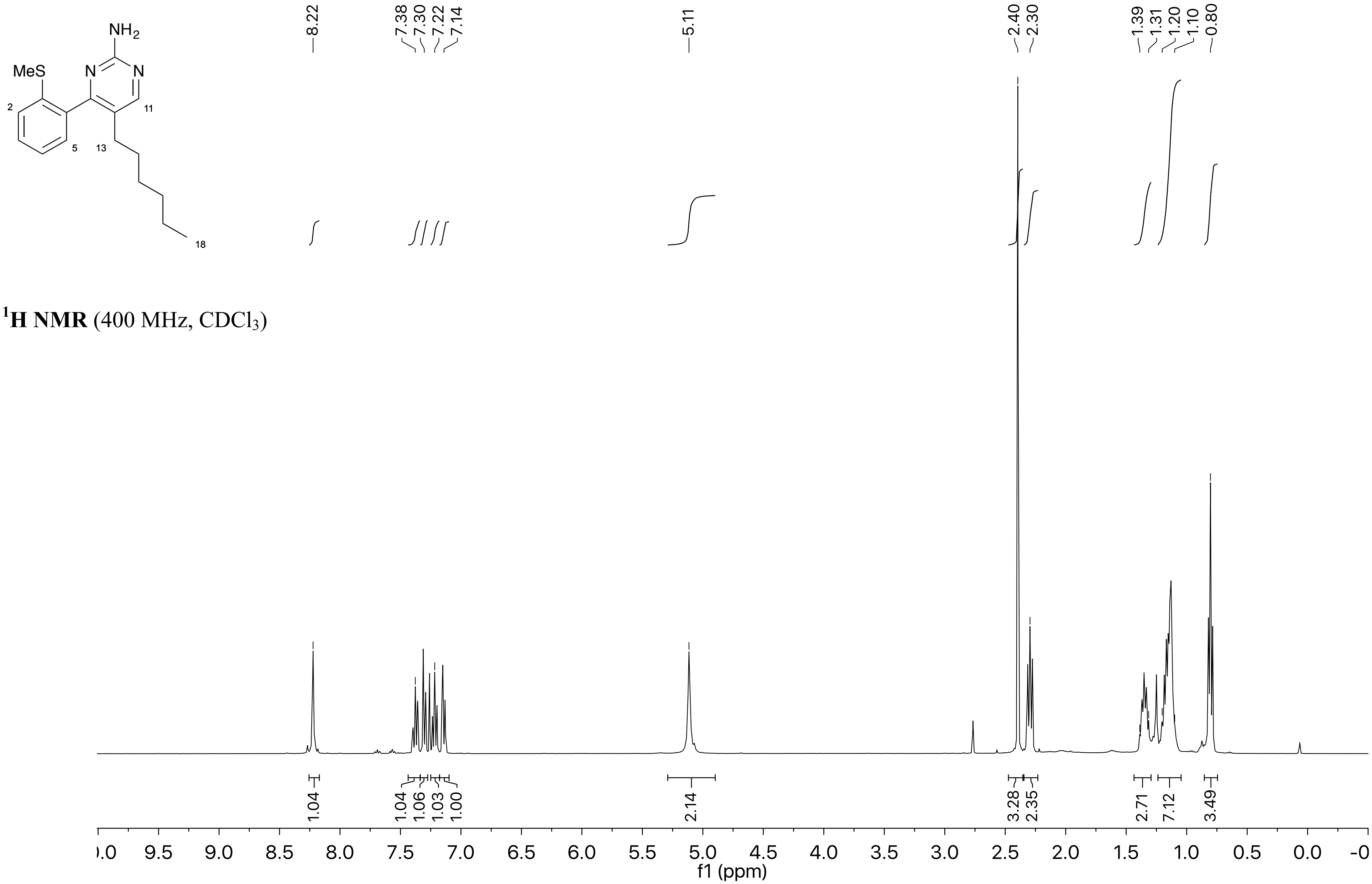

^{1}H NMR (400 MHz, $CDCl_3$)

5-Hexyl-4-(2-(methylthio)phenyl)pyrimidin-2-amine, 8b

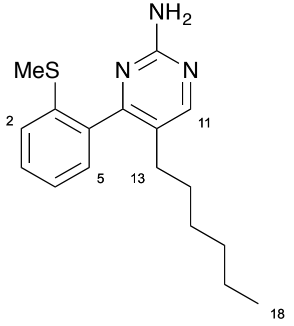

^{13}C NMR (101 MHz, $CDCl_3$)

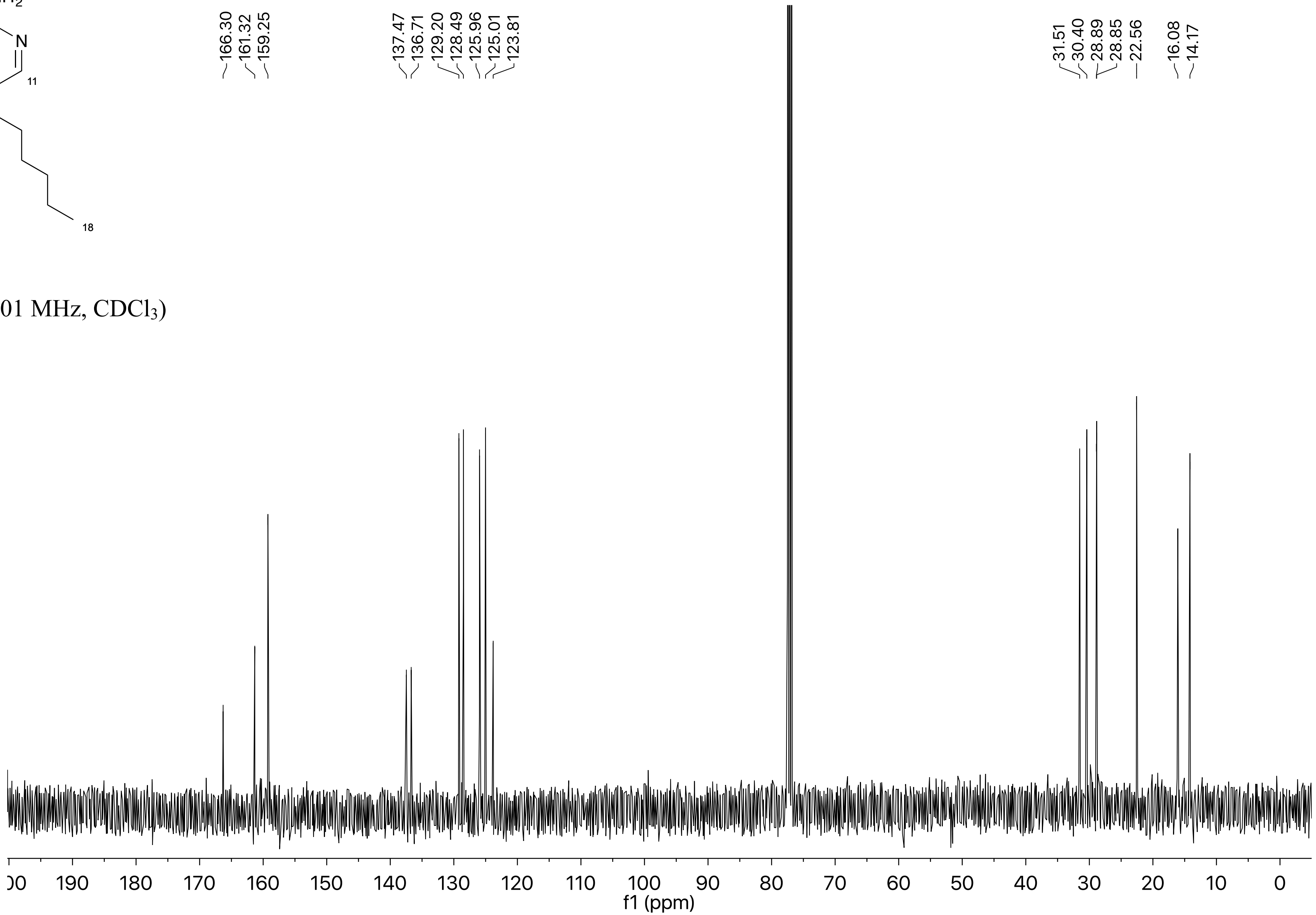

5-Hexyl-4-(2-(methylthio)phenyl)-*N*-phenylpyrimidin-2-amine, 8c

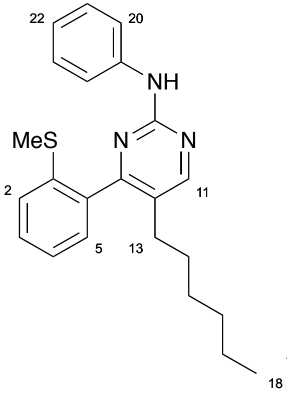

^{1}H NMR (400 MHz, $CDCl_3$)

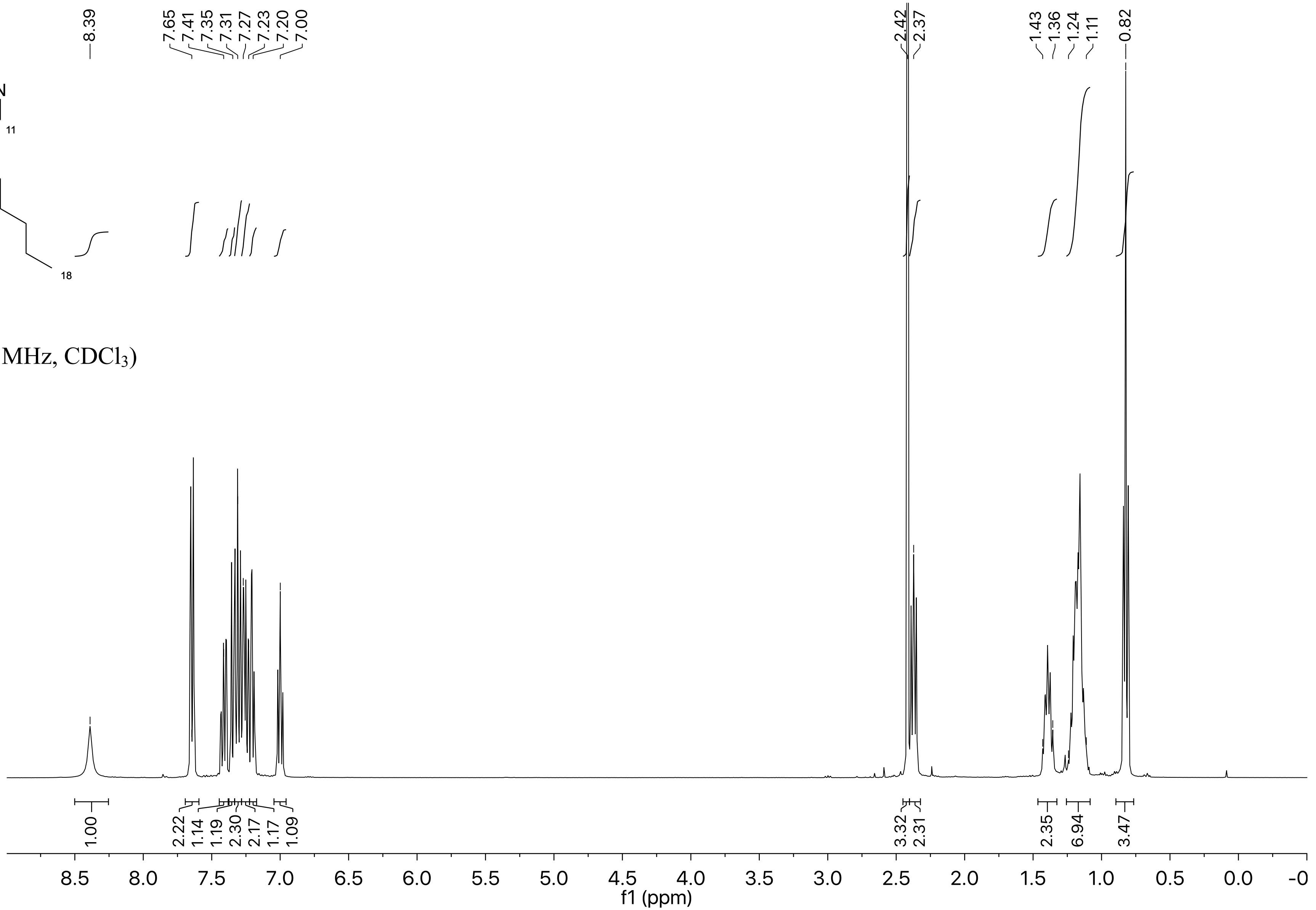

5-Hexyl-4-(2-(methylthio)phenyl)-*N*-phenylpyrimidin-2-amine, 8c

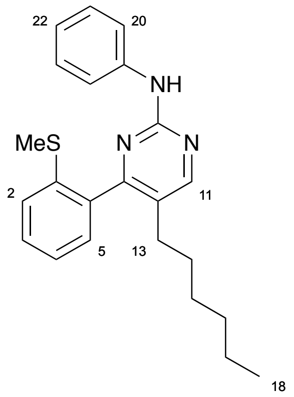

^{13}C NMR (101 MHz, $CDCl_3$)

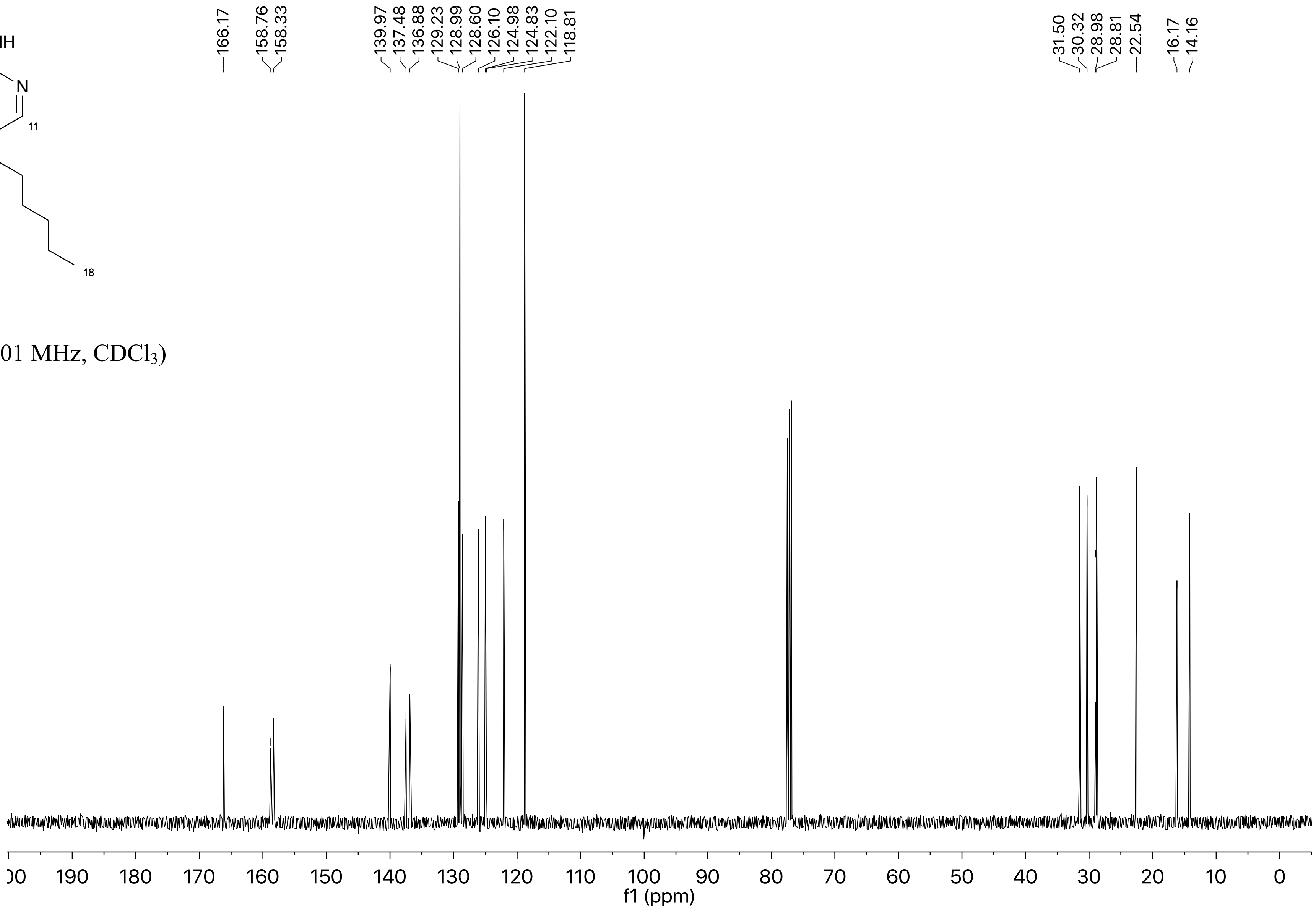

Supplement: Supplementary file 1 [file SC-008-C7SC03795C-s001.pdf]
